# Additive strongly-coordinated solvation structure towards high-voltage 600 Wh/kg-class lithium metal pouch cell

**DOI:** 10.1038/s41467-026-77095-x

**Published:** 2026-08-24

**Authors:** Zhaoxia Zhang, Qian Chen, Bixuan Li, QingWei Zhai, Xiaokang Gu, Chundi Wei, Moxuan Wang, Yuying Jiao, Zhou Zhou, Qiannan Zhang, Andrew Xiang, Chunqiao Jin, Peng Kang, Pengbo Zhai, Kai Liu, Yongji Gong

**Affiliations:** 1 Tianmushan Laboratory, Hangzhou, China; 2 School of Materials Science and Engineering, Beihang University, Beijing, China; 3 State Key Laboratory of Artificial Intelligence for Materials Science, Beihang University, Beijing, China; 4 Jinan Zhongruitai New Material Technology Co. Ltd, Jinan, China; 5 Department of Chemical Engineering, Tsinghua University, Beijing, China; 6 Center for Micro-Nano Innovation, Beihang University, Beijing, China; 7 Key Laboratory of Intelligent Sensing Materials and Chip Integration Technology of Zhejiang Province, Hangzhou, China; 8 Analytical & Testing Center, Beihang University, Beijing, China

**Keywords:** Batteries, Batteries, Batteries

## Abstract

Anions involved solvation structures have been widely designed to construct inorganic enriched interfaces to enable wide voltage window, which, however, sacrifices the anions and effective Li^+^ conductivity. Here we propose an additive-coordinated solvation structure of Li^+^, with the preferred sacrifice of additive rather than anions, to realize stable cycling of 600 Wh kg⁻¹-scale Li metal batteries. Leveraging the molecular design of additives, even a trace amount addition allows for its coordination within the inner sheath of Li^+^ solvation structure. This not only attenuates the excessive consumption of anions and solvent molecules, but also facilitates the formation of robust protective interface. Benefiting from these advantages, a 550.7 Wh kg⁻¹ Li | |LiNi_0.8_Co_0.1_Mn_0.1_O_2_ pouch cell maintains 80.0% capacity retention over 180 cycles at 0.1 C charge and 0.5 C discharge rate. Furthermore, the paired Li | | lithium-rich manganese-based layered oxide pouch cell achieves a specific energy of 602.5 Wh kg⁻¹, sustaining 80% capacity retention for 60 cycles at 0.1 C charge/discharge rate, thus validating the suitability of additive coordinated solvation structure.

## Abstract (web summary)

Lithium metal batteries can achieve high specific energies, however, electrolyte design for lithium metal batteries often sacrifice anions and conductivity. Here, authors design an electrolyte that preferentially sacrifices additives to construct robust electrode interfaces, leading to stable cycling in a 602.5 Wh kg-1 Li | |LRMO pouch cell.

## Introduction

**Fig. 1:**
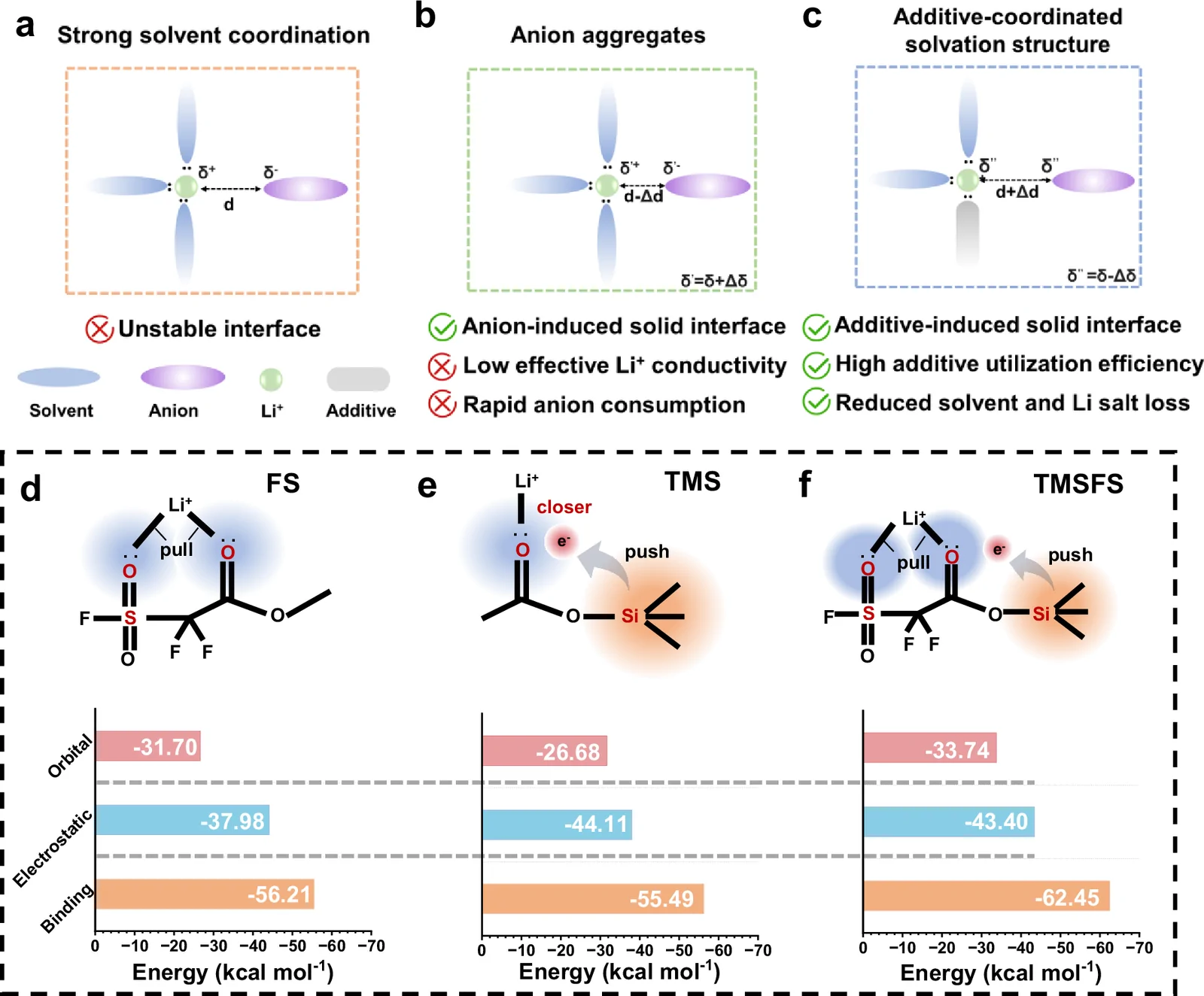
Schematic illustrations of the solvation structure. **a** Strong solvent coordination **b** anion aggregates and **c** additive-coordinated solvation structure; The molecular structures and EDA analysis of **d** FS, **e** TMS, and **f** TMSFS molecules.

Since current lithium (Li)-ion batteries based on graphite anodes are approaching their specific energy limit of ~350 Wh kg^−1^, lithium metal batteries (LMBs) pairing Li metal anodes (LMAs) with high-voltage (≥4.5 V vs. Li/Li⁺) positive electrodes, such as lithium-rich manganese-based layered oxides (LRMOs), have attracted increasing attention for achieving significant breakthroughs of 600 Wh kg^−1^ scale^1^. However, the practical application of high-voltage LMBs is plagued by the short cycle life and serious safety concerns that result from the side reactions at the negative electrode and the positive electrode interfaces due to the formation of unstable solid-electrolyte interphase (SEI)/positive electrode-electrolyte interphase (PEI)^2^. Recent studies reveal that generally, organic species (e.g., ROCO_2_Li, -(CH_2_CH_2_O)_n_-) in electrode/electrolyte interphase layers derived from ester-based solvent molecules suffer from poor passivation capability, mechanical fragility, and sluggish Li⁺ conductivity, which exacerbate Li dendrite growth, continuous electrolyte decomposition, and rapid capacity degradation, especially under high-voltage condition^3^ (Fig. 1a). It is therefore pivotal to modify the electrolyte formulations to construct high-quality interfacial layers and improve the electrochemical stability of the high-voltage LMBs.

Various solvent or Li salt engineering approaches have been proposed to improve the cyclability of high-voltage LMBs, including weak solvent design, functional group substitution, (localized) high-concentration electrolytes, and high-entropy solvents/salts mixing^4–7^. Part of these strategies are based on a principle that involves constructing Li⁺ solvation structures with enhanced Li⁺–anion interactions, such as complete ion pair (CIP) and aggregated solvation cluster (AGG) configurations^8^. These structures facilitate the formation of an inorganic-rich electrode/electrolyte interphase (EEI) on both the negative electrode and positive electrode, thereby enhancing electrode protection under high-voltage conditions^9,10^. Introducing functional additives constitutes another widely-adopted strategy to optimize interphase composition and extend battery cycle life in practical scenarios. Numerous studies have reported the utilization of multiple additives to enhance the interaction between Li⁺ and anions, thus increasing the proportion of anions within the solvation structure (Fig. 1b)^11,12^.

These design of anion-dominated solvation structures can effectively enhance interfacial stability, thus resulting in improved battery performance. However, the significant Li-anions interaction will lead to the formation of molecule clusters, resulting in increased viscosity and hydrodynamic radius, which substantially reduces the ion conductivity^13^. Moreover, anion depletion constitutes a critical failure mechanism in high-specific-energy LMBs^14^. As for additives, their low concentration (<5 wt%) leads to rapid depletion during the initial cycle phase, thereby limiting subsequent performance improvement^15^. This arises from the inability of additive molecules to penetrate the inner solvent sheath, as their binding affinity with Li⁺ is weaker than that of the solvent molecules. Consequently, their contribution to interface formation is diminished during the initial cycle. Therefore, it is urgently demanded to develop an additive coordinated solvation structure (ACSS) implemented through a highly coordinating multifunctional additive that can integrate into inner sheath of Li⁺ even at low concentrations, thereby fully utilizing the film-forming capability of the additive and mitigating the rapid consumption of solvent molecules and anions (Fig. 1c).

Here, trimethylsilyl-2,2-difluoro-2-(fluorosulfonyl) acetate (TMSFS) with strong coordinating effects was designed by integrating push-pull interactions across distinct functional group within the molecule. Specifically, the TMSFS molecules can partially occupy the coordination sites originally bound by the ester solvents, thereby improving the efficacy of the additive and facilitating the formation of an inorganic-rich EEI layer. Moreover, this strongly additive coordinating effect can significantly weaken Li^+^-anion interaction, thereby reducing the consumption of anions by more than 60% after cycling, ultimately contributing to an extended battery cycle life. Thus, even with a low addition of 1 wt% in the electrolyte, Li||LRMO full cell exhibited 70% capacity retention after 850 cycles at high-voltage of 4.8 V and 1.0 C. The practical 550.7 Wh kg^−1^ Li||LiNi_0.8_Co_0.1_Mn_0.1_O_2_ (NCM811) pouch cell exhibited long cycle life surpassing 180 cycles with 80% capacity retention at 4.5 V. Further paired with high-voltage positive electrode, the Li||LRMO pouch cell using the ACSS electrolyte demonstrated a high reversible specific energy of 602.5 Wh kg^−1^ and achieved a stable cycling performance exceeding 60 cycles with 80% capacity retention. This design sparks critical insights on the additive based solvation structure regulation for advancing the electrochemical performance of high-voltage LMB systems.

## Results and discussion

### Molecular design and characterization of ACSS electrolyte

The low concentration and relatively weak Li⁺ interaction of conventional additives hinder their effective involvement in solvation structure regulation, leading to inadequate high-voltage tolerance and limited electrochemical performance improvement. To realize ACSS electrolyte, we drew inspiration from the stable multidentate coordination rings (e.g., 1,2-dimethoxyethane, DME) to enhance the interaction between Li⁺ with additive^16^. Guided by this rationale, Methyl 2,2-difluoro-2-(fluorosulfonyl)acetate (FS) with EEI-forming function was initially identified. The α-hydrogen sites of acetate group are eliminated through fluorine substitution, thereby effectively suppressing the Claisen ester condensation reactions (CECR) and promoting the formation of fluorinated interfaces layer^17^. Electrostatic potential (ESP) maps reveal high electron density at the carbonyl oxygen (−22.52 kcal mol⁻¹) and sulfonyl oxygen atoms (−18.30 kcal mol⁻¹). This pronounced negative region exerts a strong pull effect on Li⁺, enabling the formation of thermodynamically stable six-membered chelate rings. Energy decomposition analysis (EDA) reveals that the FS ligand exhibits substantial orbital interaction energy (−31.70 kcal mol⁻¹) and electrostatic interaction energy (−37.98 kcal mol^-1^), collectively accounting for the overall Li⁺–FS binding energy of −56.21 kcal mol^−1^. However, this binding energy remains insufficient to compete with conventional solvents, hindering its ability to enter the inner solvation sheath of Li^+^(Fig. 1d and Supplementary Fig. S1a). The key to enhance Li⁺ coordination lies in increasing the electron density of the carbonyl oxygen^18^. This prompts the strategic introduction of a bulky, nonpolar functional group adjacent to the carbonyl moiety, leveraging its electron-donating effect to strengthen Li⁺ interactions. Therefore, trimethylsilyl with a large, nonpolar electron-donating group was selected as an ideal candidate. As shown in Fig. 1e and Supplementary Fig. S1b, the trimethylsilyl group of trimethylsilylacetate (TMS) exerts a strong electron‑pushing effect to the C = O, elevating its ESP to as low as −35.52 kcal mol^−1^, which demonstrates the validity of this design. However, this type of structural configuration featuring a single C=O coordination is incapable of forming strong and stable coordination with Li^+^ ions, which is confirmed by the low orbital (−26.68 kcal mol^−1^) and binding energy (−55.49 kcal mol^−1^), thereby limiting its ability to penetrate into the first solvation sheath.

Based on the above discussion, the electronic effect among different functional groups can be leveraged to rationally design additives with stable and strong coordinating ability. Specifically, trimethylsilyl group was chosen to replace alkyl chains of FS to further enhance the interaction between additive with Li⁺. The designed TMSFS exhibited higher electron cloud density and lower ESP value at the C=O (−24.71 kcal mol^−1^) and S = O (−19.31 kcal mol^−1^) groups (Fig. 1f and Supplementary Fig. S1c). The leading natural orbitals for chemical valence (NOCV) deformation densities for Li^+^–TMSFS show that electron-density rearrangement upon binding is localized predominantly on the carbonyl and sulfonyl oxygen atoms and around Li^+^. Very similar NOCV patterns are observed for Li⁺–FS, indicating that in both systems Li^+^ is coordinated by the same O-rich pocket defined by the carbonyl and sulfonyl groups (Supplementary Fig. S2). The trimethylsilyl group exhibits strong electron-donating character, while the electron-withdrawing nature of the –CF_2_–SO_2_F– moiety concentrates negative charge on the sulfonyl and carbonyl oxygen atoms. These complementary electronic effects synergistically elevate both the orbital energy (−33.74 kcal mol⁻¹) and the electrostatic energy (−43.40 kcal mol⁻¹). As a result, TMSFS achieves the highest Li⁺ binding energy among the reported candidates (−62.5 kcal mol⁻¹), fully satisfying the design criteria for the ACSS electrolyte (Supplementary Fig. S3).

**Fig. 2:**
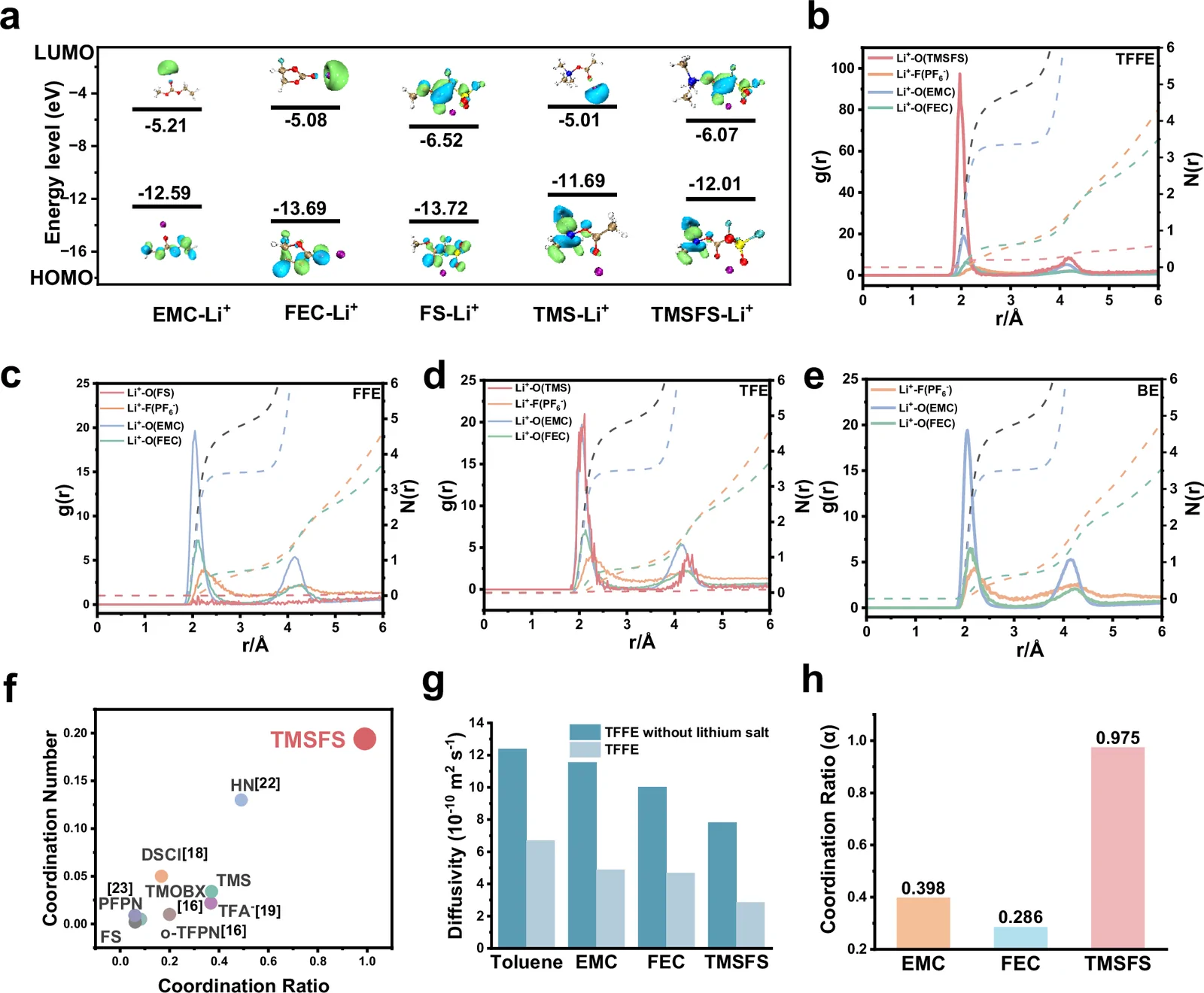
Theoretical and spectroscopic analysis of additive-Li^+^ interaction. **a** LUMO/HOMO energy level of different solvents with Li^+^, Purple: Li⁺; red: O; brown: C; gray: H; blue: Si; yellow: S; green: F; RDFs of key species coordinating with Li⁺ in **b** TFFE, **c** FFE, **d** TFE and **e** BE systems, solid line represents g(r), dashed line represents N(r); **f** Comparison of coordination numbers of additives with some recent relevant reports, the source of the literature data shown in this figure can be found in Supplementary Table S1; **g** Diffusion-ordered spectroscopy NMR (DOSY-NMR) analysis. The diffusivities of multiple components (toluene, EMC, FEC, TMSFS) in TFFE and TFFE without LiPF_6_. Toluene was used as an internal reference (**h**) corresponding coordination ratios calculated through DOSY.

To gain deeper insights into how TMSFS modulates the Li⁺ solvation environment, theoretical calculations were first investigated to compare baseline electrolyte (BE, 1 M LiPF_6_ dissolved in EMC: FEC = 4:1 (by volume)), TMS formulated electrolyte (TFE), FS formulated electrolyte (FFE) and TMSFS formulated electrolyte (TFFE) systems. Moreover, the energy levels of the lowest unoccupied molecular orbital (LUMO) and the highest occupied molecular orbital (HOMO) were also calculated according to molecular orbital theory^19^. As depicted in Fig. 2a, TMSFS-Li^+^ exhibited the highest HOMO energy level (−12.01 eV) and the lowest LUMO energy level (−6.07 eV) compared to EMC-Li^+^ and FEC-Li^+^. To verify whether the rational design of TMSFS translates into the desired oxidation behavior, frontier orbital and spin-density analyses were performed (Supplementary Fig. S4). The results indicate that oxidation is preferentially localized on the trimethylsilyl moiety, as evidenced by singly occupied molecular orbital (SOMO) localization and dominant spin accumulation on the Si-containing fragment, with negligible contribution from the fluorosulfonyl group, thereby favoring the formation of beneficial interfacial oxidation products rather than detrimental decomposition pathways. These results indicated that TMSFS undergoes preferential oxidation and reduction over solvents.

Molecular dynamics (MD) simulation was further extended to analyze the evolution of solvation structures, providing atomistic insights into how TMSFS modifies the coordination environment around Li⁺ ions (Supplementary Fig. S5). Our MD model uses a fixed-charge framework with the OPLS-AA force field, where partial atomic charges are derived from RESP fitting to the DFT electrostatic potential and scaled by an electronic continuum correction (ECC) to account for missing electronic polarization. The proportion of additive molecules within the simulation box model was estimated based on an addition level of 1 wt%. The radial distribution function (RDF) analysis showed that the intensity of the first peak at r≈ Å in the RDF profile of Li^+^ and O atoms of TMSFS (g(r)=97.33) was much higher than that of the TMS and FS, demonstrating the successful molecule design (Fig. 2b–e). Further comparative analysis of the coordination numbers showed that TFFE exhibits less coordinated EMC and PF_6_^−^ (Li^+^EMC_3.307_FEC_0.560_TMSFS_0.194_PF_6_^−^_0.264_) compared to BE system (Li^+^EMC_3.464_FEC_0.591_PF_6_^−^_0.360_), and upon further increasing the TMSFS content to 5 wt% and 10 wt%, CN (TMSFS) increases to 0.820 and 1.389, respectively (Supplementary Fig. S6). This indicates that TMSFS partially replaces EMC and FEC solvent molecules and anions in the first solvation shell of Li⁺, thereby effectively suppressing interfacial side reactions involving solvent molecules and delaying the excessive consumption of anions. Meanwhile, a comparison of the coordination numbers of additives with some recent relevant reports revealed that the TMSFS additive exhibited a relatively high coordination ratio of ~0.99 and strong coordination ability at 1 wt% addition level, demonstrating improved Li⁺ coordination capability of TMSFS (Fig. 2f)^20–27^.

Raman spectra of BE and TFFE also revealed that the incorporation of TMSFS reconstructs the Li⁺ solvation environment^28,29^. As the TMSFS content increased from 0 to 5 wt%, the coordination fractions of FEC, PF_6_^−^, and EMC decreased progressively, from 0.369, 0.397, and 0.487 (0 wt%) to 0.298, 0.345, and 0.416 (1 wt%), and further to 0.231, 0.265, and 0.386 (5 wt%), respectively (Supplementary Figs. S7 and S8), indicating that TMSFS effectively reduced the presence of solvent molecules and anions within the solvation sheath. Meanwhile, internal-referenced diffusion-ordered spectroscopy (IR-DOSY) utilizing toluene as an internal reference was employed to measure the solvent diffusivity before and after the addition of lithium salt. The two-dimensional ^1^H NMR results reveal that upon the addition of the TMSFS, the as-calculated coordination ratio (α) of EMC and FEC decreases from 0.44 and 0.31 in BE to 0.39 and 0.28 in TFFE, respectively. Meanwhile, TMSFS exhibits a relatively high coordination fraction of 0.975. These results further substantiate the strong Li⁺-coordinating capability of TMSFS and its effectiveness in attenuating solvent–Li⁺ coordination (Fig. 2g, h and Supplementary Figs. S9–11)^30^. Detailed deduction of D–α analysis is presented in the method. Also, the effect of TMSFS additive in altering the Li^+^ solvation structure was further confirmed by ^7^Li NMR (Supplementary Fig. S12), where the ^7^Li chemical shift displaced to lower values from −0.56 ppm (BE) to −0.58 ppm after adding 1 wt% TMSFS and further shift to −0.64 ppm as the addition amount increase to 5 wt%. The upfield shift implies the increased shielding effect on the Li^+^ nucleus, resulting from the weakened interaction between Li^+^ with anions^31^. Moreover, due to the reduced formation of large-radius anion aggregates of ACSS, Li^+^ conductivity in the bulk phase was slightly increased from 2.35 mS cm^−1^ to 2.47 mS cm^−1^, demonstrating the advantages of ACSS in enhancing the kinetics of Li^+^ transport (Supplementary Fig. S13).

### Structure characterization of PEI layer of LRMO positive electrodes

**Fig. 3:**
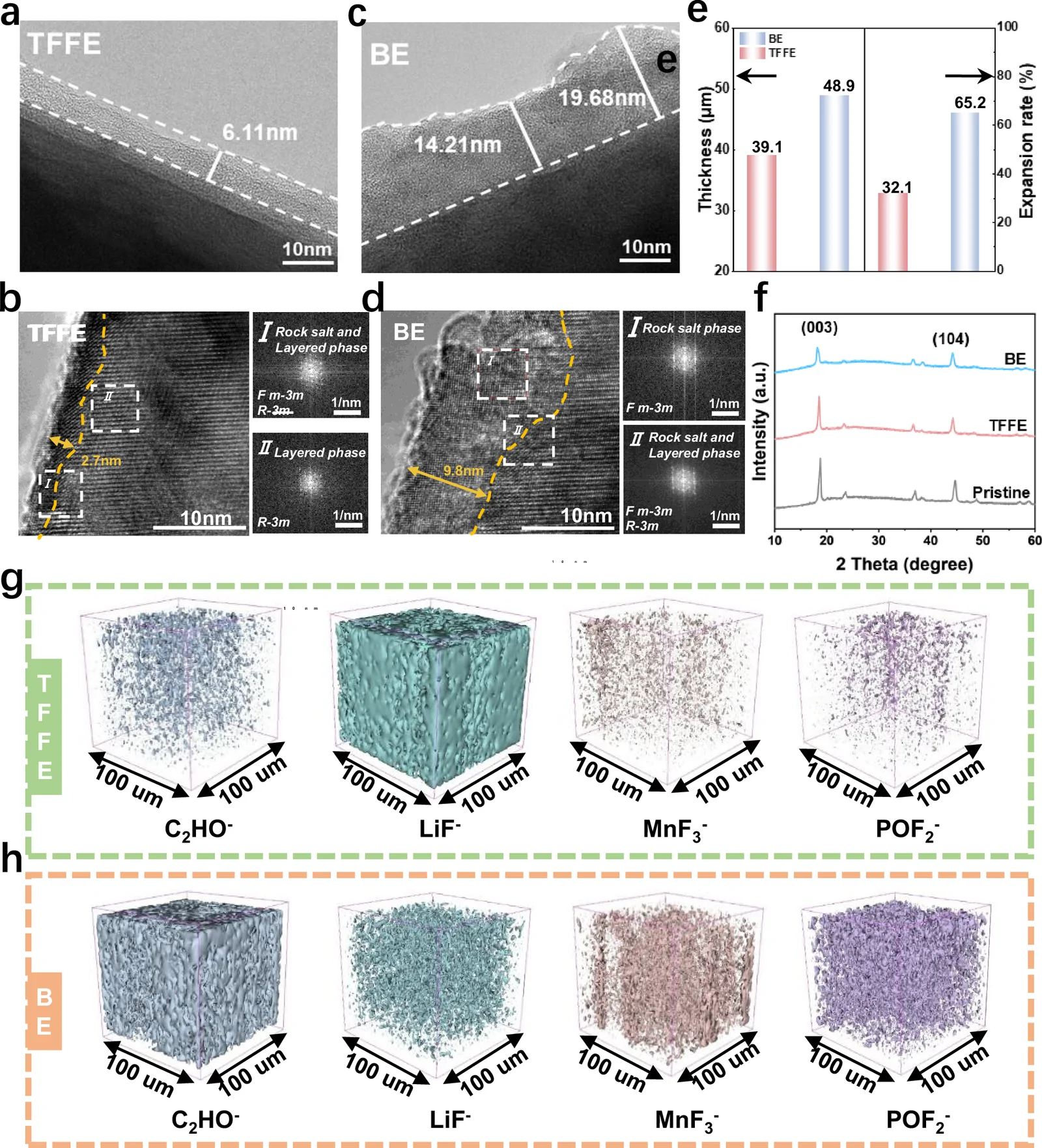
Positive electrode interphase evolution and structural stability. TEM images of the (**a**) PEI layer and (**b**) positive electrode surface from Li||LRMO full cell assembled with TFFE after 100 cycles at 0.9 mA cm^−2^; TEM images of (**c**) PEI layer and (**d**) positive electrode surface of BE counterpart system after 100 cycles at 0.9 mA cm^−2^ (TEM images were taken under different defocus conditions to selectively highlight PEI thickness or surface phase evolution with the corresponding FFT pattern on the right); **e** Thickness variation of the positive electrode after 100 cycles at 0.9 mA cm^−2^; **f** XRD patterns of fresh LRMOs and LRMOs after 100 cycles at 0.9 mA cm^−2^ with BE and TFFE electrolytes; 3D images of sputtering time-dependent of selected secondary ion fragments of different PEI (**g**) TFFE, (**h**) BE after 100 cycles at 0.9 mA cm^−2^. All the tests were performed at 25 °C ± 0.5 °C.

Multi-scale characterization techniques were employed to prove the enhanced PEI layer and suppression of structural phase transitions of post-cycled LRMO positive electrodes. The LRMO particles displayed minimal structural damage and surface defects after 100 cycles in the TFFE electrolyte (Supplementary Fig. S14a). TEM analysis and Fast Fourier Transform (FFT) patterns showed a continuous and uniform PEI layer with a thickness of ~6.11 nm was spotted and that LRMO particles well retained their original layered structure with only a 2.7-nm-thick subsurface region undergoing phase transformation to the rock-salt structure, while the bulk interior kept its pristine layered phase structure (Fig. 3a, b). In sharp contrast, serious cracks could be observed on the LRMO particle in BE electrolyte (Supplementary Fig. S14b), and BE system showed an uneven and much thicker PEI with a thickness over 14 nm (Fig. 3c), indicating phase degradation and a structural transition from a layered to a rock-salt configuration within a near-surface region of 9.8 nm in thickness (Fig. 3d). The cross-section SEM images revealed that the thickness of the positive electrode cycled in TFFE increases from 29.6 ${{\rm{\mu }}}{{\rm{m}}}$ to 39.1 ${{\rm{\mu }}}{{\rm{m}}}$, while the thickness of the positive electrode in BE expands dramatically to 48.9 ${{\rm{\mu }}}{{\rm{m}}}$ (Fig. 3e and Supplementary Fig. S15). To further validate the positive electrode-stabilizing efficacy of ACSS, X-ray diffraction (XRD) analysis was conducted. As illustrated in Fig. 3f, the I (003)/I (104) ratios for pristine LRMO, cycled LRMO from TFFE-based cell and LRMO from BE-based cell are 1.522, 1.469, and 1.149, respectively, suggesting that TFFE mitigated the structural degradation of the positive electrode during cycling process^32^. Conversely, the (003) peak of the BE-based sample shifted to a lower 2θ angle compared to the pristine material, accompanied by peak splitting into two distinct reflections, which indicated the severe irreversible phase transition^33^.

Depth sputtering time-of-flight secondary-ion mass spectroscopy (ToF-SIMS) was performed to investigate the role of ACSS in the formation of the PEI (Supplementary Fig. S16). As shown in Fig. 3g, h, fewer C_2_HO^−^ organic and rich inorganic species, such as LiF^−^, SiOC_2_H_6_^−^ and SO_3_^−^, were uniformly dispersed across the PEI layer of TFFE (Supplementary Fig. S17)^34^. Meanwhile, MnF_3_^−^ (from active mass dissolution products) and POF_2_^−^ (from LiPF_6_ decomposition) were significantly reduced compared to BE system, indicating the protective effect of the TMSFS on the positive electrode and the reduction of lithium salt consumption at the interface^35^. These results indicated that the ACSS effectively suppressed the decomposition of lithium salts and solvents on the interface of electrode, and the resulting ACSS-derived PEI contributed to preserve the structural integrity of positive electrode during extended cycling under high-voltage conditions.

### SEI optimization and reversibility improvement of lithium metal negative electrodes

To systematically evaluate the impact of ACSS-derived SEI on cell performance, Li||Cu half-cell was firstly assembled to investigate the Li metal plating/stripping process. Firstly, electrochemical performance tests were conducted with different additions of TMSFS. As shown in Supplementary Fig. S18, half-cells with 1 wt% TMSFS exhibit the most stable cycling compared with the baseline electrolyte and other additive contents. Cross-sectional FIB–SEM and surface images reveal that increasing the TMSFS concentration leads to a progressively thicker interfacial reaction layer on the Li metal surface (Supplementary Fig. S19), which is attributed to excessive parasitic reactions between TMSFS and Li metal, as reported previously. Such aggravated interfacial reactions inevitably increase the negative electrode impedance and deteriorate reaction kinetics, as evidenced by the EIS results (Supplementary Fig. S20). Therefore, BE + 1 wt% TMSFS was selected to formulate TFFE.

**Fig. 4:**
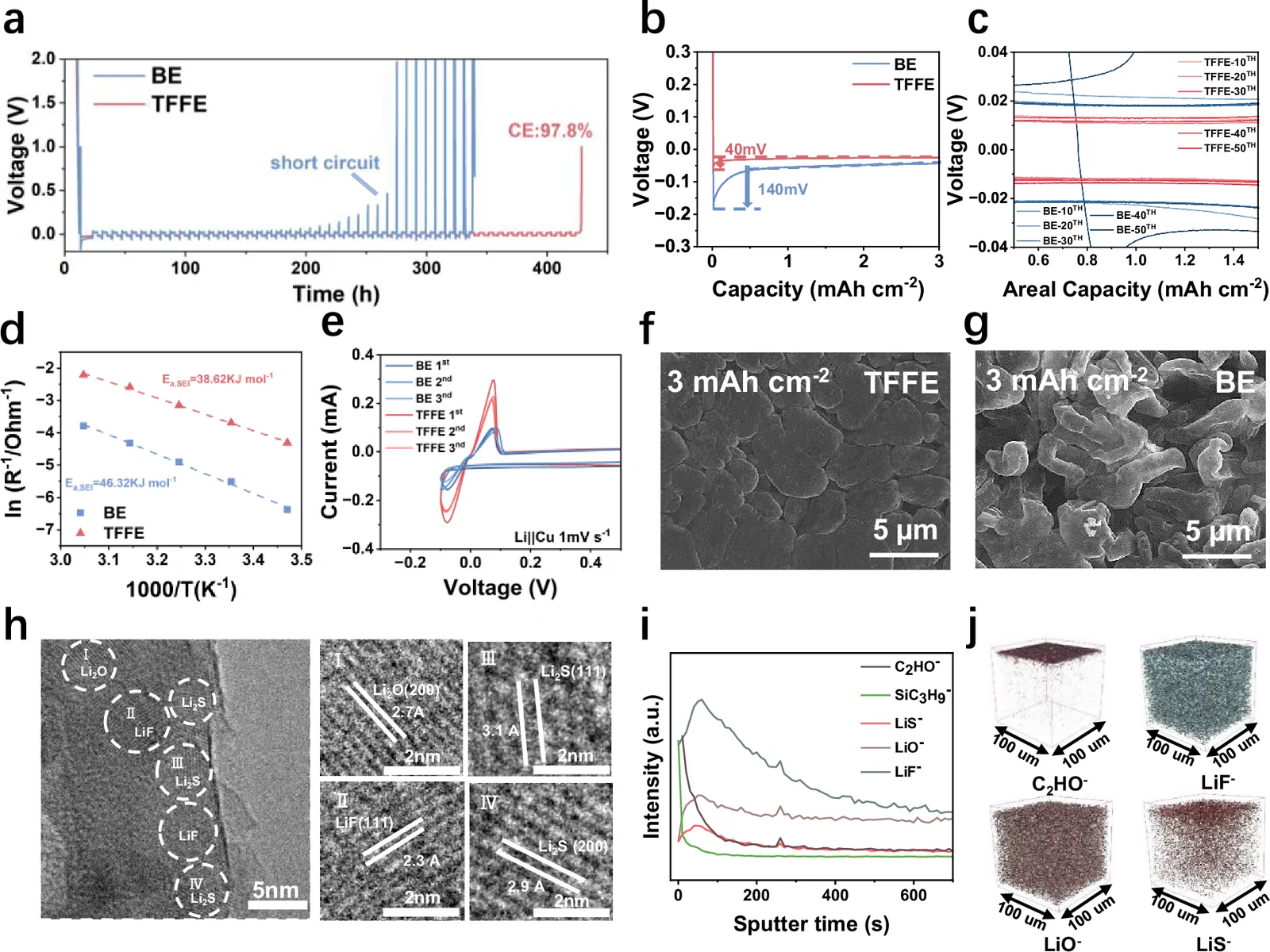
Interfacial chemistry and lithium deposition behavior. **a** Average CE of BE- and TFFE-based Li||Cu half-cell over 50 cycles at 0.5 mA cm^−2^ and 2 mAh cm^−2^; **b** Voltage-areal capacity profiles during the nucleation process for BE- and TFFE-based half-cells; (c) Voltage–areal capacity profiles of TFFE and BE systems at 0.5 mA cm^−2^ and 2 mAh cm^−2^, recorded at different cycle numbers; **d** Arrhenius plots of temperature-dependent Li⁺ transport resistance (R_SEI_) of the as-formed SEI in BE- and TFFE-based Li||Cu half-cells after 3 mAh cm^−2^ of Li depositing on Cu electrodes at 0.5 mA cm^−2^; **e** CV curves of BE- and TFFE-based Li||Cu half-cells at 1 mV s^−1^; SEM images of 3 mAh cm^−2^ of Li depositing on Cu electrodes at 0.5 mA cm^−2^ of **f** TFFE- and **g** BE-based half-cells; **h** Cryo-TEM image of SEI layer derived from TFFE after 0.5 mAh cm^-2^ of Li depositing on Cu electrodes at 0.5 mA cm^−2^, the test was performed at −170 °C to preserve the native SEI structure and minimize beam damage; **i** Sputtering time-dependent compositional profiles of selected secondary ion fragments of different SEI component after 3 mAh cm^−2^ of Li depositing on Cu electrodes at 0.5 mA cm^−2^and **j** corresponding 3D images.

At current density of 0.5 mA cm^−2^ with a fixed areal capacity of 2 mAh cm^−2^ (Fig. 4a), the cell using the TFFE achieved an average Coulombic efficiency (CE) of 97.8% over 50 cycles, whereas the counterparts using the BE failed after 30 cycles, the FFE achieved a lower average CE of 96.47%, and the TFE failed after 40 cycles due to severe polarization (Supplementary Fig. S21). Notably, during initial Li plating, the TFFE system exhibited the lowest overpotential of 40 mV, while the counterparts showed an overpotential of 140, 80, 105 mV (BE, FFE, TFE, respectively) (Fig. 4b and Supplementary Fig. S22). The voltage profiles also demonstrate that the TFFE-based cell maintained stable Li plating/stripping with low polarization (<12 mV), whereas the BE-based cell exhibits higher polarization (>20 mV) and failed at approximately 50 cycles (Fig. 4c). These results demonstrated that the ascendent efficacy ACSS in inducing the formation of stable SEI layer and promoting reversible Li plating/stripping process. The symmetrical cell assembled with TFFE electrolyte demonstrated stable and reversible Li plating/stripping behavior for over 2000 hours at a current density of 1.0 mA cm^−2^ and a fixed capacity of 1.0 mAh cm^−2^, maintaining a low overpotential of 50 mV (Supplementary Fig. S23). In contrast, symmetrical cell using BE exhibited significantly higher overpotential after prolonged cycling (post-1000 h) and shorter cycle lifespan. The sudden change observed in the initial overpotential were attributed to the formation of the SEI layer.

As derived from the Tafel plot in Supplementary Fig. S24, the exchange current density (I_0_) of the TFFE-based system reached to 0.38 mA cm^−2^, six times higher than that of BE (0.06 mA cm^−2^). Furthermore, the activation energy of Li^+^ transport through SEI (E_a,SEI_) and charge transfer (E_a,ct_) were calculated according to the Arrhenius law (Supplementary Fig. S25 and Table S2)^36,37^. By analyzing the temperature-dependent R_SEI_ profiles and charge transfer R_ct_ profiles, the E_a,SEI_ and E_a,ct_ for the ACSS-derived SEI were determined to be 38.62 and 39.66 kJ mol^−1^, significantly lower than those of 46.32 and 49.25 kJ mol^−1^ of the BE system (Fig. 4d and Supplementary Fig. S26). Concurrently, cyclic voltammetry (CV) scan of half-cell using TFFE also demonstrated sharp and symmetric redox peaks (Fig. 4e), indicative of enhanced Li^+^ transport kinetics and reduced overpotential^38^. This stark contrast underscores the role of ACSS-derived SEI in reducing Li⁺ deposition energy barriers, thereby enhancing interfacial charge transfer kinetics and Li plating/stripping reversibility^39,40^. Scanning electron microscopy (SEM) images of Li deposition further validated the effectiveness of the ACSS-derived SEI. When 3 mAh cm^−2^ of Li was deposited on Cu electrodes, uniformly distributed Li nuclei with compact and homogeneous morphology were observed in the TFFE system (Fig. 4f). In contrast, BE displayed mossy and irregular Li deposits under the same conditions (Fig. 4g).

To elucidate the origin of enhanced interfacial Li⁺ transport kinetics, surface X-ray photoelectron spectroscopy (XPS) was first employed to analyze the concrete composition of SEI. The Li *1s* and C*1s* spectra results demonstrated that TMSFS facilitated the formation of a multi-component and inorganic-rich SEI dominated by Li_2_S, LiF, and Li_2_O (Supplementary Fig. S27a,b). Notably, the relative contributions of solvent-derived components (Li_2_CO_3_/ROCO_2_Li and Li_x_PO_y_F_z_) were significantly reduced in the ACSS-derived SEI, consistent with the modified solvation structure that minimizes solvent participation in SEI formation (Supplementary Fig. S27c)^41^. Meanwhile, depth-profile XPS of the Li negative electrode after 10 and 50 cycles (Supplementary Fig. S28) shows clear signatures of TMSFS-derived species (Li_2_S and Li_x_SO_y_ fragments), demonstrating that the additive contributes directly to the formation of the inorganic-rich SEI. The presence of these species indicates that TMSFS persist participating in SEI formation during tens of cycles. Cryo-transmission electron microscopy (Cryo-TEM) provided more comprehensive insights into the microstructure of the ACSS-derived SEI layer. Specifically, a uniform interfacial architecture was observed with an overall thickness of approximately 42 nm, and the elements F, S, and O were homogeneously distributed across the surface of the Li metal (Supplementary Fig. S29). The corresponding high-resolution TEM images revealed distinct lattice spacings associated with the crystalline phases in various regions, where the lattice spacings corresponding to the Li_2_S (200), Li_2_O (200), and LiF (111) planes were measured as 0.29 nm, 0.27 nm, and 0.23 nm, respectively (Fig. 4h)^42^. Furthermore, the chemical composition and spatial distribution of SEI layer were evaluated by using ToF-SIMS. Inorganic components such as LiO^−^, LiS^−^, and LiF^−^ exhibited a uniform distribution across over the top surface of the Li metal, with their signal intensities showing a decline after sputtering (Fig. 4i, j), consistent with the results of cryo-TEM. Notably, the signal of SiC_3_H_9_^−^ was also detected on the Li metal surface, confirming the decomposition of TMSFS and its participation in SEI formation (Supplementary Fig. S30).

### Electrochemical performance of lithium metal full cell

**Fig. 5:**
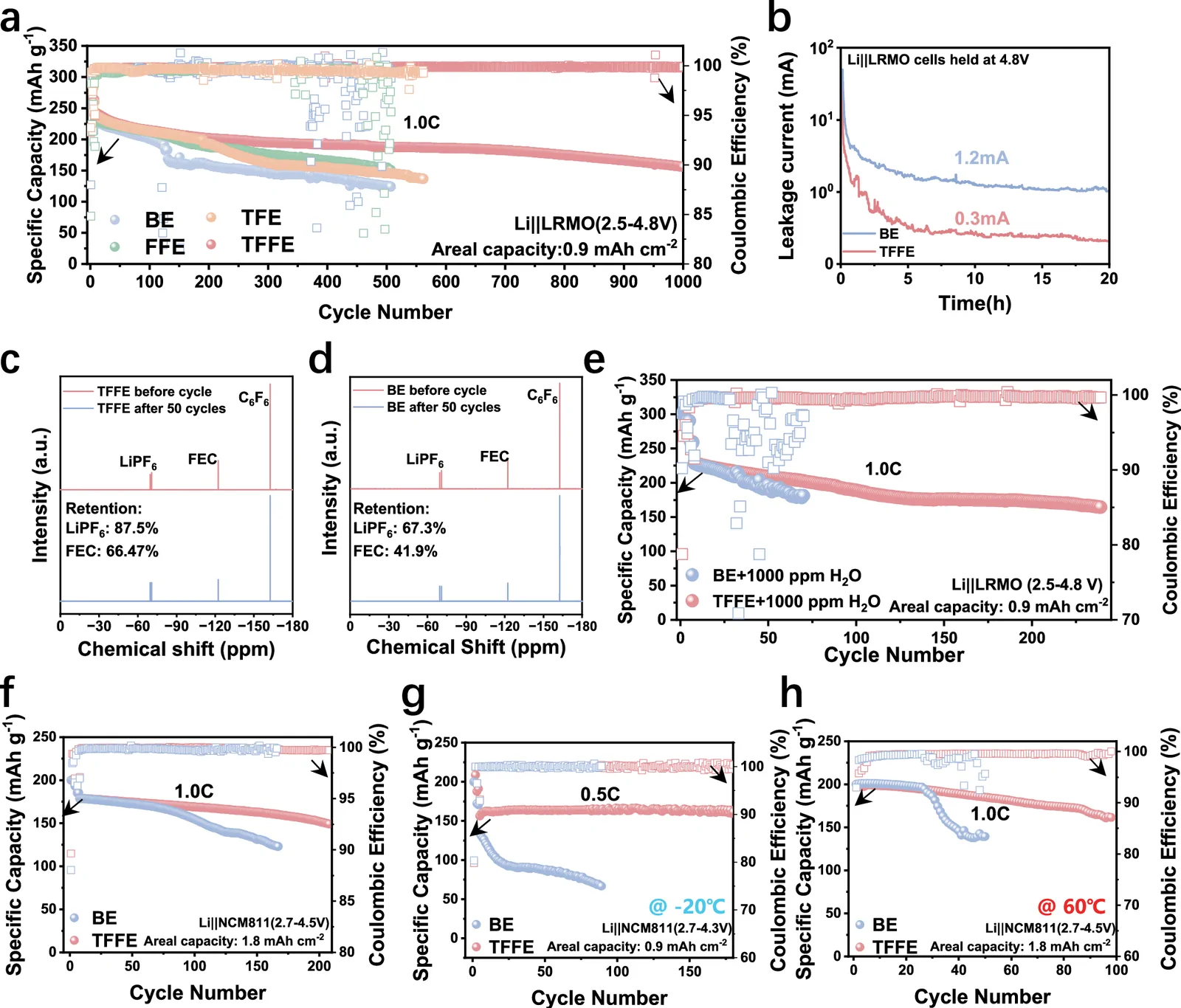
Electrochemical performance and operational stability of full cells. **a** Cycling stability and Coulombic efficiency of Li||LRMO full cells assembled with BE and TFFE electrolytes, tested from 2.5 to 4.8 V (1 C = 300 mA g^−^¹); **b** Leakage current test of full batteries with BE and TFFE electrolytes; ^19^F NMR spectra of (**c**) TFFE and (**d**) BE electrolytes after 50 cycles in Li||LRMO cells at 0.9 mA cm^−2^; **e** Cycling stability of Li||LRMO full cells (1 C = 300 mA g^-^¹) with BE and TFFE electrolytes contaminated with 1000 ppm H_2_O; Comparative cycling performance of Li||NCM811 full cells assembled with BE and TFFE electrolytes under (**f**) ambient temperature of 25 °C ± 0.5 °C, **g** low-temperature of −20 °C ± 0.5 °C; **h** high-temperature of 60 °C ± 0.5 °C, 1 C = 200 mA g^−^¹.

To evaluate the impact of ACSS on high-voltage LMB performance, Li||LRMO coin cell were assembled. Firstly, linear sweep voltammetry (LSV) shows a preferential oxidation peak indicating the prioritized decomposition of TMSFS in TFFE system (Supplementary Fig. S31), after which the oxidation effect of the formulated electrolyte was restrained for the reason of the as-formed robust EEIs, demonstrating enhanced high-voltage tolerance. As shown in Fig. 5a, the TFFE-based cell retained 70% capacity retention with an average CE of 99.87% after 850 cycles at 1 C and presents stable charge/discharge curves (Supplementary Fig. S32a), while BE-based cell decayed to below 50% capacity retention after 500 cycles (Supplementary Fig. S32b), FFE-based cell exhibited a capacity retention of below 70% after 400 cycles (Supplementary Fig. S32c), and the TFE-based cell maintained ~60% capacity retention after 500 cycles under identical cycling conditions (Supplementary Fig. S32d). Furthermore, full-cell test results with different additive contents indicated that the addition of 1 wt% exhibited the optimal performance (Supplementary Fig. S33), which was caused by the fierce side reactions caused by excessive addition and the corresponding thicker and less conductive interface reaction layer. Rate performance test showed that the TFFE-based Li||LRMO cell delivered a discharge capacity of 150 mAh g^−1^ at a high rate of 5 C (Supplementary Fig. S34), whereas BE-based exhibited a low capacity of 75 mAh g^−^^1^, illustrating the effectiveness of ACSS to enhance the battery reaction kinetics. Furthermore, floating tests with leakage current monitoring were conducted on Li||LRMO cell held at 4.8 V. After 10 cycles, TFFE-based cell demonstrated a polarization current of 0.27 mA cm^−2^ during a 20-hour voltage holding, significantly lower than that of BE-based cell (1.06 mA cm^−2^) (Fig. 5b). This four-fold reduction in polarization current underscored the critical role of ACSS in enhancing high-voltage stability^43^. Selected dQ/dV-voltage curves revealed that TFFE-based cell obtained a reduced polarization and capacity decay, preserving a well-defined dQ/dV profile, with the Mn reduction peak remaining at 2.85 V (Supplementary Fig. S35a), while LRMO materials undergone serious irreversible phase transitions and capacity decay in BE, showing a further peak shift to 2.78 V (Supplementary Fig. S35b), consistent with the results observed in EIS measurements (Supplementary Fig. S36). Additionally, TFFE-based Li||LRMO cell maintained 80% capacity retention after 260 cycles at 2.0 C (Supplementary Fig. S37), while BE-based counterparts failed within 100 cycles. Galvanostatic intermittent titration technique (GITT) analysis further elucidated the improved Li⁺ diffusion kinetics at LRMO positive electrodes. The calculated Li⁺ diffusion coefficient (D_Li+_) for TFFE-based cell was significantly higher than that of BE-based systems (Supplementary Fig. S38), aligning well with the enhanced rate capability and EIS results^44^.

To elucidate the role of ACSS in reducing the consumption of Li salt and solvent in the electrolyte, the residual component contents after cycling were analyzed using ^19^F NMR spectroscopy^45^. The results show that TFFE-based cell retains a higher concentration of LiPF_6_ (87.5%) and FEC (66.5%), with consumption ratio reducing by more than 60% and 40%, respectively, compared to BE-based counterparts (Fig. 5c, d). This suggests that the coordinated-TMSFS molecules well exert their EEI-forming functionality, minimizing electrolyte decomposition and consequently enhancing the battery cycle life. Inductively coupled plasma mass spectrometry (ICP-MS) was employed to measure the concentrations of transition metal (TM) elements (Mn, Co, Ni) released from LRMO after 50 cycles (Supplementary Fig. S39). The amounts of Mn, Co, and Ni deposit on the LMA in TFFE-based cell were 42%, 26%, and 50% of those in BE-based cell, respectively. This reduction indicated the effectiveness of ACSS on preserving the structural integrity of the positive electrode material and suppressing TM dissolution and shuttling.

Trace amounts of water present in the electrolyte will induce hydrolysis of Li salts, resulting in the generation of HF. This can lead to the dissolution of TMs at the positive electrode and degradation of the EEI layer, ultimately compromising the cycle life of batteries. The TMS functional groups containing Si–O structures are known for scavenging harmful HF in the electrolyte, thereby offering protection to electrode materials against acidic corrosion^46^. Accordingly, to further investigate the protective efficacy of TMSFS under aqueous contamination, the electrochemical performance was evaluated after introducing 1000 ppm H_2_O. As shown in the ^19^F NMR spectra, TFFE displays a stronger PF_6_^−^ peak at 75 ppm compared to BE (Supplementary Fig. S40)^47^. Peaks at 192 ppm and 85 ppm, corresponding to hydrolysis products of LiPF_6_ (PO_2_F_2_^−^ and HF, respectively)^48^ were significantly lower than those of BE, as evidenced by color change of electrolyte after standing for 12 days (Supplementary Fig. S41). Under 4.8 V and 1 C conditions, Li||LRMO full cell using TFFE with 1000 ppm H₂O retained a specific capacity of 170 mAh g^−^^1^ after 200 cycles, whereas the cell using BE with 1000 ppm H₂O degraded to below 100 mAh g^−1^ under same conditions, indicating that the TMSFS additive could effectively suppress HF generation and thereby reduce the damage to EEI (Fig. 5e).

For the purpose of validating the practical impact of ACSS on other high-voltage positive electrodes and on improving wide temperature range performance, Li | |NCM811 full cell were assembled and tested under different temperatures. Full cell using TFFE exhibited stable cycling performance under 25°C ± 0.5 °C, retaining 85% capacity after 200 cycles at 1 C (2.7–4.5 V), whereas BE counterparts degrade below 80% retention after only 120 cycles (Fig. 5f). The dQ/dV-voltage profiles showed that the TFFE-based system exhibited a less pronounced structural degradation trend compared to BE-based counterparts (Supplementary Fig. S42a, b). At a low temperature (−20 °C ± 0.5 °C), sluggish kinetics affected LMB performance due to severely restricted Li⁺ transport in both electrodes and electrolytes^49^. Meanwhile, TFFE-based full cell maintained a specific capacity of about 160 mAh g^−1^ after 200 cycles at 0.5 C (Fig. 5g), while BE-based cell suffered rapid capacity decay under 100 mAh g^−1^ within 30 cycles. At elevated temperatures (60 °C ± 0.5 °C), cell using TFFE retained about 165 mAh g^−1^ after 100 cycles at 1 C, whereas BE-based cell failed catastrophically after 25 cycles (Fig. 5h). The dQ/dV analysis showed that TFFE-based system exhibited attenuated structural degradation even at this high temperature. (Supplementary Figs. S42c, d). These findings emphasize the efficacy of the ACSS in significantly enhancing the cycling stability of Li||high-voltage positive electrode cell, even under harsh operational conditions.

### Mechanism of ACSS and performance of high specific energy Li metal pouch cells

**Fig. 6:**
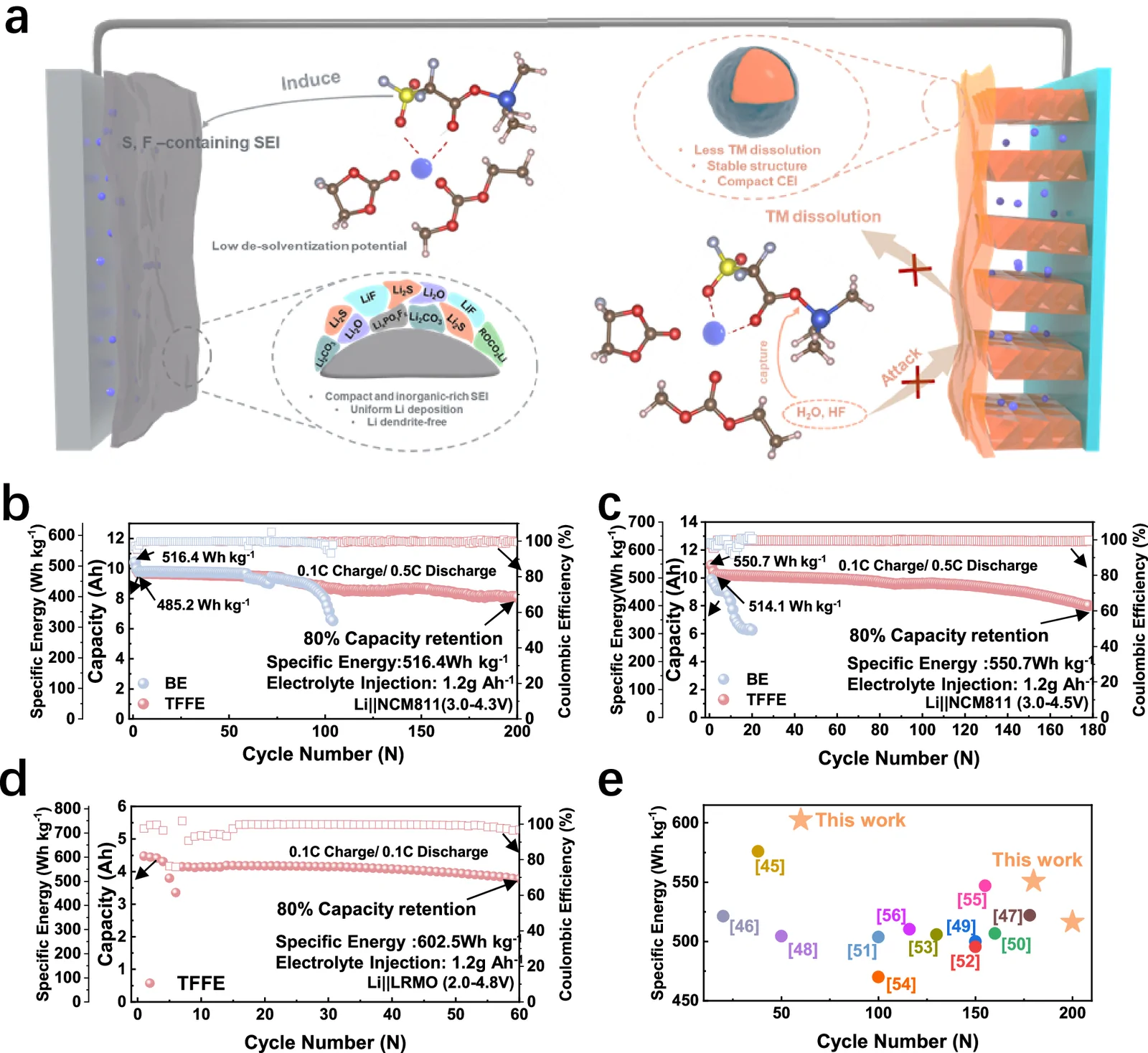
Practical pouch-cell validation of the ACSS strategy. **a** Schematic illustration of the working mechanism of ACSS in stabilizing lithium metal batteries, light blue: Li⁺; red: O; brown: C; pink: H; blue: Si; yellow: S; gray: F; Cycling performance comparison of Li||NCM811 pouch cells assembled with BE and TFFE electrolytes (**b**) from 3.0 to 4.3 V (**c**) from 3.0 to 4.5 V, specific energy was calculated based on a total mass of 76.2 g, using the actual median voltage of each cycle; **d** Cycling performance of Li||LRMO pouch cells assembled with TFFE electrolytes from 2.0 to 4.8 V, specific energy was calculated based on a total mass of 26.3 g, using the actual median voltage of each cycle; **e** specific energy and cycle life benchmarking of the LMB pouch cell in this work compared to recently reported advanced electrolyte systems (specific energy was calculated from the first-cycle discharge at 0.1 C), the source of the literature data shown in this figure can be found in Supplementary Table S6.

Based on the analysis of theoretical calculation and characterization tests, the working mechanism of ACSS in stabilizing LMBs is illustrated in Fig. 6a. The ACSS electrolyte concurrently enables positive electrode and negative electrode interfacial stabilization mechanisms. The TMSFS additive can not only form a conformal protective layer at the positive electrode, preserving the structural integrity of positive electrode particles during high-voltage cycling, but also facilitate the formation of a mechanically robust and highly ionically conductive inorganic-rich SEI layer, enabling homogeneous and reversible Li deposition at the negative electrode.

To evaluate the practical performance of the ACSS electrolyte, 10 Ah-class Li∥NCM811 pouch cells were assembled (Supplementary Fig. S43 and Table S4). All specific energy values were calculated based on the total cell mass, including packaging materials and tabs, with an electrolyte injection amount of 1.2 g Ah^−1^. And pouch cells were evaluated at different discharge rates, from which a moderate rate (<0.5 C) was identified as the most suitable testing condition for reliable performance assessment (Supplementary Fig. S44). The TFFE-based Li||NCM811 pouch cell achieved a high specific energy of 516.4 Wh kg^−1^ and stable cycling performance at 4.3 V for over 200 cycles with 80% capacity retention, while BE-based pouch cell failed after 90 cycles (Fig. 6b). Further increasing the cut-off voltage to 4.5 V, the TFFE-based Li|| NCM811 pouch cell made a significant increase in specific energy to 550.7 Wh kg^−1^ and surpasses 180 cycles with 80% capacity retention, while BE-based counterpart failed within 20 cycles (Fig. 6c and Supplementary Fig. S45). As shown in Supplementary Fig. S46, TFFE-based pouch cells exhibit consistently high CE (>98%) throughout cycling, indicating interface-stable failure dominated by electrolyte depletion, whereas the control cells show simultaneous CE and capacity collapse, characteristic of Li negative electrode degradation, leading to premature failure. After disassembling the pouch cell, SEM image of the Li metal negative electrode in TFFE-based pouch cell exhibited compact and homogeneous morphology without visible dendritic features (Supplementary Fig. S47a), while the Li metal negative electrode in BE-based cell displayed mossy and irregular Li deposits under identical cycling conditions (Supplementary Fig. S47b). Meanwhile, SEM image of positive electrodes revealed that positive electrode particles in TFFE-based cell maintained structural integrity with minimal damage (Supplementary Fig. S48a), whereas BE counterparts suffered from severe particle cracking and fragmentation (Supplementary Fig. S48b).

In order to further improve the specific energy of the battery, a high reversible 602.5 Wh kg^−1^ pouch cell was assembled by matching Li metal with high mass loading of LRMO positive electrode (Supplementary Table S5). The Li||LRMO pouch cell with ACSS electrolyte demonstrated a stable cycling performance from 2.0 to 4.8 V exceeding 60 cycles with 80% capacity retention (Fig. 6d and Supplementary Fig. S49). To demonstrate the enhanced performance, a comparison was made with some recent relevant reports about high specific energy pouch cell (Fig. 6e and Supplementary Table S5). Compared to recently reported high specific energy LMB pouch cell (≥450 Wh kg^−1^), the TMSFS-formulated electrolyte demonstrated enhanced cycling stability, achieving high specific energy and long-term stable cycling in LMB pouch cell. The results revealed favorable performance in both specific energy and cycle life, collectively highlighting the promising potential of ACSS electrolyte implemented through TMSFS additive for practical high-energy-density pouch cell applications^50–61^.

## Discussion

In this work, we report an ACSS electrolyte achieved by strongly coordinating TMSFS additive for stable cycling of high-voltage practical LMBs. Even with a low addition of 1 wt%, the ACSS electrolyte features the effective reconfiguration of the Li⁺ solvation structure with TMSFS additives displacing the solvent molecules and anions from the inner solvation sheath. This not only mitigates the intense consumption of the baseline components in the electrolyte but also reinforces the Li metal compatibility and high-voltage stability (≥4.8 V vs. Li/Li⁺) through the formation of dense and uniform inorganic-rich EEIs (derived from TMSFS additive decomposition). Our designed ACSS electrolyte enables long cyclability for the high-energy-density LMBs even under harsh testing conditions. The practical 550.7 Wh kg⁻¹ Li||NCM811 pouch cell maintained 80.0% capacity retention over 180 cycles and the Li||LRMO pouch cell achieved a specific energy of 602.5 Wh kg⁻¹. This work demonstrates that the ACSS design principles open up encouraging avenue towards advancing electrolyte for high-voltage practical LMB systems.

## Methods

### Materials

Lithium hexafluorophosphate (LiPF_6_), EC, fluoride ethylene carbonate (FEC), and ethylmethyl carbonate (EMC) were purchased from DoDoChem. Methyl 2,2-difluoro-2-(fluorosulfonyl) acetate (FS), trimethylsilyl acetate (TMS) and trimethylsilyl-2,2-difluoro-2-(fluorosulfonyl) acetate (TMSFS) were provided by Kasei Industry Development Co. (TCI Shanghai), all with a purity of 99.99%. The LiNi_0.8_Co_0.1_Mn_0.1_O_2_ (NCM811) (purity 99.9%) and lithium-rich manganese-based layered oxides (LRMO, Li_1.2_Mn_0.54_Co_0.13_Ni_0.13_O_2_) (purity 99.9%) positive electrode material, Super P and polyvinylidene fluoride (PVDF, Mw = 1,100,000, average particle size ≈ 100 μm) were obtained from Shenzhen Kejing Zhida Technology Co., Ltd without any treatment. The Copper (Cu, 99.9%, 10μm), aluminum (Al, 99.9%) current collectors poly (vinylidene fluoride), positive electrode materials, and Li foils (99.95%, for coin cells (CR2025), Li foil discs (thickness 0.45 mm, diameter 15.6 mm) were punched; for pouch cells, Li foils of 50 or 100 μm thickness and 100 mm width were cut to size. The Li electrodes were stored under inert gas and dry air for ≤1 month) were supplied by Canrd New Energy Technology Co., Ltd.

### Electrolytes preparation

First, the mixed solvent with volume ratio of 1:4 was prepared by FEC and EMC was ready. Then BE electrolytes were prepared by dissolving 1 M LiPF_6_ in two mixed solvents, respectively. The FFE, TFE, and TFFE were modified by adding 1.0 wt% of FS, TMS and TMSFS, respectively, into BE.

### Electrochemical measurements

The galvanostatic intermittent titration technique (GITT) measurements were conducted at 0.05 C with a 0.33-h charge/discharge and a 1-h interval period in each with a data acquisition rate of 1 point per 10 s. The galvanostatic measurements and GITT were evaluated at 25 °C ± 0.5 °C by Neware battery testing system (CT-4008). Electrochemical impedance spectroscopy (EIS) experiments were performed at frequencies between 100 kHz and 0.1 Hz, using a potentiostatic mode with an amplitude of 10 mV (10 points per decade) at 25 °C ± 0.5 °C. Before each measurement, the cells were rested at open‑circuit voltage for 2 h to reach a quasi‑stationary state. The fitting quality was evaluated by *χ*², and all values were below 1 × 10⁻³, indicating a reliable fitting. Linear sweep voltage (LSV) curve test at a scan rate of 0.2 mV s^−1^ between 3 and 6 V of Li||LRMO batteries were done on the CHI 760E electrochemical workstation. And the ionic conductivity test (conducted based on SS||SS coin cell) was calculated based on the following Eq. 1:

1 $${{\rm{\sigma }}}=\frac{{{\rm{L}}}}{{{\rm{R}}}\cdot {{\rm{S}}}}$$

where *L* represents the thickness of the separator, *R* is obtained from the EIS measurements of the SS/electrolyte/SS blocking cell, and *S* is the area of SS electrode. All the tests were conducted at 25 °C ± 0.5 °C.

Assembly of coin cell and electrochemical test: All CR2025-type coin cells (Diameter: 20 mm; thickness: 2.5 mm) were assembled in an Ar-filled glove box (O_2_ and H₂O < 0.1 ppm) with 40 μL electrolytes. Positive electrodes were prepared by casting N-methylpyrrolidone (NMP) slurries containing active material (LRMO or NCM811), Super P conductive carbon, and PVDF binder (8:1:1 mass ratio) onto aluminum foil current collectors (single-side coated), and punched into discs (12 mm diameter) with a manual disc cutter (MSK-T10). Full cells (Li||LRMO or Li||NCM811) were cycled between 2.5–4.8 V and 2.7–4.5 V, respectively. Almost all coin cell testings were conducted at 25 °C ± 0.5 °C using a LAND CT3001A battery test system except for two formation cycles at 25 ± 0.5 °C before −20 ± 0.5 °C and 60 ± 0.5 °C tests using a climatic chamber (BINDER KMF‑115) to maintain the desired temperatures. Mass loading of positive electrodes: LRMO-3.0 mg cm^−2^; NCM811-9.0 mg cm^−2^.

Assembly of the Li metal pouch cell: Lithium metal pouch cells were assembled in a humidity-controlled environment (dew point < −50 °C at 25 °C) using a semi-automatic laminator (MSK-111A-L, Shenzhen Kejing Star Technology Co., Ltd) equipped with Z-stacking functionality. Prior to long-term cycling, electrolyte (1.2 g Ah⁻¹) was injected in the dry room. The pouch was vacuumed for 10 s, heat‑sealed under vacuum, and then a second sealing was performed after formation cycles. The formation cycles were conducted at 0.1 C to stabilize the SEI and to release any gas generated inside the cell. The formation protocol consisted of sequential constant‑current charging steps at 0.02 C (30 min), 0.05 C (30 min) and 0.1 C (up to the cut‑off voltage), followed by a constant‑current discharge at 0.1 C (to the cut‑off voltage). During cycling, a constant external pressure of 0.25 MPa was applied by a mechanical fixture with a load cell. Electrochemical testing was performed on a LAND multichannel battery test system (Wuhan LAND Electronics) under the following conditions: Li||NCM811 cell: 3.0–4.5 V, areal capacity: 4.2 mAh cm^−2^, voltage window Li||LRMO cell: 2.5–4.8 V, areal capacity: 5.4 mAh cm^−2^. All tests were maintained at 25 °C with an applied stack pressure of 0.25 MPa during charge/discharge processes to ensure uniform electrode interfacial contact.

Materials characterization: SEM images were captured on a Hitachi SU8020 instrument. For postmortem analyses, including SEM and XPS measurements, cycled coin cells were dissembled in the Ar-filled glovebox to collect electrodes. The compositions and structure of SEI were researched by etching XPS (Thermo Scientific K-Alpha), all binding energies were referenced to the C 1 s peak of adventitious carbon at 284.8 eV and background subtraction was performed using a Shirley type background for all core‑level spectra. Depth profiling and chemical analysis data of the sample were collected on a TOF-SIMS instrument (IONTOF GmbH, Germany 2010, the Analysis & Testing Center, Beihang University). The data were recorded in ultrahigh vacuum at a pressure of 10^−9^ Torr in a negative model. Primary ion beam: Bi^3+^, 30 kev, 45 deg, MVA 100 nm, scanning area: 100×100${{\rm{\mu }}}{{\rm{m}}}$, secondary ion polarity and mass range: neg, 2–1850 amu; sputtered ion beam: Cs^+^, 1 kev, 10 s, 45 deg, etching area: 400 × 400${{\rm{\mu }}}{{\rm{m}}}$. sputtering speed = 0.8 nm/s for SiO_2_. Scanning electron microscope (SEM, Hitachi SU8020), transmission electron microscopy (TEM, JEOL JEM-F200) and X-ray diffraction (XRD, Bruker X-ray diffractometer (D8 Advance) with Cu Kα radiation) were used to observe the surface morphologies, PEI thickness and lattice structure of cycled LRMO particles, respectively. The TMs contents on Li negative electrodes were characterized by Inductively Coupled Plasma Mass Spectrometry (ICPMS) (Agilent 720ES(OES)). Cryo-TEM: Bare Cu TEM grids were used as a substrate for Li deposition in a Li||Cu half-cell. The plated Li amount was 0.5 mAh cm^−2^ at a current density of 0.5 mA cm^−2^. All the cryo-TEM images were collected at −170 °C. All cells were disassembled in an Ar‑filled glovebox (H_2_O, O_2_ < 0.1 ppm). electrodes were rinsed thrice with anhydrous DMC, dried under vacuum at 25 ± 1 °C for 12 h, then transferred to test via a vacuum‑sealed vessel; SEM and TEM samples were transported in an Ar‑filled glovebag. ^1^H and ^7^Li NMR spectra of BE, TFFE, BE + 1000 ppm H_2_O, and TFFE + 1000 ppm H_2_O electrolytes were performed using the NMR tube with an external coaxial insert, DMSO-d6 was put into the coaxial insert, and the electrolytes were out into the outer NMR tube and sealed. The tubes were removed from the glove box, and analyzed by NMR spectroscopy. ^19^F NMR spectra were acquired to test the components of the electrolyte after the cycle. For each coin cell, 20 μL of the corresponding electrolyte was used. After 50 cycles, the corresponding coin cells were disassembled and rinsed with deuterated dimethyl sulfoxide-d6 (DMSO-d6, 2.5 mL) to extract residual electrolyte. The collected solution was doped with 4.0 μL of C₆F₆ as an internal standard prior to NMR measurements. The Diffusion-ordered spectroscopy (DOSY) was performed using the NMR tube with an external coaxial insert. For the experiment, DMSO-d6 was put into the coaxial insert, and the mixture of electrolytes and toluene (internal standard) was put into the outer NMR tube. According to the Stokes-Einstein equation, the diffusion coefficient of toluene can be described as Eq. 2.

2 $${{{\rm{D}}}}_{1{{\rm{T}}}}=\frac{{{\rm{k}}}{{{\rm{T}}}}_{1}}{6{{\rm{\pi }}}{{{\rm{\eta }}}}_{1}{{{\rm{r}}}}_{1}}$$

Owing to its low polarity and low donor number (DN), toluene hardly participates in the coordination of Li ions. Consequently, its hydrodynamic radius (*r*) is expected to remain unchanged upon the addition of lithium salt. Therefore, Eq. 3 holds.

3 $$\frac{{{{\rm{D}}}}_{1{{\rm{T}}}}}{{{{\rm{D}}}}_{2{{\rm{T}}}}}=\frac{{{\rm{k}}}{{{\rm{T}}}}_{1}}{6{{\rm{\pi }}}{{{\rm{\eta }}}}_{1}{{{\rm{r}}}}_{1}}/\frac{{{\rm{k}}}{{{\rm{T}}}}_{2}}{6{{\rm{\pi }}}{{{\rm{\eta }}}}_{2}{{{\rm{r}}}}_{2}}$$

Where *D*_1T_ and *D*_2T_ are respectively the diffusion coefficient of toluene before and after the addition of LiPF_6_, *T*_1_ and *T*_2_ are respectively the temperature of the FEC: EMC = 1:4 solution before and after the addition of LiPF_6_, and the temperature was carefully controlled to remain constant during all measurements, *η*_1_ and *η*_2_ are the respective viscosity, *k* is the Boltzmann constant, and *r*_1_ and *r*_2_ represents the respective hydrodynamic radius.

The solvated solvent is supposed to present a similar diffusion coefficient as Li^+^, as represented in Eq. 4. Based on approximately the same hydrodynamic diameter (D_1solvent)_ of the non-coordinating solvent before and after the LiPF_6_ addition, we estimate the non-coordinating free solvent’s diffusion coefficient (D_free slovent_) in the salted electrolyte as follows (Eq. (5)). The diffusion coefficient of solvent measured in the salted electrolyte D_2solvent_ represents the average of the diffusion coefficients of coordinated solvent (D_Co-solvent_) and free solvent (D_free solvent_), as described in Eq. 6, where α denotes the coordination ratio of solvent.

4 $${{{\rm{D}}}}_{{{\rm{Li}}}}={{{\rm{D}}}}_{{{\rm{Co}}}\,{{\rm{slvent}}}}$$

5 $${{{\rm{D}}}}_{{{\rm{free\; solvent}}}}=\left(\frac{\frac{{{\rm{k}}}{{{\rm{T}}}}_{2}}{6{{\rm{\pi }}}{{{\rm{\eta }}}}_{2}{{{\rm{r}}}}_{2}}}{\frac{{{\rm{k}}}{{{\rm{T}}}}_{1}}{6{{\rm{\pi }}}{{{\rm{\eta }}}}_{1}{{{\rm{r}}}}_{1}}}\right){{{\rm{D}}}}_{1{{\rm{solvent}}}}=\frac{{{{\rm{T}}}}_{2}{{{\rm{\eta }}}}_{1}}{{{{\rm{T}}}}_{1}{{{\rm{\eta }}}}_{2}}{{{\rm{D}}}}_{1{{\rm{solvent}}}}$$

6 $${{{\rm{D}}}}_{2{{\rm{solvent}}}}={{{\rm{D}}}}_{{{\rm{Co}}}{{\rm{solvent}}}}{{\rm{\alpha }}}+{{{\rm{D}}}}_{{{\rm{free}}}\; {{\rm{solvent}}}}(1-{{\rm{\alpha }}})$$

Using Eqs. 2–6, the coordination percentage (α) of solvents can be calculated.

Theoretical calculation: DFT Calculations: All quantum chemistry calculations were performed using the ORCA 6.0 program package^62^. The geometries of all individual molecules, including EMC, FEC, FS, TMS, TMSFS, and PF_6_^−^, were fully optimized. The calculations were performed using the B3LYP functional, augmented with Grimme’s D3(BJ) dispersion correction, and the ma-def2-TZVP basis set^63^. Frequency analyses were subsequently performed at the same level of theory to verify that all optimized structures correspond to true local minima on the potential energy surface, as confirmed by the absence of imaginary frequencies. The resulting optimized structures were used as the basis for the subsequent MDs force field parameterization. The energies of HOMO and LUMO were extracted from these calculations to evaluate the electrochemical stability of the molecules. In addition, oxidation potentials of EMC, FEC, TMSFS and PF_6_^−^ were obtained from ΔSCF calculations between the neutral and one-electron oxidized species, and spin-density analysis was used to identify the preferred oxidation sites. Furthermore, the binding energy (E_b_) between a Li⁺ and the solvent or additive molecules was calculated to assess their interaction strength. The binding energy was determined using the following equation:

$${{{\rm{E}}}}_{{{\rm{b}}}}={{{\rm{E}}}}_{{{{\rm{Li}}}}^{+}-{{\rm{molecule}}}}-({{{\rm{E}}}}_{{{\rm{molecule}}}}+{{{\rm{E}}}}_{{{{\rm{Li}}}}^{+}})$$

where ${{{\rm{E}}}}_{{{{\rm{L}}}{{\rm{i}}}}^{+}-{{\rm{molecule}}}}$, ${{{\rm{E}}}}_{{{\rm{molecule}}}}$ and ${{{\rm{E}}}}_{{{{\rm{Li}}}}^{+}}$ represent the energies of the optimized Li⁺-molecule complex, the isolated optimized molecule, and the Li⁺ ion, respectively. Energy decomposition analysis combined with natural orbitals for chemical valence (EDA–NOCV) was additionally performed for selected Li^+^–additive complexes to analyze the nature of Li^+^–additive bonding. The ESP on the van der Waals surface of the optimized molecules was analyzed using the Multiwfn 3.8 program^64^. The ESP was evaluated based on the highly effective algorithm proposed^65^, providing insights into the reactive sites of the molecules; MD Simulations: Classical MD simulations were conducted using the Large-scale Atomic/Molecular Massively Parallel Simulator (LAMMPS)^66^ to investigate the structure and dynamics of the Li⁺ solvation sheath. All interactions were described using the OPLS-AA (Optimized Potentials for Liquid Simulations - All Atom) force field. The partial atomic charges for all molecules were obtained from RESP fitting to the DFT electrostatic potential and uniformly scaled following the electronic continuum correction (ECC) scheme^67^, as also adopted in prior OPLS-AA MD studies of LiPF_6_/carbonate electrolytes^68^. The simulations were performed in a cubic simulation box with an edge length of 60.46 Å. For the TFFE system, the simulation box contained 133 LiPF_6_ molecules, 982 EMC molecules, 348 FEC molecules, and 13 TMSFS molecules, corresponding to 1 M LiPF_6_ in EMC: FEC = 4:1 (by volume) with 1 wt% TMSFS. The other electrolyte systems (BE, FFE, and TFE) were constructed analogously at the same salt concentration and solvent ratio, with or without the corresponding additive. A blank control system without any additive was also simulated. With the force field parameters established, the simulation protocol commenced with an energy minimization to relax the system. This was followed by a multi-stage equilibration process: first, a 5 ps run in the NVE ensemble; then, a gradual heating to 300 K over 50 ps and a subsequent 100 ps stabilization period in the NVT ensemble; and finally, a 100 ps density equilibration in the NPT ensemble at 300 K and 1 atm. The temperature and pressure during the respective NVT and NPT stages were regulated by a Nose -Hoover thermostat and barostat^69^. Following the complete equilibration, a 2 ns production simulation was carried out under the same NPT conditions. The coordination number (CN) of Li⁺ ions was calculated to quantify the solvation environment during the production simulation. The coordination number was determined using the following equation:

$${{\rm{C}}}{{\rm{N}}}=4{{\rm{\pi }}}{{\rm{\rho }}}\int _{0}^{{{{\rm{r}}}}_{\min }}{{\rm{g}}}({{\rm{r}}}){{{\rm{r}}}}^{2}{{\rm{dr}}}$$

where ρ is the number density of the coordinating atoms, g(r) is the radial distribution function between the central ion and the coordinating atoms, and ${r}_{\min }$ is the position of the first minimum in the radial distribution function curve.

## Supplementary information

Supplementary Information

Description of Additional Supplementary Files

Supplementary Data 1

Supplementary Data 2

Transparent Peer Review file

## Source data

Source Data

## Data Availability

Source data are provided are provided in the Source Data file. Source data are provided with this paper.
