# Supplementary material for: Additive strongly-coordinated solvation structure towards high-voltage 600 Wh/kg-class lithium metal pouch cell
Source: Nat Commun. 2026 Aug 24;17:10119. doi: 10.1038/s41467-026-77095-x (PMC13597530; doi:10.1038/s41467-026-77095-x)
Supplement: Supplementary file 1 [file 41467_2026_77095_MOESM1_ESM.pdf]

Supplementary Materials for

Additive Strongly-Coordinated Solvation Structure towards High-Voltage 600 Wh/kg-class Lithium Metal pouch cells

Zhaoxia Zhang[1,2]‡, Qian Chen[1,2]‡*, Bixuan Li[1,2,3]‡, QingWei Zhai[4], Xiaokang Gu[1,2], Chundi Wei[1,2], Moxuan Wang[1,2], Yuying Jiao[1,2], Zhou Zhou[1,2], Qiannan Zhang[1,2], Andrew Xiang[1,2], Chunqiao Jin[1,2], Peng Kang[1,2,3], Pengbo Zhai[1,2]*, Kai Liu[5*] and Yongji Gong[1,2,6,7,8]*

1 Tianmushan Laboratory, Hangzhou 310023, China.
2 School of Materials Science and Engineering, Beihang University, Beijing 100191, China.
3 State Key Laboratory of Artificial Intelligence for Materials Science, Beihang University, Beijing 100029, China.
4 Jinan Zhongruitai New Material Technology Co., LTD, Jinan 250300, China.
5 Department of Chemical Engineering, Tsinghua University, Beijing, China.
6 Center for Micro-Nano Innovation, Beihang University, Beijing 100029, China.
7 Key Laboratory of Intelligent Sensing Materials and Chip Integration Technology of Zhejiang Province, Hangzhou, 310051, China.
8 Analytical&Testing Center, Beihang University, Beijing 100029, China.
Correspondence and requests for materials should be addressed to Y.G., K.L., P.Z., and Q.C. (email: yongjigong@buaa.edu.cn; liukai2019@tsinghua.edu.cn; bht0022@tmslab.cn; bhtb026@tmslab.cn)

‡These authors contributed equally to this work

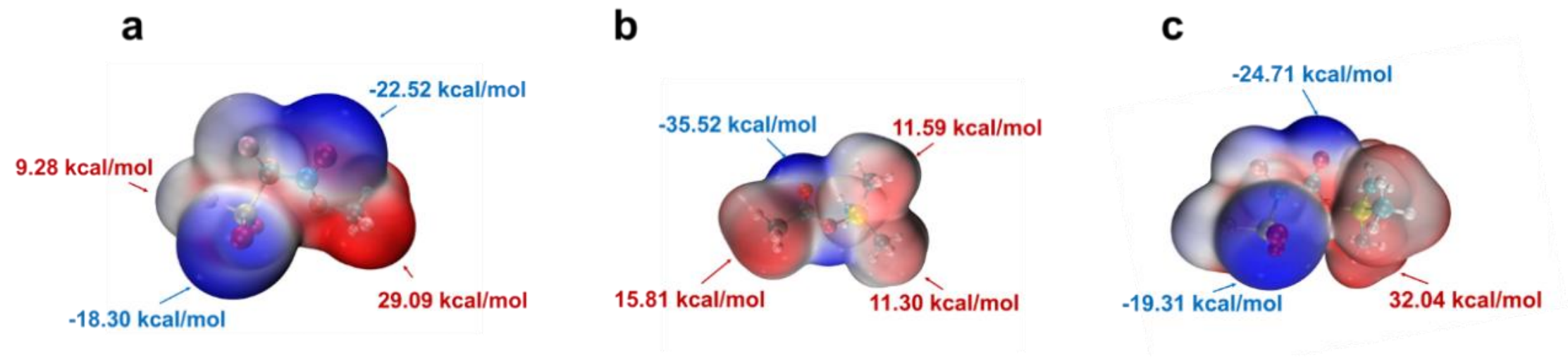

Supplementary Figure 1. The ESP maps roles of (a) FS, (b) TMS and (c) TMSFS molecules, red: O; green: C; gray: H; yellow (connected to CH_3): Si; yellow (connected to F): S; pink: F.

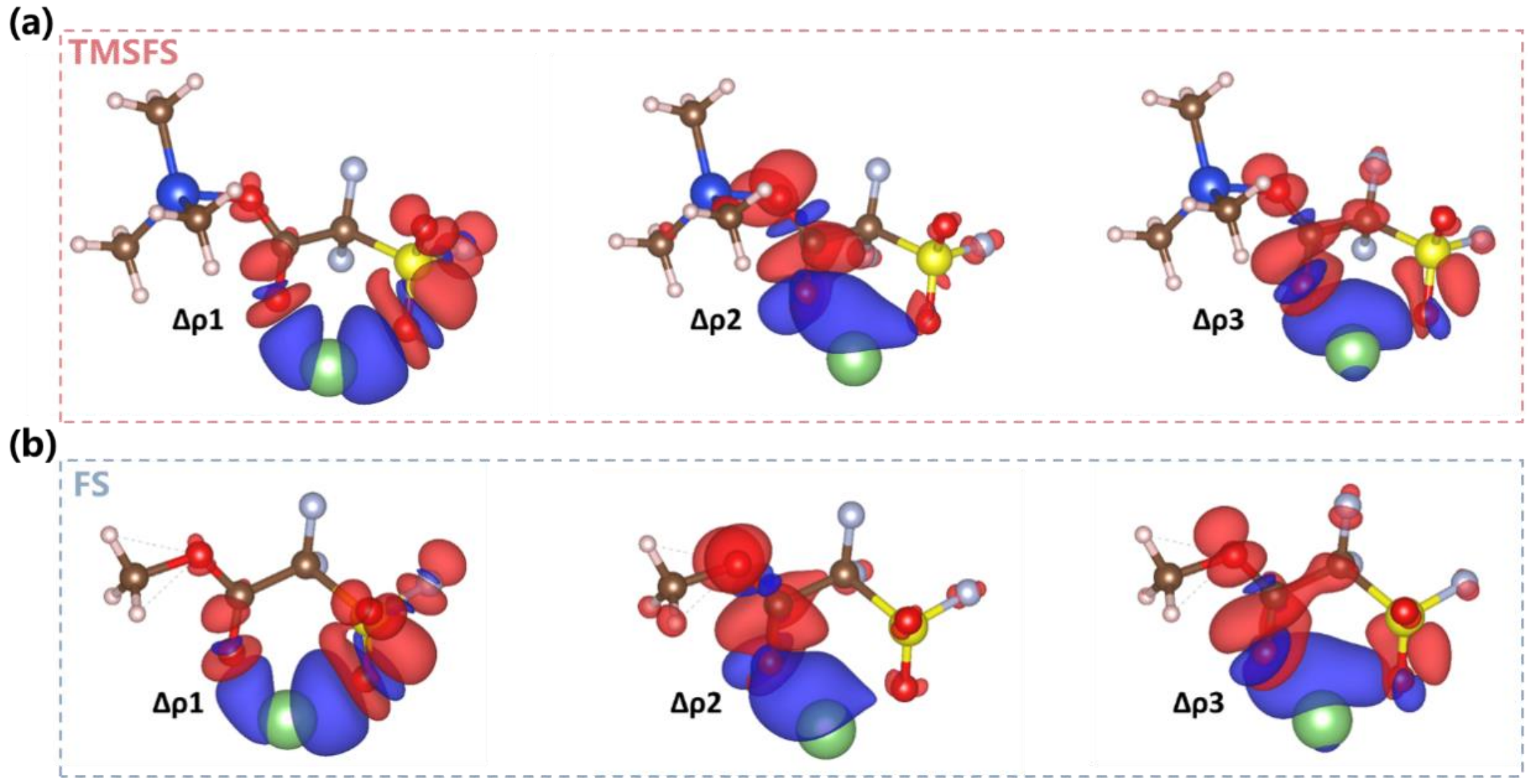

Supplementary Figure 2. EDA–NOCV (Energy Decomposition Analysis combined with Natural Orbitals for Chemical Valence) pattern comparisons of (a)TMSFS and (b)FS, Green: Li^+; red: O; brown: C; pink: H; blue: Si; yellow: S; gray: F.

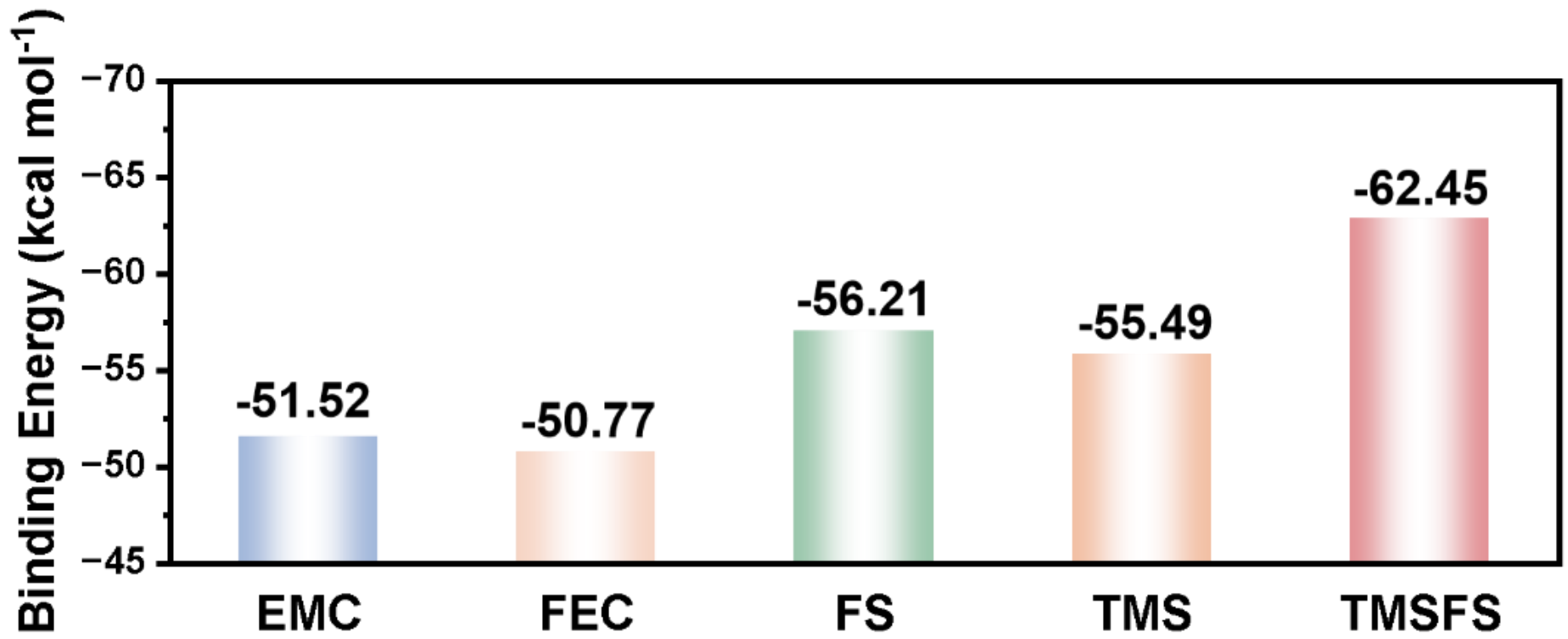

Supplementary Figure 3. Comparison of binding energies among solvents molecules (EMC and FEC) and additive molecules (FS, TMS and TMSFS).

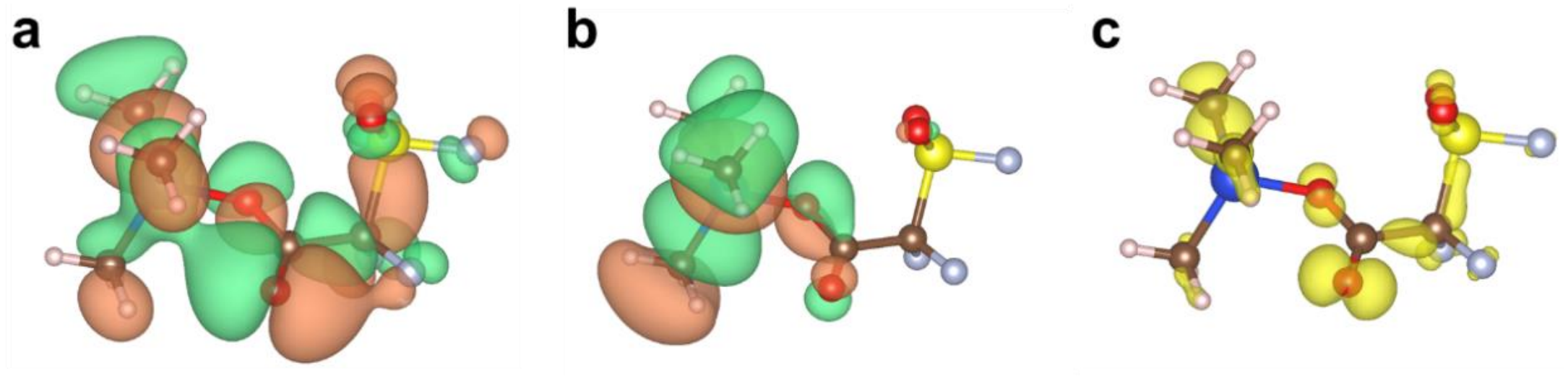

Supplementary Figure 4. (a) HOMO of neutral TMSFS; (b) SOMO of $TMSFS^+$ and (c) spin-density map of $TMSFS^+$, Purple: Li^+; red: O; brown: C; gray: H; blue: Si; yellow: S; green: F.

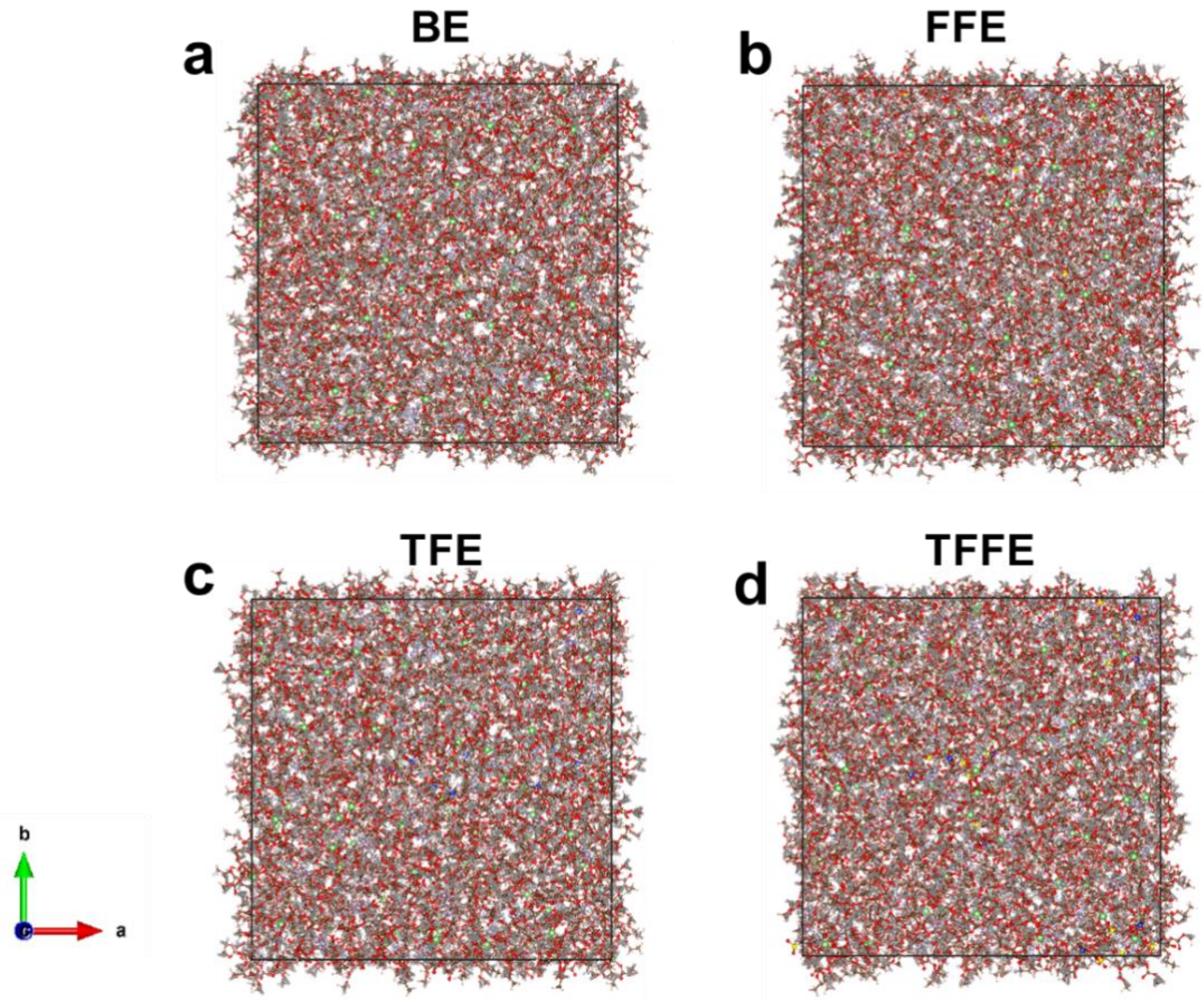

Supplementary Figure 5. Classical molecular dynamics (MD) simulations and schematic representations of solvation structures of (a) BE, (b) FFE, (c) TFE and (d) TFFE, Green: Li^+; red: O; brown: C; pink: H; blue: Si; yellow: S; gray: F.

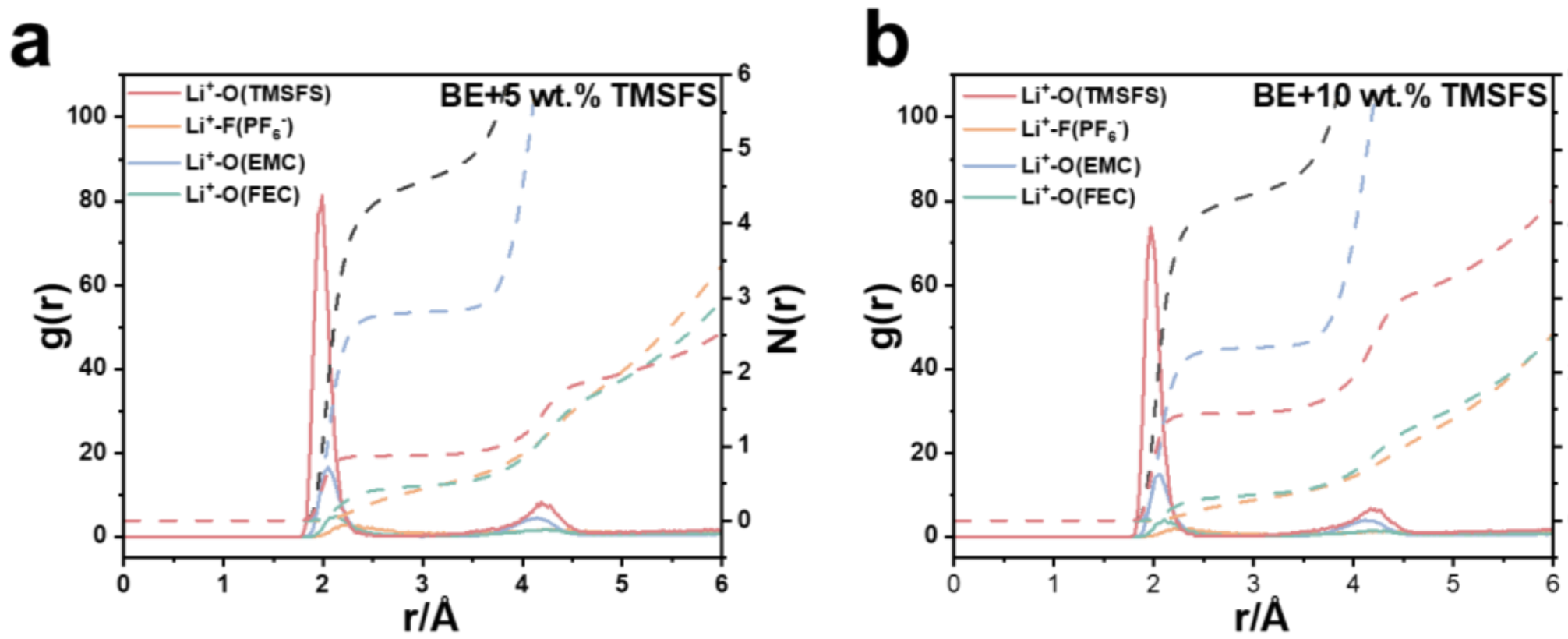

Supplementary Figure 6. RDFs of key species coordinating with Li^+ in (a) BE+5 wt.% TMSFS, and (b) BE+10 wt.% TMSFS, solid line represents g(r), dashed line represents N(r).

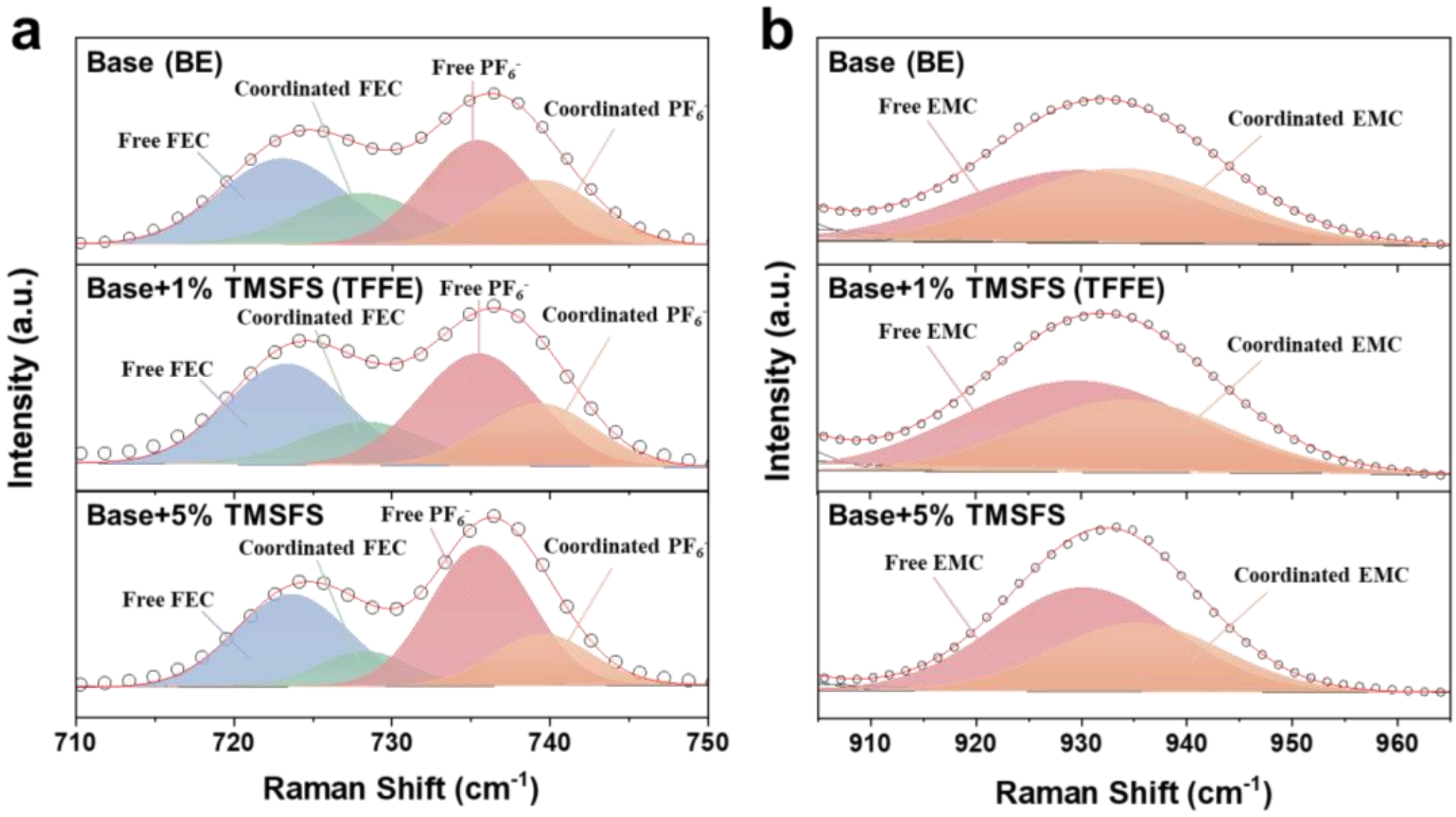

Supplementary Figure 7. Raman spectra of BE and BE+1 wt. %, 5 wt.% from (a)710 to 750 cm^{-1} and (b)905 to 965 cm^{-1}.

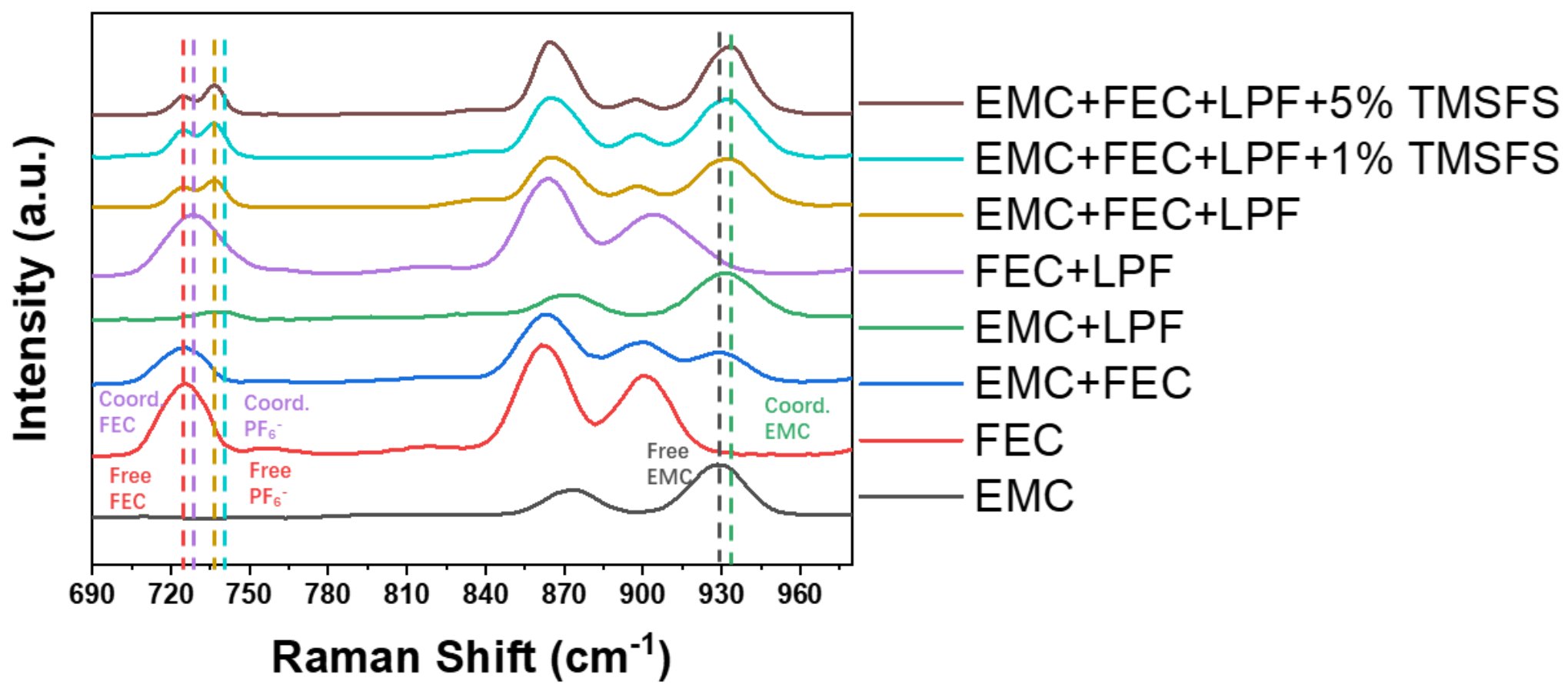

Supplementary Figure 8. Raman spectra analysis of Li^+ solvation structures in electrolytes with different contents from 690 to 970 cm^{-1}.

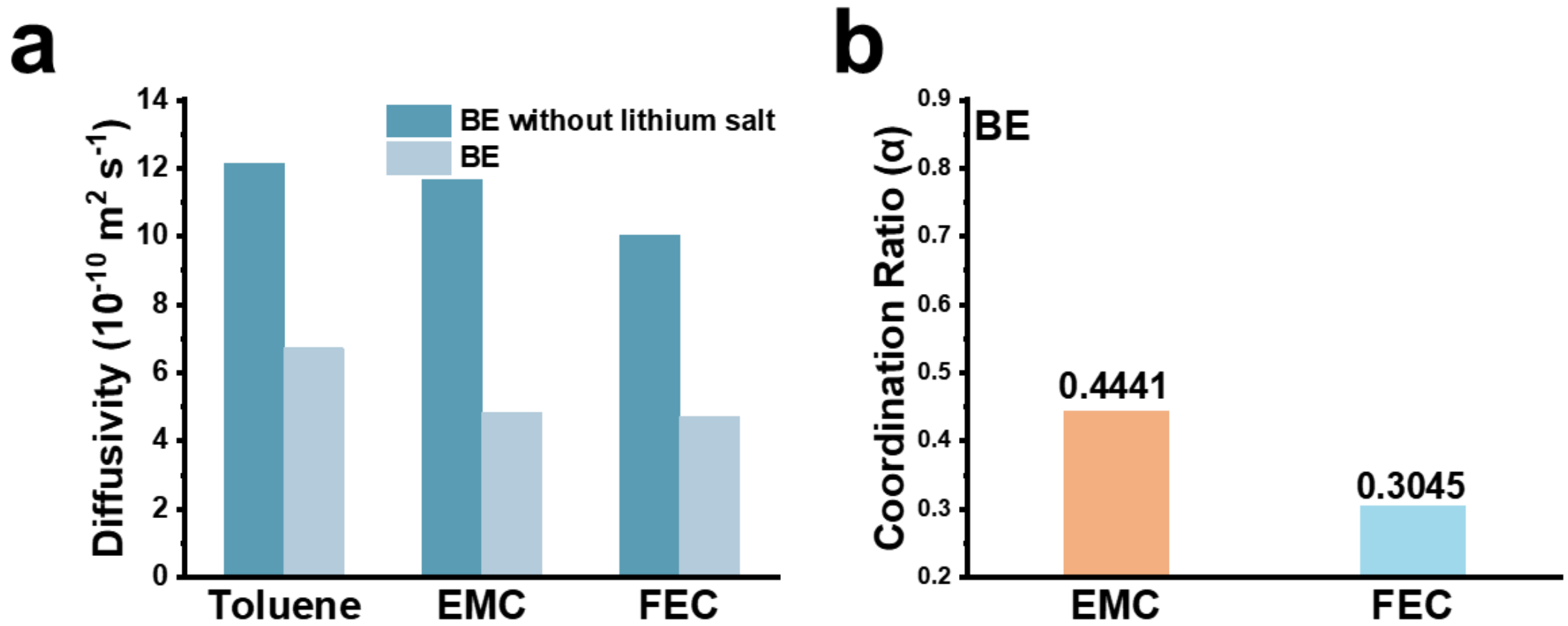

Supplementary Figure 9. Diffusion-ordered spectroscopy NMR (DOSY-NMR) analysis. The diffusivities of multiple components (toluene, EMC, FEC, TMSFS,) in (a)BE and BE without LiPF6 and (b)Corresponding coordination ratios calculated through DOSY of BE. Toluene was used as an internal reference.

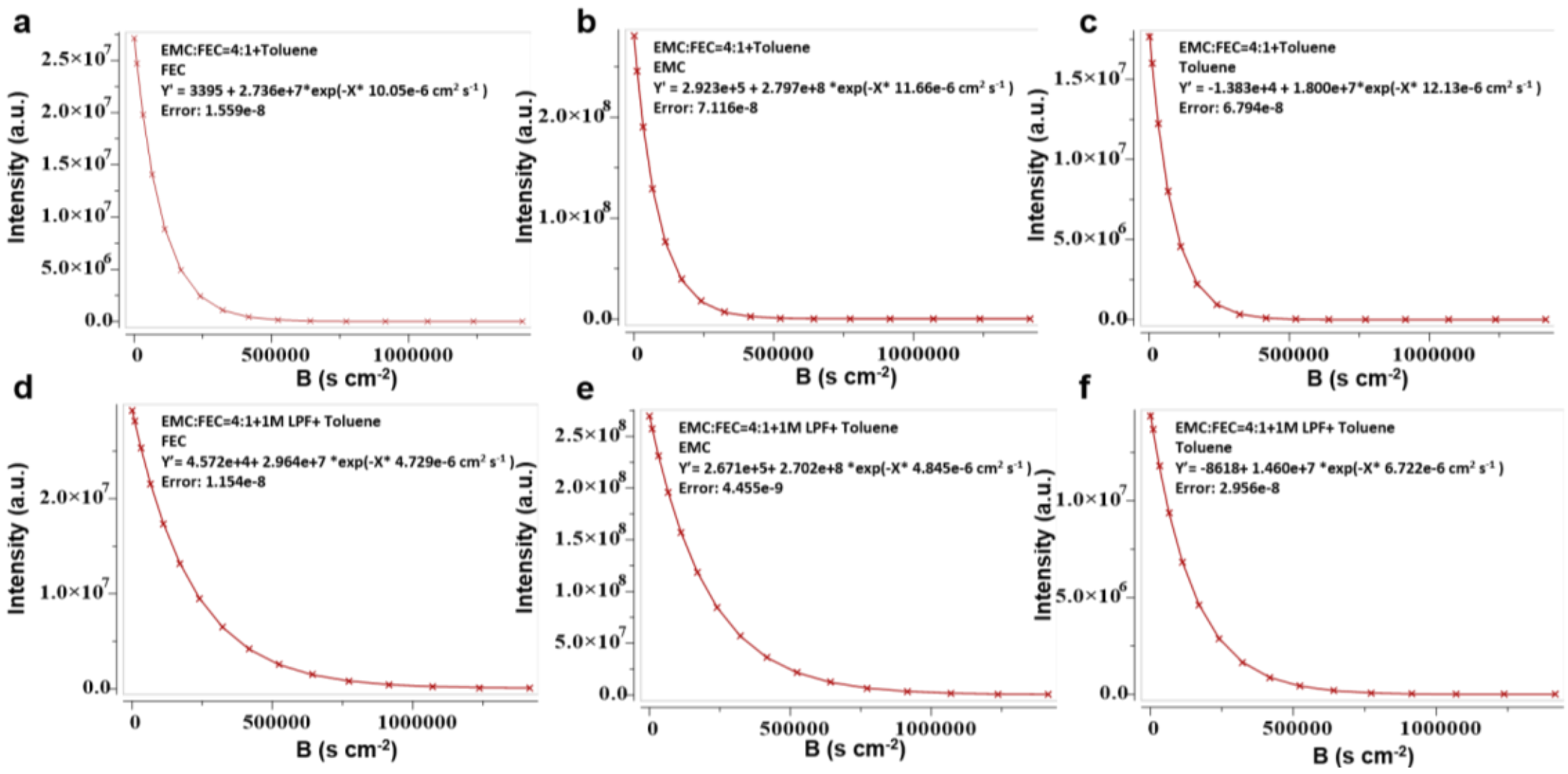

Supplementary Figure 10. DOSY-NMR fittings of signal intensity for BE electrolyte. (a) FEC, (b) EMC, (c) Toluene in EMC: FEC = 4:1 + Toluene (by bolume) system and (d) FEC, (e) EMC, (f) Toluene in EMC: FEC = 4:1 (by bolume) + 1M $LiPF_6$ + Toluene system.

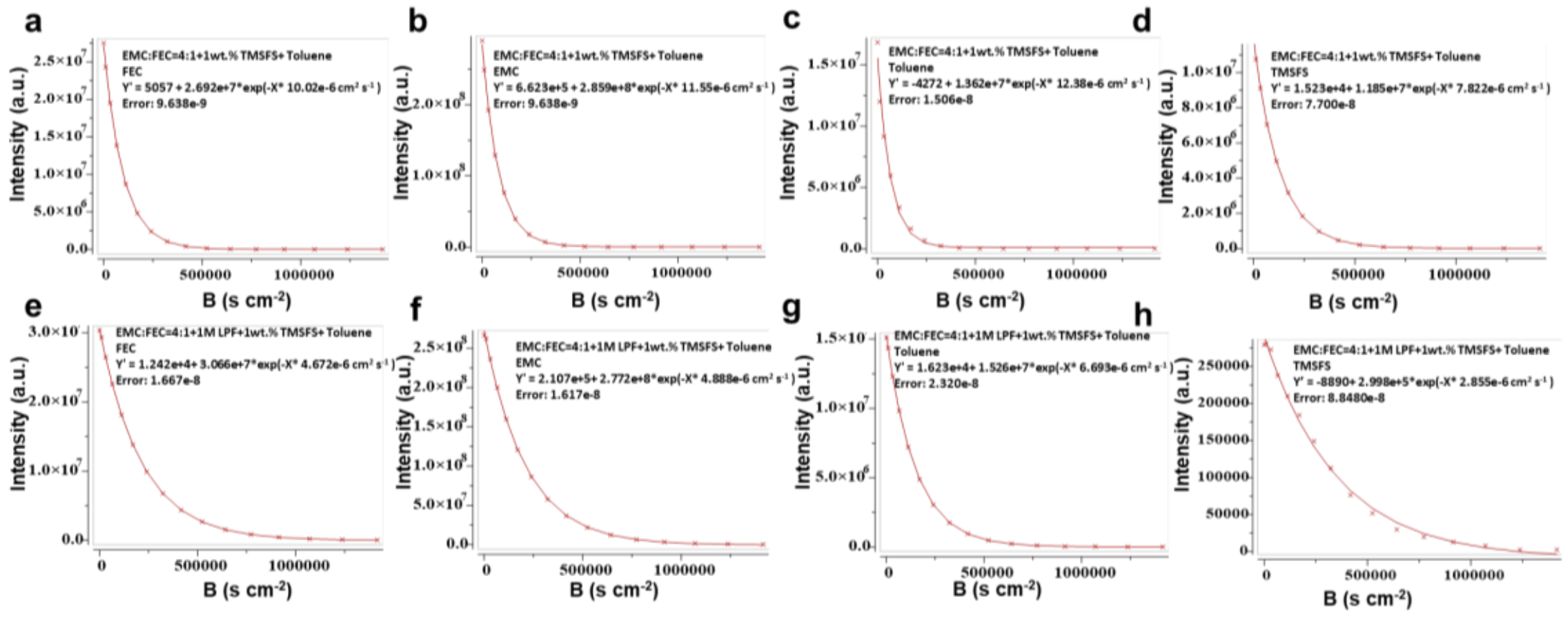

Supplementary Figure 11. DOSY-NMR fittings of signal intensity for TFFE. (a) FEC, (b) EMC, (c) Toluene, (d) TMSFS in EMC: FEC = 4:1+ 1wt.% TMSFS + Toluene (by bolume) system and (e) FEC, (f) EMC, (g) Toluene and (h) TMSFS in EMC: FEC = 4:1 (by bolume) + 1M $LiPF_6$ + 1wt.% TMSFS + Toluene system.

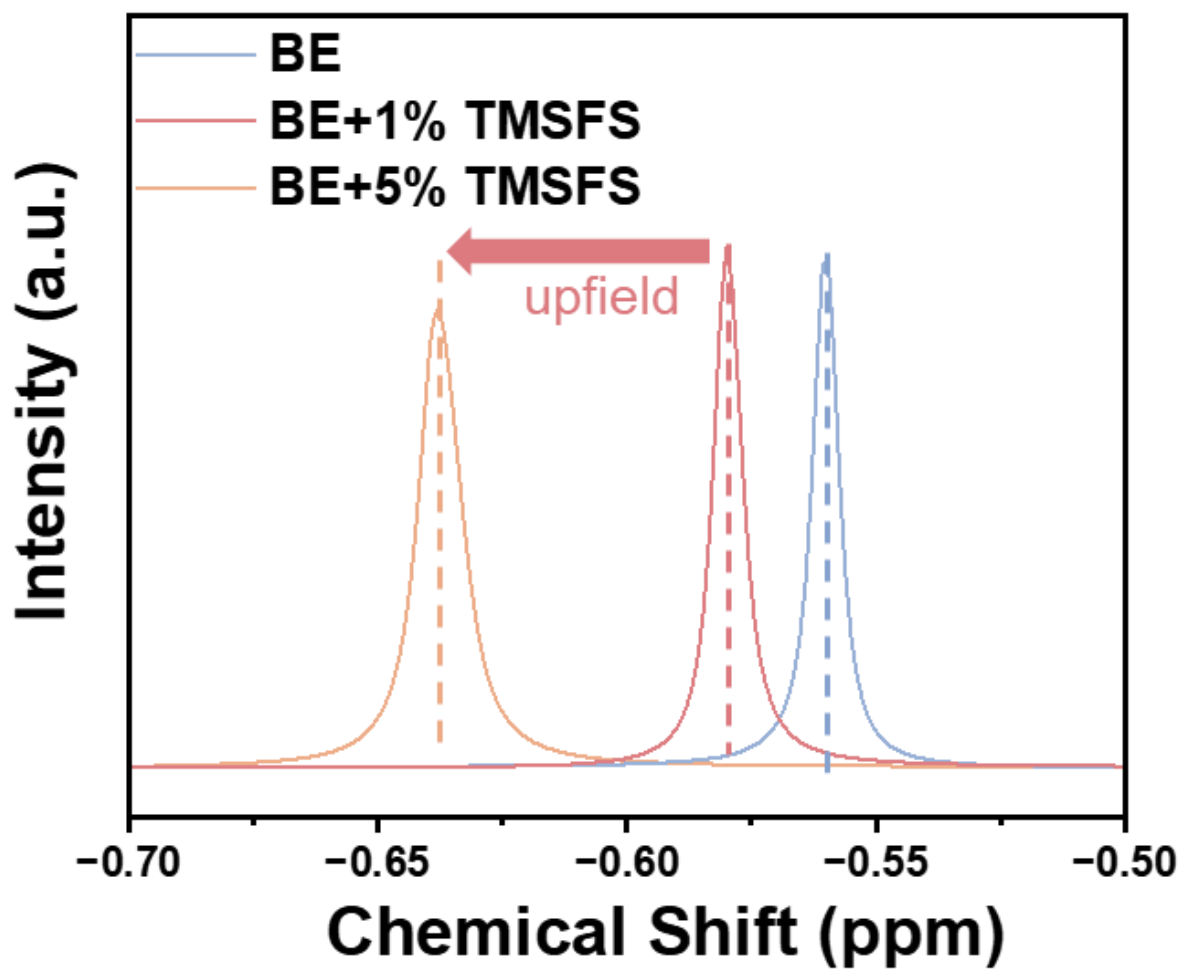

Supplementary Figure 12. DOSY-NMR fittings of signal intensity for TFFE.

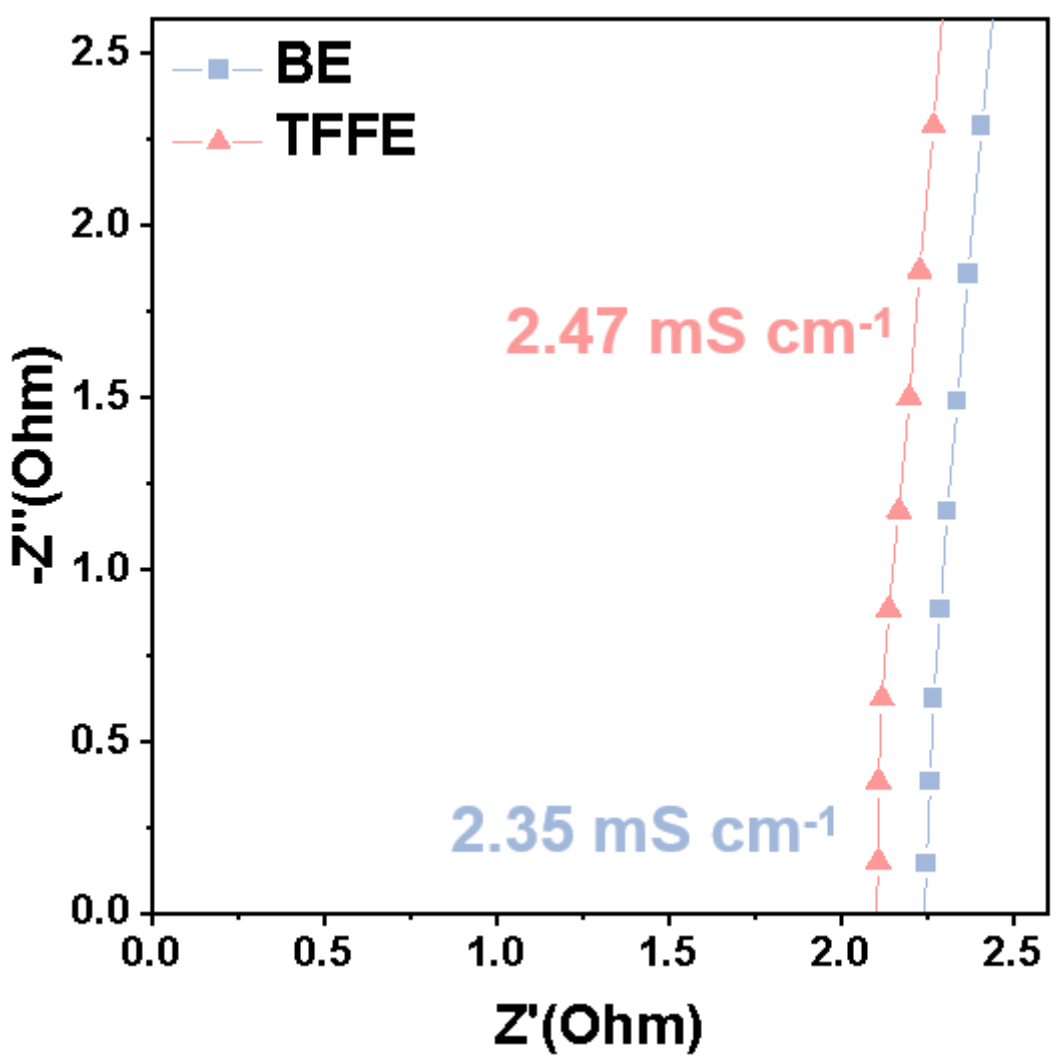

Supplementary Figure 13. Li^{+} conductivity test of BE and TFFE.

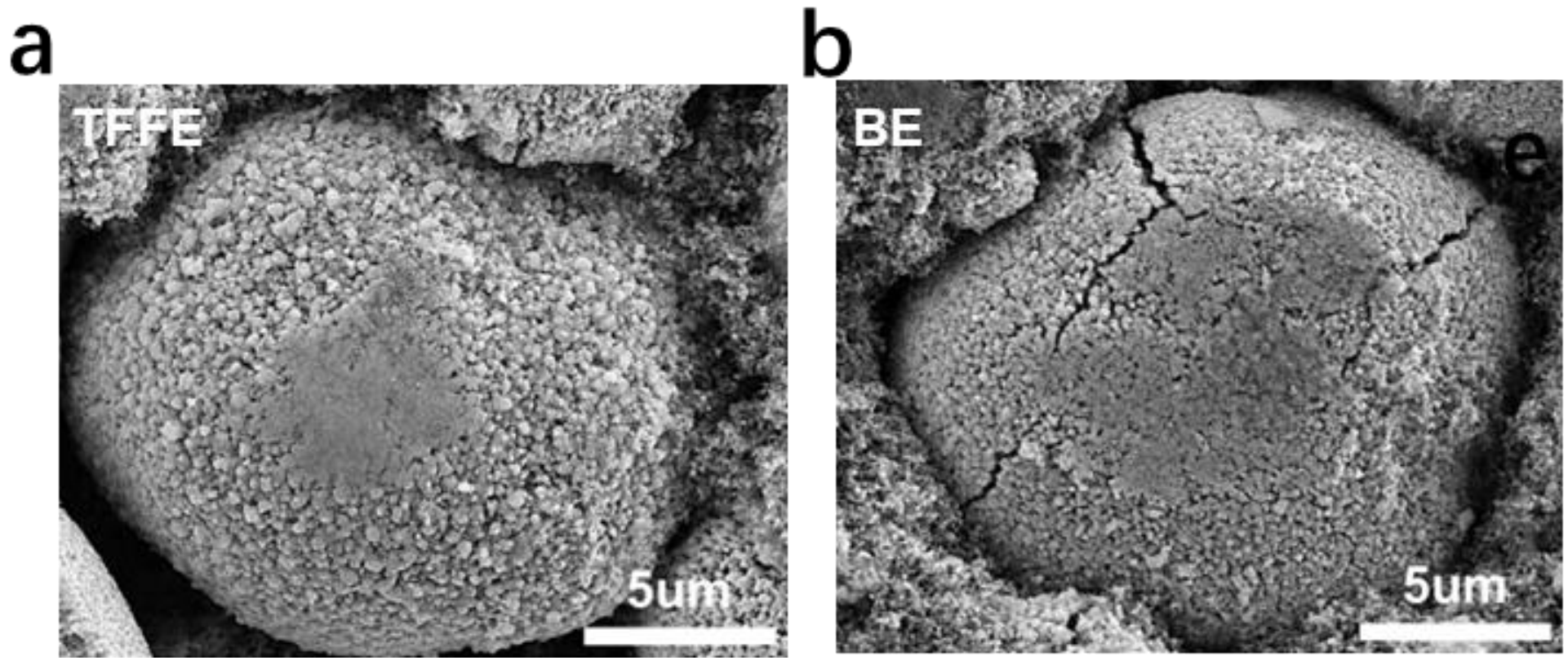

Supplementary Figure 14. (a) SEM image of the positive electrode surface from Li||LRMO full cell assembled with TFFE after 100 cycles at the condition of 0.9 mA cm^{-2}; (b) SEM image of the BE counterpart after 100 cycles at the condition of 0.9 mA cm^{-2}. The tests were performed at ambient temperature.

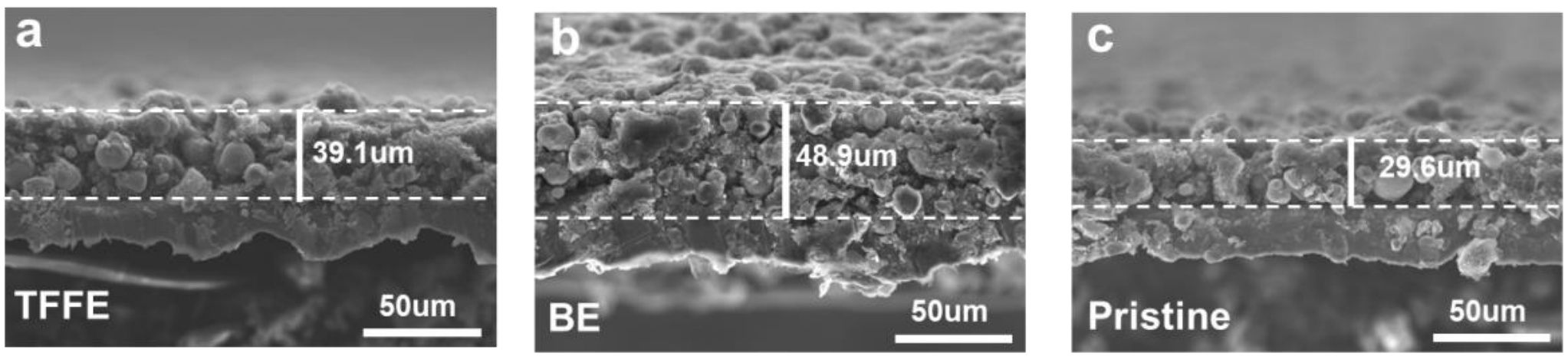

Supplementary Figure 15. Cross-section SEM images of post-cycled LRMO positive electrodes of (a) TFFE- (b) BE-based and (c) pristine full-cells after 100 cycles at the condition of 0.9 mA cm^{-2}. The tests were performed at 25℃±0.5 ℃.

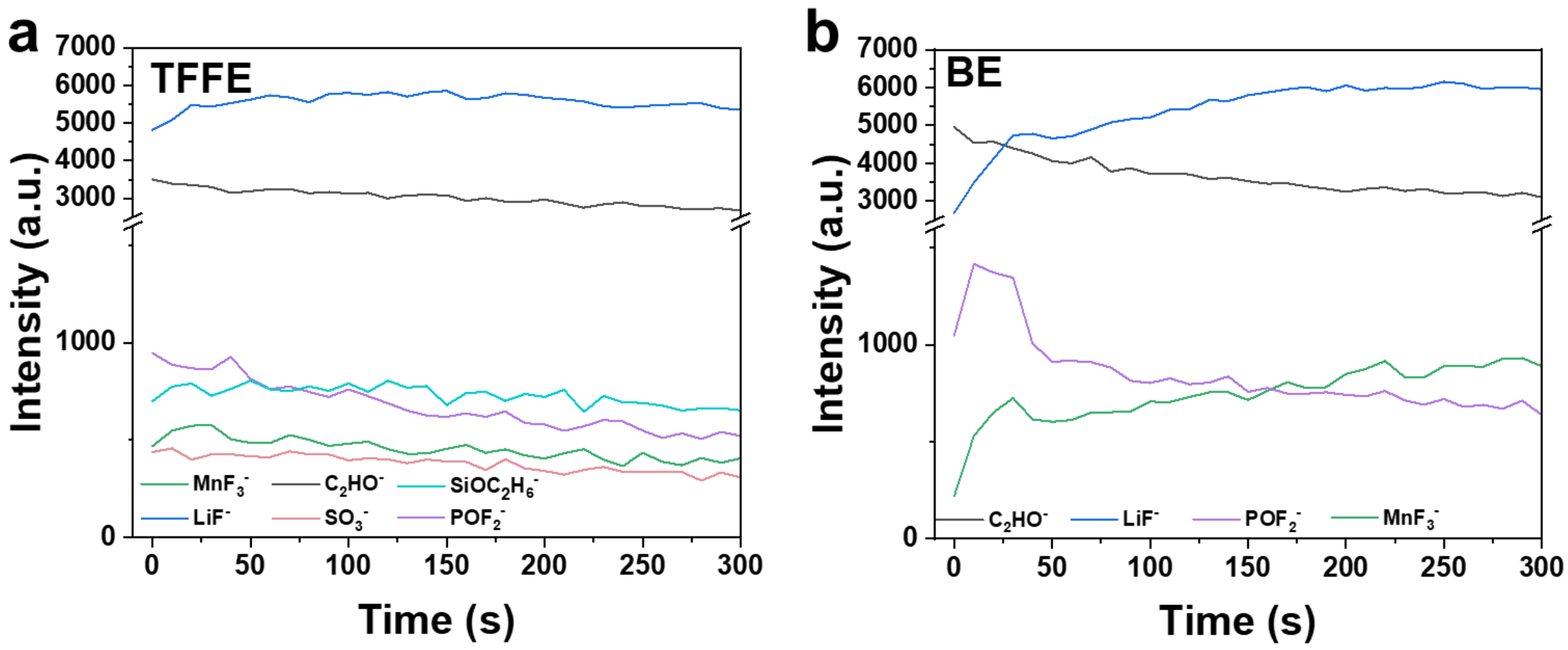

Supplementary Figure 16. (a) curves of selected secondary ion fragments of different CEI in (a)TFFE-based system and (b) BE-based system after 100 cycles at the condition of 0.9 mA cm^{-2}. The tests were performed at 25℃±0.5 ℃.

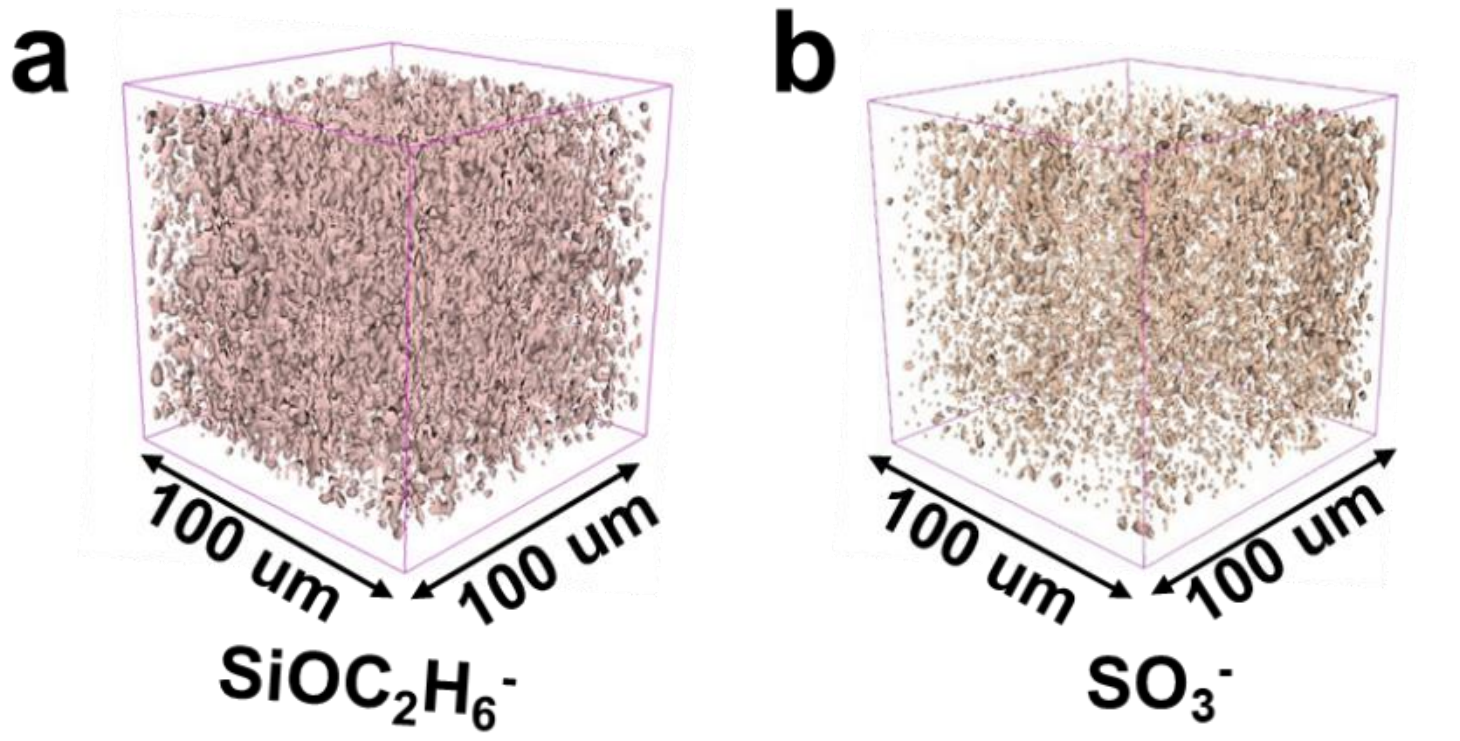

Supplementary Figure 17. (a) curves of selected secondary ion fragments of (a) $SiOC_2H_6^-$ and (b) SO_3^- in TFFE.

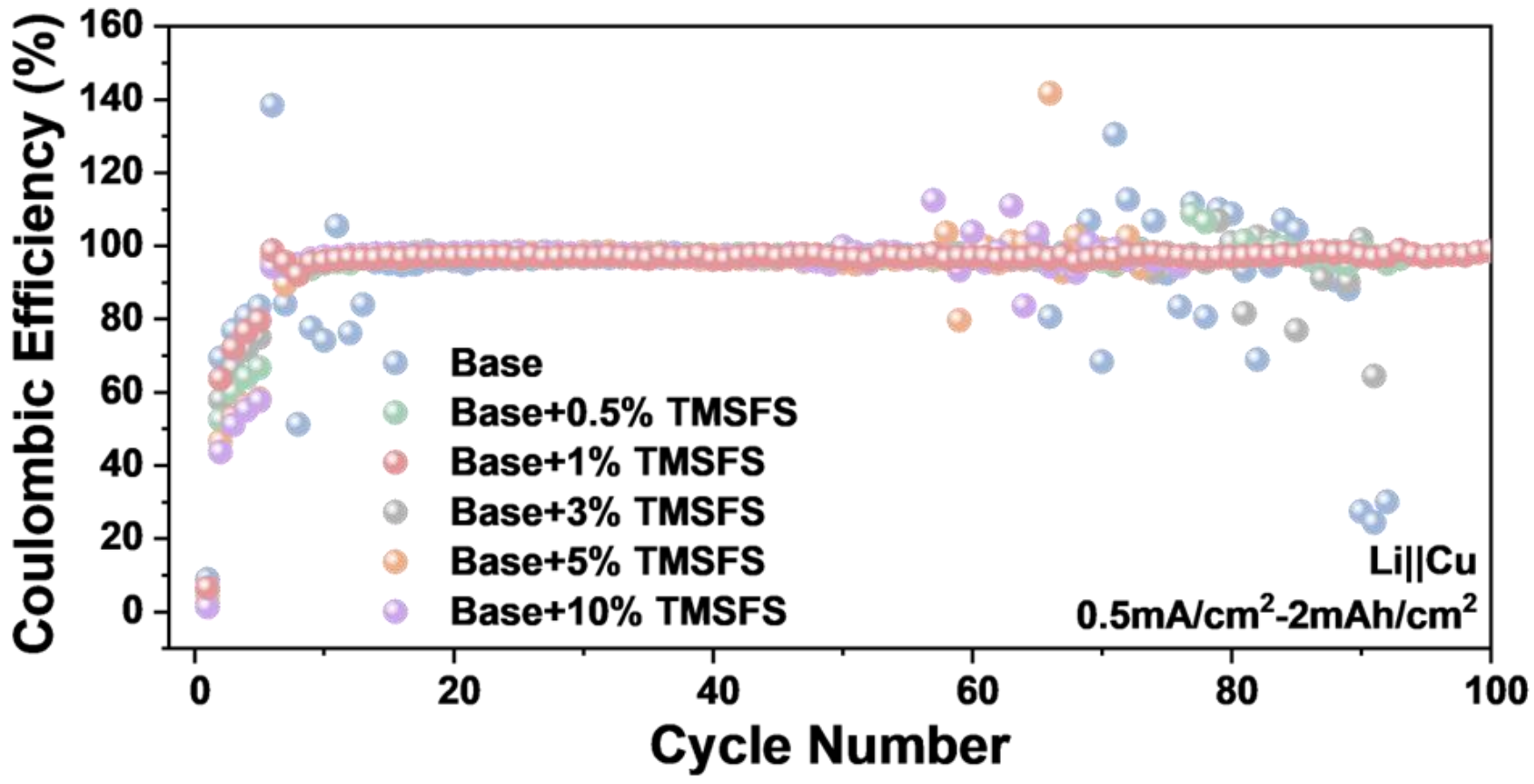

Supplementary Figure 18. Cycling stability of BE and different amounts of TMSFS based Li||Cu half-cell at 0.5 mA cm^{-2} and 2 mAh cm^{-2};

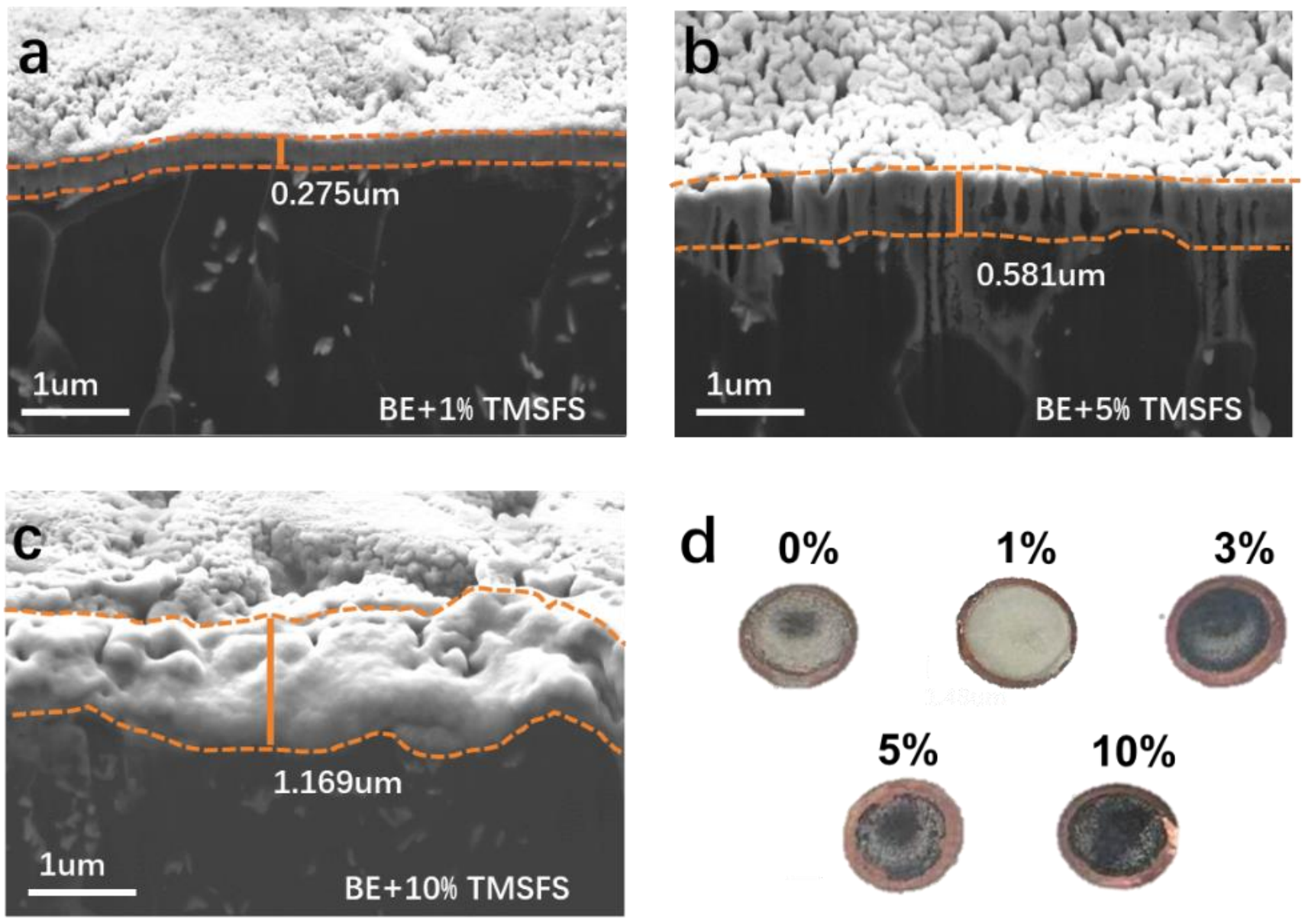

Supplementary Figure 19. Cross-section FIB-SEM images of interface reaction layer after 3 mAh cm^{-2} of Li depositing on Cu foil at 0.5 mA cm^{-2} after 50 cycles of (a) BE+1wt.% TMSFS (TFFE), (b) BE+5wt.% TMSFS, (c) BE+10wt.% TMSFS and (d) corresponding shots of surface states.

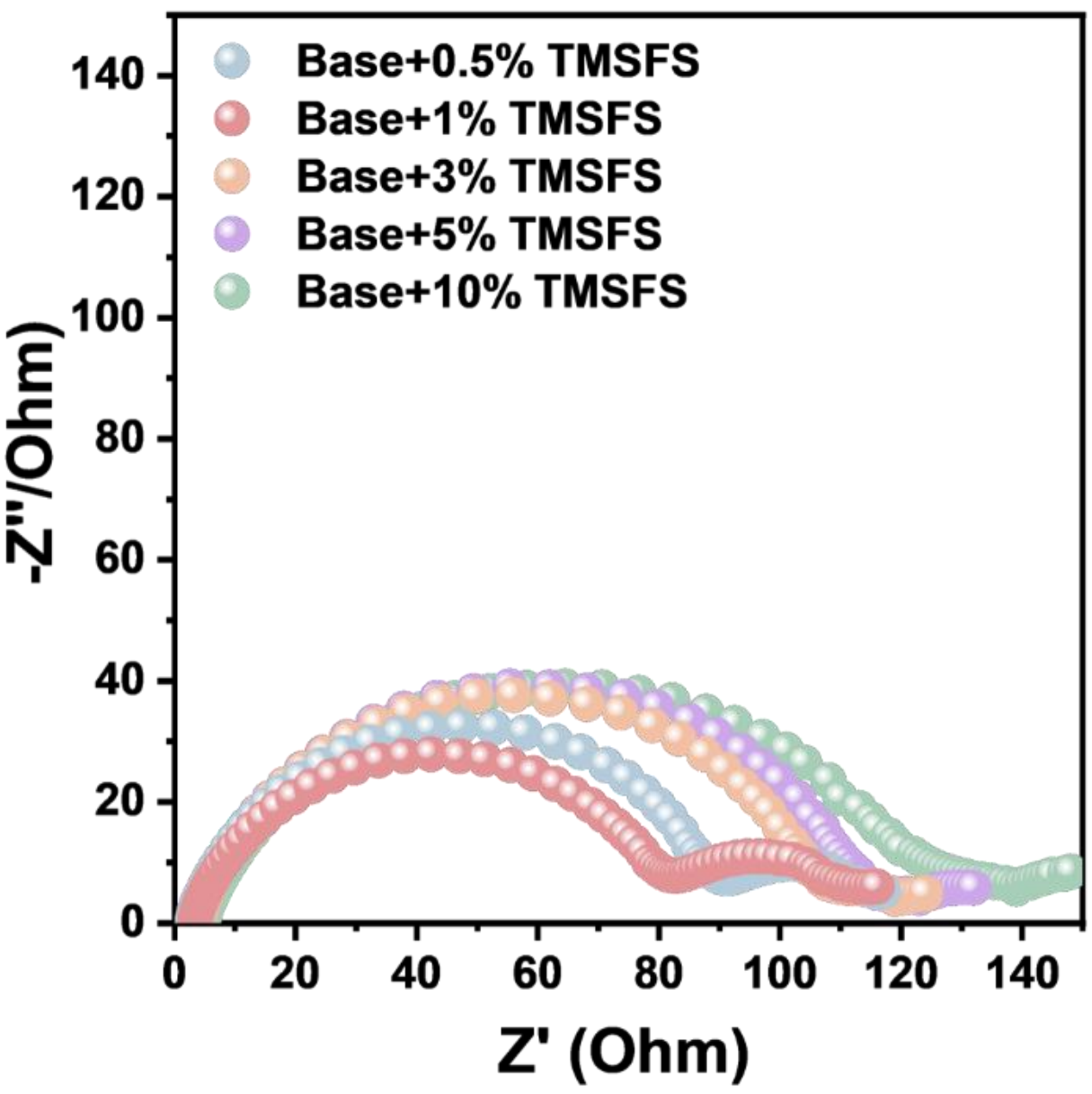

Supplementary Figure 20. EIS spectra measurements of cells after 3 mAh cm^{-2} of Li depositing on Cu foil at 0.5 mA cm^{-2} by BE+ different amounts of TMSFS.

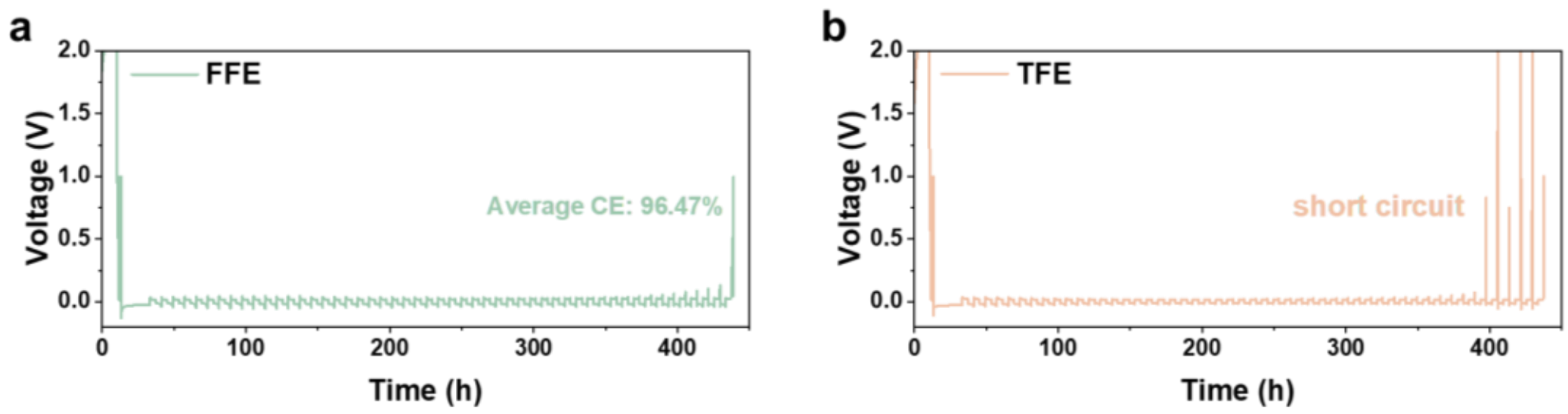

Supplementary Figure 21. Average CE of (a)FFE- and (b)TFE-based Li||Cu half-cells over 50 cycles at 0.5 mA cm^{-2} and 2 mAh cm^{-2}.

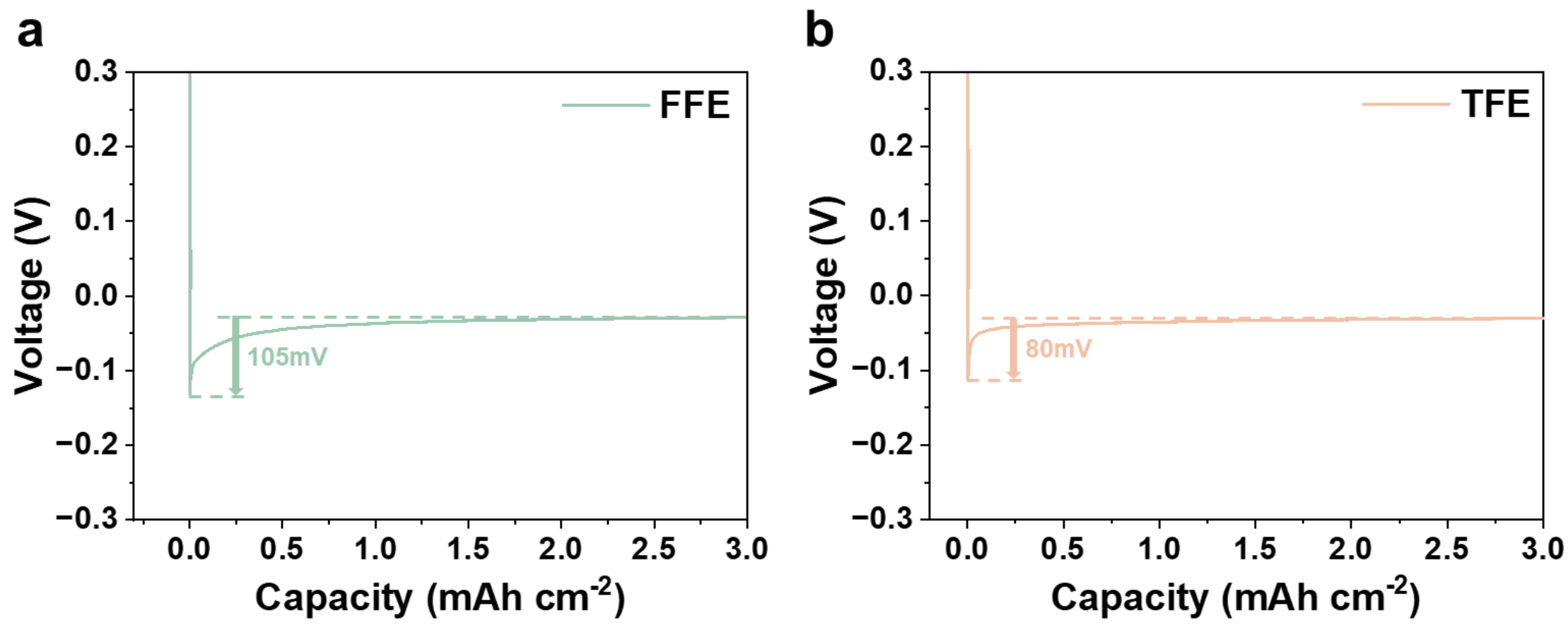

Supplementary Figure 22. Voltage-areal capacity profiles during the nucleation process for (a)FFE- and (b)TFE-based half-cells.

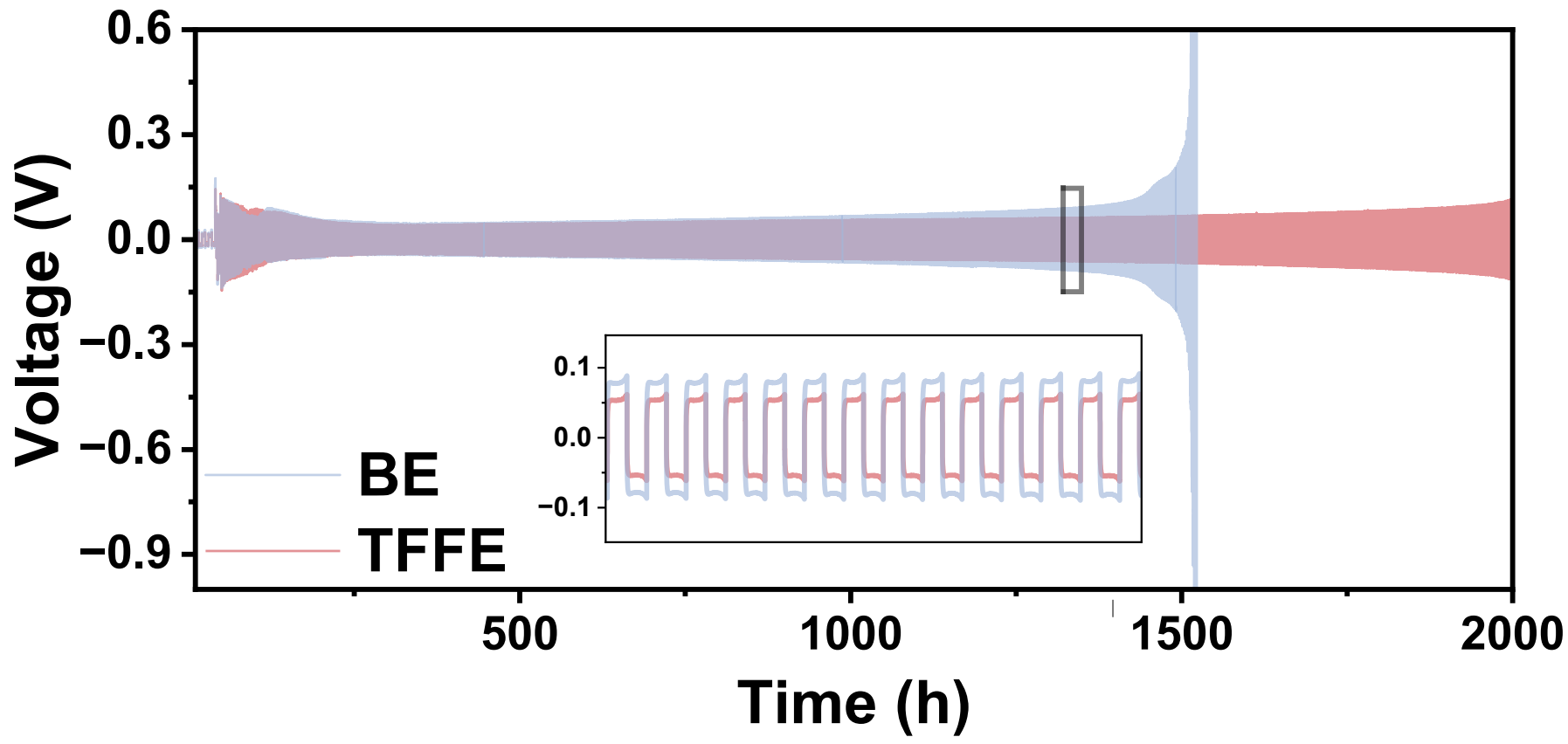

Supplementary Figure 23. Li plating/strippingstability of BE- and TFFE-assembled Li||Li symmetric cells at 1 mA cm^{-2} and 1 mAh cm^{-2}.

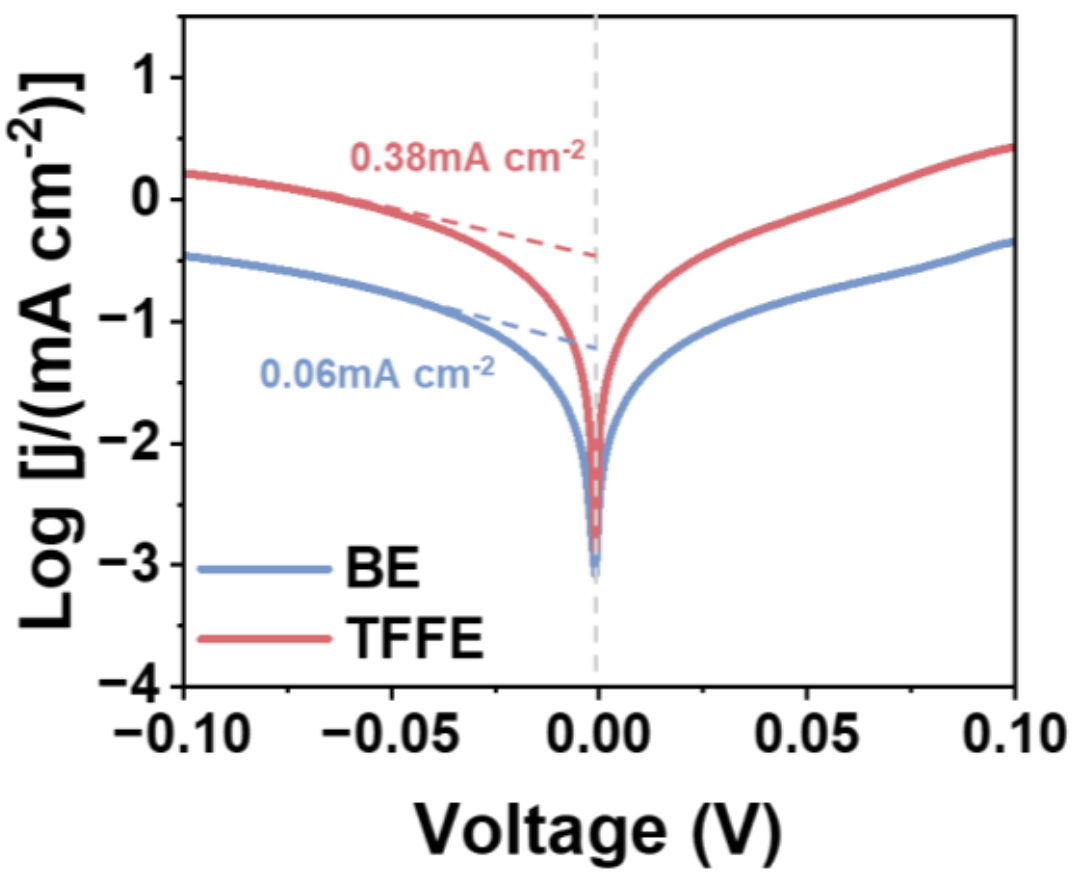

Supplementary Figure 24. Tafel plots of BE- and TFFE-based Li||Li symmetric cells tested at 1 mV s^{-1}.

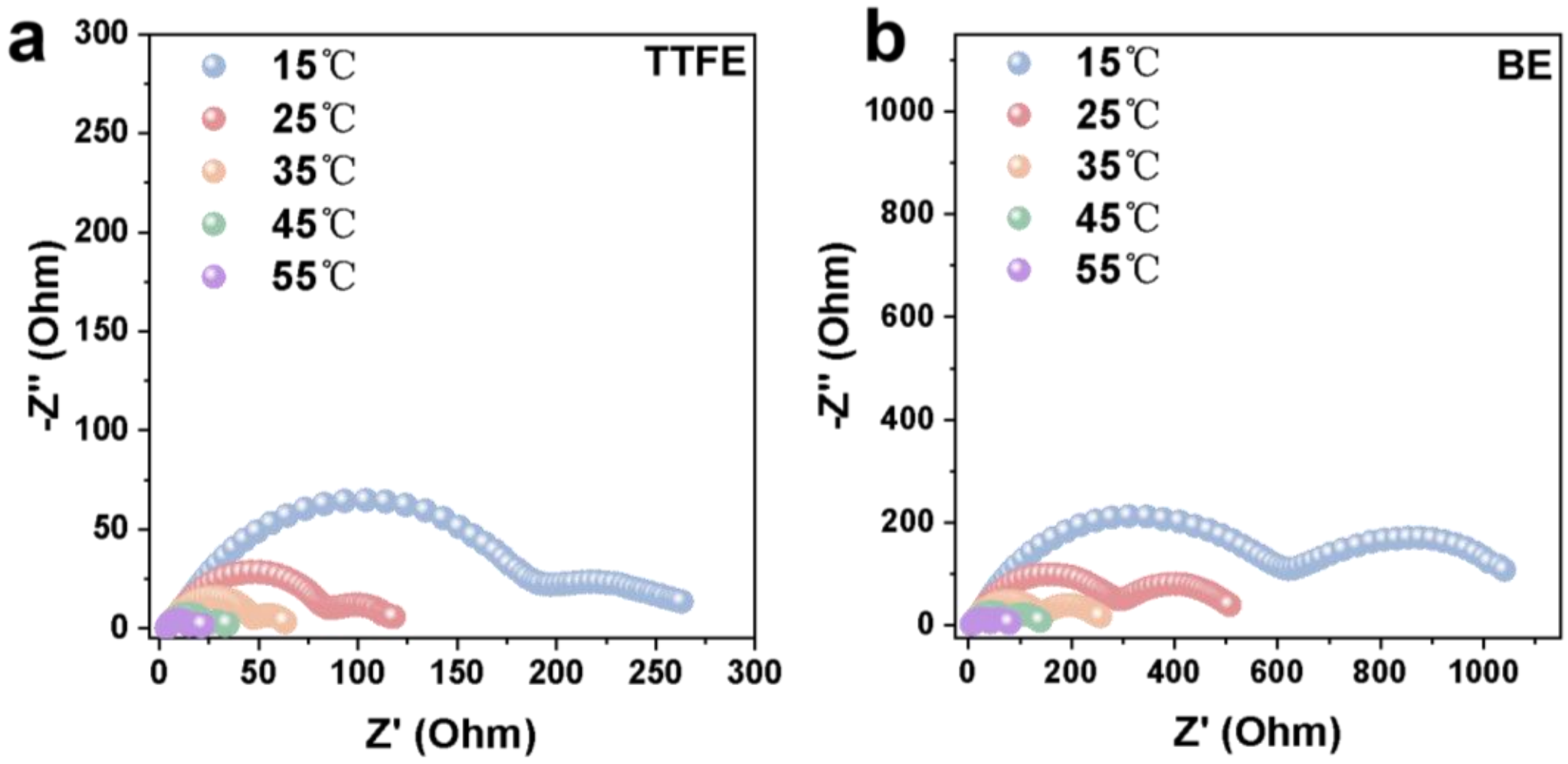

Supplementary Figure 25. Temperature-dependent EIS spectra of the as-formed SEI in (a) TFFE- and (b)BE-based Li||Cu half-cells after 3 mAh cm^{-2} of Li depositing on Cu electrodes at 0.5 mA cm^{-2}.

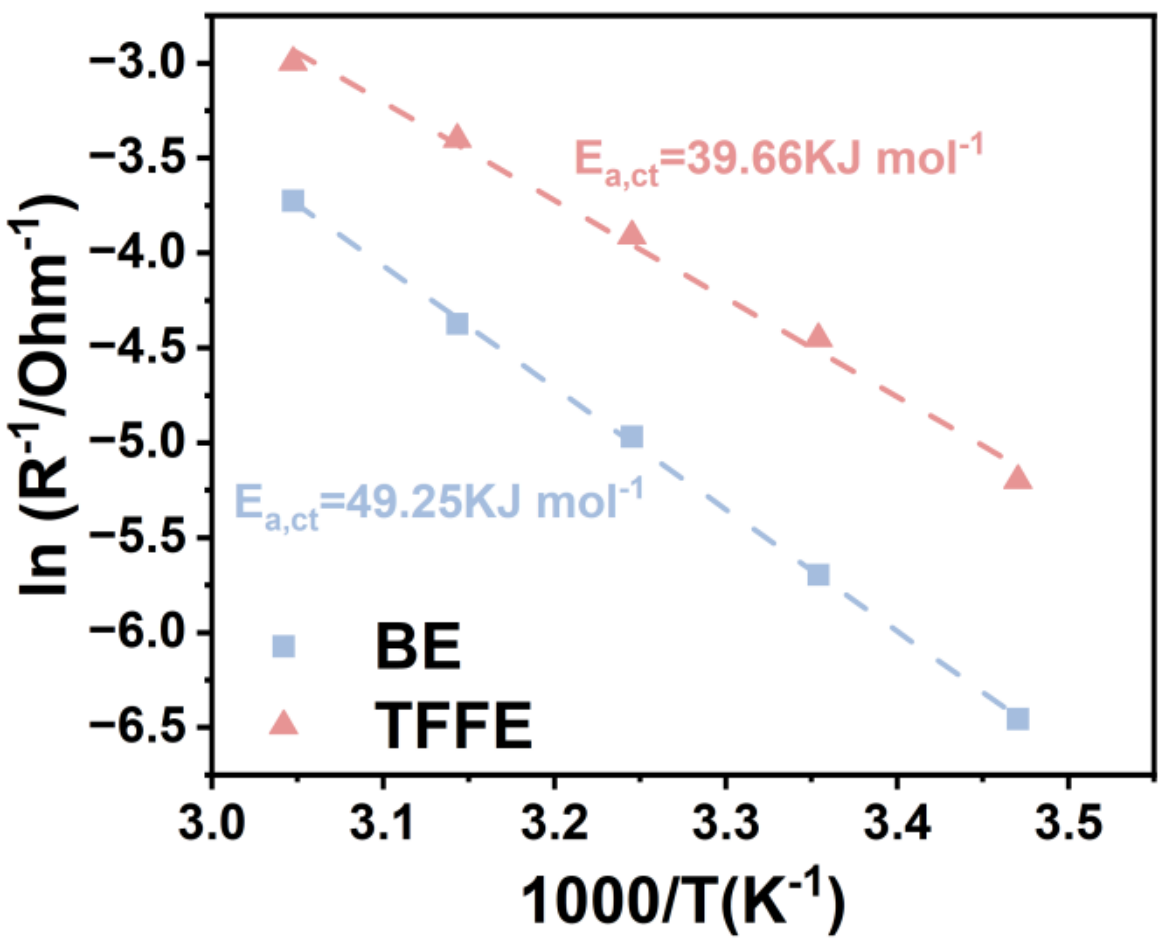

Supplementary Figure 26. Arrhenius plots of charge transfer resistance (R_{ct}) of the as-formed SEI in BE- and TFFE-based Li||Cu half-cells after 3 mAh cm^{-2} of Li depositing on Cu electrodes at 0.5 mA cm^{-2}.

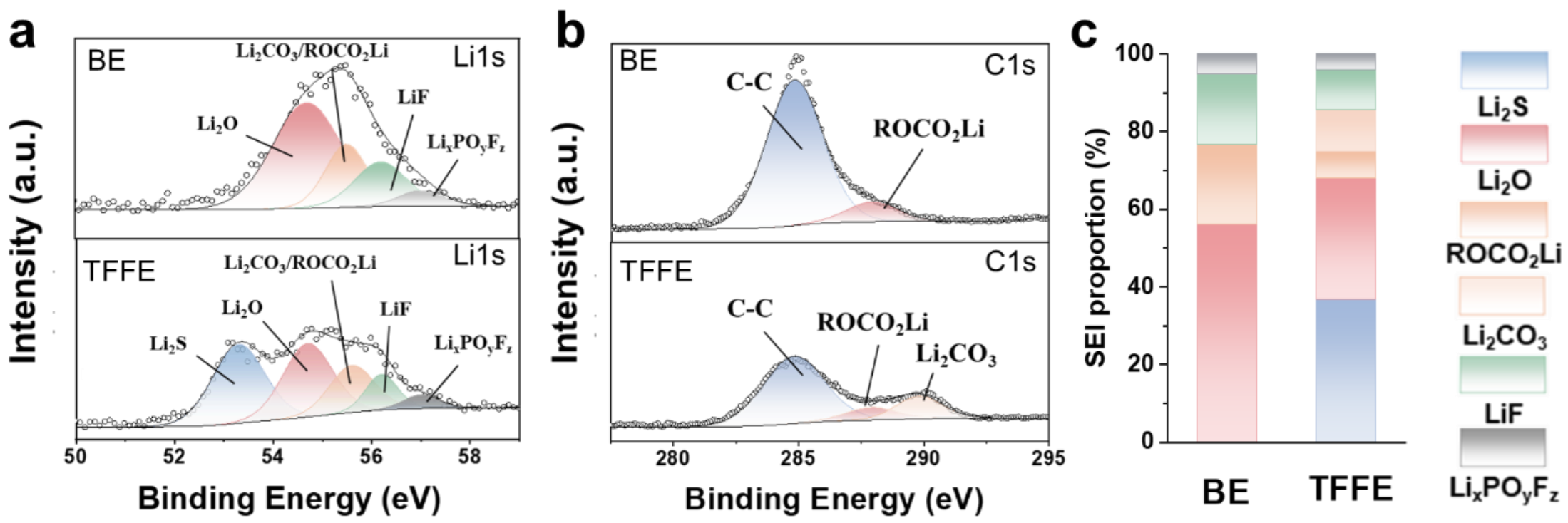

Supplementary Figure 27. Surface XPS spectra of (a)Li 1s, (b)C 1s regions in the SEI layers of 3 mAh cm^{-2} of Li deposits at 0.5 mA cm^{-2} from BE and TFFE systems (c) proportion of different components of SEI.

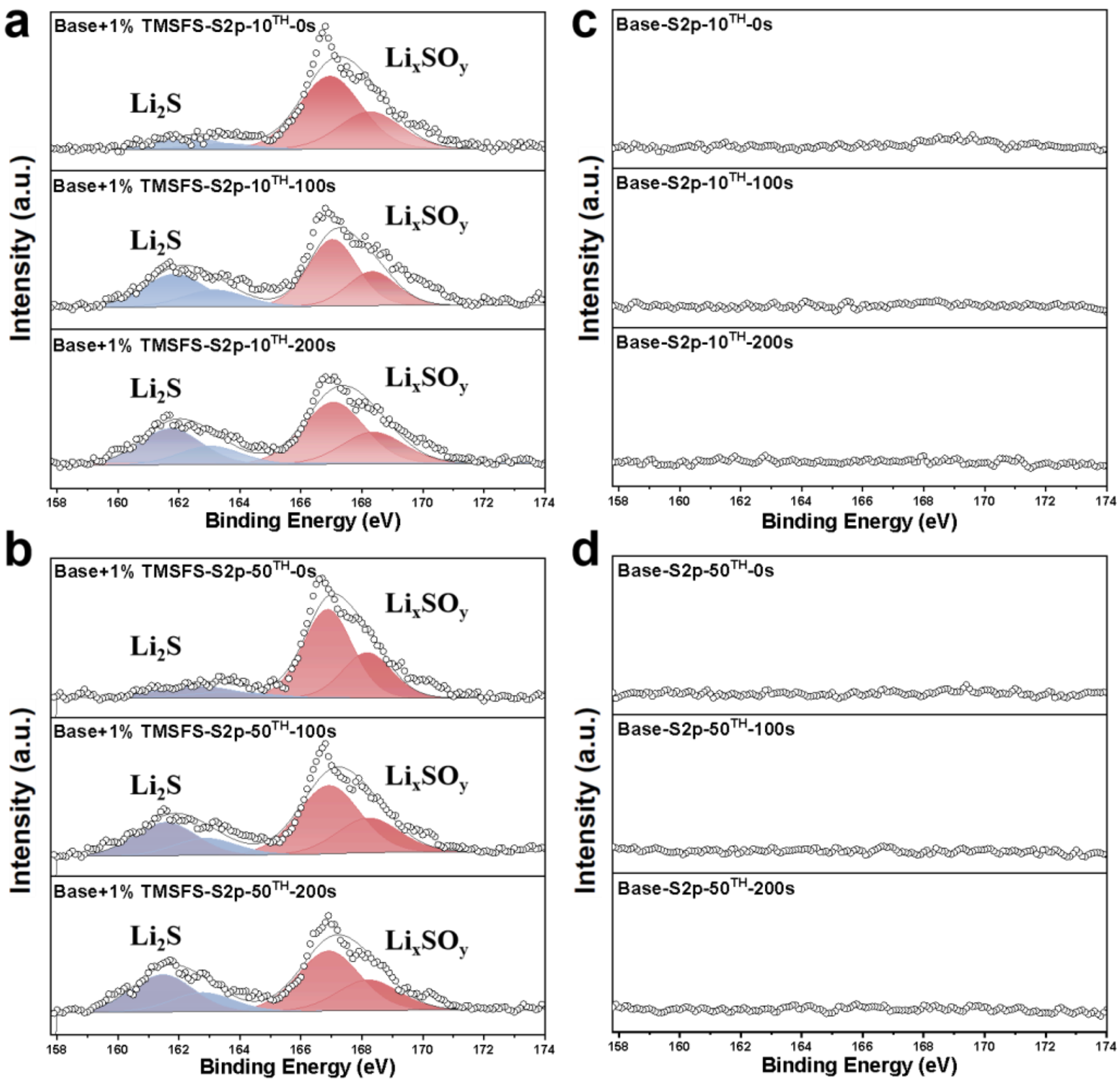

Supplementary Figure 28. Depth-profile XPS of S2p regions in the SEI layers after 3 mAh cm^{-2} of Li deposits at 0.5 mA cm^{-2} from (a) TFFE (Base+1% TMSFS) after 10 cycles (b) 50 cycles and (c) BE (Base) systems after 10 cycles and (d)50 cycles.

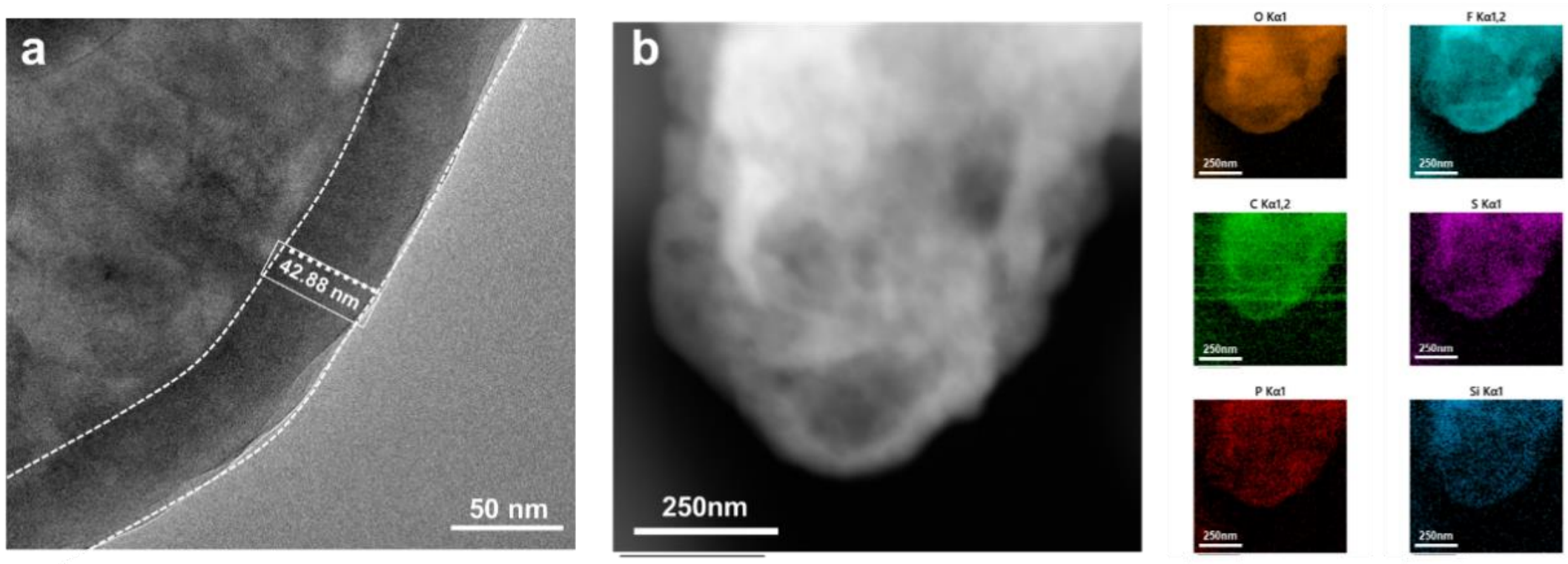

Supplementary Figure 29. (a) Cryo-TEM images of 3 mAh cm^{-2} of Li deposits on Cu electrode at 0.5 mA cm^{-2} and the schematic illustrations of SEI for TFFE and (b)corresponding element mapping of SEI.

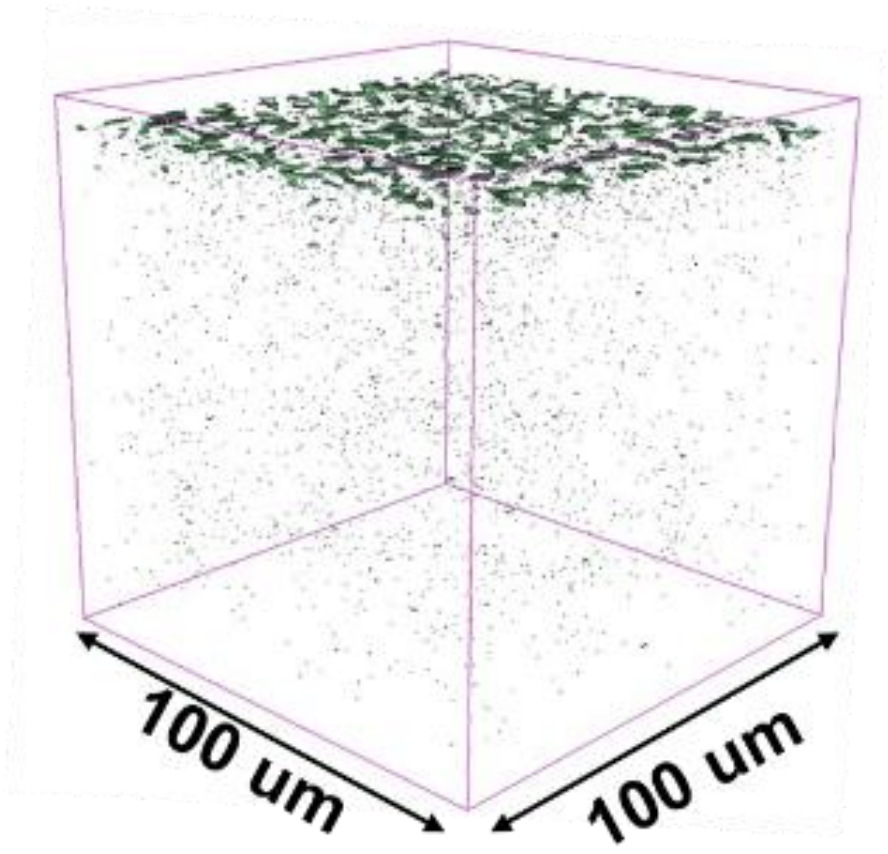

Supplementary Figure 30. TOF-SIMS 3D renderings of $SiC_3H_9^-$ distributions in the SEI layers of Li||Cu electrodes after cycling in TFFE.

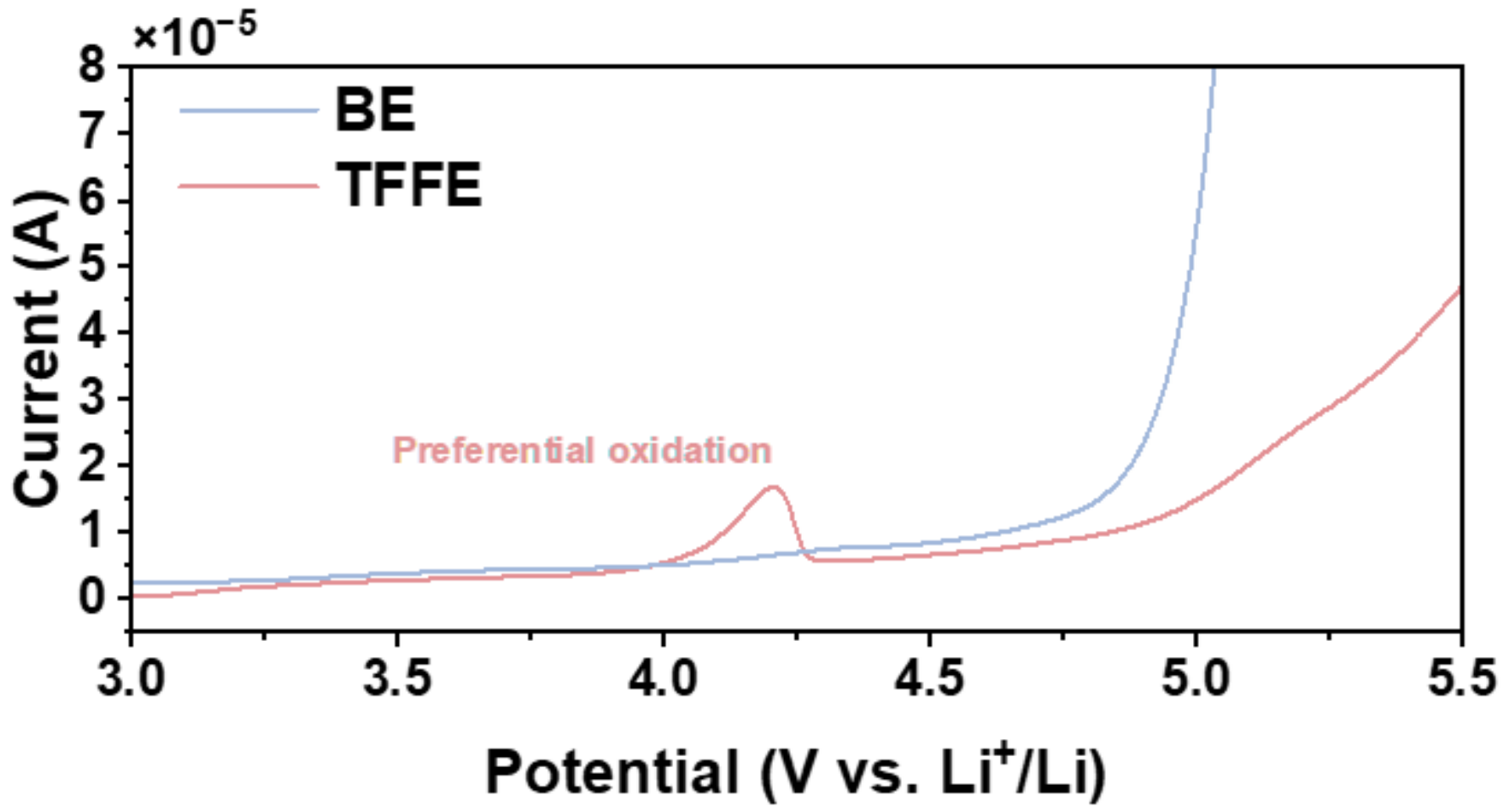

Supplementary Figure 31. LSV curves of Li||SS cells in BE and TFFE systems, with a scan rate of 0.1 mV s^{-1}, the test was performed in a two-electrode configuration, with a lithium metal as the reference electrode and a stainless steel as the working electrode.

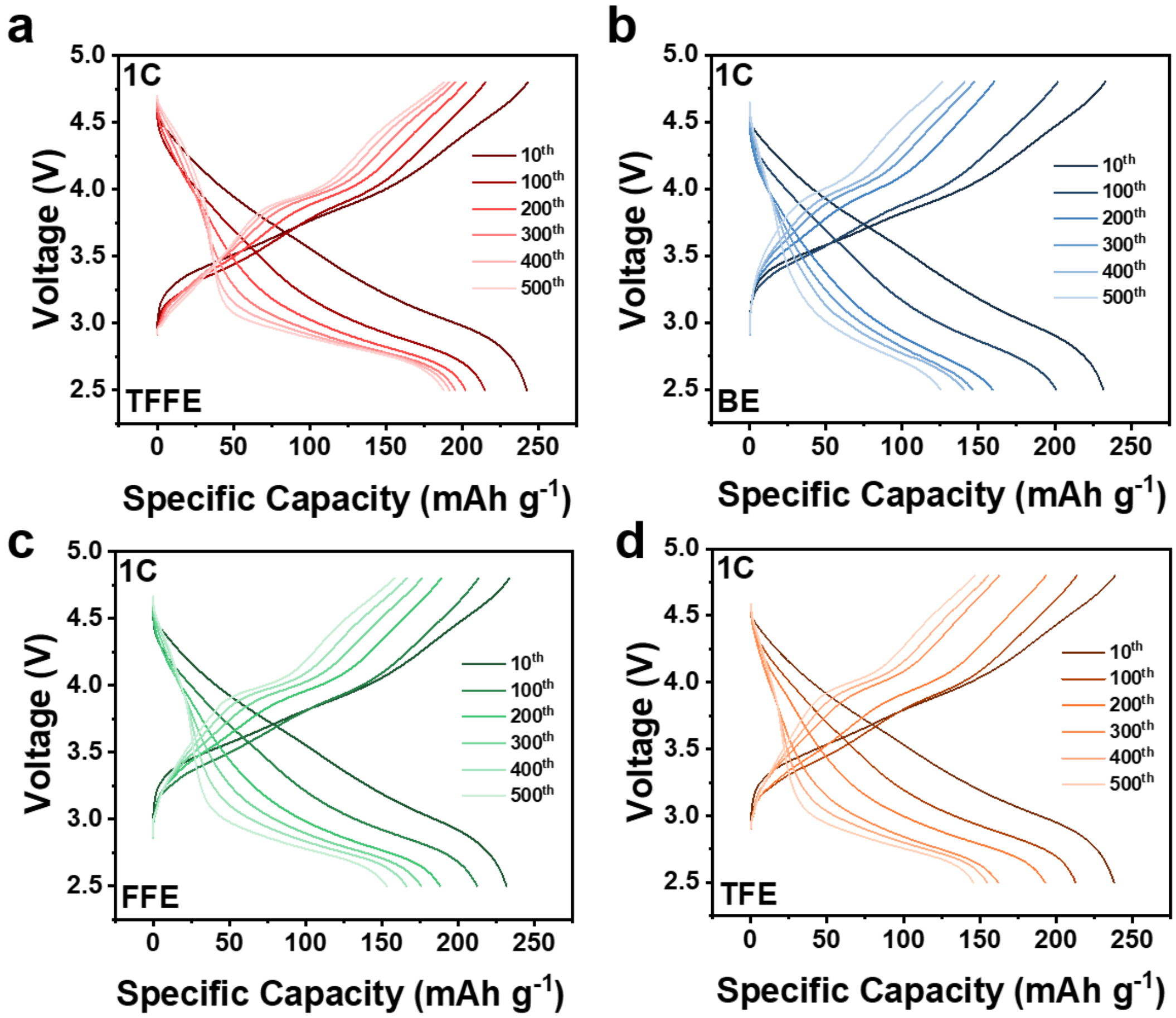

Supplementary Figure 32. Charge/discharge curves at 1C of Li||LRMO full cells assembled with (a)TFFE, (b)BE, (c)FFE and (d)TFE from 2.5V to 4.8V under different cycles, respectively.

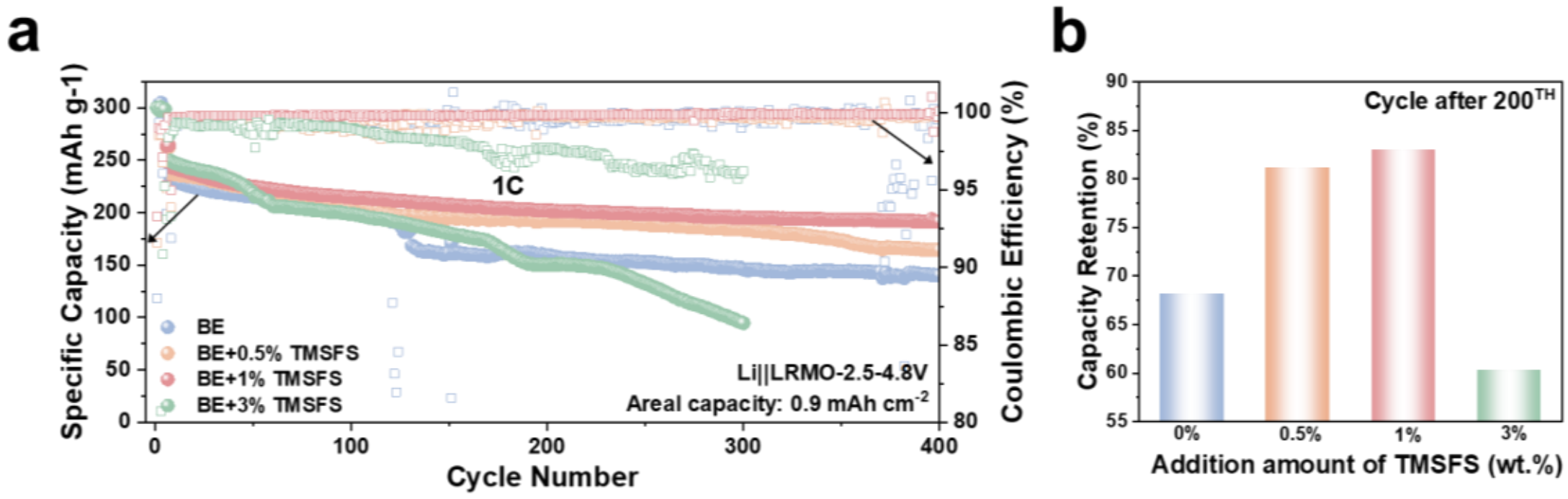

Supplementary Figure 33. (a)Li||LRMO Full coin cells assembled with different content of TMSFS and (b)corresponding capacity retention after 200 cycles.

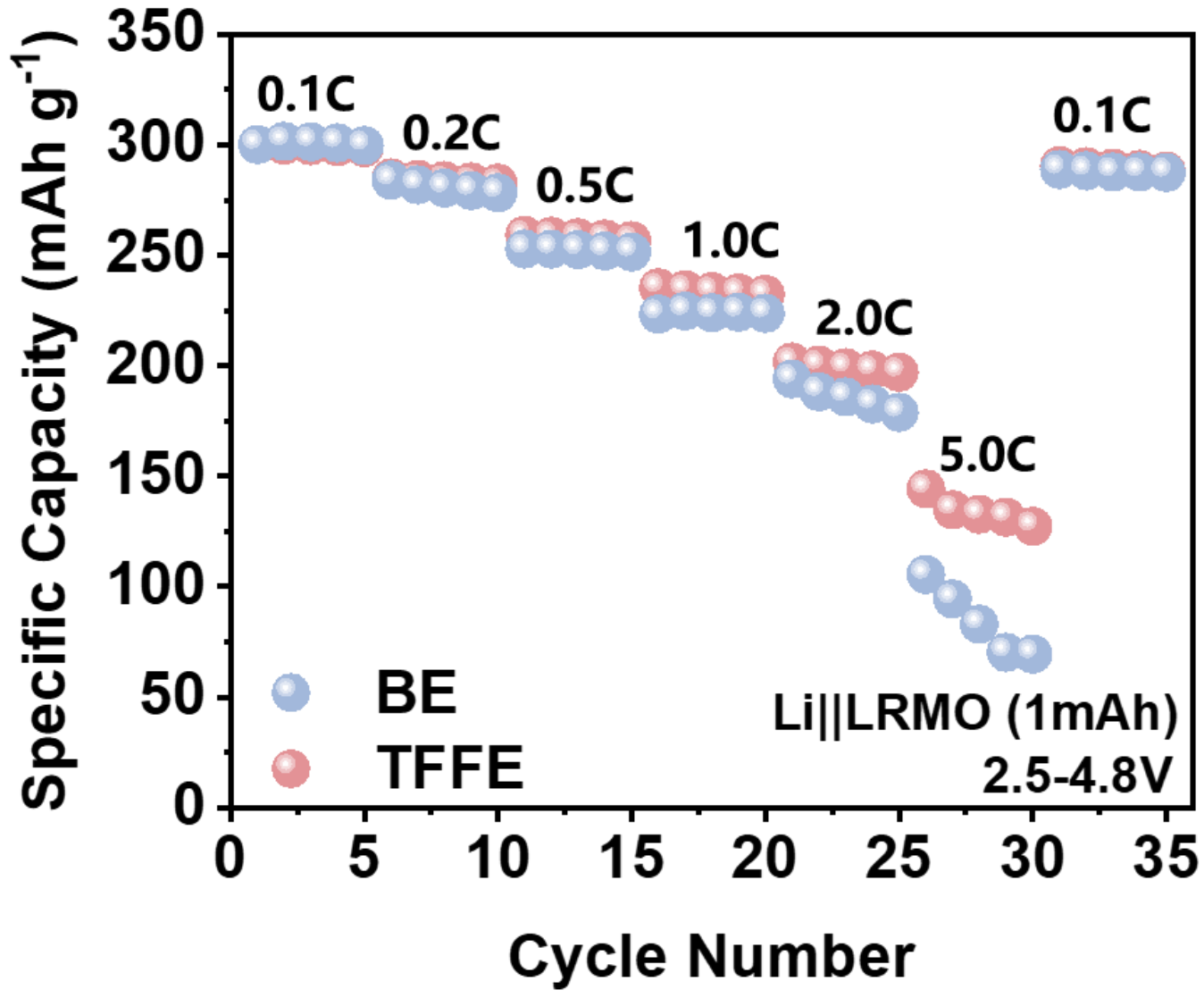

Supplementary Figure 34. Rate capability of Li||LRMO full cells assembled with BE and TFFE electrolytes.

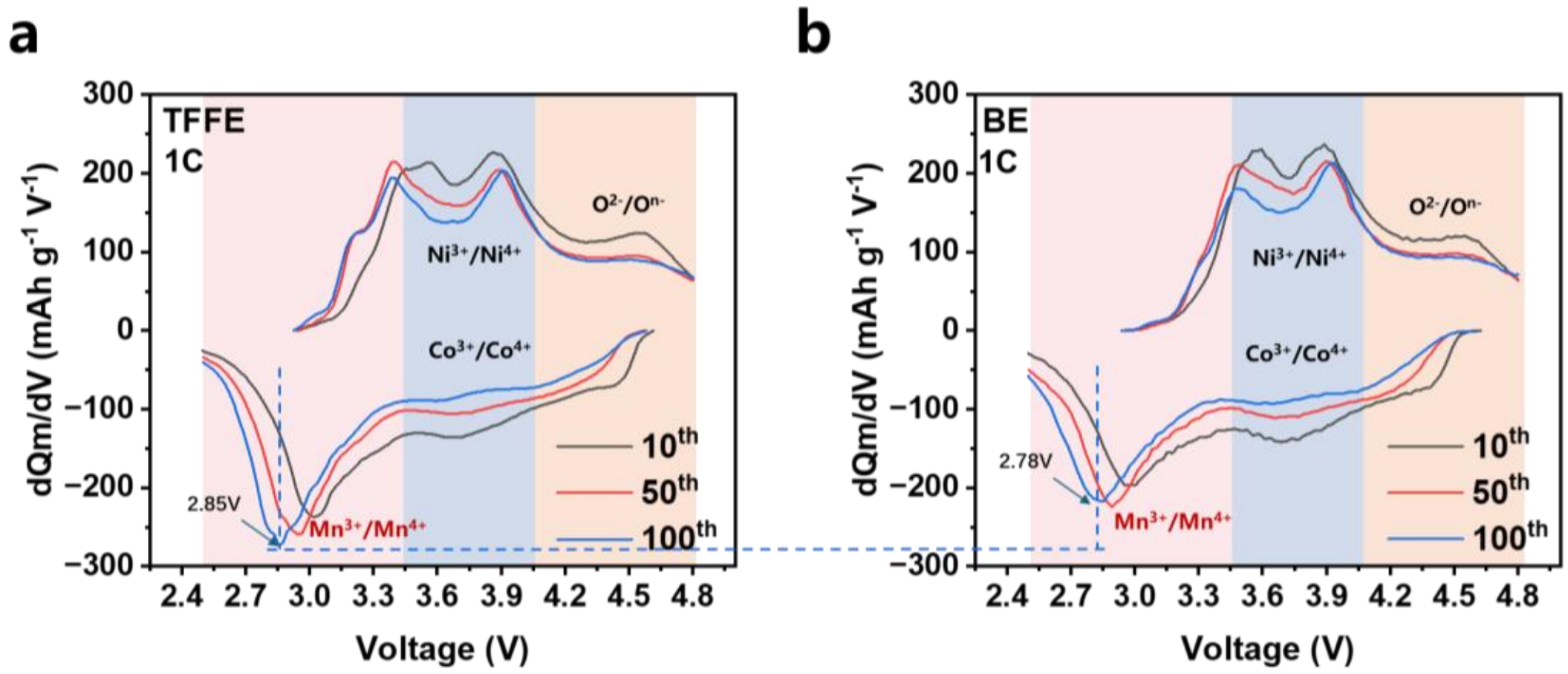

Supplementary Figure 35. dQ/dV-Voltage of Li||LRMO full cell from 10-100 cycles at 1C of (a)TFFE and (b) BE system .

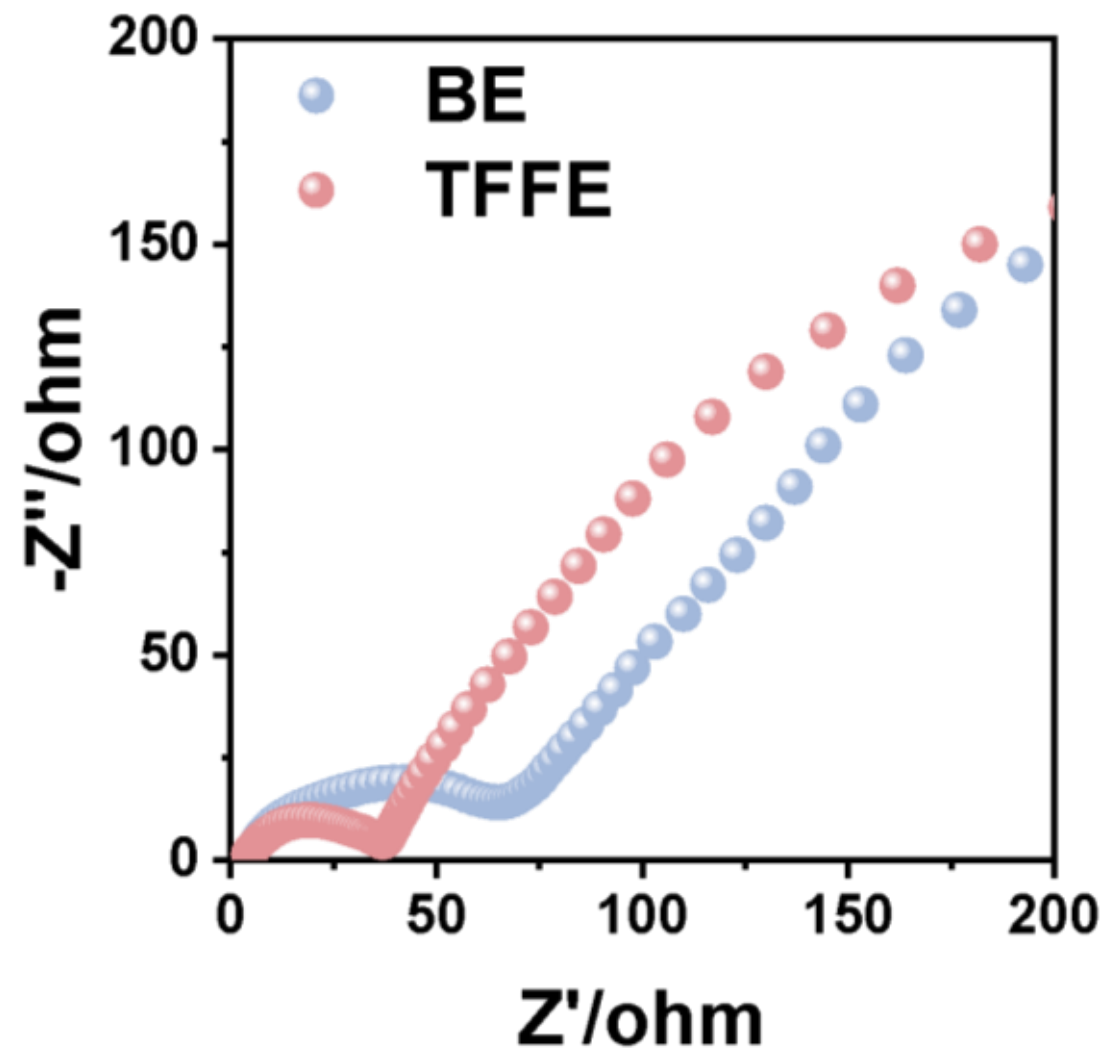

Supplementary Figure 36. Electrochemical impedance spectroscopy of Li||LRMO batteries with different electrolytes.

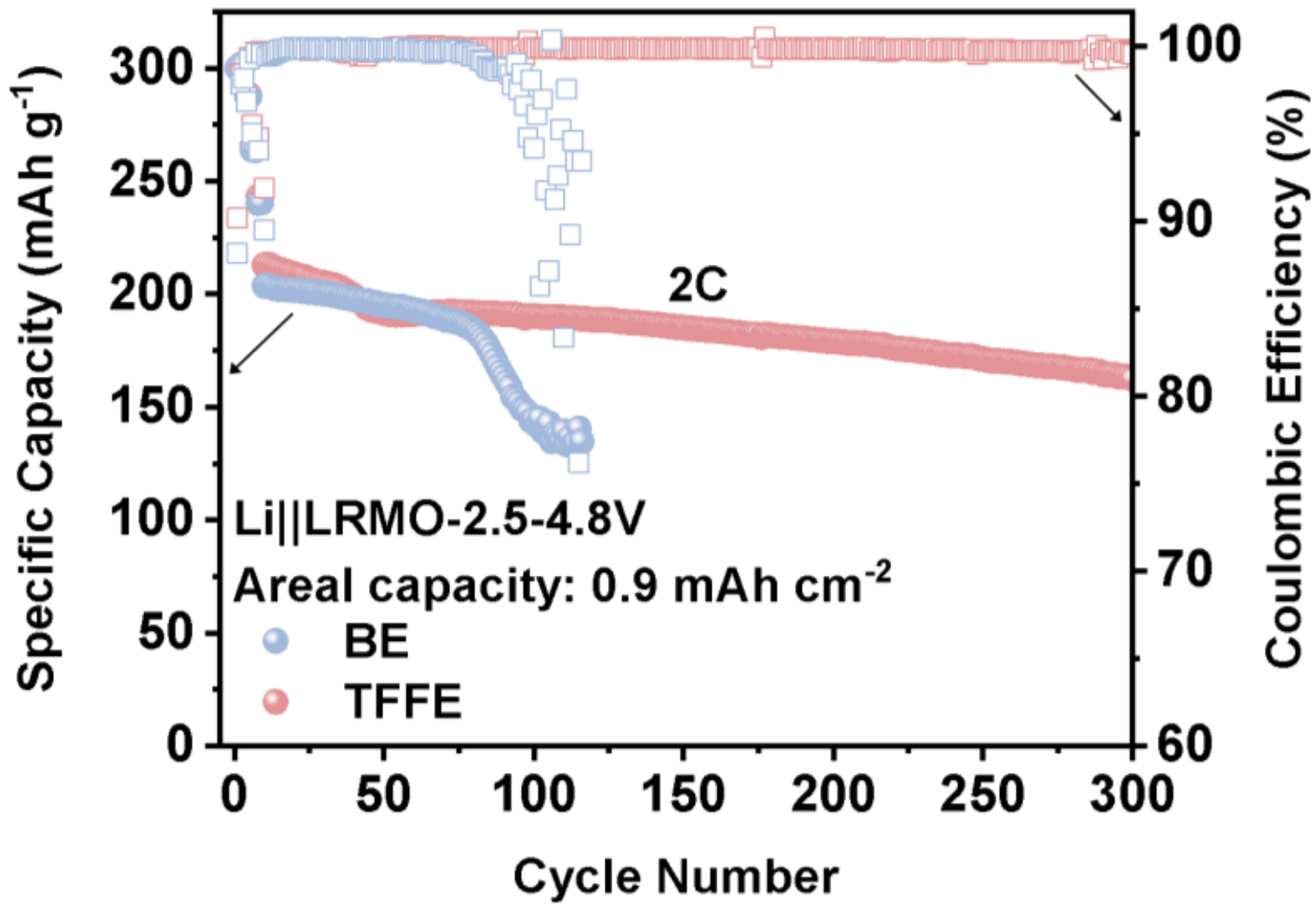

Supplementary Figure 37. Cycling stability and Coulombic efficiency of Li||LRMO full cells assembled with BE and TFFE electrolytes, tested between 2.5 and 4.8 V at 2C.

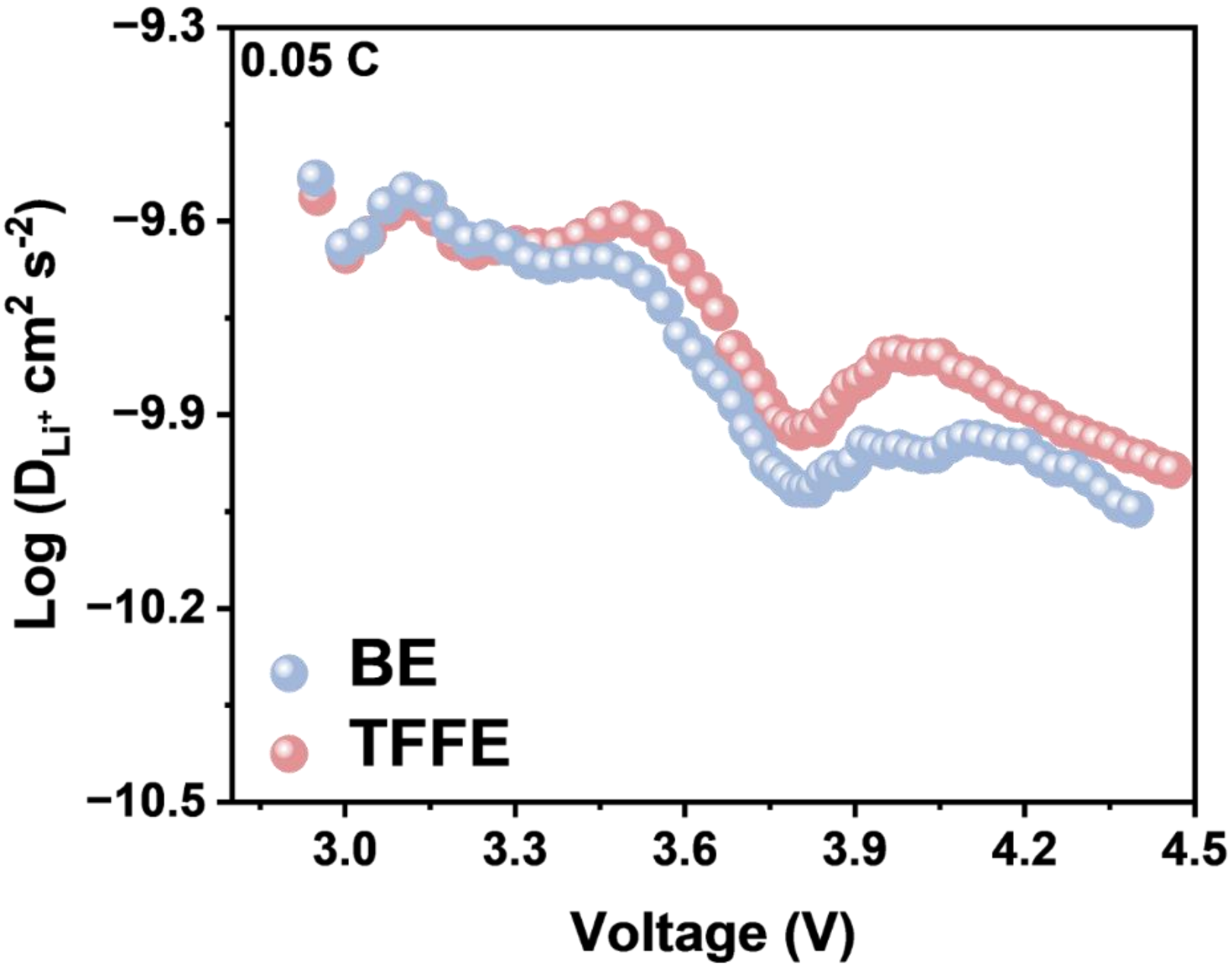

Supplementary Figure 38. The calculated Li^+ diffusion coefficient corresponding to the charge curve at 0.05C after 50 cycles at 1C of Li||LRMO assembled with BE and TFFE.

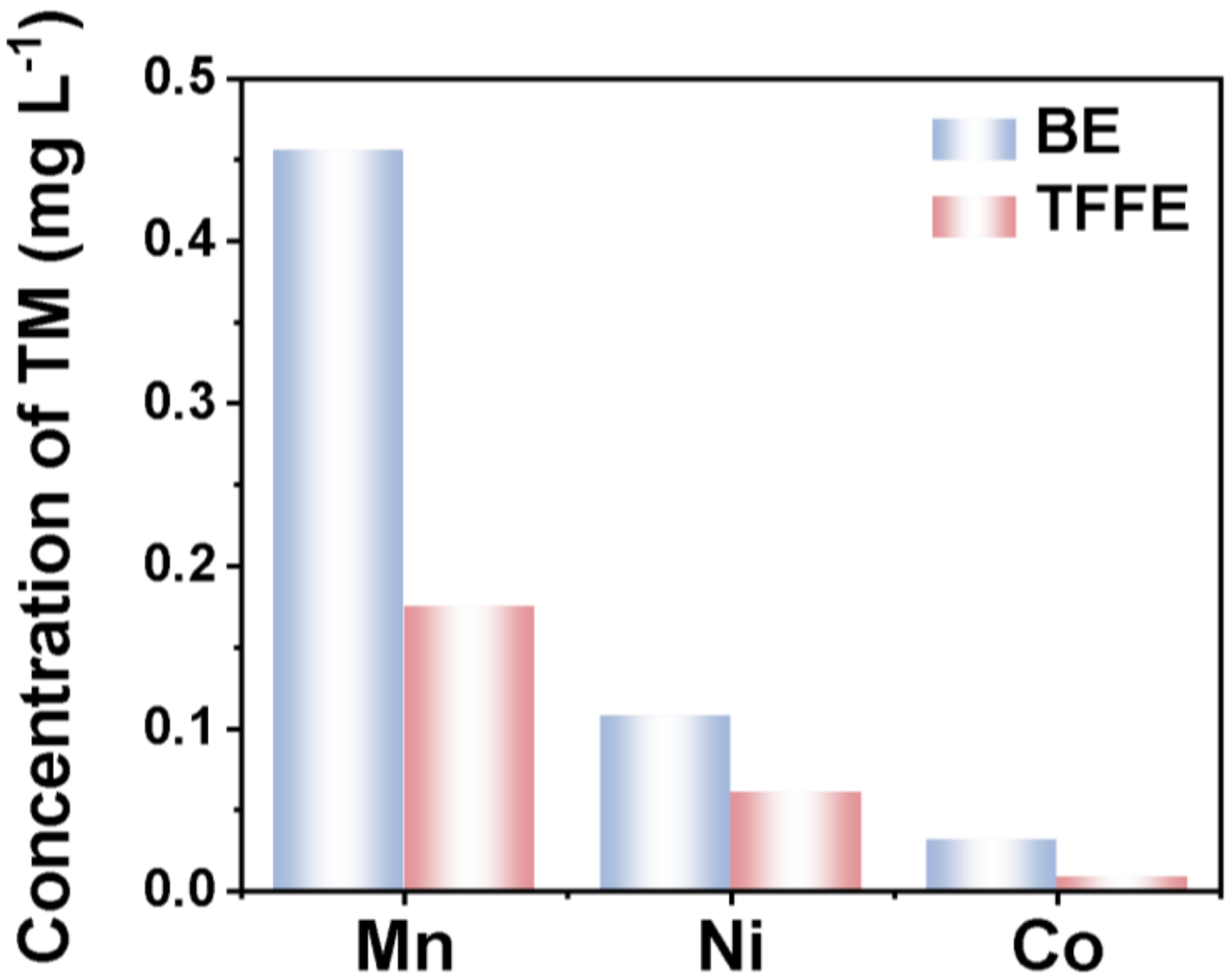

Supplementary Figure 39. Deposition amounts of transition metal (TM) ions (Mn, Co, Ni) on Li negative electrodes after 50 cycles at 1C with different electrolytes.

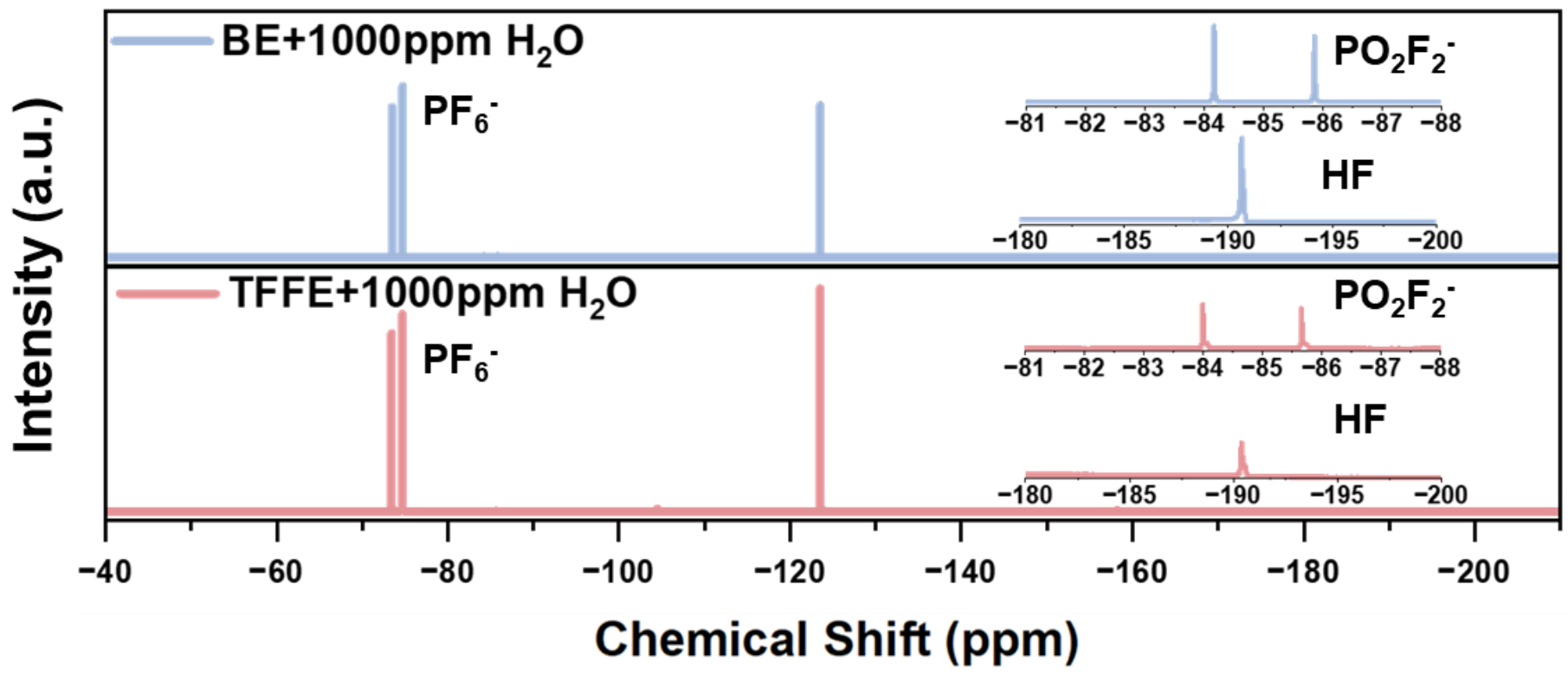

Supplementary Figure 40. ^{19}F NMR spectra of BE and TFFE electrolytes after aging with 1000 ppm H_2O.

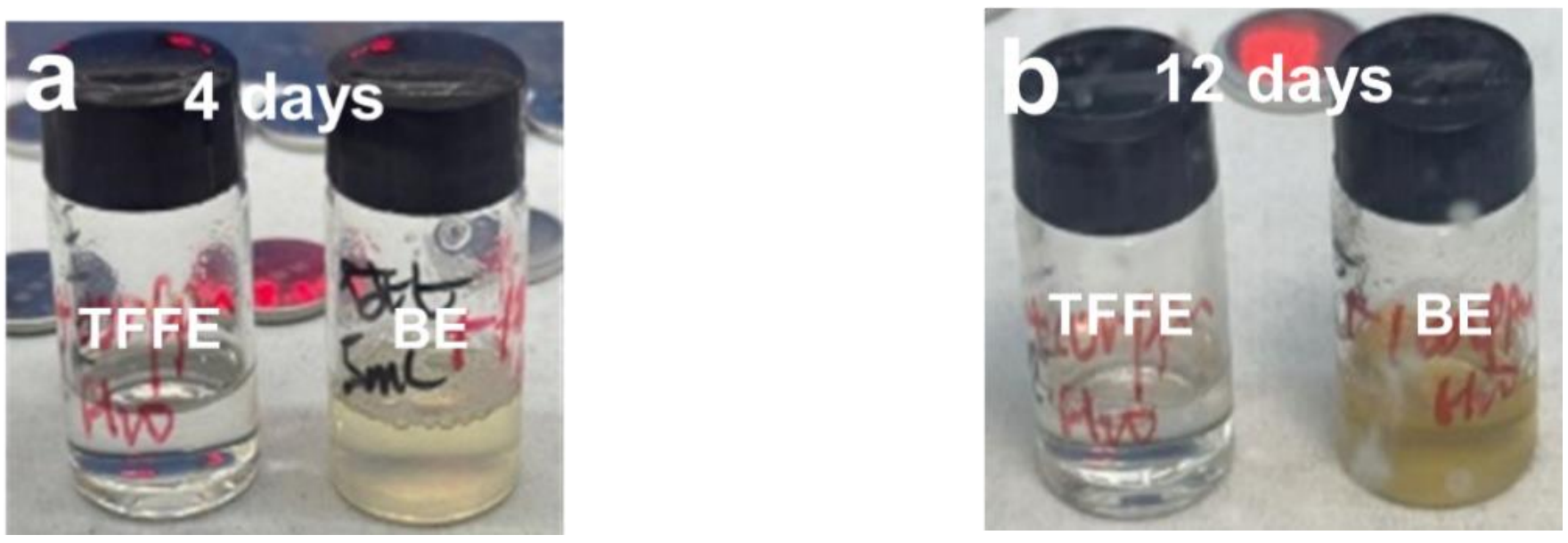

Supplementary Figure 41. Color changes of BE+1000ppm H_2O and TFFE+1000ppm H_2O after standing (a) 4 and (b) 12 days.

`

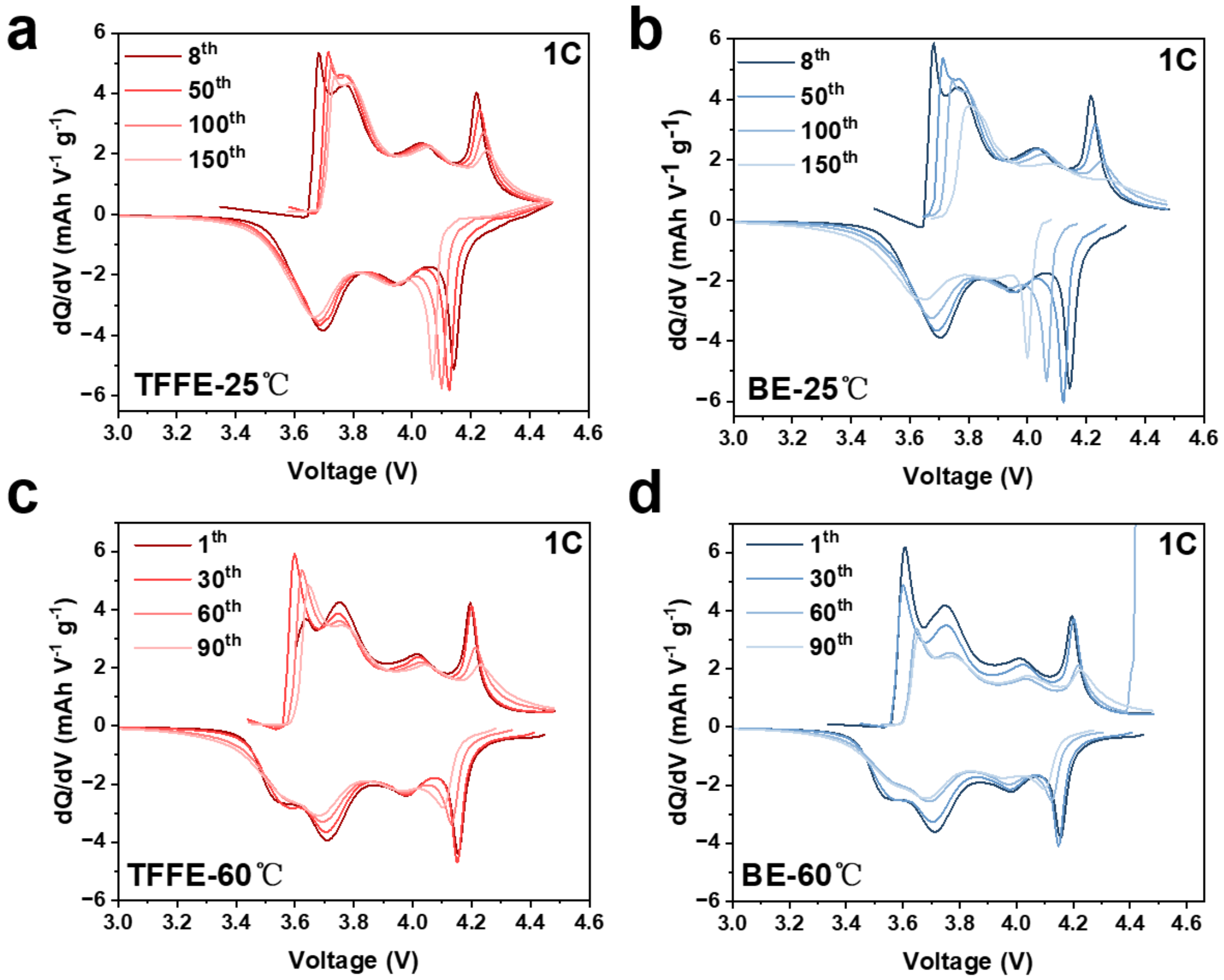

Supplementary Figure 42. dQ/dV-Voltage of Li||NCM811 full cells at 25℃ of (a)TFFE and (b)BE; and 60℃ of (c)TFFE and (d)BE from 2.7V to 4.5V at 1C.

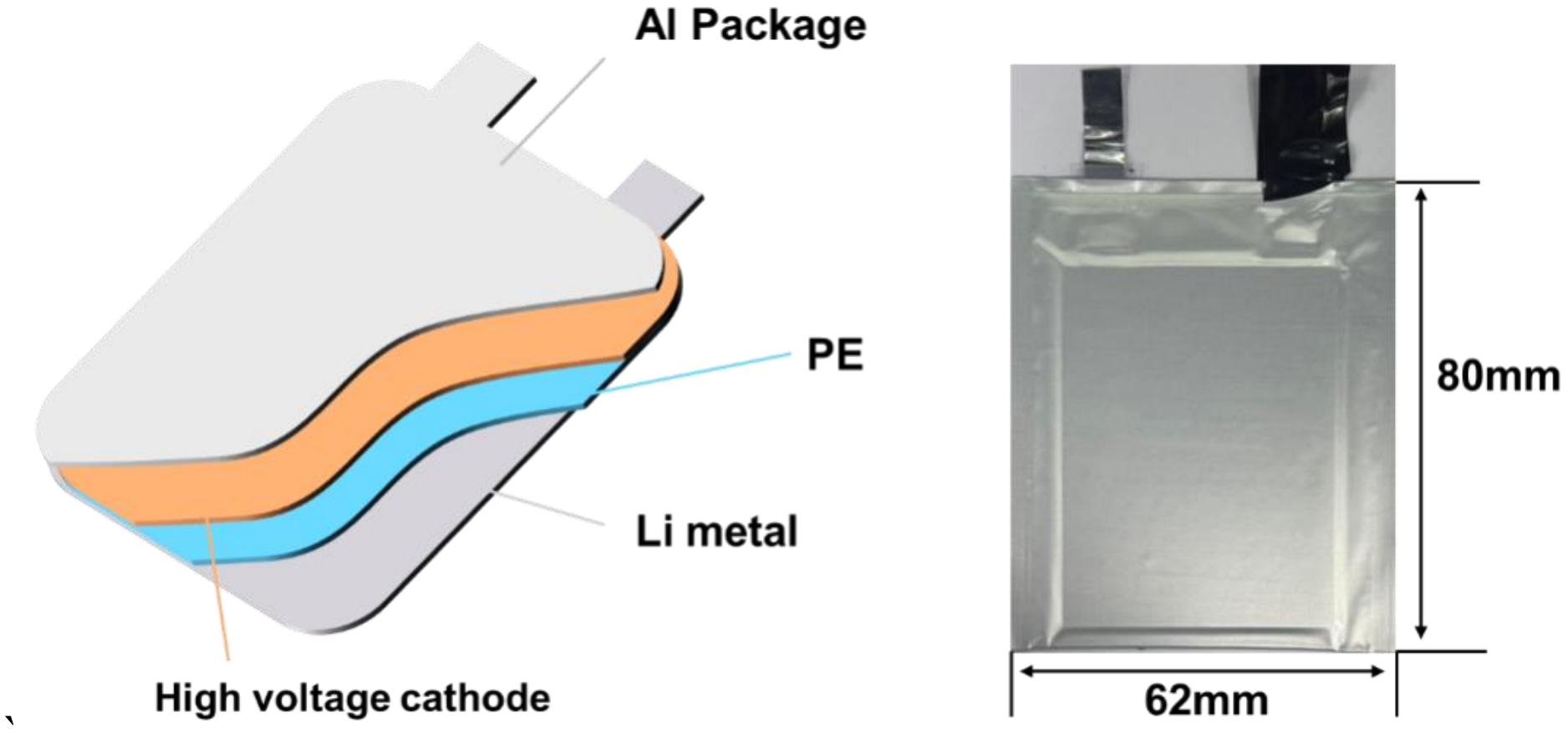

Supplementary Figure 43. Schematic module of pouch cell.

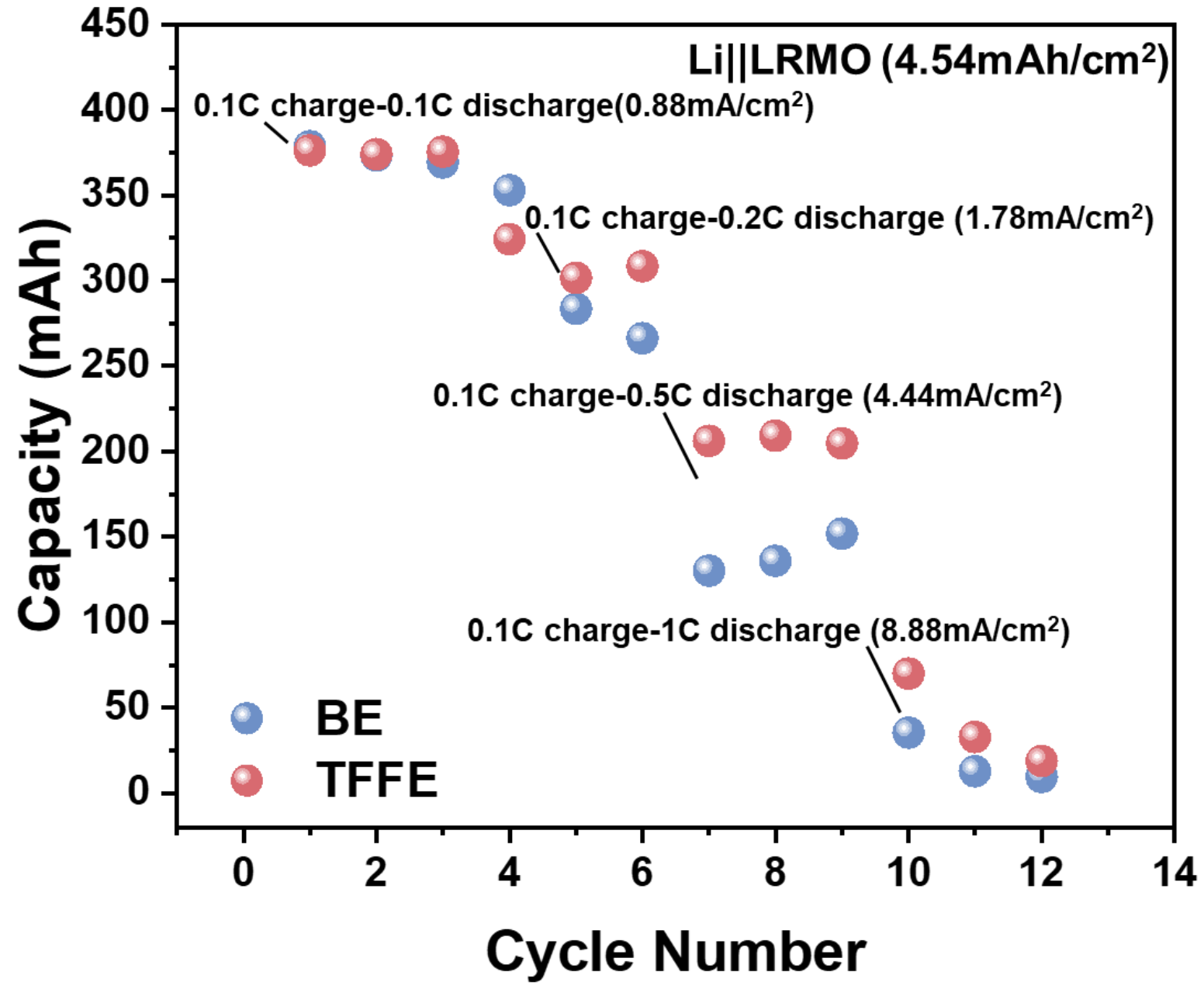

Supplementary Figure 44. Rate capability of Li||LRMO pouch cells assembled with BE and TFFE electrolyte.

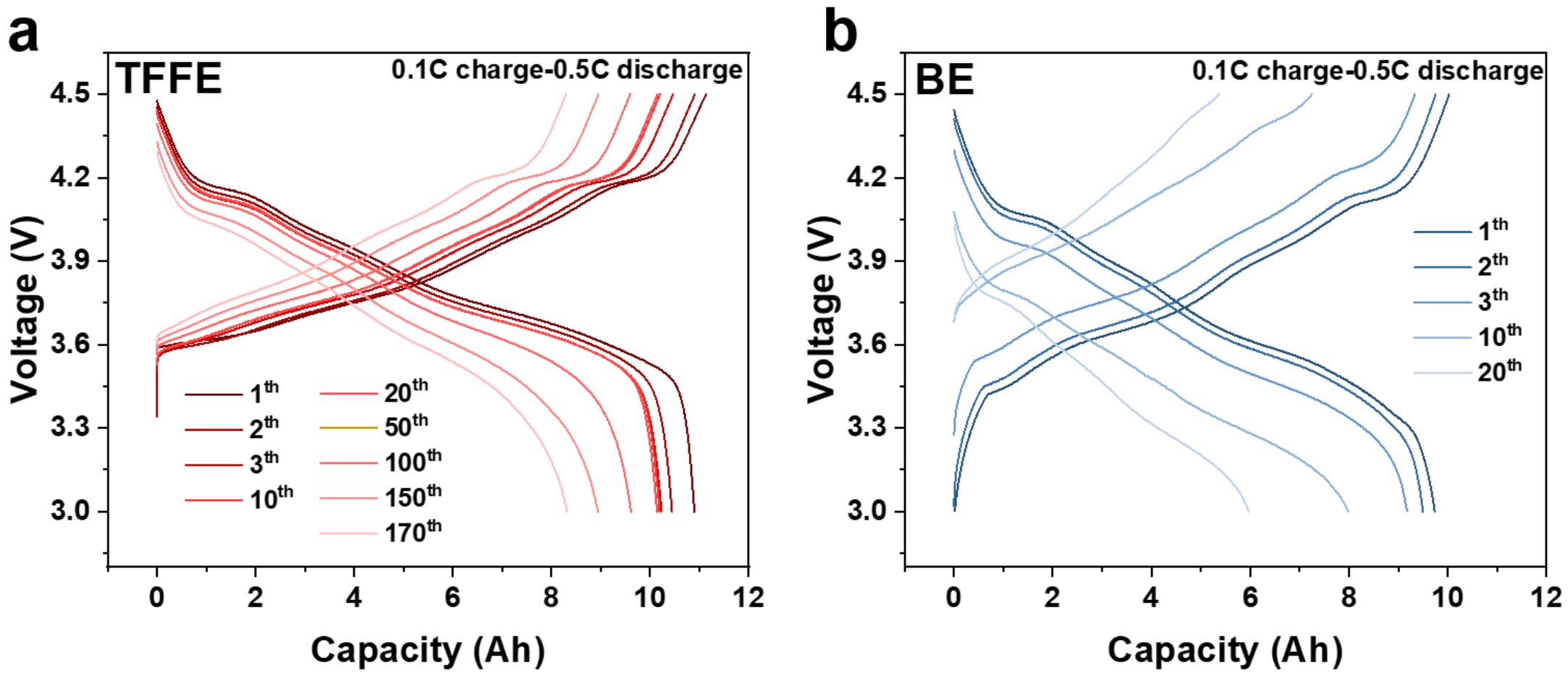

Supplementary Figure 45. Charge/discharge curves of pouch cells assembled with (a)TFFE and (b)BE from 3.0 to 4.5V under different cycles, respectively.

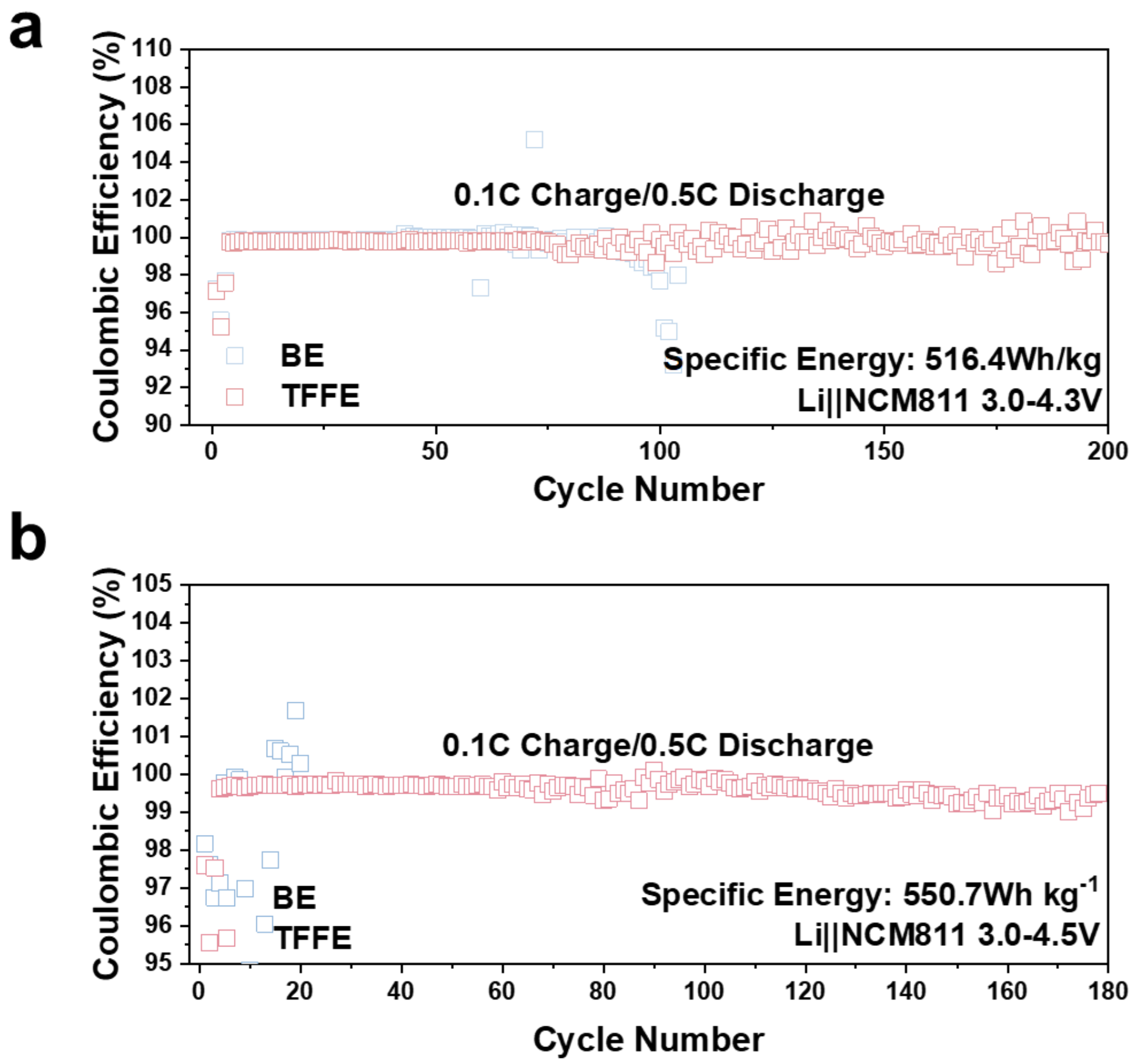

Supplementary Figure 46. The enlarged CE profile of assembled pouch cells. (a)516.4 Wh/kg and (b)550.7 Wh/kg.

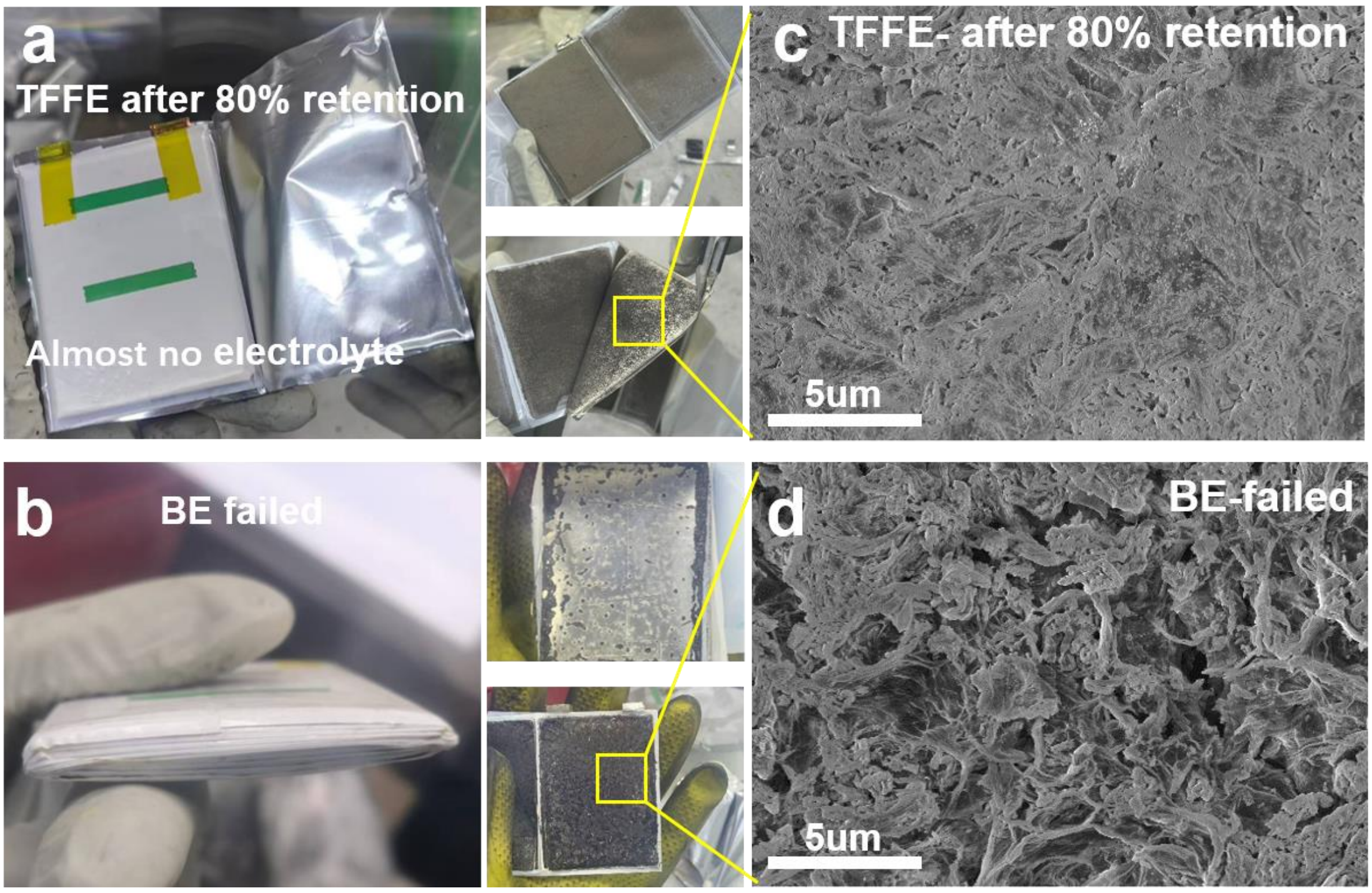

Supplementary Figure 47. Representative image of a Li-metal pouch cell after cycling assembled with (a)TFFE, (b)BE, and the corresponding Li metal SEM images of (c)TFFE and (d)BE system.

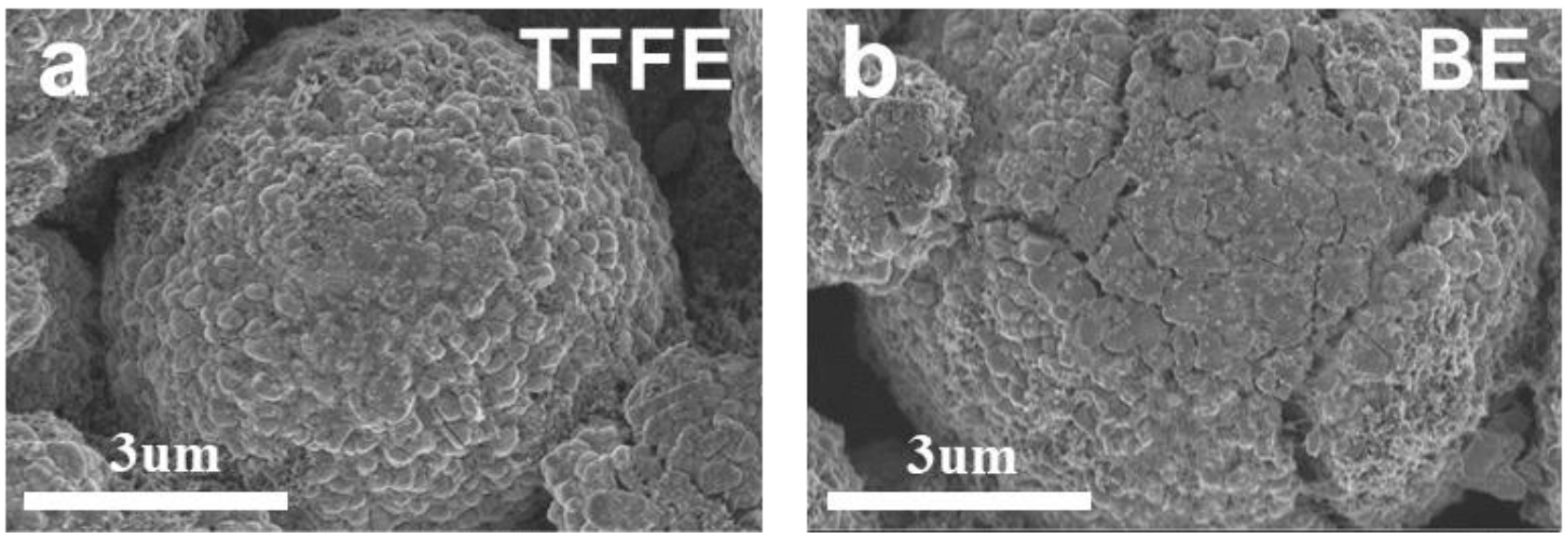

Supplementary Figure 48. SEM images of the positive electrode surface in pouch cells after cycling with (a) TFFE and (b) BE electrolytes.

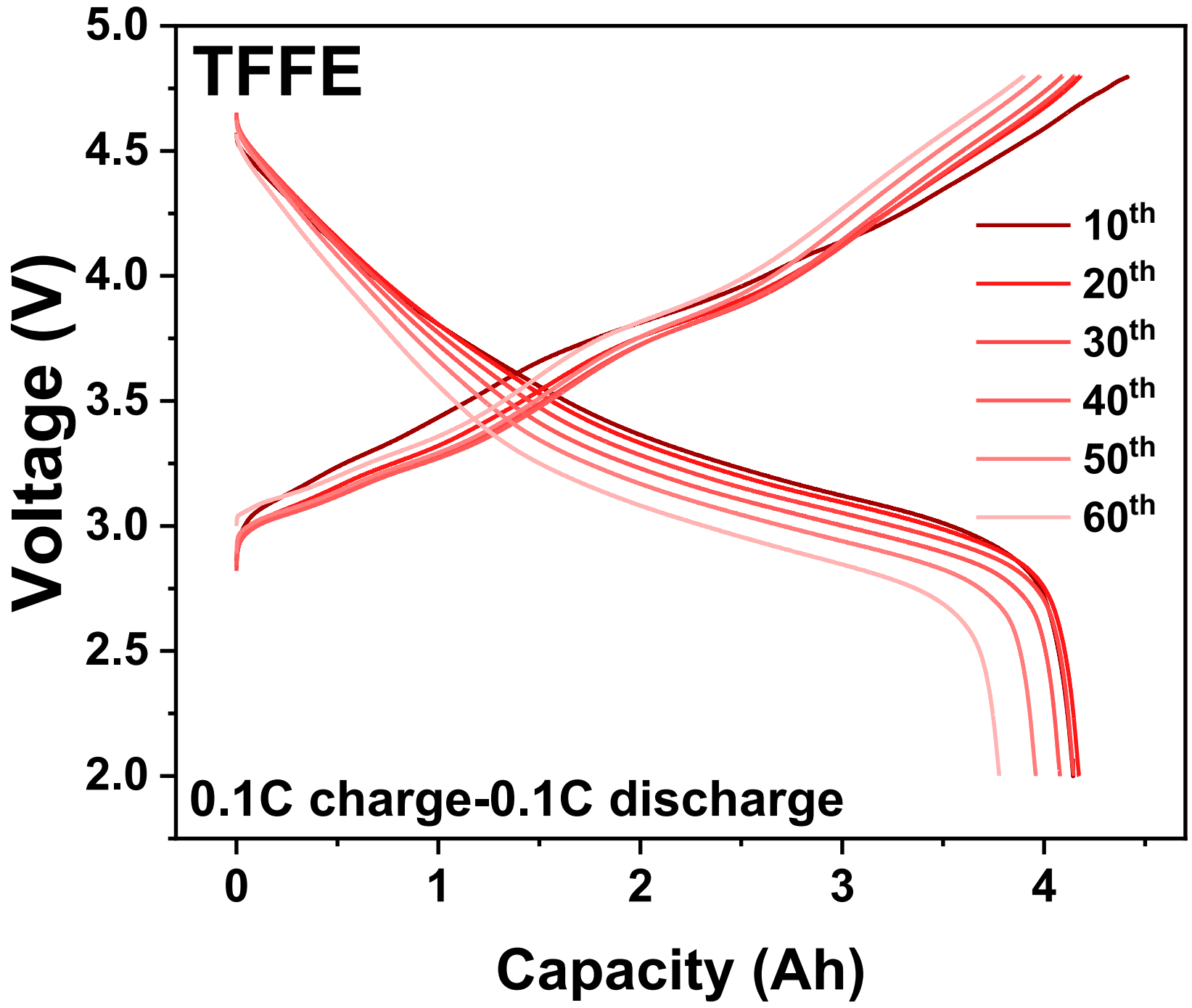

Supplementary Figure 49. Charge/discharge curves of Li||LRMO pouch cell assembled with TFFE from 2.0 V to 4.8 V under different cycles.

Supplementary Table 1. Comparison of coordination numbers of additives reported in the literature.

**Sample**	**Additive content%**	**Coordination number**	**Coordination ratio**	**Ref**
**TMSFS**	**1.0**	**0.194**	**0.99**	**This work**
TMS	1.0	0.034	0.37	This work
FS	1.0	0.002	0.061	This work
o-TFPN	1.0	0.01	0.2	1
TMOBX	1.0	0.005	0.083	2
DSCl	1.0	0.05	0.167	3
TFA^-	1.0	0.022	0.367	4
HN	2.0	0.18	0.49	5
PFPN	~5.0	close to 0	0.06	6

Supplementary Table 2. Equivalent-circuit fitting and detailed analysis on the obtained EIS spectra of TFFE- and BE-based half cells at different temperature.

**Equivalent circuit**	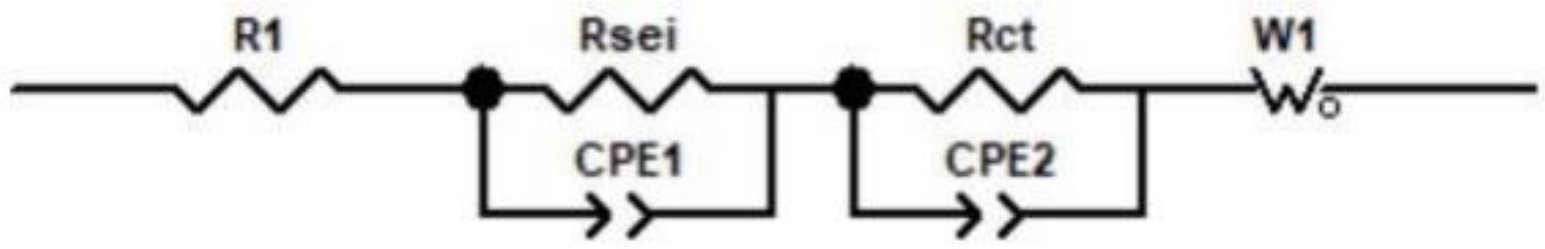		
**Electrodes**	**Temperature (°C)**	**R_{SEI} (ohm) (error%)**	**R_{ct} (ohm) (error%)**
**TFFE**	15	74.57 (1.70%)	181.16 (2.03%)
	25	40.08 (1.85%)	85.77 (1.70%)
	35	23.54 (2.61%)	49.94 (2.76%)
	45	13.29 (2.58%)	24.18 (2.70%)
	55	9.08 (3.95%)	12.22 (3.86%)
**BE**	15	586.61 (1.64%)	636.2 (1.73%)
	25	248.33 (1.86%)	297.7 (1.94%)
	35	136.91 (1.85%)	143.8 (2.06%)
	45	75.31 (1.98%)	79.31 (2.19%)
	55	44.09 (2.82%)	41.46 (2.91%)

Supplementary Table 3. Peak position of XPS fitting results.

Element	Binding Energy (eV)	Assigned to
Li	53.8	Li_2S
	54.7	Li_2O
	55.6	Li_2CO_3/$ROCO_2Li$
	56.2	LiF
	57.0	$Li_xPO_yF_z$
C	289.8	Li_2CO_3
	287.9	$ROCO_2Li$
	284.8	C-C

Supplementary Table 4. The specifications of the NCM811 pouch cell

Cell component	specification	Parameters
Positive electrode (NCM811)	Number	17
	Specific areal capacity (mAh cm^{-2}, each side)	4.2
	Size (cm^2)	7.5*9.5
	Thickness of Al foil (μm)	10
Negative electrode	Number	18
	Size (cm^2)	7.7*9.7
	Thickness of Li foil (μm)	100
N/P	Ratio	4.91
Electrolyte	Injection factor (g Ah^{-1})	1.2
Separator	Thickness (μm)	16
Package and tabs	Weight (g)	2.8
Full cell	Capacity at 4.3V/4.5V (Ah)	10.4/10.9
	Weight (g)	76.2
	Specific energy at 4.3 V (Wh kg^{-1})	516.4
	Specific energy at 4.5 V (Wh kg^{-1})	550.7

The specific energy was calculated based on the first-cycle discharge at 0.1C.

Supplementary Table 5. The specifications of the LRMO pouch cell.

Cell component	specification	Parameters
Positive electrode (LRMO)	Number	10
	Specific areal capacity (mAh cm^{-2}, each side)	5.4
	Size (cm^2)	5.7*7.5
	Thickness of Al foil (μm)	12
Negative electrode	Number	11
	Size (cm^2)	5.9*7.7
	Thickness of Li foil (μm)	50
N/P	Ratio	1.91
Electrolyte	Injection factor (g Ah^{-1})	1.2
Separator	Thickness (μm)	16
Package and tabs	Weight (g)	1.9
Full cell	Capacity (Ah)	4.5
	Weight (g)	26.3
	Specific energy (Wh kg^{-1})	602.5

The specific energy was calculated based on the first-cycle discharge at 0.1C.

Supplementary Table 6. The comparison of pouch cell performance with studies on High Density LMB

Negative electrode	Positive electrode	Specific Energy (Wh kg^{-1})	Capa-city (Ah)	Current density (C)	Cycling performance	Ref
**Li**	NCM811	550.7	10.4	0.1/0.5	capacity retention of 80% after 180 cycles	This work
**Li**	LRMO	602.5	4.5	0.1/0.1	capacity retention of 80% after 60 cycles	This work
**Li**	AZ0.3SNCM	504.5	10.83	0.1/0.3	capacity retention of 73.7% after 50 cycles	9
**Li**	LNMO	505.9	19	0.1/0.1	capacity retention of 91% after 130 cycles	10
**Li**	LRMO	576	9	0.1/0.2	capacity retention of 84.4% after 38 cycles	11
**Li**	NCM811	495.5	5.97	0.1/0.3	capacity retention of 80% after 150 cycles	12
**Li**	NCM811	470	5.2	0.1/0.5	capacity retention of 81% after 100 cycles	13
**Li**	NCM811	503.7	7.3	0.1/0.5	capacity retention of 84.1% after 100 cycles	14
**Li**	NCM83	506.7	7	0.1/0.5	capacity retention of 80% after 160 cycles	15
**Li**	NCM83	521.3	39	0.1/0.1	stably cycle for 20 cycles	16
**Li**	NCM811	547	7.5	0.1/0.2	capacity retention of 79% after 155 cycles	17
**Li**	NCM811	510.3	5.6	0.1/0.25	capacity retention of 84% after 116 cycles	18
**Li**	NCM83	522	7.3	0.1/0.3	capacity retention of 80.9% after 178 cycles	19

**Li**	NCM811	500	6	0.2/0.2	capacity retention of 92% after 150 cycles	20

Reference

1. Li X*, et al.* Cyano-Functionalized Hybrid Electrode-Electrolyte Interphases Enabled by Cyano-Substituted Tetrafluorobenzene Derivatives Additives for High-Voltage Lithium Metal Batteries. *Adv. Funct. Mater.*, 2421329 (2025).
2. Wang W*, et al.* Construction of Ionic Conductive Electrode/ Electrolyte Interphases via Li^+ Coordination Regulator for 4.7 V Li/ $LiNi_{0.9}Co_{0.05}Mn_{0.05}O_2$ Batteries. *Adv. Funct. Mater.*, 2507638 (2025).
3. Lei Y, Xu X, Xu Z, Yin J, Jiang S, Gao Y. Deciphering DSCl-Assisted Solvation and Interfacial Dynamic Evolution for High-Performance Lithium Batteries. *Adv. Energ. Mater.*, 2501096 (2025).
4. Gao Y*, et al.* Transesterification Induced Multifunctional Additives Enable High-Performance Lithium Metal Batteries. *Angewandte Chemie International Edition* **63**, e202403668 (2024).
5. Liu Z*, et al.* Molecule polarity and steric hindrance design to nominate organosilicon additive for high-voltage $LiCoO_2$. *Energy Storage Materials* **77**, 104177 (2025).
6. Liu J, Li X, Huang J, Yang G, Ma J. Additive-guided Solvation-regulated Flame-retardant Electrolyte Enables High-voltage Lithium Metal Batteries with Robust Electrode Electrolyte Interphases. *Adv. Funct. Mater.* **34**, 2312762 (2024).
7. Zhang F, Zhang P, Zhang W, Gonzalez PR, Tan DQ, Ein-Eli Y. Five Volts Lithium Batteries with Advanced Carbonate-Based Electrolytes: A Rational Design via a Trio-Functional Addon Materials. *Adv. Mater.* **36**, 2410277 (2024).
8. Zou Y*, et al.* Non-Flammable Electrolyte Enables High-Voltage and Wide-Temperature Lithium-Ion Batteries with Fast Charging. *Angew. Chem. Int. Ed.* **62**, e202216189 (2023).
9. Ou X*, et al.* Enabling high energy lithium metal batteries via single-crystal Ni-rich cathode material co-doping strategy. *Nat. Commun.* **13**, 2319 (2022).
10. Jie Y*, et al.* Towards long-life 500 Wh kg^{-1} lithium metal pouch cells via compact ion-pair aggregate electrolytes. *Nat. Energy* **9**, 987-998 (2024).
11. Liu X, Li Y, Liu J, Wang H, Zhuang X, Ma J. 570 Wh kg^{-1}-Grade Lithium Metal Pouch Cell with 4.9V Highly Li^+ Conductive Armor-Like Cathode Electrolyte Interphase via Partially Fluorinated Electrolyte Engineering. *Adv. Mater.* **36**, 2401505 (2024).
12. Wu L-Q*, et al.* Regulating Amine Substitution in Fluorosulfonyl-Based Flame-Retardant Electrolytes for Energy-Dense Lithium Metal Batteries. *J. Am. Chem. Soc.* **147**, 16506-16521 (2025).
13. Yang W*, et al.* A Bipolar Solvent Molecule Design for Wide-Temperature High-Voltage Lithium Metal Batteries. *Adv. Mater.* , 2505285 (2025).
14. Qiao R*, et al.* Non-fluorinated electrolytes with micelle-like solvation for ultra-high-energy-density lithium metal batteries. *Chem* **11**, 102306 (2025).
15. Chen Q*, et al.* Macroscopically uniform interface layer with Li^+ conductive channels for high-performance Li metal batteries. *Nat. Commun.* **15**, 10045 (2024).

16. Su H*, et al.* Achieving Practical High-Energy-Density Lithium-Metal Batteries by a Dual-Anion Regulated Electrolyte. *Adv. Mater.* **35**, 2301171 (2023).
17. Ji H*, et al.* Liquid–liquid interfacial tension stabilized Li-metal batteries. *Nature* **643**, 1255–1262 (2025).
18. Yang W, Chen A, He P, Zhou H. Advancing lithium metal electrode beyond 99.9% coulombic efficiency via super-saturated electrolyte with compressed solvation structure. *Nat. Commun.* **16**, 4229 (2025).
19. Tang T, Sun C, Li Y, Tong M, Lu J, Lai C. Long-Lifespan 522 Wh kg^{-1} Lithium Metal Pouch Cell Enabled by Compound Additives Engineering. *Angewandte Chemie International Edition* **64**, e202417471 (2025).
20. Zhang S*, et al.* Oscillatory solvation chemistry for a 500 Wh kg^{-1} Li-metal pouch cell. *Nat. Energy* **9**, 1285-1296 (2024).
