# Supplementary material for: Additive strongly-coordinated solvation structure towards high-voltage 600 Wh/kg-class lithium metal pouch cell
Source: Nat Commun. 2026 Aug 24;17:10119. doi: 10.1038/s41467-026-77095-x (PMC13597530; doi:10.1038/s41467-026-77095-x)
Supplement: Supplementary file 2 [file 41467_2026_77095_MOESM2_ESM.pdf]

Description of Additional Supplementary Files

File Name: Supplementary Data 1
Description: The XYZ coordinates of all models in the DFT.

File Name: Supplementary Data 2
Description: The XYZ coordinates of all models in MD theoretical calculation.
