# Supplementary material for: Additive strongly-coordinated solvation structure towards high-voltage 600 Wh/kg-class lithium metal pouch cell
Source: Nat Commun. 2026 Aug 24;17:10119. doi: 10.1038/s41467-026-77095-x (PMC13597530; doi:10.1038/s41467-026-77095-x)
Supplement: Supplementary file 3 [file 41467_2026_77095_MOESM5_ESM.pdf]

nature portfolio

Peer Review File

Additive Strongly-Coordinated Solvation Structure towards High-Voltage 600Wh/kg-class Lithium Metal pouch cell

Corresponding Author: Professor Yongji Gong

This file contains all reviewer reports in order by version, followed by all author rebuttals in order by version.

Version 0:

Reviewer comments:

Reviewer #1

(Remarks to the Author)
The authors proposed an additive-coordinated-solvation electrolyte using the trimethylsilyl-2,2-difluoro-2-(fluorosulfonyl) acetate (TMSFS) additive, which enables stable cycling of a 550.7 Wh kg^{-1} Li||NCM811 pouch cell over 180 cycles and a 600 Wh kg^{-1} Li||LRMO pouch cell over 60 cycles. The demonstrated high-energy and cycling performance of the Li metal pouch cells is noteworthy. Therefore, the manuscript can be considered for publication on Nature Communications after the following questions are addressed:

1. Does the energy density calculation take into account the mass of the entire cell (including packaging and tabs)? If so, could the authors clearly state this in the main text?
2. The Li metal CE of the designed electrolyte is only 97.8%, yet the cycling performance of the pouch cells is impressive. How does the author understand the relationship between electrolyte CE, battery energy density, and cycle life?
3. DFT calculations and MD simulations show that even a small amount of TMSFS can more easily enter the first solvation shell of Li^+. However, the experimental results from Raman or NMR suggest that the effect of TMSFS on the electrolyte solvation structure is not very pronounced. Could the authors use additional characterization methods to demonstrate the additive strongly coordinated solvation structure? Alternatively, the limited observed effect on solvation structure maybe due to the low additive content, and if so, could higher additive concentrations be tested to examine the trend in solvation structure changes?
4. According to the authors' description, TMSFS interacts more strongly with Li^+ compared to the anion and solvents. Could the authors clarify whether $LiPF_6$ can dissolve in TMSFS alone?

Other minor issues:
5. Figure 2: Could the authors provide the Raman spectra of pure FEC and EMC solvents in the main text or Supplementary Materials for reference?
6. Page 7, Line 164: Typo errors in the chemical formula.
7. Figure S11: What is the reason for the sudden change in the overpotential of the symmetrical cell during the first 20 cycles?
8. The rate information should be provided in Figure S19.
9. The dQ/dV-Voltage profiles in Figure S20 are difficult to interpret in terms of phase transitions and capacity decay; could the authors label these features more clearly in the figure?

Reviewer #2

(Remarks to the Author)
This work describes an additive coordinated solvation structure-based strategy to enhance the stability and lifespan of high-voltage lithium metal batteries (LMBs). A newly designed TMSFS molecule with electron push–pull effects coordinates strongly with Li^+, replacing solvent and anion species in the inner solvation shell. This mitigates excessive solvent/anion consumption and promotes the formation of inorganic-rich SEI/CEI layers (Li_2S, LiF) for interfacial stability. As a result, Li/NCM811 pouch cells achieved 550.7 Wh/kg with 80% retention over 180 cycles, while Li/|LRMO pouch cells reached 602.5 Wh/kg with 80% retention for 60 cycles. These findings demonstrate that even a low concentration (1 vol%) additive can significantly improve the practical performance of high-energy-density LMBs. Although the suggested results are

interesting, there are several points that should be revised in the revision process.
1. A deeper discussion on the effect of molecular structure on its electrochemical behaviors should be attempted. For instance, the authors utilized acetate functional groups, which have been well-recognized for facilitating electrochemical oxidation due to their presence. In this case, how can the electrochemical oxidation reaction be controlled in the presence of such a weak functional group? More specifying the molecular structure of TMSFS seems to be necessary.
2. Although there are electron-withdrawing groups (F) in the TMSFS, the authors mentioned that the other electron-donating group can participate in increasing Li+ coordination; it is somewhat ambiguous. There are different functional groups in the TMSFS; which functional group mainly contributes to enhancing the Li+ coordination, and why? A deeper discussion is also necessary in this section. Another word, further discussion on the TMSFS, including its role within the functional group, should be provided.
3. In Figure 1, the conceptual mechanism of the TMSFS was suggested; however, it is difficult to understand the pull-push mechanism. The logic shown in Fig. 1d should be validated or clarified with more scientific discussion.
4. Although the electrochemical performances seem to be improved, dependence on the TMSFS amounts should be suggested in the main manuscript.
5. Voltage profiles were not suggested, even if they provide important information to understand the effect of TMSFS. The authors should include it.
6. One of the ambiguous points is whether Li2S is a good component for a stable SEI layer on the Li anode? Is it a good ionic conductor?
7. Lack of basic properties depending on the amount of TMSFS should be provided.

Reviewer #3

(Remarks to the Author)
The authors have systematically investigated a new electrolyte design—an additive-coordinated solvation structure (ACSS) of Li^+—which preferentially sacrifices the additive rather than anions to enable stable cycling of 600 Wh kg^{-1} lithium metal batteries (LMBs).
They discovered that by leveraging the electron push–pull effect among functional groups within the trimethylsilyl-2,2-difluoro-2-(fluorosulfonyl) acetate (TMSFS) molecule, even trace additive levels allow TMSFS to coordinate within the inner sheath of the Li^+ solvation structure, reducing the participation of ester solvent molecules and anions. This suppresses excessive anion and solvent consumption, while facilitating the formation of robust protective interphases on both cathode and anode surfaces, thereby enhancing high-voltage stability and Li-metal compatibility.
Overall, this work provides valuable insight into electrolyte design strategies for high-Ni cathodes and high-energy-density LMBs. After some minor revisions, I would recommend this manuscript for publication in Nature Communications.
Comments and Suggestions
1. As the authors described, uniformly distributed Li nuclei with compact and homogeneous morphology were observed in the TFFE system. Typically, a moderate CEI thickness (5–10 nm) offers sufficient chemical and mechanical protection for high-voltage systems. It is therefore interesting that the TFFE system exhibits a CEI thickness of only 2.7 nm, yet provides effective coverage without leading to surface cracking or continuous side reactions. The authors are encouraged to provide additional characterization or discussion to clarify the stability mechanism of such a thin CEI.
2. The 0.1 C energy densities reported for the Li-metal pouch cells are recommended to be revised to cycled energy densities, as this would more accurately reflect practical performance.
3. Please indicate the rate in Supplementary Figure 19. It is particularly interesting that the TFFE and BE systems only diverge at the highest rate. Why are the pouch cells not tested under the same rate and voltage window as the coin cells? A brief explanation or justification would improve the clarity of the data comparison.

Reviewer #4

(Remarks to the Author)
This paper investigates the role of a designed additive in lithium metal batteries. The central rationale lies in that the electron-donating functional group (trimethylsiloxy) enhances the coordination between lithium and the additive, thereby forming a stable six-membered ring structure that facilitates the formation of robust SEI and CEI layers. The designed electrolyte demonstrates relatively good performance in lithium metal batteries.
However, the paper suffers from two major issues. First, the novelty of the design strategy is insufficient — the concept of tuning electron-withdrawing or electron-donating functional groups is rather common and fails to meet the journal's standard for innovation. Second, the scientific rationale behind using trace additives to improve the stability of lithium metal batteries remains questionable. The core issue is that the SEI on lithium metal surfaces is renewed during each plating/stripping cycle, meaning that reactive trace additives have limited influence throughout the lithium lifecycle. This viewpoint has also been supported by previous work from Prof. J. R. Dahn (A. Eldesoky et al., J. Electrochem. Soc. 168, 120508 (2021)). Below are my suggestions for the manuscript.

1. The primary reason why lithium metal pouch cells cannot achieve long cycling performance comparable to that of lithium-ion batteries lies in the rapid depletion of the electrolyte. This depletion mainly originates from the continuous renewal of the SEI layer on the lithium surface and the (electro)chemical corrosion of lithium. Although TMSFS–Li^+ possesses a low LUMO energy level that facilitates its decomposition on the lithium metal surface, its limited concentration means it is rapidly consumed during cycling and thus cannot play a decisive role throughout the battery's lifetime. Therefore, the low LUMO energy level should not be regarded as an advantage of this additive. I suggest that the authors shift the focus of the paper

toward the formation of the cathode–electrolyte interphase (CEI), or alternatively, increase the TMSFS content instead of treating it merely as a trace additive.

2. The amount of additive should be consistently defined as either a mass fraction or a volume fraction. In Fig. 2e and the Methods section, it is described as a mass fraction, whereas in the main text it is presented as a volume fraction. In addition, the authors should clarify the rationale for selecting this particular concentration of additive or provide an optimization study of the additive content.

3. In your calculations, the binding energy between Li and EMC is more negative (compared to that between Li and FEC). However, in the MD simulations, after introducing TMSFS, it primarily replaces EMC rather than FEC. This result is puzzling. In addition, it remains unclear why the coordination number of PF_6^- is significantly reduced upon the trace addition of TMSFS.

4. In the MD simulations, the total coordination number of Li is typically around 4.5 (and rarely below 4), which is determined by the van der Waals radius of lithium. However, in the electrolyte containing TMSFS, the reported total coordination number is only 3.5, which appears problematic. Please verify whether the simulation box volume and system density are reasonable. Moreover, in the RDF results, the peak intensity of Li–TMSFS is 4.5 times that of Li–FEC. Considering that each TMSFS molecule contributes two coordinating oxygen atoms, while each FEC molecule contributes one (both containing three oxygens in total), and that the number of FEC molecules is 13 times that of TMSFS, the coordination number of FEC should theoretically be **13 / (4.5 / 2) = 5.77** times that of TMSFS. However, the authors report only a 2.55-fold difference. Please re-examine the calculation procedure.

5. Please indicate the electrolyte injection amount in the figure, so that readers can better understand the performance level of the cells reported by the authors. Please check the presentation of units throughout the manuscript. For example, in expressions such as “1 M,” there should be a space between the number and the unit (“1 M,” not “1M”).

Version 1:

Reviewer comments:

Reviewer #1

(Remarks to the Author)
The authors have satisfactorily addressed all the concerns and comments raised in my previous review. I have no further comments at this stage and recommend the manuscript for publication.

Reviewer #2

(Remarks to the Author)
Most of questions are revised, therefore I recommend this draft to be published in this journal.

Reviewer #4

(Remarks to the Author)
The authors have provided a comprehensive and generally satisfactory response. However, I would like to raise two additional comments:

1. Concerning the interpretation of self-diffusion coefficients in relation to coordination ratios.
There appears to be an issue in using self-diffusion coefficients to quantitatively infer coordination proportions. The authors did not clearly specify how the coordination ratio was calculated. It should be noted that upon salt addition, the viscosity of the system typically increases. As a result, the diffusion coefficients of all species decrease regardless of whether they participate in coordination. Therefore, the observed reduction in diffusion coefficients can at most provide qualitative evidence for changes in coordination behavior, rather than a quantitative determination of coordination ratios. A more rigorous analysis or complementary structural evidence would be necessary to substantiate such claims.

2. Regarding the choice of force field in MD simulations.
Although polarizable force fields can, in principle, improve the physical realism of charge distributions, they do not necessarily offer clear advantages over conventional force fields in terms of applicability range, methodological complexity, accessible system size, or computational cost. For current electrolyte systems, well-established and validated simulation protocols already exist and have demonstrated reliable accuracy in predicting monovalent ion coordination numbers, system density, viscosity, and related physicochemical properties. In this regard, I would recommend the use of the OPLS-AA/L force field in combination with 1.14CM1A-LBCC charges, with appropriate charge scaling applied when necessary. Nevertheless, it should also be acknowledged that each methodology has its own limitations, and based on the results presented, the force field currently employed by the authors appears to be acceptable.

Version 2:

Reviewer comments:

Reviewer #4

(Remarks to the Author)
The authors have adequately addressed all the issues and comments raised in my previous review. I have no further remarks at this stage and recommend the manuscript for publication.

Open Access This Peer Review File is licensed under a Creative Commons Attribution 4.0 International License, which permits use, sharing, adaptation, distribution and reproduction in any medium or format, as long as you give appropriate credit to the original author(s) and the source, provide a link to the Creative Commons license, and indicate if changes were made.
In cases where reviewers are anonymous, credit should be given to 'Anonymous Referee' and the source.
The images or other third party material in this Peer Review File are included in the article’s Creative Commons license, unless indicated otherwise in a credit line to the material. If material is not included in the article’s Creative Commons license and your intended use is not permitted by statutory regulation or exceeds the permitted use, you will need to obtain permission directly from the copyright holder.
To view a copy of this license, visit https://creativecommons.org/licenses/by/4.0/

We thank the referees for the helpful and positive comments and have revised the manuscript accordingly. In this point-to-point response letter, comments from the referees are in black typeface, and our responses are in the blue typeface. Major changes have been highlighted in the revised manuscript.

Reviewer: 1

Comments:

The authors proposed an additive-coordinated-solvation electrolyte using the trimethylsilyl-2,2-difluoro-2-(fluorosulfonyl) acetate (TMSFS) additive, which enables stable cycling of a 550.7 Wh kg^{-1} Li||NCM811 pouch cell over 180 cycles and a 600 Wh kg^{-1} Li||LRMO pouch cell over 60 cycles. The demonstrated high-energy and cycling performance of the Li metal pouch cells is noteworthy. Therefore, the manuscript can be considered for publication on Nature Communications after the following questions are addressed:

Question regarding details not sufficiently explained in the text:

1. Does the energy density calculation take into account the mass of the entire cell (including packaging and tabs)? If so, could the authors clearly state this in the main text?

Response: Thanks for the reviewer's emphasizing the importance of the mass of the entire cell. The mass of the entire cell (including packaging and tabs) is taken into account of our pouch cells energy density calculation. We have supplemented the details of the mass of the entire cell and state it in the main text (Table R1 and R2).

Table R1. The specifications of the NCM811 pouch cell

Cell component	specification	Parameters
	Number	17
Cathode	Specific areal capacity (mAh cm^{-2}, each side)	4.2
(NCM811)	Size (cm^2)	7.5*9.5
	Thickness of Al foil (μm)	10
	Number	18
Anode	Size (cm^2)	7.7*9.7
	Thickness of Li foil (μm)	100
Electrolyte	Injection factor (g Ah^{-1})	1.2

Separator	Thickness (μm)	16
Package and tabs	Weight (g)	2.8
Full cell	Capacity at 4.3V/4.5V (Ah)	10.4/10.9
	Weight (g)	76.2
	Energy density at 4.3 V (Wh kg^{-1})	516.4
	Energy density at 4.5 V (Wh kg^{-1})	550.7

Table R2. The specifications of the LRMO pouch cell.

Cell component	specification	Parameters
Cathode (LRMO)	Number	10
	Specific areal capacity (mAh cm^{-2}, each side)	5.4
	Size (cm^2)	5.7*7.5
	Thickness of Al foil (μm)	12
Anode	Number	11
	Size (cm^2)	5.9*7.7
	Thickness of Li foil (μm)	50
Electrolyte	Injection factor (g Ah^{-1})	1.2
Separator	Thickness (μm)	16
Package and tabs	Weight (g)	1.9
Full cell	Capacity (Ah)	4.5
	Weight (g)	26.3
	Energy density (Wh kg^{-1})	602.5

The changes of the above aspects have been supplemented in the revised manuscript highlighted with yellow, which can also be found below:

All energy density values were calculated based on the total cell mass, including packaging materials and tabs, with an electrolyte injection amount of 1.2 g Ah^{-1}. And pouch cells were evaluated at different discharge rates, from which a moderate rate (<0.5C) was identified as the most suitable testing condition for reliable performance assessment.

Table R1 to R2 were applied as Supplementary Table 4 and Supplementary Table 5

2. The Li metal CE of the designed electrolyte is only 97.8%, yet the cycling performance of the pouch cells is impressive. How does the author understand the relationship between electrolyte CE, battery energy density, and cycle life?

Response: Thanks for the reviewer's suggestion. Firstly, the Li||Cu half cells were

carried at current density of 0.5 mA cm^{-2} with a fixed areal capacity of 2 mAh cm^{-2}, cycled for 400h and the calculated CE 97.8%, indicated only 0.022% loss of active lithium per cycle, which is sufficient for long-term cycling considering the lithium in pouch cell is excessive. Moreover, we have diligently compiled CE of several ester-based electrolytes from published papers and presented them in a table with appropriate citations (Table R3) which demonstrates a common relatively low CE of ester-based electrolytes and this work showed much enhanced CE compared to the base electrolyte.

Table R3. The comparison of average CE of several ester-based electrolytes.

Electrolyte Formulation	Current Density	Average CE	Ref.
EMC: FEC=4:1+1M $LiPF_6$+1% TMSFS	0.5mA/cm^2-2mAh/cm^2	97.8%	This work
EMC: FEC=4:1+1M $LiPF_6$	0.5mA/cm^2-2mAh/cm^2	short circuit	This work
(1) DMDOHD+1 M $LiPF_6$<br>(2) DMDOHD: FEC=5:1+1 M $LiPF_6$	0.5mA/cm^2-1mAh/cm^2	(1) 92%<br>(2) 96.41%	1
EC: DEC=1:1+1M $LiPF_6$+5% $Cu(NO_3)_2$	0.4mA/cm^2-0.8mAh/cm^2	97.32%	2
ETMA: FEC=9:1+1 M LiFSI	1mA/cm^2-1mAh/cm^2	95%	3
DMC: FEC=1:1+1 M $LiPF_6$	0.3mA/cm^2-3mAh/cm^2	97.1%	4
EC: DEC= 1:1+1 M $LiPF_6$+15 wt% FEC	0.5mA/cm^2-1mAh/cm^2	97.7%	5
EC:DMC=1：1+1 M $LiPF_6$	0.5mA/cm^2-1mAh/cm^2	97.61%	6

As for Li metal pouch-cell testing, unlike anode-free configurations, the lithium foil serves as an additional reservoir of active lithium loss.[1]Furthermore, to enhance the energy density, the electrolyte filling in pouch cells must be reduced (typically below

1.5 g Ah^{-1}). Under such restricted electrolyte conditions, greater demands are placed on the minimized loss of electrolyte components during cycling and the failure is typically attributed to the depletion of the lithium salt and solvent.[2-6] Post-mortem examination of the pouch cells assembled with TFFE after cycling reveals that the electrolyte had been almost completely depleted and the Li metal preserved a largely intact morphology (Fig. R1a and c), while the severe lithium dendrite was detected in the BE counterpart (Fig. R1b and d). This observation indicates that cell failure primarily originated from electrolyte exhaustion rather than the loss of active lithium.

Regarding the relationship between electrolyte CE, battery energy density, and cycle life, it is necessary to consider the two main failure modes of lithium metal batteries. Batteries with active lithium inventory depletion as their failure mode exhibit low electrolyte CE (< 95%), typically manifesting as a pronounced capacity drop during battery cycling. Under this failure mode, achieving a higher electrolyte CE generally correlates with extended cycle life. And increasing the amount of electrolyte can enhance the cycle life but reduce energy density. Another battery failure mode is dominated by the depletion of lithium salts in the electrolyte. Batteries exhibiting this failure mode typically demonstrate high electrolyte CE associated with the formation of a more stable, lithium salt-derived inorganic-rich SEI. Under this condition, increasing both electrolyte volume and Li salt concentration enhances the cycle life—at the expense of reduced energy density of battery.

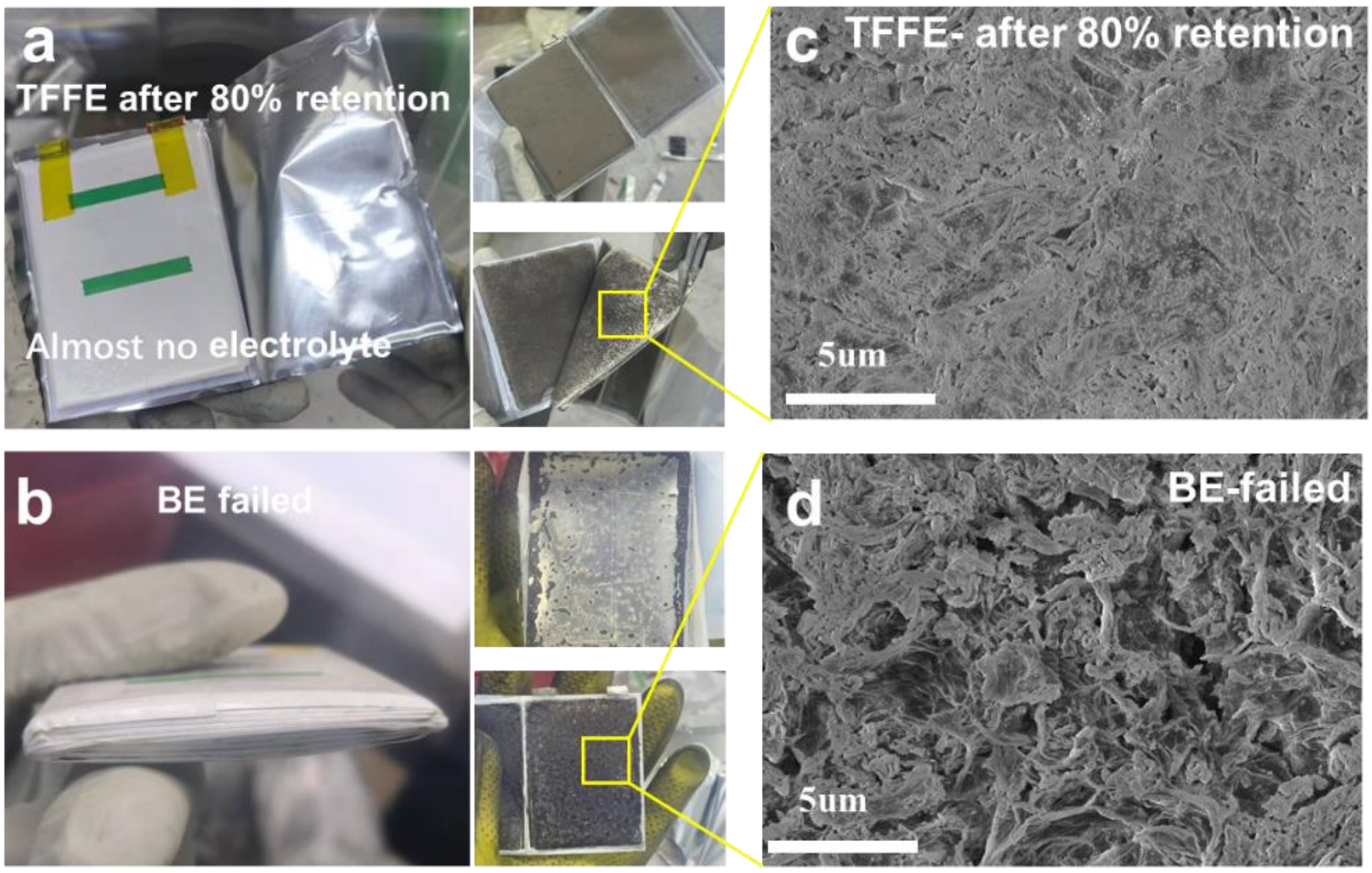

Fig. R1 Representative image of a Li-metal pouch cell after cycling assembled with (a)TFFE, (b)BE, and the corresponding SEM images of (c)TFFE and (d)BE system.

In the revised manuscript, we have provided more discussion to substantiate the role of TMSFS in enhancing the CE of the electrolyte. The changes of the above aspects have been supplemented in the revised manuscript highlighted with yellow, which can also be found below:

As shown in Supplementary Fig. S44, TFFE-based pouch cells exhibit consistently high CE (>98%) throughout cycling, indicating interface-stable failure dominated by electrolyte depletion, whereas the control cells show simultaneous CE and capacity collapse, characteristic of Li anode degradation, leading to premature failure. After disassembling the pouch cell, SEM image of the Li metal anode in TFFE-based pouch cell exhibited compact and homogeneous morphology without visible dendritic features (Supplementary Fig. S45a), while the Li metal anode in BE-based cell displayed mossy and irregular Li deposits under identical cycling conditions (Supplementary Fig. S45b). Fig. R1 were applied as Supplementary Fig. 45.

3. DFT calculations and MD simulations show that even a small amount of TMSFS can more easily enter the first solvation shell of Li^+. However, the experimental results from Raman or NMR suggest that the effect of TMSFS on the electrolyte solvation structure

is not very pronounced. Could the authors use additional characterization methods to demonstrate the additive strongly coordinated solvation structure? Alternatively, the limited observed effect on solvation structure maybe due to the low additive content, and if so, could higher additive concentrations be tested to examine the trend in solvation structure changes?

Response: Thanks for the reviewer's comment. We fully agree that additional characterization could further clarify the solvation effect of TMSFS. In response, we have performed additional characterization methods and tested higher additive concentrations to examine the evolution of the solvation structure.

Firstly, we employed internal-referenced diffusion-ordered spectroscopy (IR-DOSY), which is an effective technique to measure the coordination ratio of specific solvents in an electrolyte with more than one solvent [7]. The 2D ^1H NMR results reveal that upon the addition of the TMSFS, the as-calculated coordination ratio (α) of EMC and FEC decrease from 0.44 and 0.31 in BE to 0.39 and 0.28 in TFFE, respectively. Meanwhile, TMSFS exhibits a markedly high coordination fraction of 0.975, a more accurate descriptor of solvents' ability to solvate Li^+ than the commonly used dielectric constant. These results further substantiate the strong Li^+-coordinating capability of TMSFS and its effectiveness in attenuating solvent–Li^+ coordination. (Fig. R2 to R4).

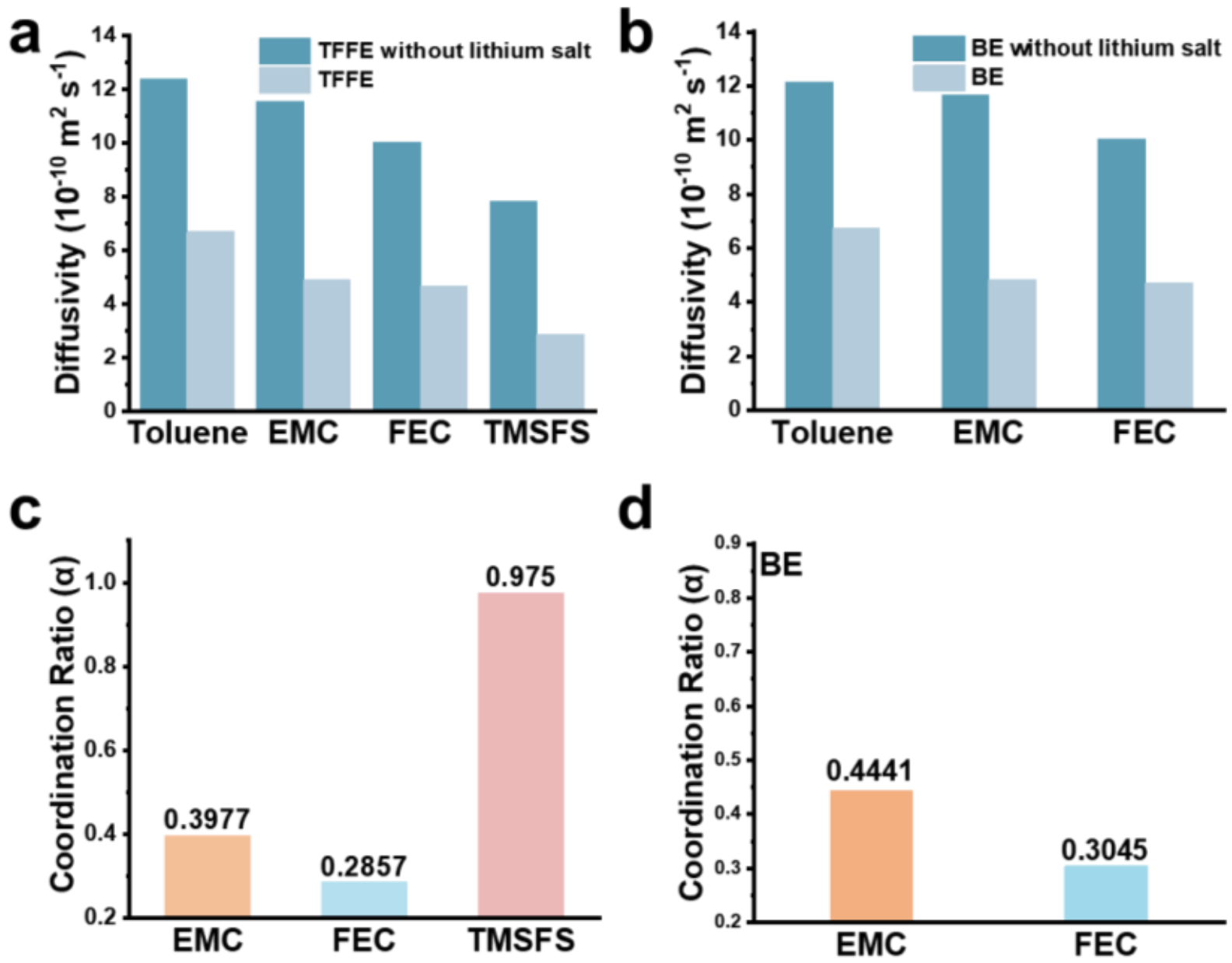

Fig. R2. Diffusion-ordered spectroscopy NMR (DOSY-NMR) analysis. The

diffusivities of multiple components (toluene, EMC, FEC, TMSFS,) in (a)TFFE and TFFE without $LiPF_6$ and (b)BE and BE without $LiPF_6$; Corresponding coordination ratios calculated through DOSY of (c)TFFE and (d)BE. Toluene was used as an internal reference.

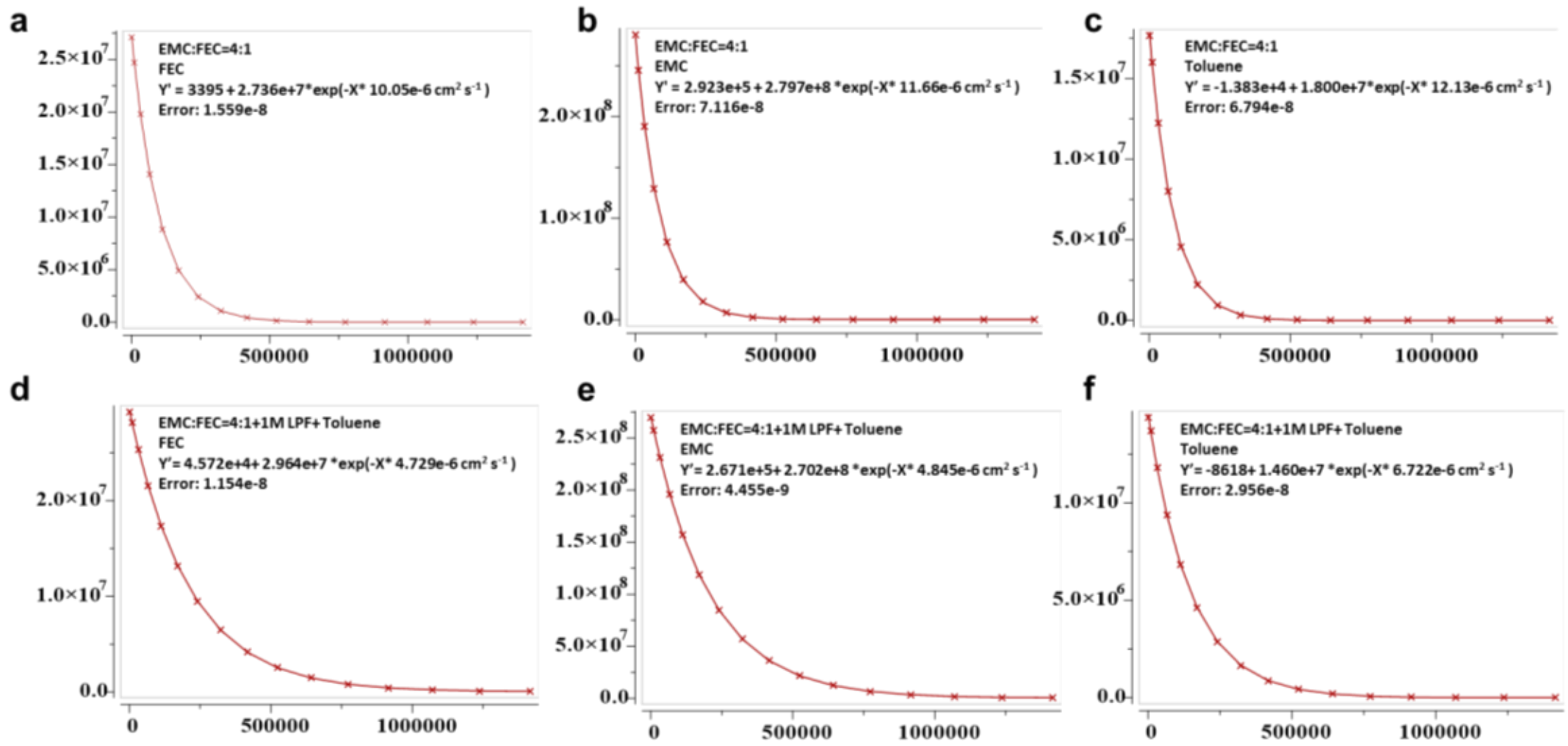

Fig. R3. DOSY-NMR fittings of signal intensity for BE.

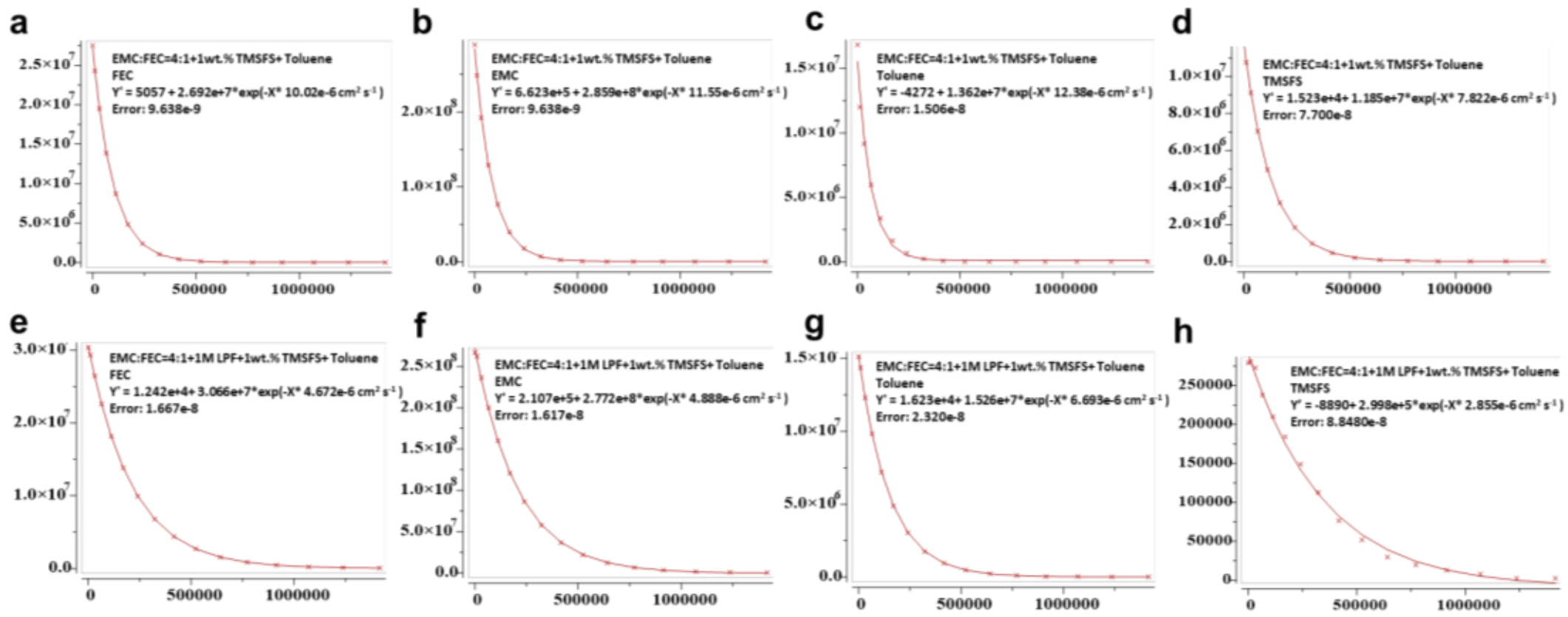

Fig. R4. DOSY-NMR fittings of signal intensity for TFFE.

Meanwhile, we conducted Raman tests for the electrolyte with a series of gradient content of TMSFS (0, 1, 5 wt.%). As shown in the Fig. R5, increasing the additive content leads to a more pronounced solvation structure reconstruction, confirming that the additive is able to partially replace solvent molecules and anions in the first solvation shell of Li^+. Specifically, as the TMSFS content increased from 0 to 5 wt.%, the coordination fractions of FEC, PF_6^-, and EMC decreased progressively, from 0.369, 0.397, and 0.487 (0 wt.%) to 0.298, 0.345, and 0.416 (1 wt.%), and further to 0.231, 0.265, and 0.386 (5 wt.%), respectively.

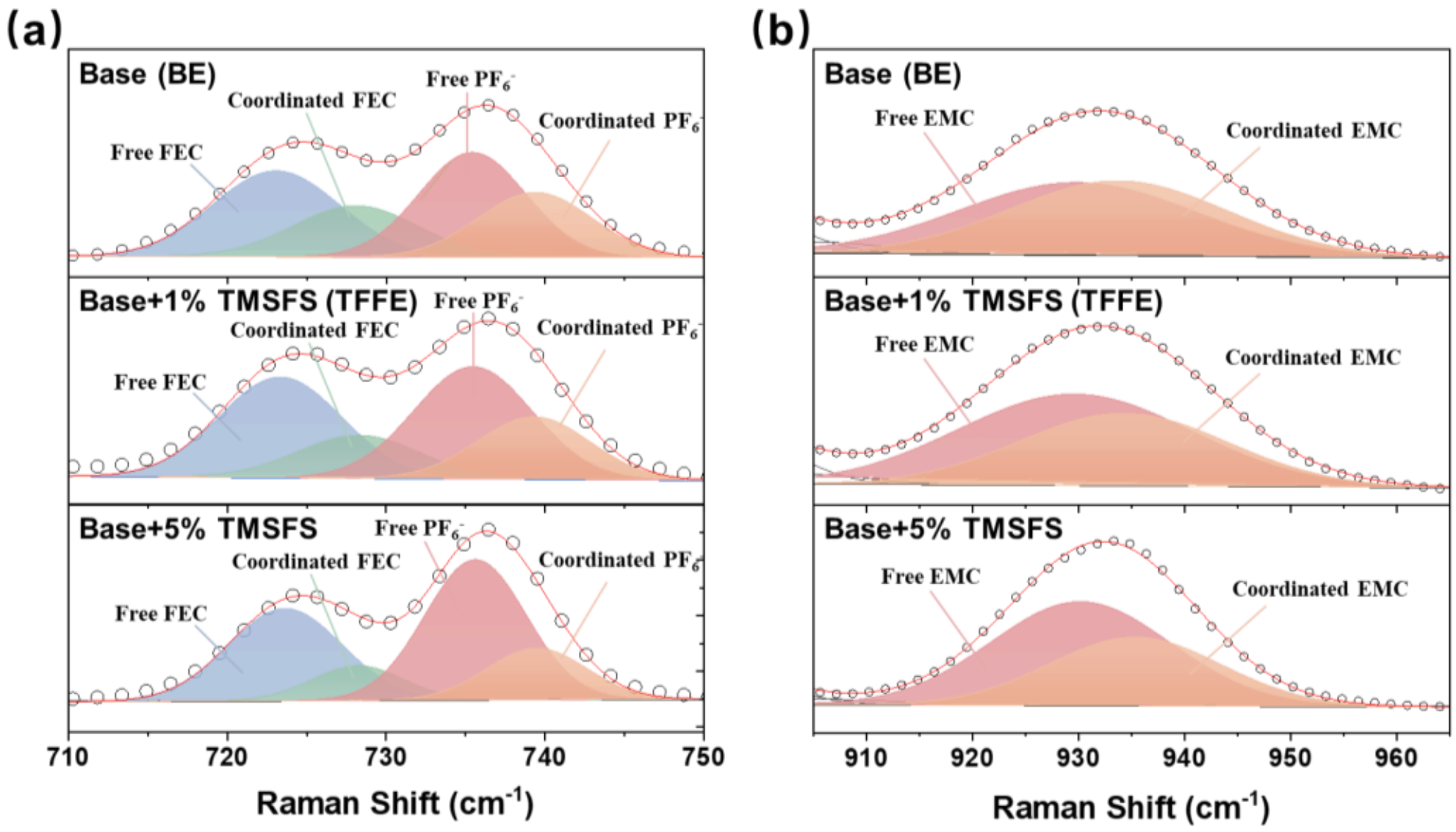

Fig. R5. Raman spectra of BE and BE+1 wt. %, 5 wt.% from (a)710 to 750 cm^{-1} and (b)905 to 965 cm^{-1}.

NMR tests with higher additive concentrations were also conducted to examine the trend in solvation structure changes. In our previous measurements, the use of deuterated DMSO as a solvent led to its partial dissolution into the electrolyte, which interfered with the NMR results. To solve this problem, a coaxial NMR tube configuration was employed to eliminate interference from the deuterated solvent.[8] The ^{7}Li chemical shift displaced to lower values from -0.56 ppm (BE) to -0.58 ppm after adding 1 wt.% TMSFS (TFFE) and further shift to -0.64 as the addition amount increase to 5 wt.% (Fig. R6). The upfield shift implies the increased shielding effect on the Li^+ nucleus, resulting from the weakened interaction between Li^+ with anions.

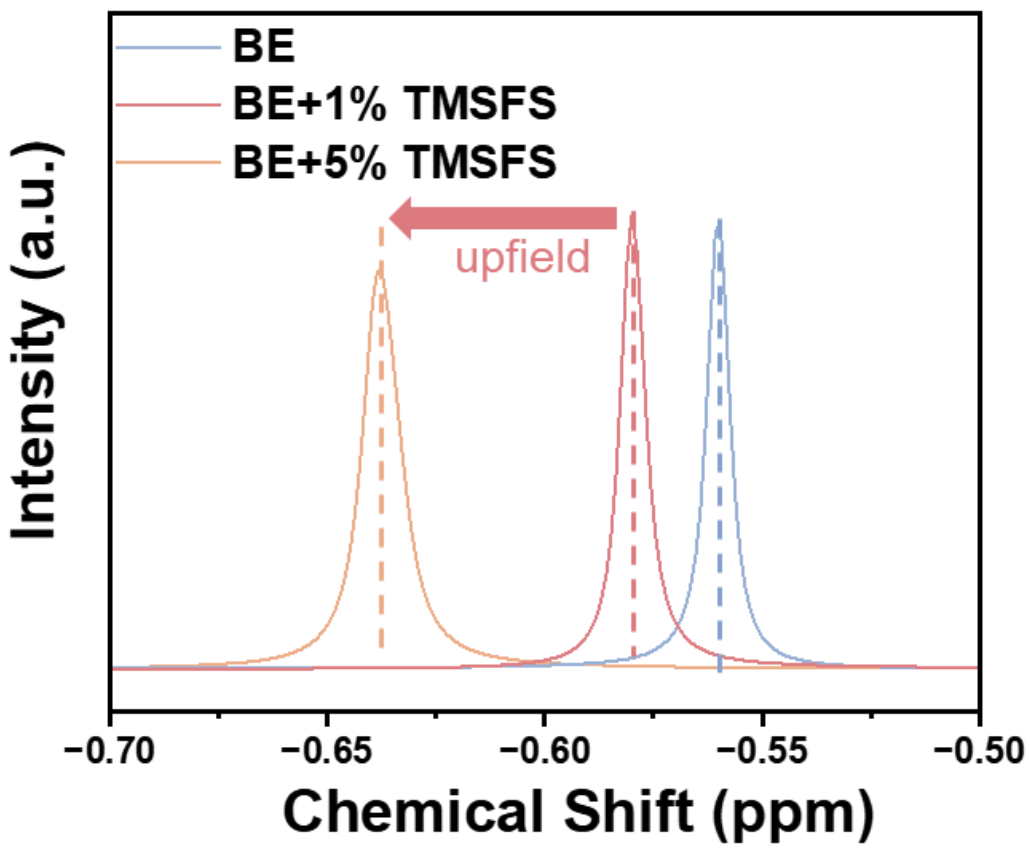

Fig. R6. ^{7}Li NMR spectra of BE and BE+1 wt.%, 5 wt.%.

In the revised manuscript, we have provided more discussion to demonstrate the effect of additive on solvation structure regulation. The changes of the above aspects have

been supplemented in the revised manuscript highlighted with yellow, which can also be found below:

As the TMSFS content increased from 0 to 5 wt.%, the coordination fractions of FEC, PF_6^-, and EMC decreased progressively, from 0.369, 0.397, and 0.487 (0 wt.%) to 0.298, 0.345, and 0.416 (1 wt.%), and further to 0.231, 0.265, and 0.386 (5 wt.%), respectively (Supplementary Figs. S5 and S6), indicating that TMSFS effectively reduced the presence of solvent molecules and anions within the solvation sheath.

Meanwhile, internal-referenced diffusion-ordered spectroscopy (IR-DOSY) were employed to measure the solvent diffusivity. The two-dimensional ^1H NMR results reveal that upon the addition of the TMSFS, the as-calculated coordination ratio (α) of EMC and FEC decrease from 0.44 and 0.31 in BE to 0.39 and 0.28 in TFFE, respectively. Meanwhile, TMSFS exhibits a markedly high coordination fraction of 0.975. These results further substantiate the strong Li^+-coordinating capability of TMSFS and its effectiveness in attenuating solvent–Li^+ coordination. (Fig. 2g, h and Supplementary Fig. S7-9).

Also, the effect of TMSFS additive in altering the Li^+ solvation structure was further confirmed by ^7Li NMR (Supplementary Figs. S10), where the ^7Li chemical shift displaced to lower values from -0.56 ppm (BE) to -0.58 ppm after adding 1 wt.% TMSFS and further shift to -0.64 ppm as the addition amount increase to 5 wt.%. The upfield shift implies the increased shielding effect on the Li^+ nucleus, resulting from the weakened interaction between Li+ with anions[29].

***Fig. R2** were applied as Supplementary Figure 7 and Fig. 2g and h.*

***Fig. R3** were applied as Supplementary Figure 8.*

***Fig. R4** were applied as Supplementary Figure 9.*

***Fig. R5** were applied as Supplementary Figure 5.*

***Fig. R6** were applied as Supplementary Figure 10.*

4. According to the authors' description, TMSFS interacts more strongly with Li^+ compared to the anion and solvents. Could the authors clarify whether $LiPF_6$ can dissolve in TMSFS alone?

Response: **Response**: Thanks for the reviewer's suggestion. The dielectric constant of TMSFS was first measured and found to be higher than those of common ester solvents (EMC, DMC, and DEC) [9], indicating its relatively strong polarity (Fig. R7 and Table R4). However, it is reported that when the number of carbon atoms in the carbon chain of a solvent molecule exceeds five, the solvation ability toward lithium salts will be compromised. [9] Consequently, TMSFS can dissolve $LiPF_6$ up to a concentration of 0.1 M (Fig. R8a) owing to its long carbon chain length (5 carbon atoms).

Simultaneously, owing to the ability of TMSFS to promote lithium salt dissociation by suppressing anion coordination, its incorporation into the matrix solvent containing 4 M $LiPF_6$ enhances the solubility of the salt (Fig. R8b).

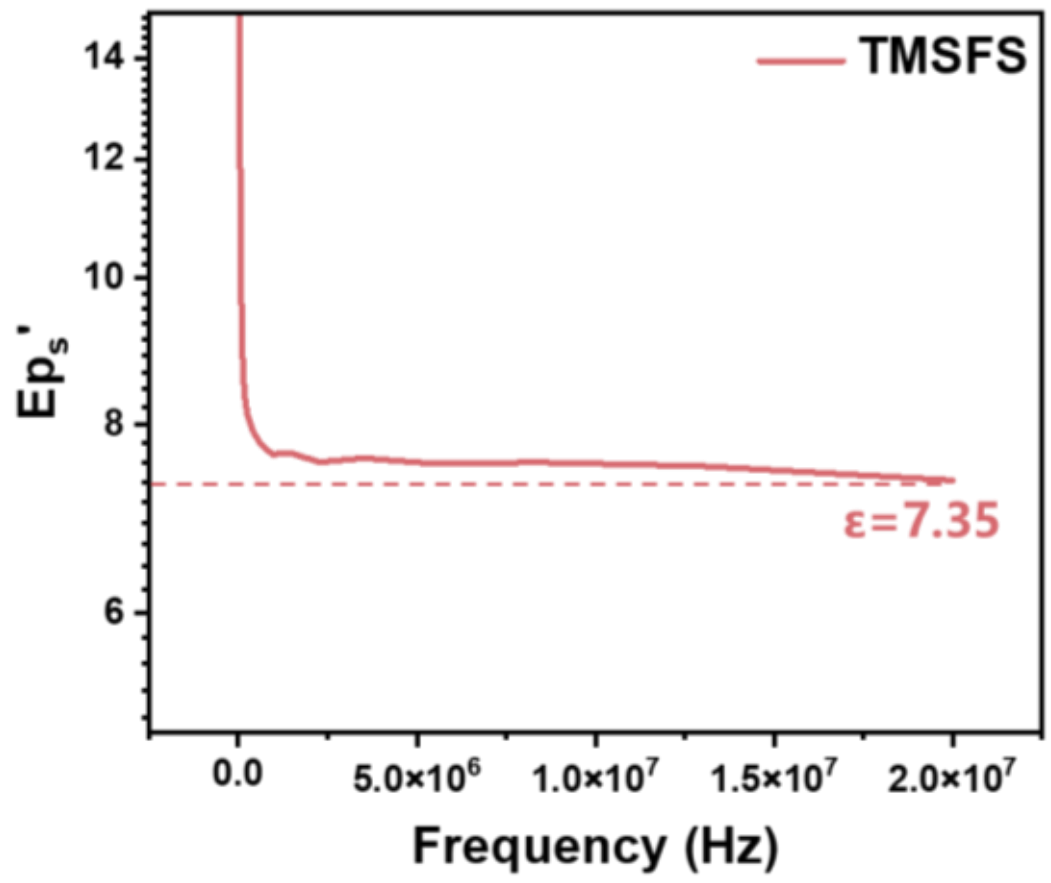

Fig. R7. Tested dielectric constant of TMSFS.

Table R4. The comparison of dielectric constant of several common electrolyte. solvents.

Solvent	Dielectric constant
**TMSFS**	**7.35**
EMC	2.96
DEC	2.81
DMC	3.11
MP	6.2
EP	5.7
EB	5.1
BA	5.1
THF	7.58
DOL	6.74
DME	5.5
DEGDME	7.23

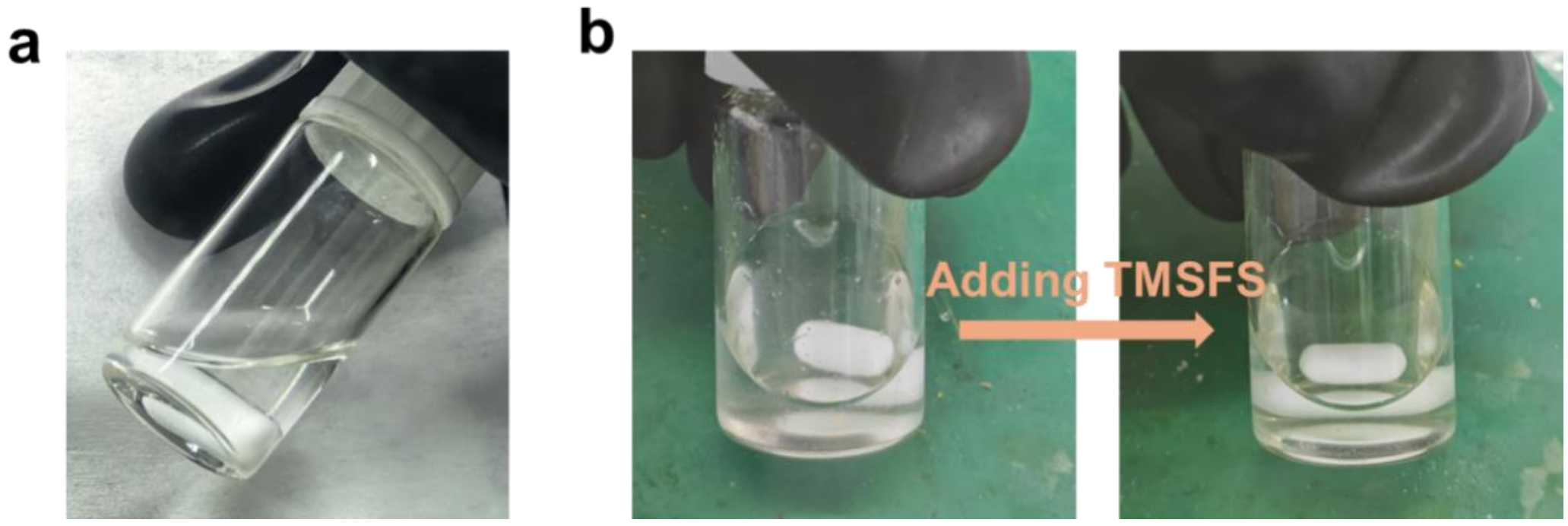

Fig. R8. (a) TMSFS dissolve 0.1M liPF6; (b) comparison of the solution of electrolyte (EMC/FEC+4 M $LiPF_6$) before and after TMSFS's adding

Other minor issues:

5. Figure 2: Could the authors provide the Raman spectra of pure FEC and EMC solvents in the main text or Supplementary Materials for reference?

Response: Thanks for the reviewer's suggestion. In the revised manuscript, we have added the Raman spectra of free FEC and EMC solvents in the Fig. R9, and the Raman peak deconvolution was performed guided by these results.

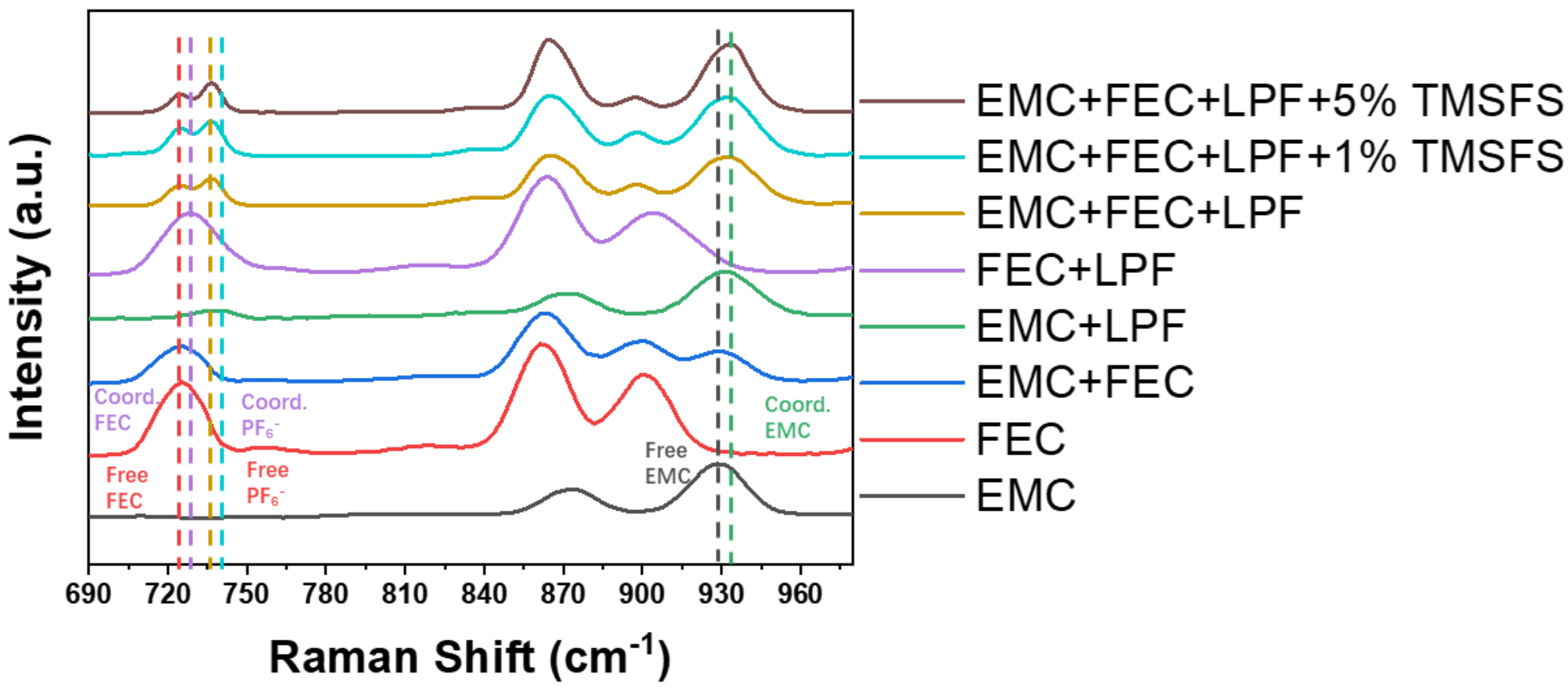

Fig. R9. Raman spectra analysis of Li^+ solvation structures in electrolytes with different contents from 690 to 970 cm^{-1}.

The changes of the above aspects have been supplemented in the revised manuscript highlighted with yellow, which can also be found below:

Fig. R9 *were applied as Supplementary Figure 6.*

6. Page 7, Line 164: Typo errors in the chemical formula.

Response: Thanks for the reviewer's suggestion. We have revised this error.

7. Figure S11: What is the reason for the sudden change in the overpotential of the symmetrical cell during the first 20 cycles?

Response: Thanks for the reviewer's comment. Firstly, the small changes in the overpotential of the symmetrical cell during the first 20 cycles are not a signal of short circuit because there is no evidence of continuous decrease[10,11], but a corresponding result of SEI forming (Fig. R10). During the initial cycles, a solid electrolyte interphase (SEI) is formed on the electrode surface. This early stage may be accompanied by fluctuations in the overpotential. As the interphase gradually stabilizes and the ion-transport pathways become optimized, the overpotential progressively approaches a steady state in the subsequent cycling process.[12]

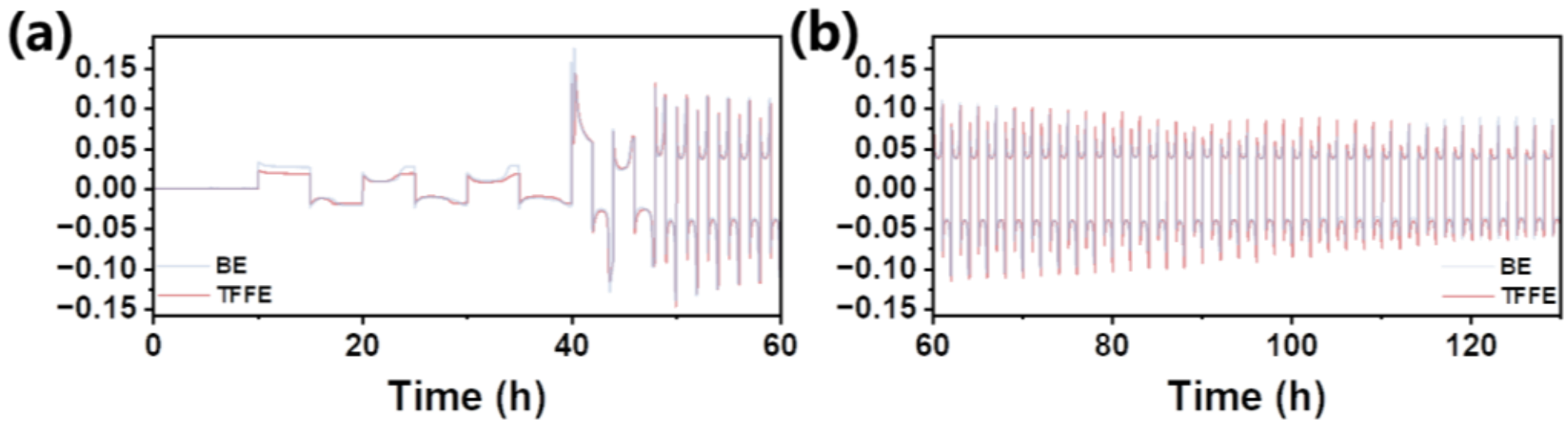

Fig. R10. First 20 cycles of BE- and TFFE-assembled Li||Li symmetric cells at 1 mA cm^{-2} and 1 mAh cm^{-2}.

In the revised manuscript, we have provided a description of the initial overpotential fluctuation phenomenon in the text:

*The sudden change observed in the initial overpotential were attributed to the formation of the SEI laye*r.

8. The rate information should be provided in Figure S19.

Response: Thanks for the reviewer's suggestion. We have provided the specific rate information in the Fig. R11.

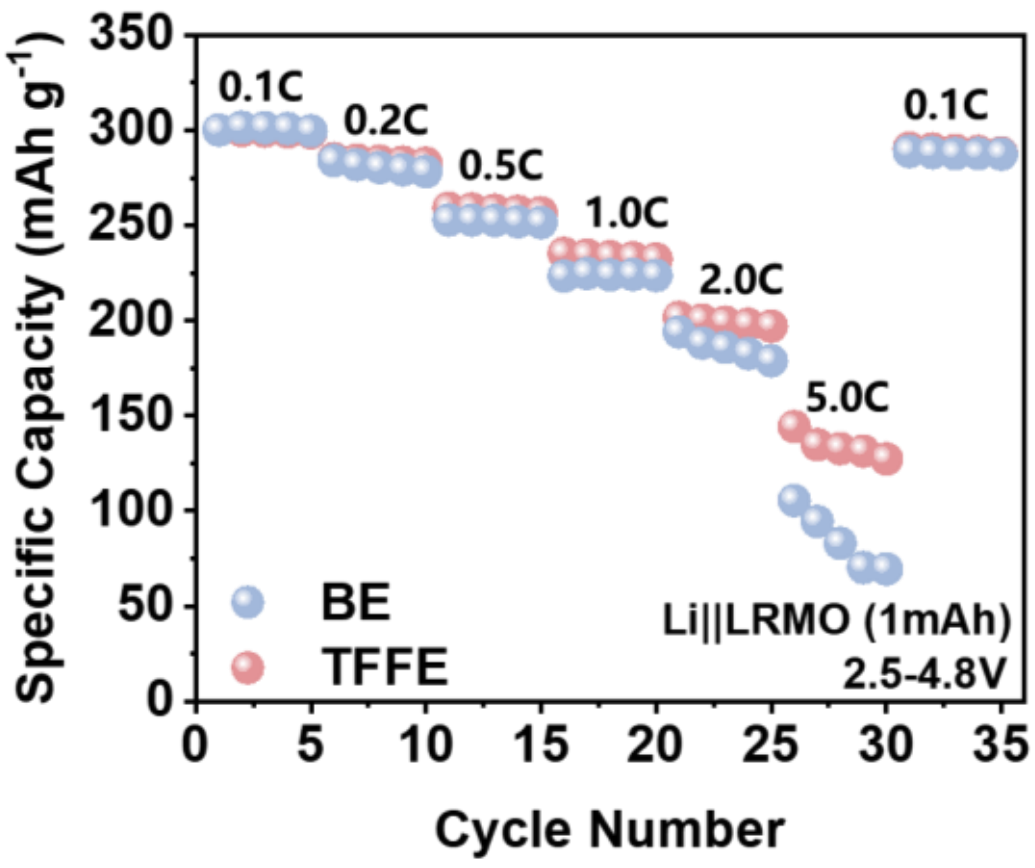

Fig. R11. Rate capability of Li||LRMO full cells assembled with BE and TFFE electrolyte.

Fig. R11 *were applied as Supplementary Figure 32.*

9. The dQ/dV-Voltage profiles in Figure S20 are difficult to interpret in terms of phase transitions and capacity decay; could the authors label these features more clearly in the figure?

Response: Thanks for the reviewer's suggestion. We have labelled the peak features attributed to anionic reduction reactions during charge-discharge process more clearly in the Fig. R12. With cycling, the reduction peak of Mn gradually shifts toward lower potential, indicating that the material undergoes a phase transition from layered phase to spinel phase, which is the main reason for a decrease of average discharge voltage.[13] After 100 cycles, the TFFE-modified system retains a well-defined dQ/dV profile, with the Mn-reduction peak stabilized at 2.85 V, whereas the BE system exhibits a further shift to 2.78 V. Such suppression of peak migration confirms the mitigated phase transition and the reduced voltage decay.[14,15]

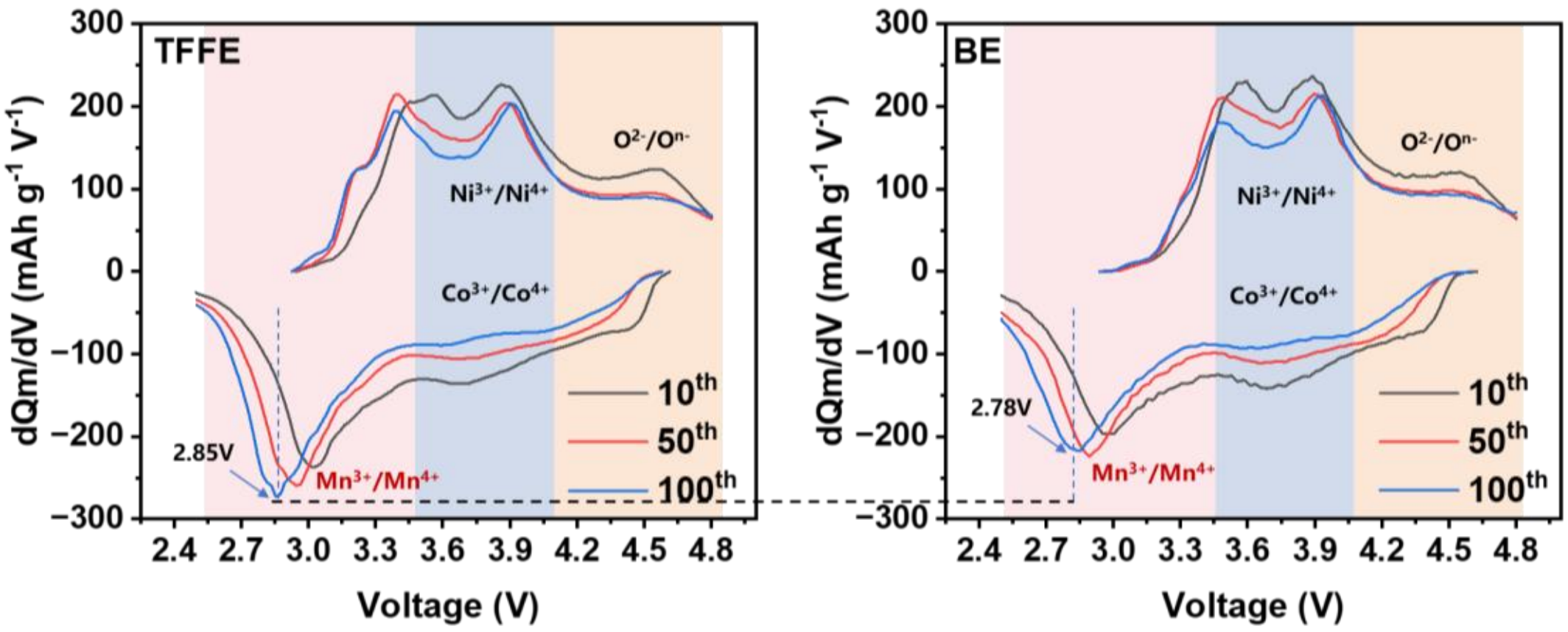

Fig. R12. DQ/DV-Voltage of Li||LRMO full cell from 10-100 cycles of (a)TFFE and (b) BE system.

In the revised manuscript, we have provided more detailed information about the dQ/dV-Voltage profiles to interpret in terms of phase transitions and capacity decay. The changes of the above aspects have been supplemented in the revised manuscript highlighted with yellow, which can also be found below:

Selected dQ/dV-voltage curves revealed that TFFE-based cell obtained a reduced polarization and capacity decay, preserving a well-defined dQ/dV profile, with the Mn reduction peak remaining at 2.85 V (Supplementary Fig. S33a), while LRMO materials undergone serious irreversible phase transitions and capacity decay in BE, showing a further peak shift to 2.78 V (Supplementary Fig. S33b), consistent with the results observed in EIS measurements (Supplementary Fig. S34).

***Fig. R12** were applied as Supplementary Figure 33.*

Reviewer :2

Comments:

This work describes an additive coordinated solvation structure-based strategy to enhance the stability and lifespan of high-voltage lithium metal batteries (LMBs). A newly designed TMSFS molecule with electron push–pull effects coordinates strongly with Li^+, replacing solvent and anion species in the inner solvation shell. This mitigates excessive solvent/anion consumption and promotes the formation of inorganic-rich SEI/CEI layers (Li_2S, LiF) for interfacial stability. As a result, Li//NCM811 pouch cells achieved 550.7 Wh/kg with 80% retention over 180 cycles, while Li//LRMO pouch cells reached 602.5 Wh/kg with 80% retention for 60 cycles. These findings demonstrate that even a low concentration (1 vol%) additive can significantly improve the practical performance of high-energy-density LMBs. Although the suggested results are interesting, there are several points that should be revised in the revision process.

1. A deeper discussion on the effect of molecular structure on its electrochemical behaviors should be attempted. For instance, the authors utilized acetate functional groups, which have been well-recognized for facilitating electrochemical oxidation due to their presence. In this case, how can the electrochemical oxidation reaction be controlled in the presence of such a weak functional group? More specifying the molecular structure of TMSFS seems to be necessary.

Response: Thanks for the reviewer's comment and we agree that acetate functionalities are generally considered electrochemically weak in the sense that they facilitate oxidation. Our design of TMSFS indeed takes advantage of this property, but embeds the acetate unit into a well-defined molecular framework that controls where and how oxidation occurs.

As noted by the reviewer, reactive carboxylate ester molecules containing α-hydrogen atoms (e.g., ethyl acetate, EA) preferentially reside at the electrode–electrolyte interface within the electric double layer. Upon direct contact with the lithium metal surface, the chemically unstable α-hydrogen undergoes Claisen ester condensation reactions (CECR), leading to severe chemical corrosion of the lithium anode accompanied by

hydrogen evolution. These parasitic reactions disrupt the electrode morphology and consequently deteriorate electrochemical performance.[16]

Our molecular design follows the principle of a sacrificial additive, aiming to preferentially decompose ahead of bulk solvents to construct a robust interphase while maximizing additive utilization efficiency. Most importantly, the presence of α-hydrogen atoms mentioned are eliminated via fluorine substitution by utilizing the present molecular design, thereby effectively suppressing such unfavorable interfacial reactions. Meanwhile, the preferential decomposition of the fluorinated additive promotes the formation of a fluoride-rich solid electrolyte interphase.

To gain deeper insight into the mechanisms by which electrochemical oxidation is controlled in the presence of an acetate functional group, we performed additional calculations, as detailed below. Firstly, DFT calculations show that the oxidation potential (ORP) of TMSFS is lower than that of EMC and FEC (Table R5), confirming that TMSFS is intrinsically more easily oxidized than the baseline carbonate solvents, which is consistent with the higher HOMO level of the TMSFS–Li^+ complex and the preferential oxidation peak observed in LSV (Fig. R13).

Table R5. The comparison of calculated oxidation potential of TMSFS, FEC and EMC.

System	ORP (eV)
TMSFS	9.786218
FEC	10.79396
EMC	10.09448

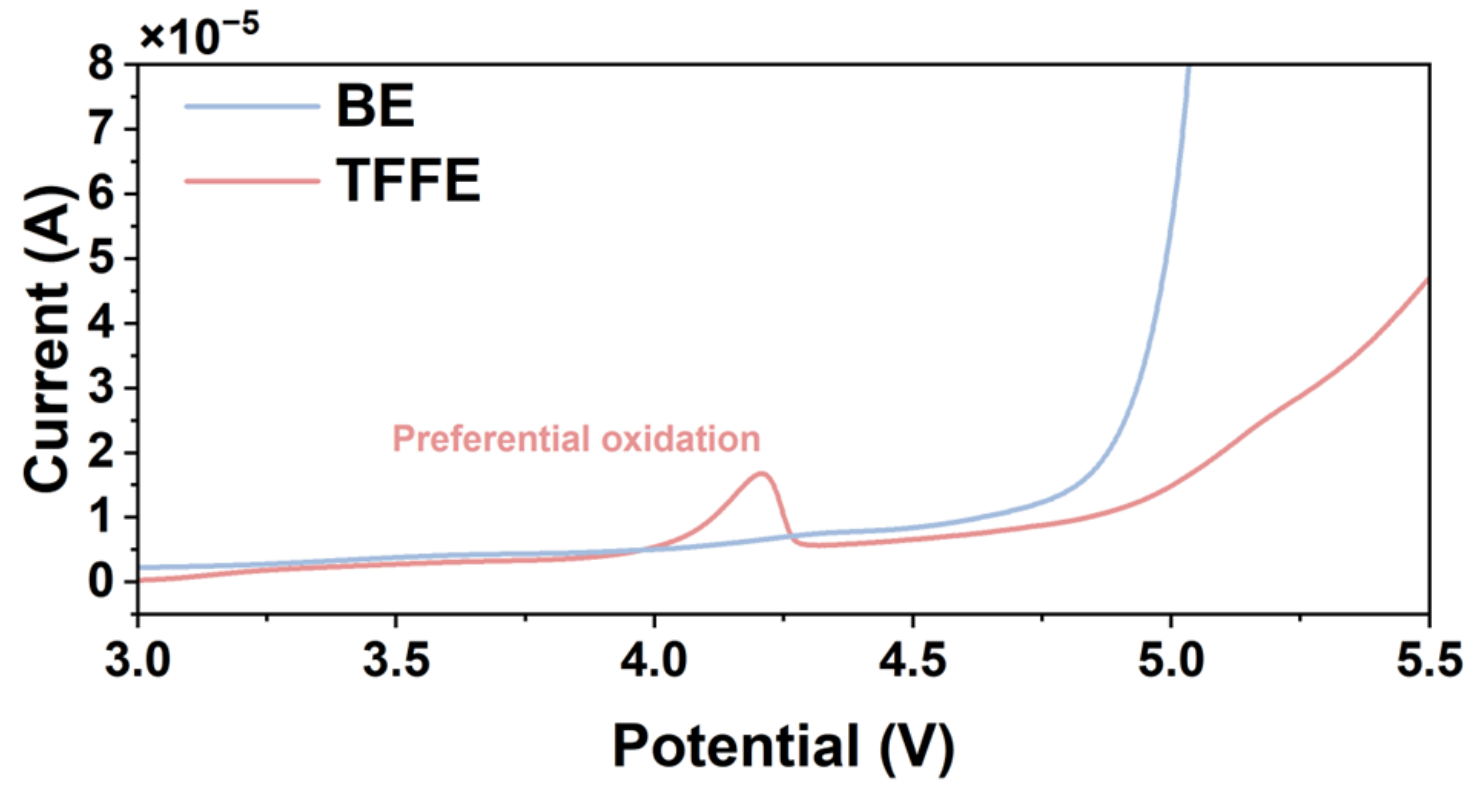

Fig. R13. LSV curves of BE and TFFE, with a scan rate of 0.1 mV s^{-1}, using Al foil

and Li foil as working electrodes.

Secondly, analysis of the frontier orbitals and spin density clarifies how the different fragments of TMSFS participate in this oxidation. The HOMO of neutral TMSFS is delocalized over the entire Si–O–C(=O)–CF_2–SO_2F backbone, indicating electronic communication between the trimethylsilyl group, the acetate bridge and the fluorosulfonyl tail (Fig. R14a). Upon vertical one-electron oxidation, the Kohn–Sham singly occupied molecular orbital (SOMO) of $TMSFS^+$ is localized predominantly on the trimethylsilyl fragment, with only a small lobe extending toward the carbonyl oxygen and essentially no contribution from the remote fluorosulfonyl end (Fig. R14b). The spin-density map of $TMSFS^+$ shows several regions of spin accumulation (Fig. R14c), located mainly on the trimethylsilyl group and around the acetate linkage, while the terminal fluorosulfonyl group displays only negligible spin density. Collectively, these results demonstrate that oxidation in TMSFS is preferentially centered on the trimethylsilyl group rather than on the ester functionality.

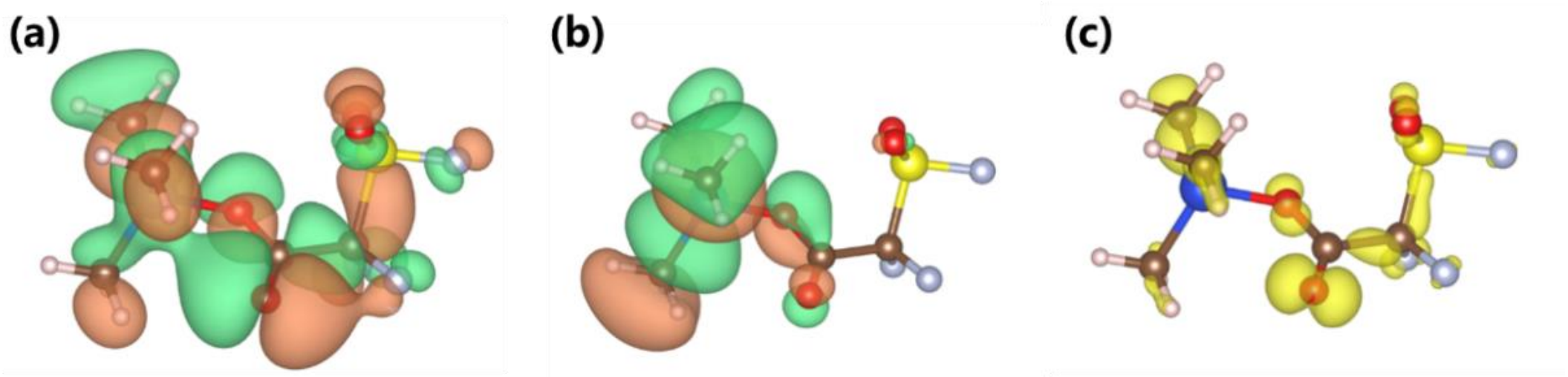

Fig. R14. (a) HOMO of neutral TMSFS; (b) SOMO of $TMSFS^+$ and (c) spin-density map of $TMSFS^+$.

To further corroborate the preferential oxidation site, we analyzed bond-length changes upon one-electron oxidation. Specifically, we compared the optimized geometries of neutral TMSFS and its radical cation $TMSFS^+$ and evaluated the bond-length variation $\%\Delta r = (r(TMSFS^+) - r(TMSFS))/r(TMSFS)$ along the Si–O–C(=O)–CF_2–SO_2F backbone. As shown in Fig. R15, the bonds associated with unfavorable decomposition pathways, such as Si2–O6, O7–C14, and C13–C14, all become slightly shorter upon oxidation (negative Δr values), indicating that these connections are actually strengthened rather than selectively weakened and are therefore unlikely to serve as primary cleavage sites. In contrast, the Si2–C12 and O6–C14 bonds exhibit much larger

positive Δr values, pointing to a preferential weakening of the trimethylsilyl–carbon bond and the adjacent acetate linkage after oxidation.

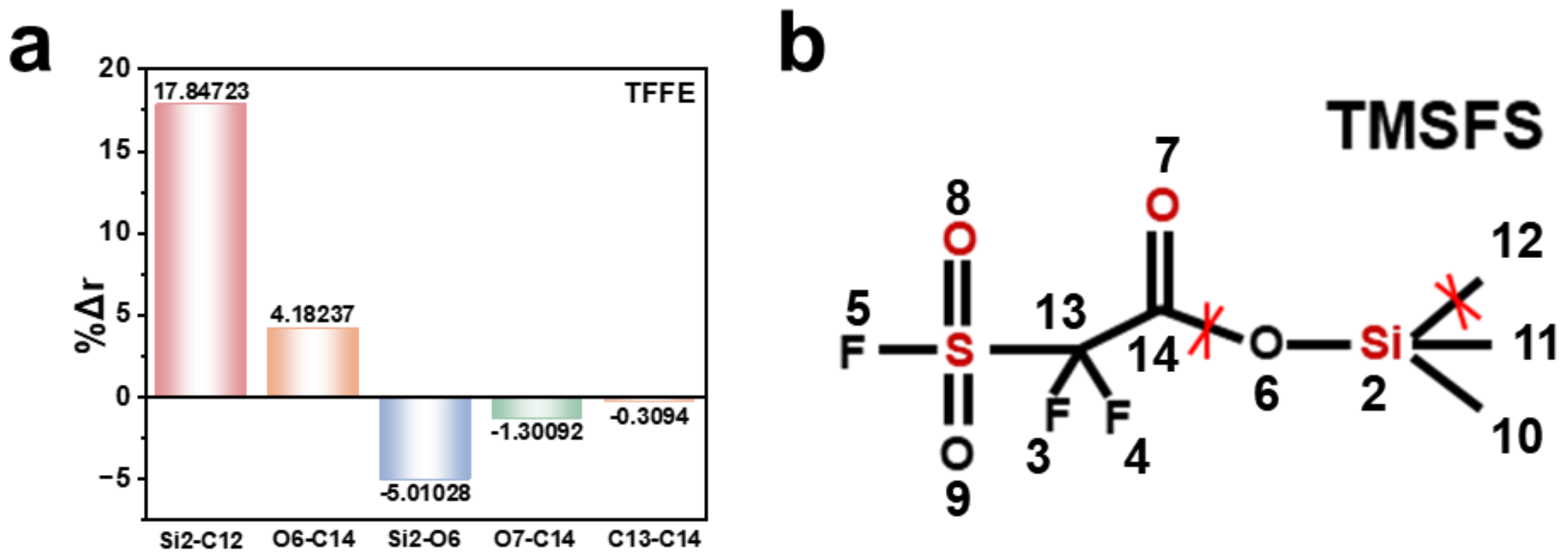

Fig. R15. (a) Bond-length variations of several main bonds; (b) the corresponding number of atoms of TMSFS.

ToF-SIMS analysis reveals the presence of $SiOC_2H_6^-$ species in the interfacial layers on both the cathode and anode sides (Fig. R16), which further validates the reliability of the bond-length variation trends. The Si–O network further exhibits enhanced tolerance toward high-voltage operation and strongly reducing environments.[17,18]

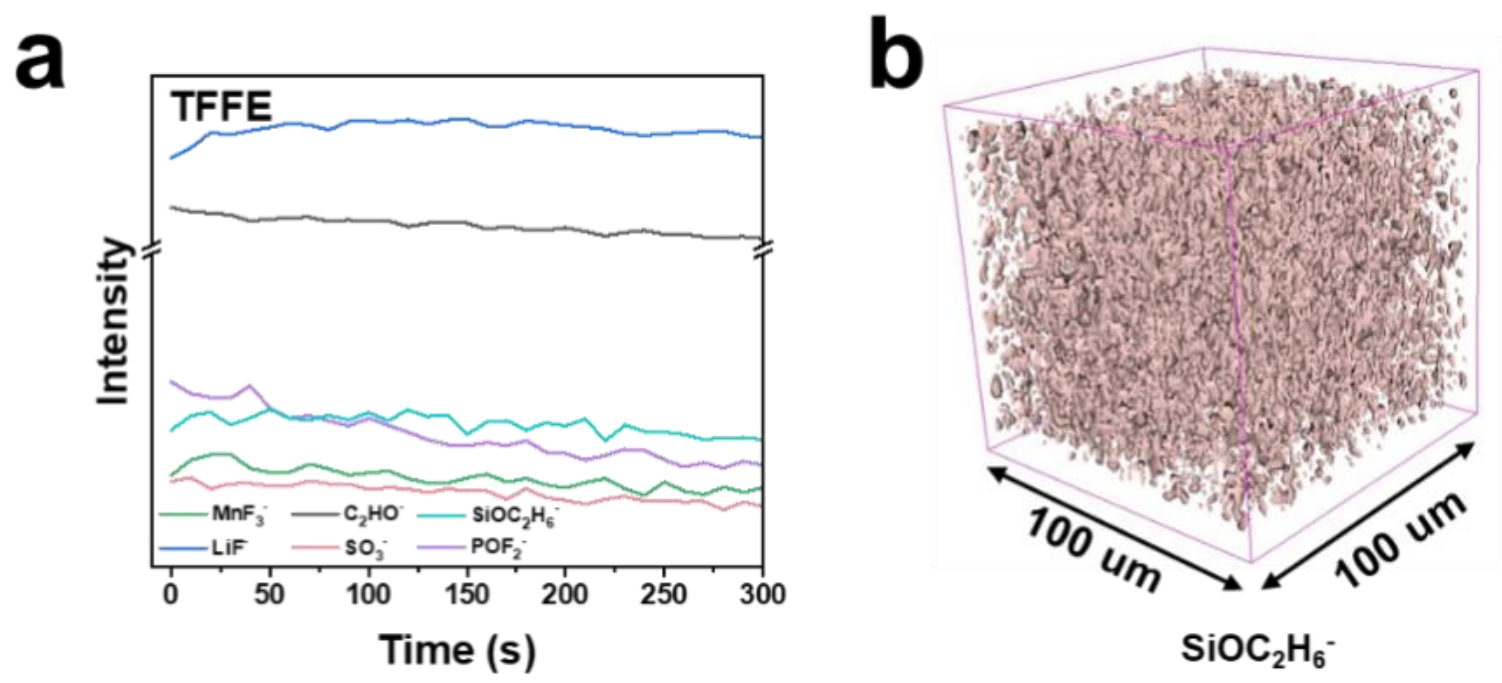

Fig. R16. (a) Sputtering time-dependent compositional profiles of selected secondary ion fragments of different CEI component; corresponding 3D images of $SiOC_2H_6^-$ fragment.

Overall, the TMSFS molecules can preferentially decompose on the electrode surface to generate favorable interface species while suppressing the formation of of harmful substances. In the revised manuscript, we have provided more information about the oxidability of TMSFS. The changes of the above aspects have been supplemented in the revised manuscript highlighted with yellow, which can also be found below:

The α-hydrogen sites of acetate group are eliminated through fluorine substitution,

thereby effectively suppressing the Claisen ester condensation reactions (CECR) and promoting the formation of fluorinated interfaces layer.

The trimethylsilyl group exhibits strong electron-donating character, while the electron-withdrawing nature of the –CF_2–SO_2F– moiety concentrates negative charge on the sulfonyl and carbonyl oxygen atoms. These complementary electronic effects synergistically elevate both the orbital energy (-33.74 kcal mol^{-1}) and the electrostatic energy (-43.40 kcal mol^{-1}). As a result, TMSFS achieves the highest Li^+ binding energy among the studied reported candidates (-62.5 kcal mol^{-1}), fully satisfying the design criteria for the ACSS electrolyte.

To verify whether the rational design of TMSFS translates into the desired oxidation behavior, frontier orbital and spin-density analyses were performed (Supplementary Fig. S2). The results indicate that oxidation is preferentially localized on the trimethylsilyl moiety, as evidenced by singly occupied molecular orbital (SOMO) localization and dominant spin accumulation on the Si-containing fragment, with negligible contribution from the fluorosulfonyl group, thereby favoring the formation of beneficial interfacial oxidation products rather than detrimental decomposition pathways.

***Fig. R13** were applied as Supplementary Figure 29.*

***Fig. R14** were applied as Supplementary Figure 2.*

***Fig. R16** were applied as Supplementary Figure 14 and 15.*

2. Although there are electron-withdrawing groups (F) in the TMSFS, the authors mentioned that the other electron-donating group can participate in increasing Li^+ coordination; it is somewhat ambiguous. There are different functional groups in the TMSFS; which functional group mainly contributes to enhancing the Li^+ coordination, and why? A deeper discussion is also necessary in this section. Another word, further discussion on the TMSFS, including its role within the functional group, should be provided.

Response: Thanks for the reviewer's comment regarding the role of different functional groups in TMSFS and their contribution to Li^+ coordination. Firstly, compared with the

TMS and FS molecules, TMSFS exhibits the highest binding energy toward Li^+, indicating its superior capability for strong Li^+ coordination. In response to the reviewer's comment, energy decomposition analysis (EDA) was performed to quantitatively elucidate the underlying Li^+–TMSFS interaction mechanism. The EDA results reveal that the strong affinity between TMSFS and Li^+ originates predominantly from two contributions: electrostatic energy and orbital energy.

Table R6. Energy Decomposition Analysis of TMSFS, FS and TMS.

System	Binding Energy	Orbital Energy	Electrostatic Energy
TMSFS	-62.45	-33.74	-43.40
TMS	-55.49	-26.68	-44.11
FS	-56.21	-31.70	-37.98

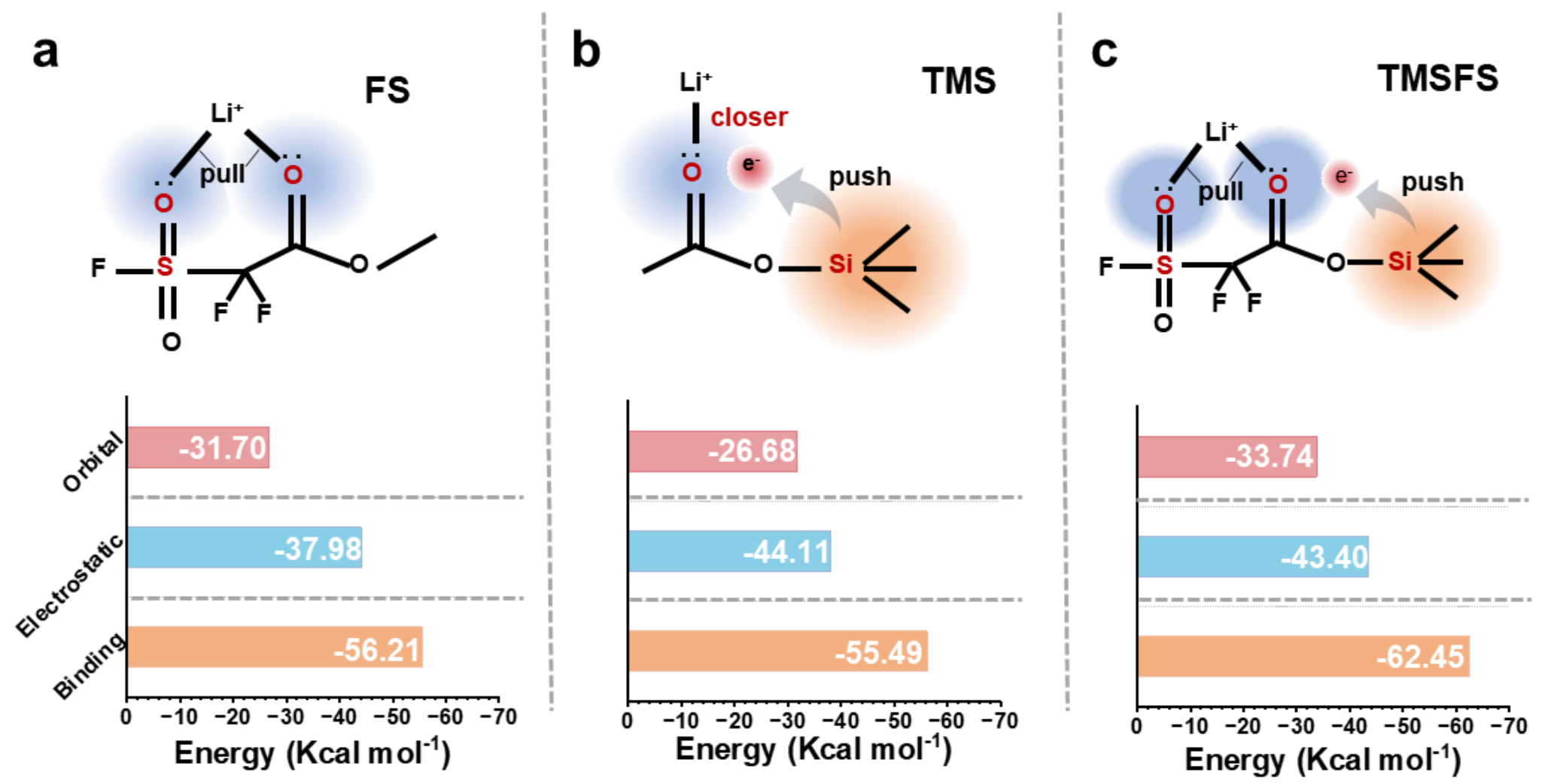

Fig. R17. EDA on the Li^+ complexes with (a) FS, (b)TMS, and (c)TMSFS.

As illustrated in Fig. R17 and Table R6, the Li^+–FS interaction is characterized by a more pronounced orbital contribution (-31.70 kcal mol^{-1}), indicating that the $–SO_2F–CHF_2-CO_2–$ fragment is highly polarizable and readily undergoes electron redistribution upon Li^+ coordination. In contrast, the Li^+–TMS complex exhibits a dominant electrostatic contribution (-44.11 kcal mol^{-1}), which can be attributed to the strong electron-donating effect of the trimethylsilyl group that enriches negative charge at the carbonyl and sulfonyl oxygen atoms, leading to a strong Coulombic attraction

between Li^+ and O.

For the TMSFS molecule, these two effects are synergistically combined to enhance the interaction to Li^+. The electron-donating effect of the trimethylsilyl group provides a strong electrostatic component comparable to that of TMS, while the electron-withdrawing nature of the fluorinated $–CF_2–SO_2F–$ moiety pulls electron density from the organic backbone and concentrates negative charge on the sulfonyl and carbonyl oxygen atoms. As a result, TMSFS exhibits the highest binding energy toward Li^+ and the enhanced strong Li^+–TMSFS coordination was achieved.

In the revised manuscript, we have provided deeper discussion over the molecular structure of TMSFS and how electrochemical oxidation reaction can be controlled. The changes of the above aspects have been supplemented in the revised manuscript highlighted with yellow, which can also be found below:

Electrostatic potential (ESP) maps reveal high electron density at the carbonyl oxygen (-22.52 kcal mol^{-1}) and sulfonyl oxygen atoms (-18.30 kcal mol^{-1}). This pronounced negative region exerts a strong pull effect on Li^+, enabling the formation of thermodynamically stable six-membered chelate rings. Energy decomposition analysis (EDA) reveals that the FS ligand exhibits substantial orbital interaction energy (-31.70 kcal mol^{-1}) and electrostatic interaction energy (-37.98 kcal mol^{-1}), collectively accounting for the overall Li^+–FS binding energy of -56.21 kcal mol^{-1}.

As shown in Fig. 1e and Supplementary Fig. S1b, the trimethylsilyl group of Trimethylsilylacetate (TMS) exerts a strong electron-pushing effect to the C=O, elevating its ESP to as low as -35.52 kcal mol^{-1}, which demonstrates the validity of this design. However, this type of structural configuration featuring a single C=O coordination is incapable of forming strong and stable coordination with Li^+ ions, which was confirmed by the low orbital (-26.68 kcal mol^{-1}) and binding energy (-55.49 kcal mol^{-1}), thereby limiting its ability to penetrate into the first solvation sheath.

The trimethylsilyl group exhibits strong electron-donating character, while the electron-withdrawing nature of the $–CF_2–SO_2F–$ moiety concentrates negative charge on the sulfonyl and carbonyl oxygen atoms. These complementary electronic effects synergistically elevate both the orbital energy (-33.74 kcal mol^{-1}) and the electrostatic

energy (-43.40 kcal mol^{-1}). As a result, TMSFS achieves the highest Li^+ binding energy among the studied reported candidates (-62.5 kcal mol^{-1}), fully satisfying the design criteria for the ACSS electrolyte.

***Fig. R17** were applied as Fig. 1 d, e and f.*

3. In Figure 1, the conceptual mechanism of the TMSFS was suggested; however, it is difficult to understand the pull-push mechanism. The logic shown in Fig. 1d should be validated or clarified with more scientific discussion.

Response: Thanks for the reviewer's comment regarding the "push–pull" mechanism illustrated in Fig. 1d, e and f. We agree that the concept requires clearer clarification. The "pull" effect corresponding to the lone pair electrons on both carbonyl (C=O) and sulfonyl (S=O) functional groups act as electron-withdrawing motifs to coordinate with Li^+, enabling the formation of stable six-membered rings with Li^+. Moreover, the strong electron-donating effect of the trimethylsilyl group can push the electron towards the carbonyl oxygen atoms, leading to the stronger Coulombic attraction toward Li^+. As for the TMSFS molecule, the electron-donating effect of the trimethylsilyl group provides a strong electrostatic component comparable to that of TMS, while the electron-withdrawing nature of the fluorinated $–CF_2–SO_2F–$ moiety reinforces the concentrated electron distribution at the carbonyl and sulfonyl oxygen atoms, further enhancing Li^+–TMSFS coordination.

To be more detailed, the leading Natural Orbitals for Chemical Valence (NOCV) deformation densities for Li^+–TMSFS ($\Delta\rho_1$–$\Delta\rho_3$ in Fig. R18a) show that electron-density rearrangement upon binding is localized predominantly on the carbonyl and sulfonyl oxygen atoms and around Li^+, whereas the silyl and fluorinated substituents display only weak, remote polarization. Very similar NOCV patterns are observed for Li^+–FS (Fig. R18b), indicating that in both systems Li^+ is coordinated by the same O-rich pocket defined by the carbonyl and sulfonyl groups. Accordingly, TMSFS does not introduce an additional coordination site; instead, the trimethylsilyl group acts as an electron-donating "push" unit, while the fluorosulfonyl moiety increases electron density at the coordinating oxygens, thereby enhancing their ability to attract Li^+

("pull"). The EDA–NOCV analysis thus provides quantitative support for the push–pull mechanism proposed in Fig. 1d. As a result, the combined effect of electron redistribution ("push") and enhanced Li^+ coordination ("pull") synergistically strengthens the interaction between TMSFS and Li^+.

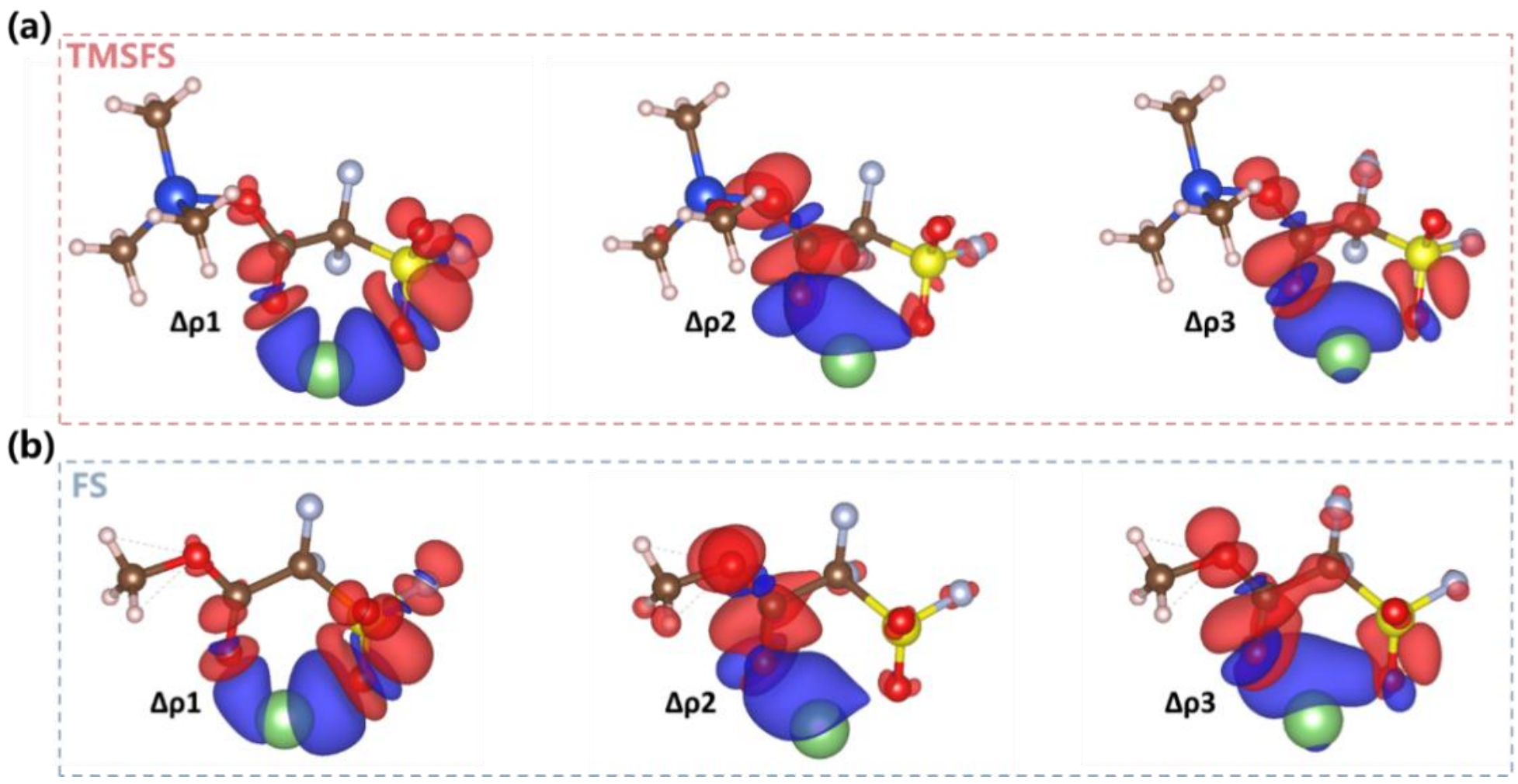

Fig. R18. EDA–NOCV (Energy Decomposition Analysis combined with Natural Orbitals for Chemical Valence) pattern comparisons of (a)TMSFS and (b)FS.

In the revised manuscript, we have provided further discussion to validate the rational basis of the push–pull concept proposed in Fig. 1. The changes of the above aspects have been supplemented in the revised manuscript highlighted with yellow, which can also be found below:

Electrostatic potential (ESP) maps reveal high electron density at the carbonyl oxygen (-22.52 kcal mol^{-1}) and sulfonyl oxygen atoms (-18.30 kcal mol^{-1}). This pronounced negative region exerts a strong pull effect on Li^+, enabling the formation of thermodynamically stable six-membered chelate rings. Energy decomposition analysis (EDA) reveals that the FS ligand exhibits substantial orbital interaction energy (-31.70 kcal mol^{-1}) and electrostatic interaction energy (-37.98 kcal mol^{-1}), collectively accounting for the overall Li^+–FS binding energy of -56.21 kcal mol^{-1}.

As shown in Fig. 1e and Supplementary Fig. S1b, the trimethylsilyl group of Trimethylsilylacetate (TMS) exerts a strong electron-pushing effect to the C=O, elevating its ESP to as low as -35.52 kcal mol^{-1}, which demonstrates the validity of this design. However, this type of structural configuration featuring a single C=O coordination is incapable of forming strong and stable coordination with Li^+ ions, which

was confirmed by the low orbital (-26.68 kcal mol^{-1}) and binding energy (-55.49 kcal mol^{-1}), thereby limiting its ability to penetrate into the first solvation sheath.

The leading natural orbitals for chemical valence (NOCV) deformation densities for Li^+–TMSFS show that electron-density rearrangement upon binding is localized predominantly on the carbonyl and sulfonyl oxygen atoms and around Li^+. Very similar NOCV patterns are observed for Li^+–FS, indicating that in both systems Li^+ is coordinated by the same O-rich pocket defined by the carbonyl and sulfonyl groups.

The trimethylsilyl group exhibits strong electron-donating character, while the electron-withdrawing nature of the –CF_2–SO_2F– moiety concentrates negative charge on the sulfonyl and carbonyl oxygen atoms. These complementary electronic effects synergistically elevate both the orbital energy (-33.74 kcal mol^{-1}) and the electrostatic energy (-43.40 kcal mol^{-1}). As a result, TMSFS achieves the highest Li^+ binding energy among the studied reported candidates (-62.5 kcal mol^{-1}), fully satisfying the design criteria for the ACSS electrolyte.

4. Although the electrochemical performances seem to be improved, dependence on the TMSFS amounts should be suggested in the main manuscript.

Response: Thanks for the reviewer's suggestion regarding the dependence of electrochemical performance on the TMSFS content. In response, we have systematically investigated the effect of TMSFS concentration on the electrochemical behavior of half cells. As shown in the Fig. R19, cells containing 1 wt.% TMSFS exhibit the most stable cycling performance compared to both the baseline electrolyte and electrolytes with higher additive contents.

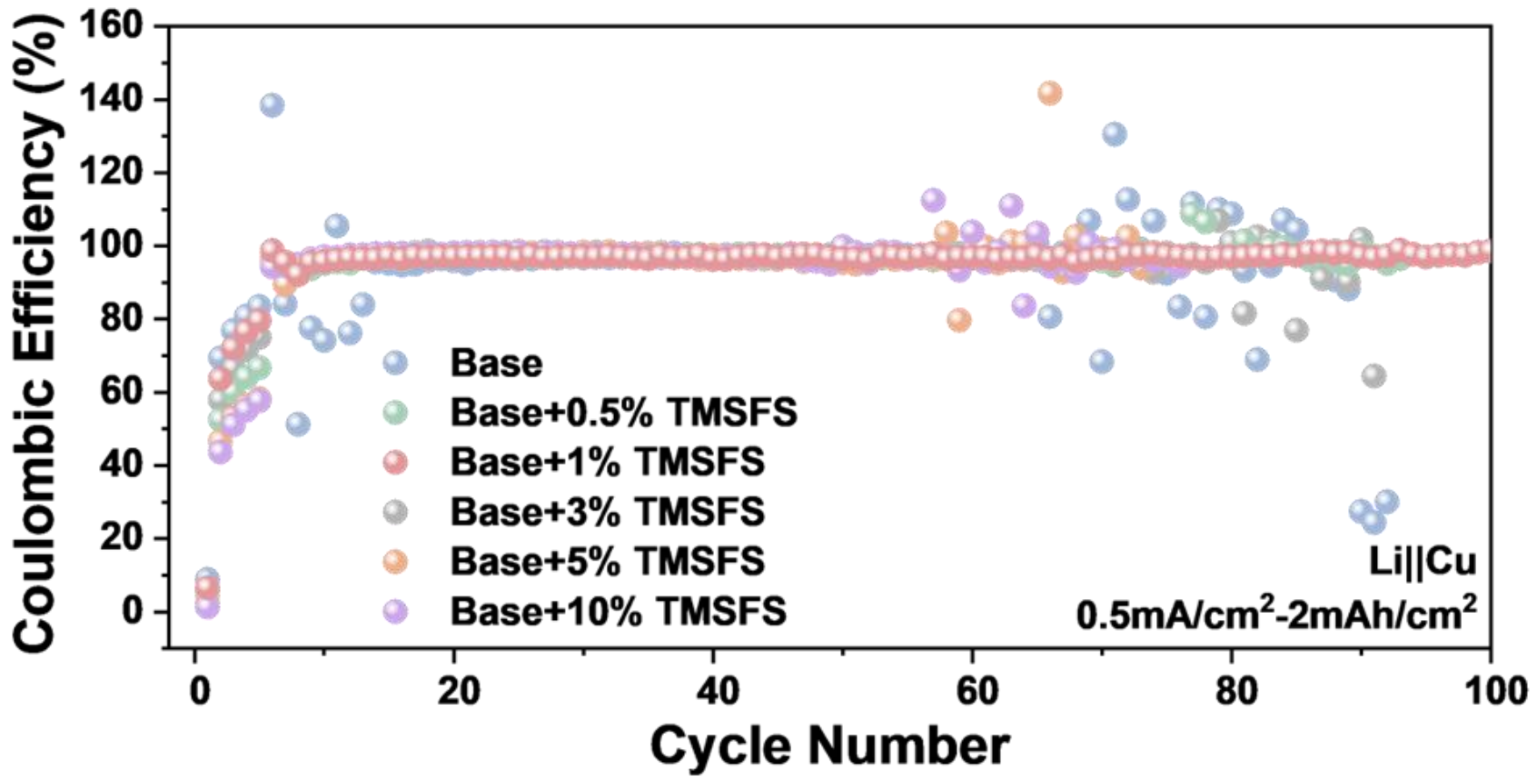

Fig. R19. Cycling stability of BE and different amounts of TMSFS based Li||Cu half-cell at 0.5 mA cm^{-2} and 2 mAh cm^{-2}.

Furthermore, 3mAh cm^{-2} of Li was deposited on the Cu foil to form SEI with different addition amount of TMSFS. According to the EIS results in Fig. R20, the impedance of the formed SEI showed a higher tendency as the addition amount increased from 1 wt.% to 10 wt.%, which indicates excessive addition may induce thicker or uneven SEI. Meanwhile, the cross-section FIB-SEM images and shots of surface states after several cycles (Fig. R21) also showed thicker interface reaction layer suggesting fierce side reactions caused by excessive addition of TMSFS.

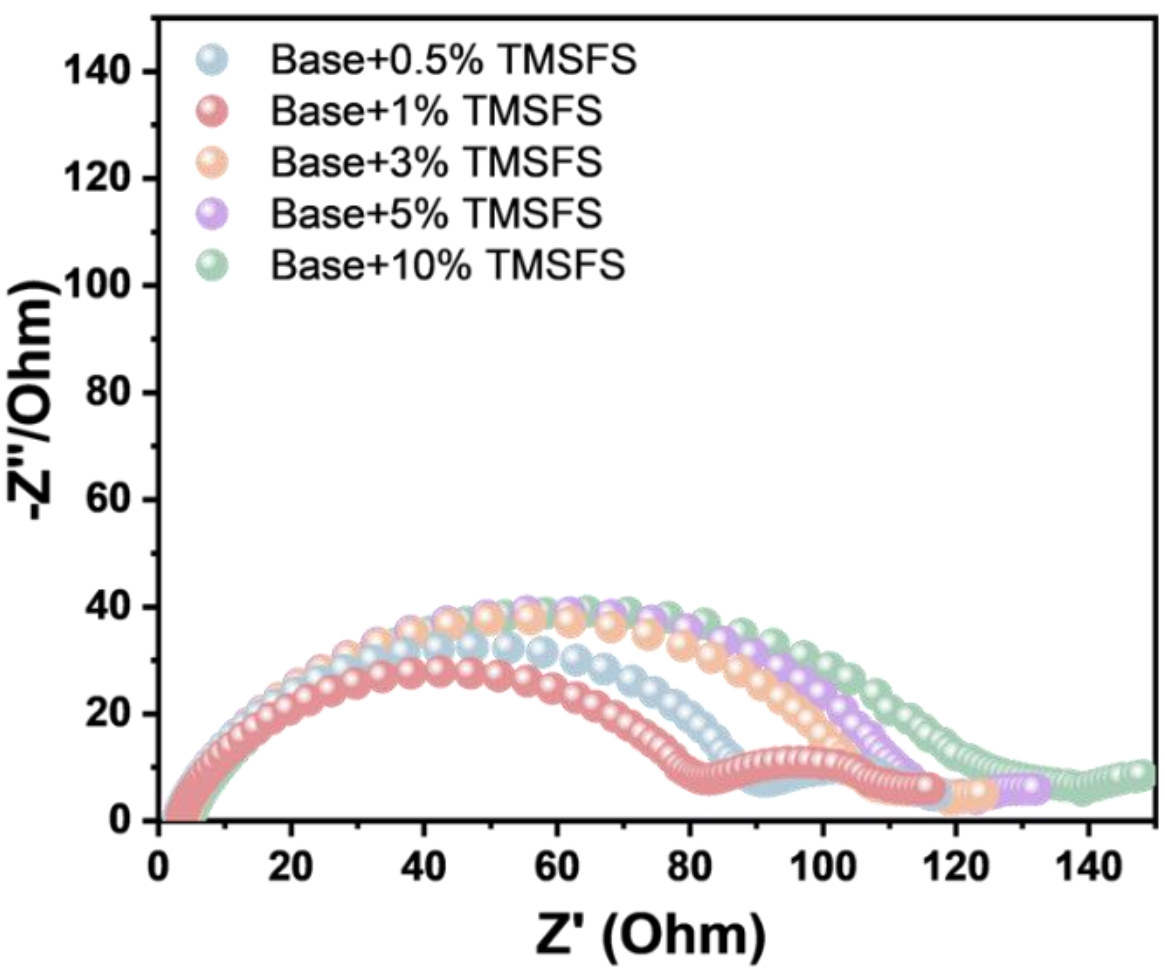

Fig. R20. EIS spectra measurements of cells after activation assembled by BE+ different amounts of TMSFS.

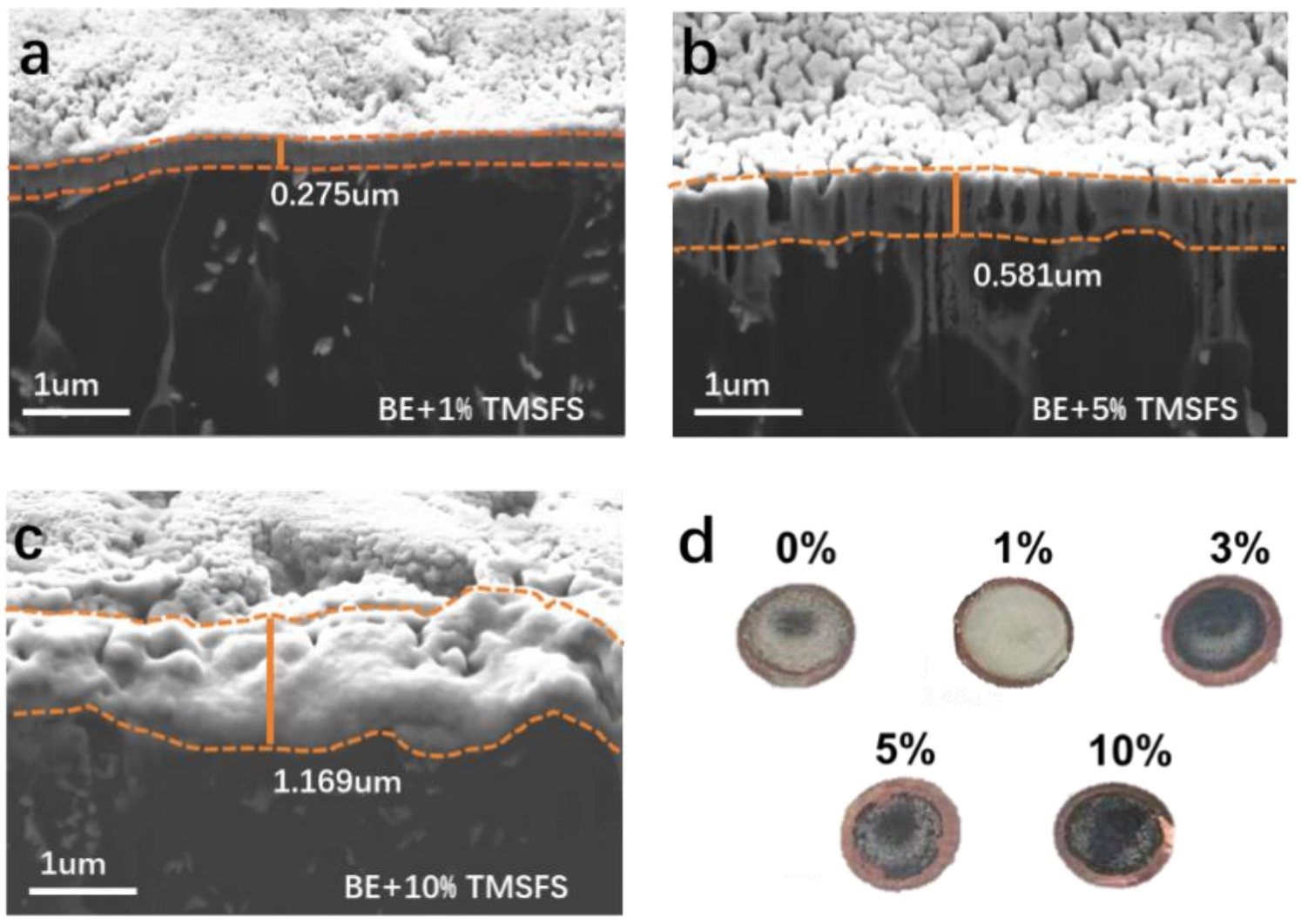

Fig. R21. Cross-section FIB-SEM images of deposited Li on Cu foil after several cycles of (a) BE+1wt.% TMSFS (TFFE)-, (b) BE+5wt.% TMSFS, (c) BE+10wt.% TMSFS and (d) corresponding shots of surface states.

Also, Li||LRMO full cells were assembled to test the cycling stability of different addition systems. 1wt.% addition of TMSFS system showed the most favorable cycling stability and capacity retention (Fig. R22).

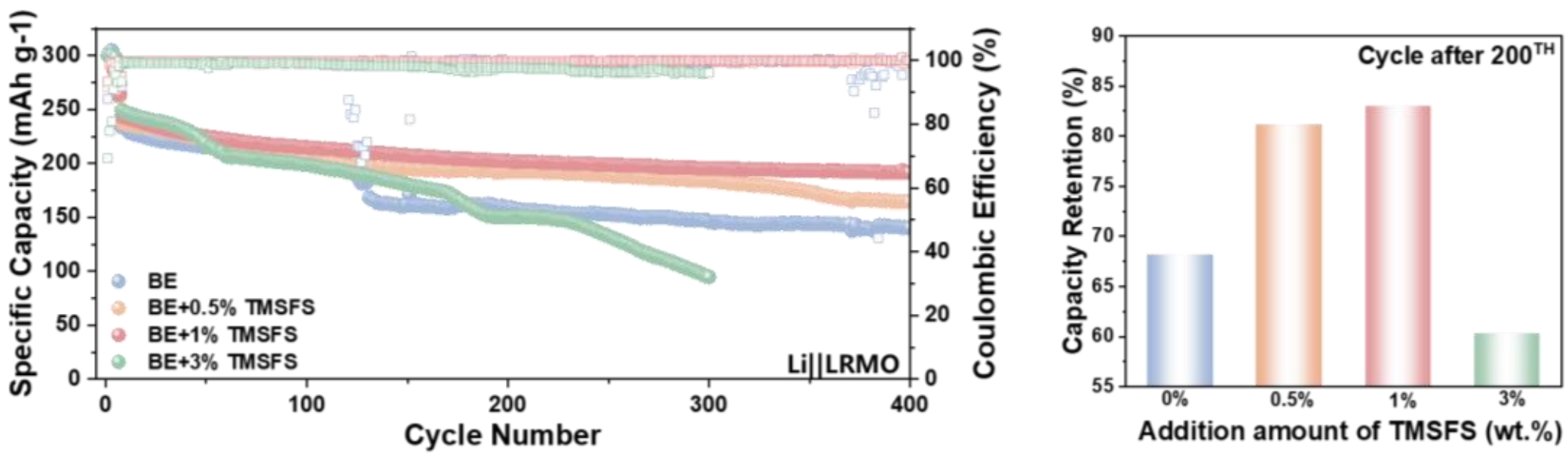

Fig. R22. (a) Li||LRMO Full coin cells assembled with different content of TMSFS and (b)corresponding capacity retention after 200 cycles.

These results demonstrate that 1 wt.% TMSFS represents an optimal balance between effective interfacial regulation and additive stability.

In the revised manuscript, we have provided further discussion to clarify the dependence on the TMSFS amounts. The changes of the above aspects have been supplemented in the revised manuscript highlighted with yellow, which can also be found below:

Firstly, electrochemical performance tests were conducted with different addition of TMSFS. As shown in Supplementary Fig. S16, half-cells with 1 wt.% TMSFS exhibit the most stable cycling compared with the baseline electrolyte and other additive contents.

Cross-sectional FIB–SEM and surface images reveal that increasing the TMSFS concentration leads to a progressively thicker interfacial reaction layer on the Li metal surface (Supplementary Fig. S17), which is attributed to excessive parasitic reactions between TMSFS and Li metal, as reported previously.

Furthermore, full-cell test results with different additive contents indicated that the addition of 1 wt.% exhibited the optimal performance (Supplementary Fig. S31), which was caused by the fierce side reactions caused by excessive addition and the corresponding thicker and less conductive interface reaction layer.

Fig. R19 *were applied as Supplementary Figure 16.*

Fig. R20 *were applied as Supplementary Figure 18.*

Fig. R21 *were applied as Supplementary Figure 17.*

Fig. R22 *were applied as Supplementary Figure 31.*

5. Voltage profiles were not suggested, even if they provide important information to understand the effect of TMSFS. The authors should include it.

Response: Thanks for the reviewer's suggestion. We have provided additional voltage profile in the revised manuscript.

Firstly, average CE of BE- and TFFE-based Li||Cu half-cell over 50 cycles at 0.5 mA cm^{-2} and 2 mAh cm^{-2} (Fig. R23a) and voltage-areal capacity profiles during the nucleation process for BE- and TFFE-based half-cells (Fig. R23b) have been provided. Besides, we have provided additional voltage profile in the revised manuscript. According to Fig. R23c, the voltage profiles show that the cell assembled with TFFE cycles stably for 50 cycles with a reduced polarization of less than 12 mV at current density of 0.5 mA cm^{-2} with a fixed areal capacity of 2 mAh cm^{-2}, whereas the counterpart using BE electrolyte exhibits significantly higher polarization exceeding 20 mV and fails around 50 cycles. Also, cycling stability of BE- and TFFE-assembled Li||Li symmetric cells at 1 mA cm^{-2} and 1 mAh cm^{-2} (Fig. R23d) and CV curves of BE- and TFFE-based Li||Cu half-cells at 1 mV s^{-1} (Fig. R23e) have been provided to help better understand the effect of TMSFS in electrochemical behavior and interfacial kinetics.

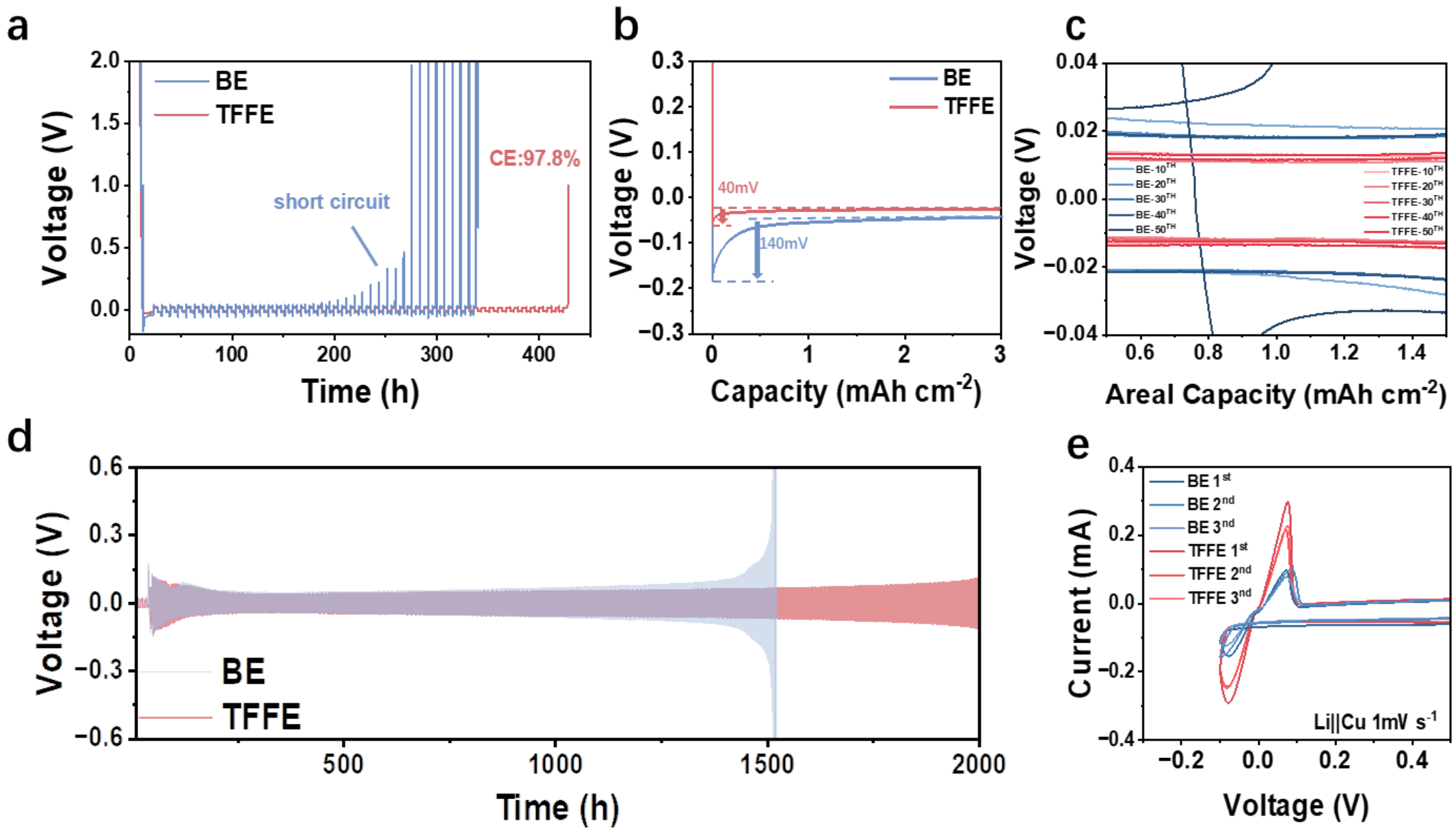

Fig. R23. (a) Average CE of BE- and TFFE-based Li||Cu half-cell over 50 cycles at 0.5 mA cm-2 and 2 mAh cm^{-2}; (b)Voltage–areal capacity profiles of TFFE and BE systems at 0.5 mA cm^{-2} and 2 mAh cm^{-2}, recorded at different cycle numbers (c) Voltage-areal capacity profiles during the nucleation process for BE- and TFFE-based half-cells;,; (d)Cycling stability of BE- and TFFE-assembled Li||Li symmetric cells at 1 mA cm^{-2} and 1 mAh cm^{-2}; (e)CV curves of BE- and TFFE-based Li||Cu half-cells at 1 mV s^{-1}.

Besides, Charge/discharge curves of coin cells assembled with TFFE and BE (Fig. R24) and dQ/dV-Voltage of Li||LRMO full cell from 10-100 cycles of TFFE and BE system (Fig. R25) have been shown to evaluate the impact of ACSS on high-voltage LMB performance.

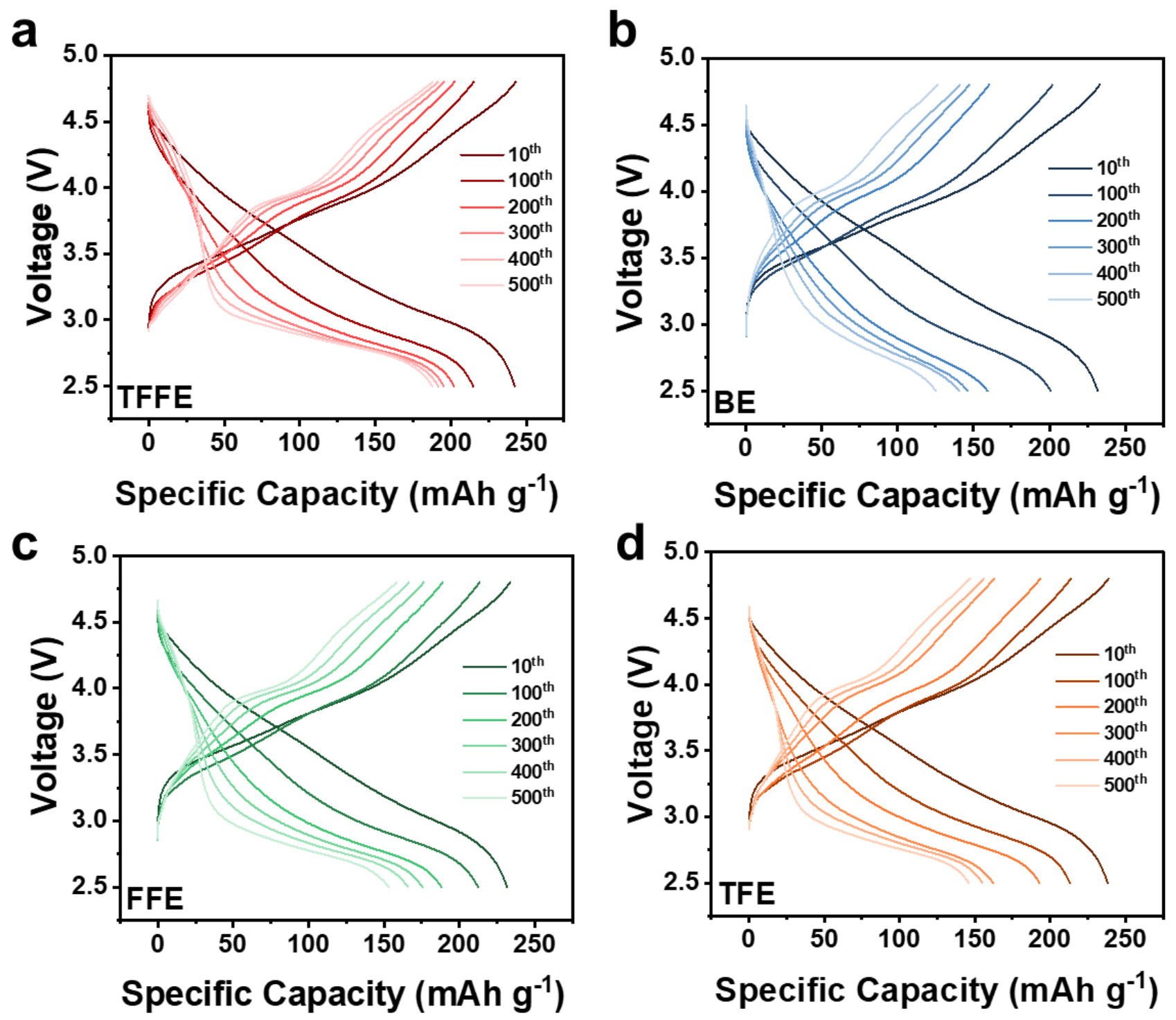

Fig. R24. (a) Charge/discharge curves of coin cells assembled with (a)TFFE, (b)BE, (c)FFE and (d)TFE from 2.5V to 4.8V under different cycles, respectively.

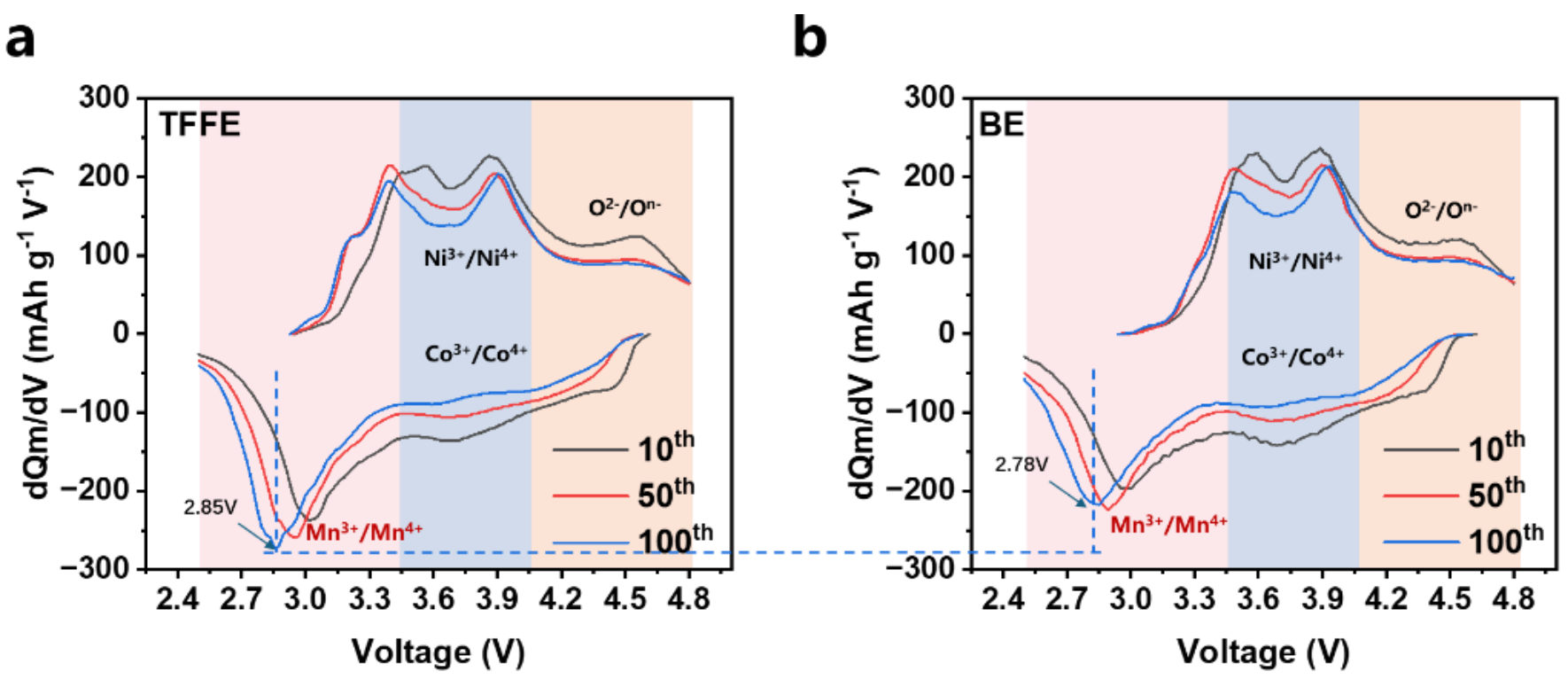

Fig. R25. (a) dQ/dV-Voltage of Li||LRMO full cell from 10-100 cycles of (a)TFFE and (b) BE system.

In the revised manuscript, we have provided the voltage profile and related discussion.

The changes of the above aspects have been supplemented in the revised manuscript highlighted with yellow, which can also be found below:

The voltage profiles also demonstrate that the TFFE-based cell maintained stable cycling with low polarization (<12 mV), whereas the BE-based cell exhibits higher polarization (>20 mV) and failed at approximately 50 cycles (Fig. 4c).

Fig. R23 *were applied as Fig. 4a, b, c, e and Supplementary Figure 21.*

Fig. R24 *were applied as Supplementary Figure 30.*

Fig. R25 *were applied as Supplementary Fig. 34*

6. One of the ambiguous points is whether Li_2S is a good component for a stable SEI layer on the Li anode? Is it a good ionic conductor?

Response: Thanks for the reviewer's suggestion for confirming the effect of Li_2S. Here are some references supporting Li_2S is a good component for a stable SEI and a good ionic conductor. It is reported that Li_2S demonstrates a good ionic conductivity of $\approx 10^{-5}$ S cm^{-1} and poor electronic conductivity of $\approx 10^{-13}$ S cm^{-1}, which implies that Li ions can easily migrate through Li_2S while electrons are prohibited.[19,20] Moreover, Li_2S is known to exhibit excellent chemical stability against Li metal and is less soluble than many organic SEI components[21], which helps improve long-term SEI robustness. Several theoretical and experimental studies have shown that Li_2S-containing SEI phases can reduce parasitic reactions and inhibit dendritic Li growth through their mechanical softness and high interfacial stability. In our work, Li_2S is one component within a mixed inorganic SEI (LiF/Li_2O), contributing to maintaining structural integrity, resisting continuous electrolyte decomposition and uniform lithium deposition during extended cycling.

In the revised manuscript, we have provided the advantage of Li_2S. The changes of the above aspects have been supplemented in the revised manuscript highlighted with yellow, which can also be found below:

To elucidate the origin of enhanced interfacial Li^+ transport kinetics, surface X-ray photoelectron spectroscopy (XPS) was first employed to analyze the concrete composition of SEI. The Li 1s and C1s spectra results demonstrated that TMSFS facilitated the formation of a multi-component and inorganic-rich SEI dominated by Li_2S, LiF, and Li_2O (Supplementary Fig. S25a and 25b).

7. Lack of basic properties depending on the amount of TMSFS should be provided.

Response: Thanks for the reviewer's suggestion. We have systematically provided some basic properties.

Firstly, we conducted Raman tests for the electrolyte with a series of gradient content

of TMSFS (0, 1, 5wt.%). As shown in the Fig. R26, increasing the additive content leads to a more pronounced solvation structure reconstruction, confirming that the additive is able to partially replace solvent molecules and anions in the first solvation shell of Li^+. Specifically, as the TMSFS content increased from 0 to 5 wt.%, the coordination fractions of FEC, PF_6^-, and EMC decreased progressively, from 0.369, 0.397, and 0.487 (0 wt.%) to 0.298, 0.345, and 0.416 (1 wt.%), and further to 0.231, 0.265, and 0.386 (5 wt.%), respectively.

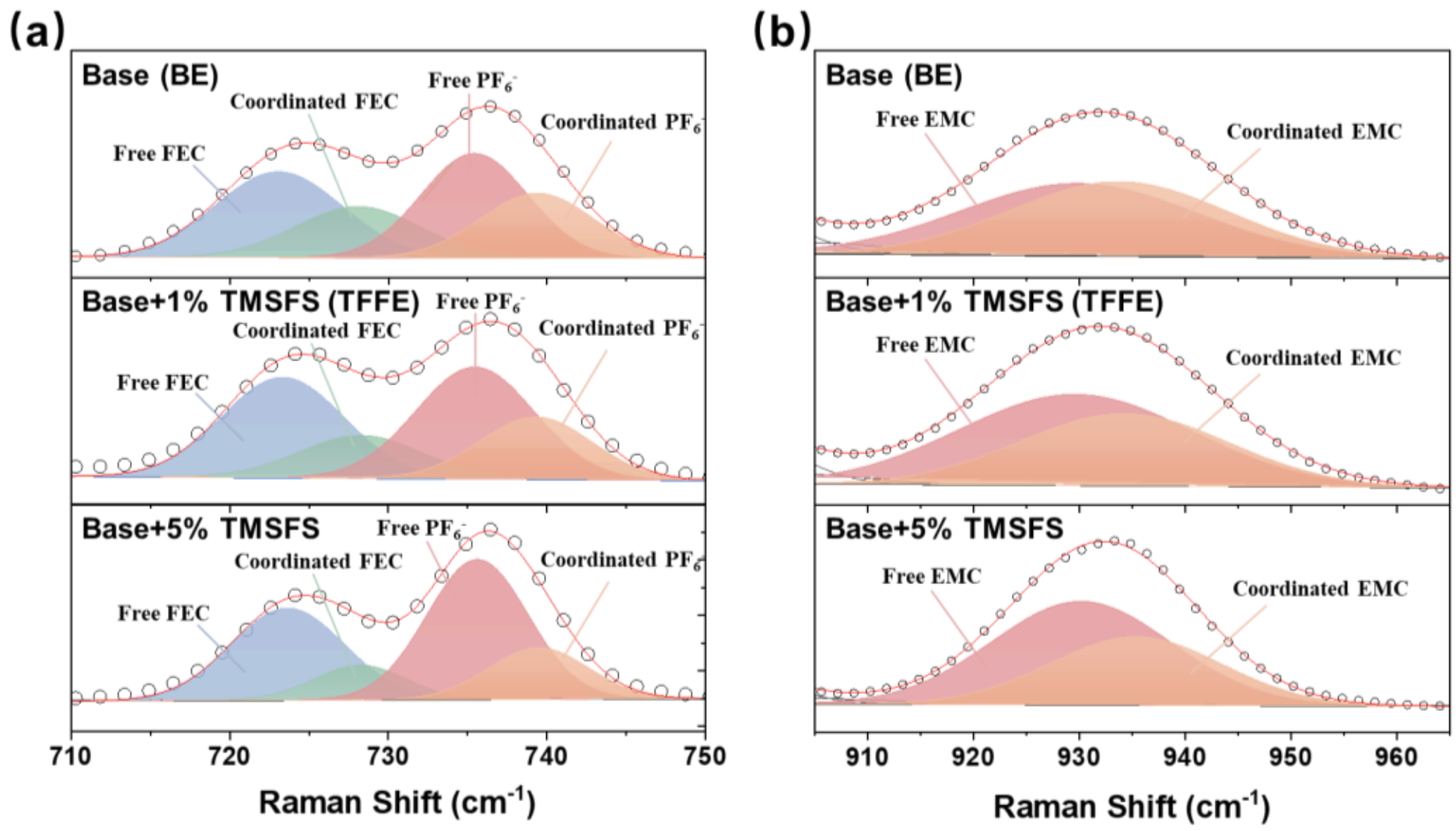

Fig. R26. (a) Raman spectra of BE and BE+1 wt. %, 5 wt.% from (a)710 to 750 cm^{-1} and (b)905 to 965 cm^{-1}.

NMR tests with higher additive concentrations were also conducted to examine the trend in solvation structure changes. In our previous measurements, the use of deuterated DMSO as a solvent led to its partial dissolution into the electrolyte, which interfered with the NMR results. In the present study, a coaxial NMR tube configuration was employed to eliminate interference from the deuterated solvent.[8] The ^7Li chemical shift displaced to lower values from -0.56 ppm (BE) to -0.58 ppm after adding 1 wt.% TMSFS (TFFE) and further shift to -0.64 as the addition amount increase to 5 wt.% (Fig. R27). The upfield shift implies the increased shielding effect on the Li^+ nucleus, resulting from the weakened interaction between Li^+ with anions and solvents.

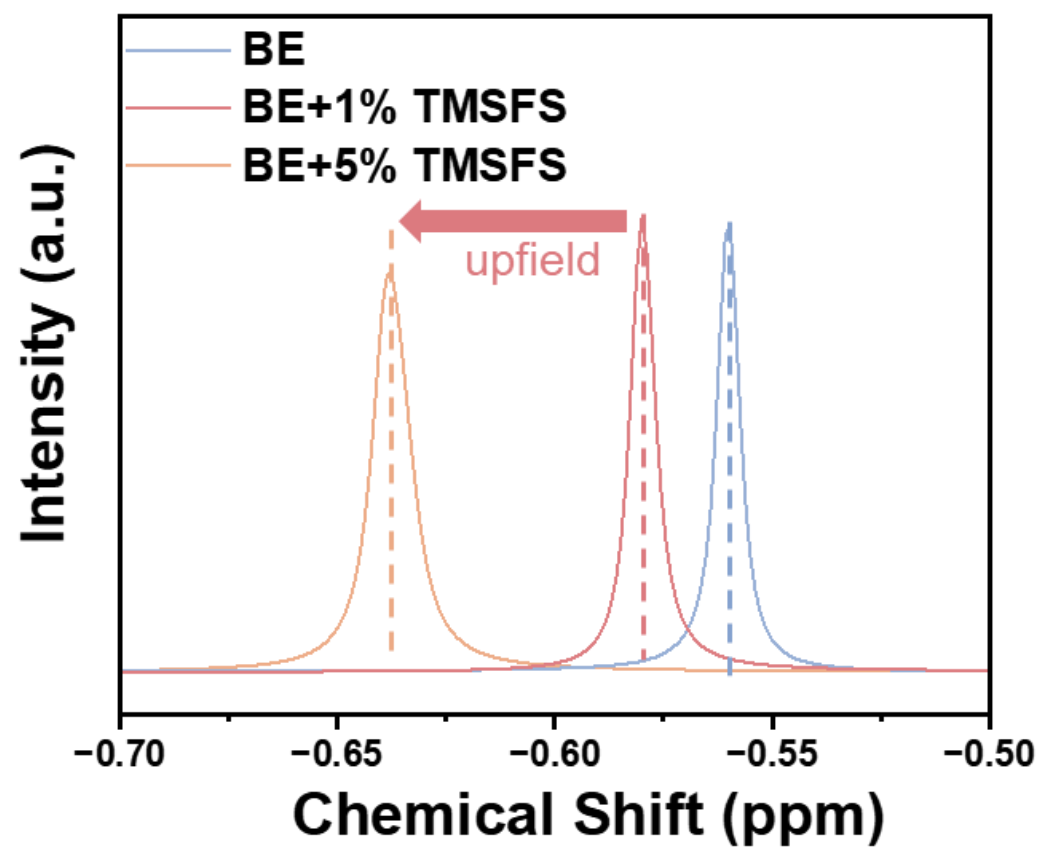

Fig. R27. ^{7}Li NMR spectra of BE and BE+1 wt.%, 5 wt.%.

In the revised manuscript, we have provided and discussed the basic properties depending on the amount of TMSFS. The changes of the above aspects have been supplemented in the revised manuscript highlighted with yellow, which can also be found below:

As the TMSFS content increased from 0 to 5 wt.%, the coordination fractions of FEC, PF_6^-, and EMC decreased progressively, from 0.369, 0.397, and 0.487 (0 wt.%) to 0.298, 0.345, and 0.416 (1 wt.%), and further to 0.231, 0.265, and 0.386 (5 wt.%), respectively (Supplementary Figs. S5 and S6), indicating that TMSFS effectively reduced the presence of solvent molecules and anions within the solvation sheath.

Also, the effect of TMSFS additive in altering the Li^+ solvation structure was further confirmed by ^{7}Li NMR (Supplementary Figs. S10), where the ^{7}Li chemical shift displaced to lower values from -0.56 ppm (BE) to -0.58 ppm after adding 1 wt.% TMSFS and further shift to -0.64 ppm as the addition amount increase to 5 wt.%. The upfield shift implies the increased shielding effect on the Li^+ nucleus, resulting from the weakened interaction between Li^+ with anions[29].

Fig. R26 *were applied as Supplementary Figure 5.*

Fig. R27 *were applied as Supplementary Figure 10.*

Reviewer: 3

Comments:

The authors have systematically investigated a new electrolyte design—an additive-coordinated solvation structure (ACSS) of Li^+—which preferentially sacrifices the

additive rather than anions to enable stable cycling of 600 Wh kg^{-1} lithium metal batteries (LMBs).

They discovered that by leveraging the electron push–pull effect among functional groups within the trimethylsilyl-2,2-difluoro-2-(fluorosulfonyl) acetate (TMSFS) molecule, even trace additive levels allow TMSFS to coordinate within the inner sheath of the Li^+ solvation structure, reducing the participation of ester solvent molecules and anions. This suppresses excessive anion and solvent consumption, while facilitating the formation of robust protective interphases on both cathode and anode surfaces, thereby enhancing high-voltage stability and Li-metal compatibility.

Overall, this work provides valuable insight into electrolyte design strategies for high-Ni cathodes and high-energy-density LMBs. After some minor revisions, I would recommend this manuscript for publication in Nature Communications.

Comments and Suggestions

1. As the authors described, uniformly distributed Li nuclei with compact and homogeneous morphology were observed in the TFFE system. Typically, a moderate CEI thickness (5–10 nm) offers sufficient chemical and mechanical protection for high-voltage systems. It is therefore interesting that the TFFE system exhibits a CEI thickness of only 2.7 nm, yet provides effective coverage without leading to surface cracking or continuous side reactions. The authors are encouraged to provide additional characterization or discussion to clarify the stability mechanism of such a thin CEI.

Response: Thanks for the reviewer's comment. We fully agree that accurate identification of the CEI layer is essential for understanding its protective function. The structure previously shown in Fig. 5b, d was indeed not the CEI, but rather the surface reconstruction region associated with TM migration during cycling. We are sorry for this mistake and recognize that its appearance may have led to confusion regarding the actual CEI thickness.

To address this issue, we conducted additional TEM measurements, specifically focusing on the electrode/electrolyte interface to visualize the true CEI. The newly acquired TEM images (now provided in the Fig. R28) clearly show a continuous and

uniform CEI layer with a thickness of ~6.11 nm in the TFFE system, which falls within the commonly reported range (5–10 nm) for high-voltage cathode systems, while BE system shows an uneven and much thicker CEI with a thickness over 14 nm. These results confirm that the improved interfacial stability arises from the formation of a moderately thick, mechanically robust CEI of TFFE.

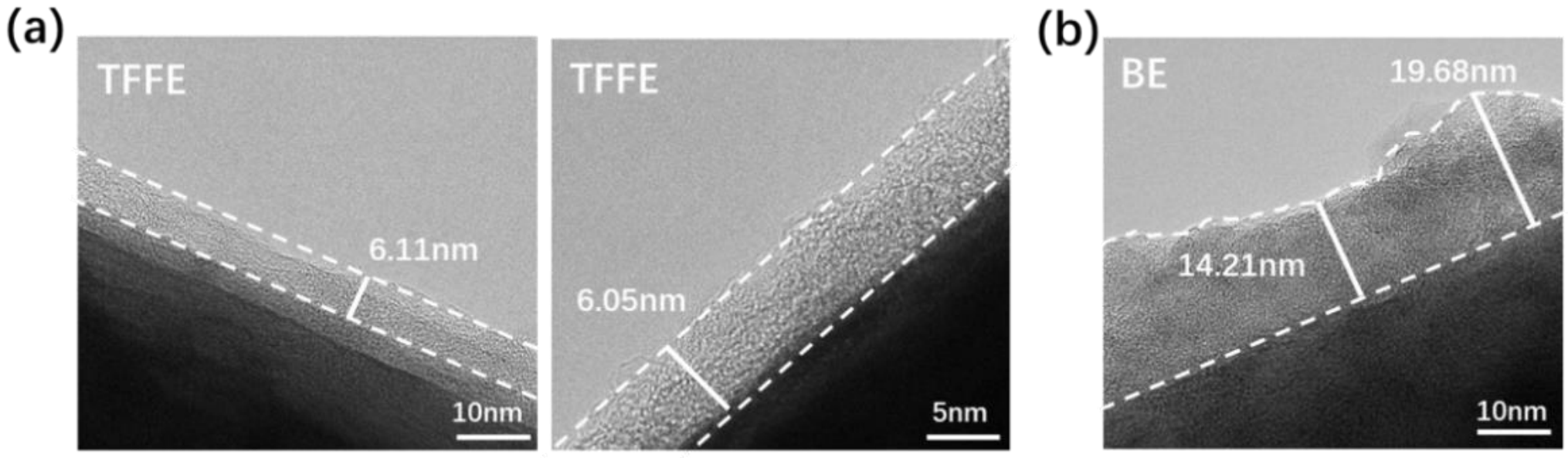

Fig. R28. TEM images of the CEI layer from Li||LRMO full cell assembled with (a)TFFE and (b)BE after 100 cycles.

Moreover, the LRMO particles displayed minimal structural damage and surface defects after 100 cycles in the TFFE electrolyte (Fig. R29a). TEM analysis and Fast Fourier Transform (FFT) patterns showed a well retained original layered structure with only a 2.7-nm-thick subsurface region undergoing phase transformation to the rock-salt structure, while the bulk interior kept its pristine layered phase structure (Fig. R29b). In sharp contrast, serious cracks could be observed on the LRMO particle in BE electrolyte, indicating phase degradation and a structural transition from a layered to a rock-salt configuration within a near-surface region of 9.8 nm in thickness ((Fig. R29c and d).

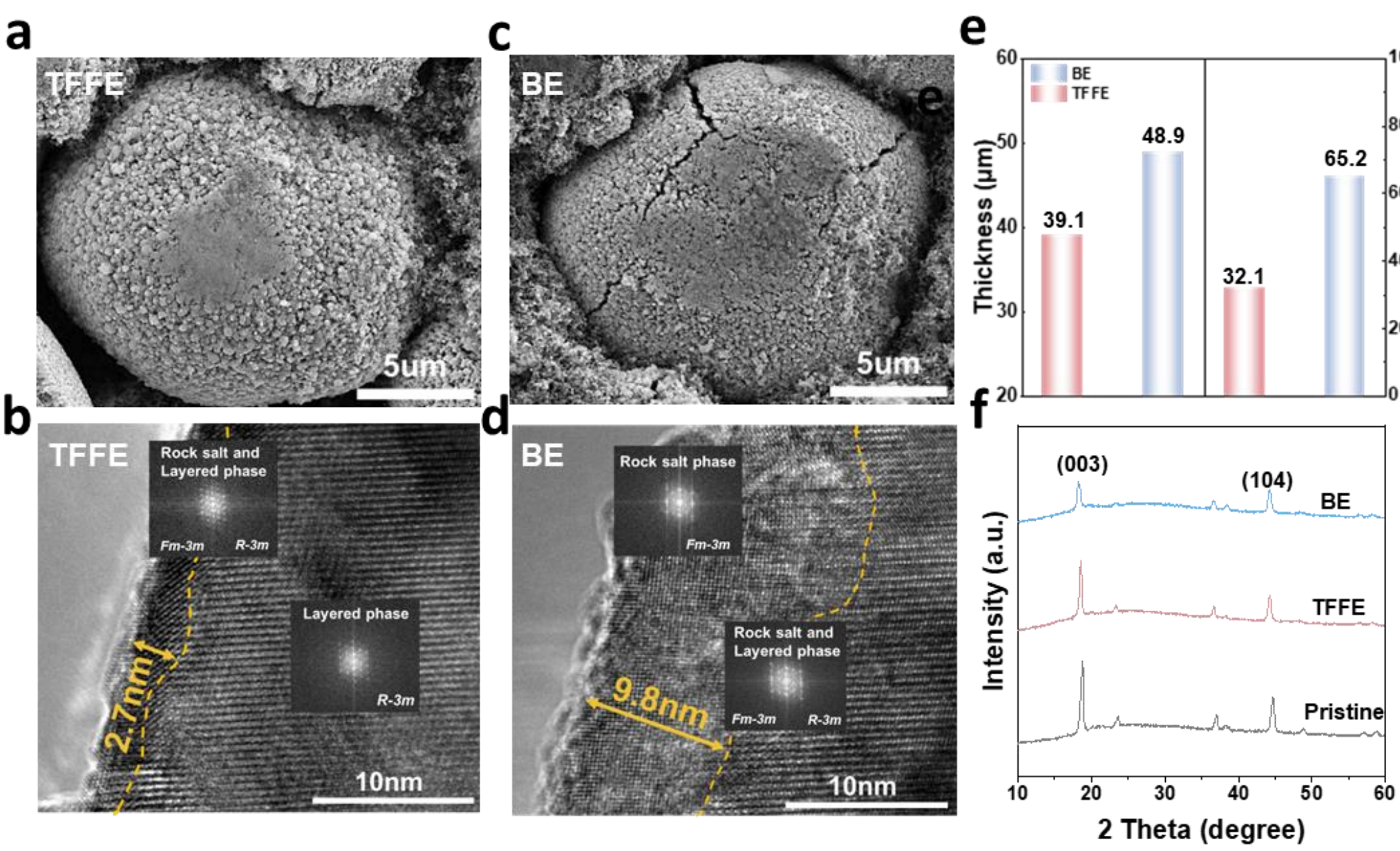

Fig. R29. (a) SEM image of the cathode surface from Li||LRMO full cell assembled with TFFE after 100 cycles; (b) TEM image of the cathode surface from Li||LRMO full cell assembled with TFFE after 100 cycles; (c) SEM image of the BE counterpart; (d) TEM image of the BE counterpart; (e) Thickness variation of the cathode after 100 cycles; (f) XRD patterns of fresh LRMOs and LRMOs after 100 cycled with BE and TFFE electrolytes.

The cross-section SEM images revealed that the thickness of the cathode cycled in TFFE increases from 29.6 μm to 39.1 μm, while the thickness of the cathode in BE expands dramatically to 48.9 μm (Fig. R29e). To further validate the cathode-stabilizing efficacy of ACSS, X-ray diffraction (XRD) analysis was conducted. As illustrated in Fig. R29f, the I (003)/I (104) ratios for pristine LRMO, cycled LRMO from TFFE-based cell and LRMO from BE-based cell are 1.522, 1.469, and 1.149, respectively, suggesting that TFFE mitigated the structural degradation of the cathode during cycling process. Conversely, the (003) peak of the BE-based sample shifted to a lower 2θ angle compared to the pristine material, accompanied by peak splitting into two distinct reflections, which indicated the severe irreversible phase transition.

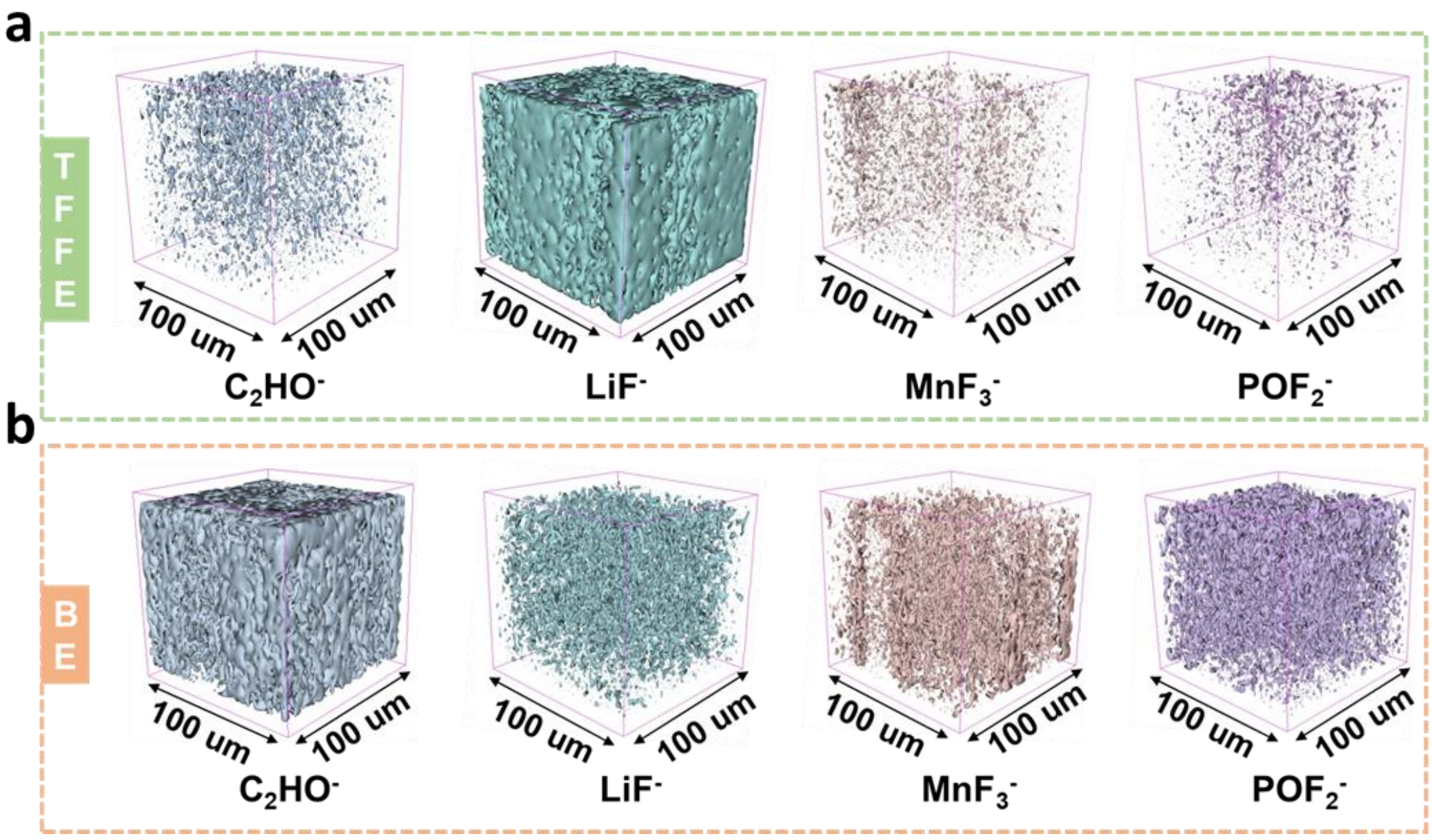

Fig. R30. 3D images of sputtering time-dependent of selected secondary ion fragments of different CEI (g) TFFE, (h) BE.

Depth sputtering ToF-SIMS was performed to investigate the role of ACSS in the formation of the CEI. As shown in Fig. R30, less C_2HO^- organic and rich inorganic species, such as LiF^-, $SiOC_2H_6^-$ and SO_3^-, were uniformly dispersed across the CEI layer of TFFE. Meanwhile, MnF_3^-(from active mass dissolution products) and POF_2^-(from $LiPF_6$ decomposition) were significantly reduced compared to BE system, indicating the protective effect of the TMSFS on the cathode and the reduction of lithium salt consumption at the interface. These results indicated that the ACSS effectively suppressed the decomposition of lithium salts and solvents on the interface of electrode, and the resulting ACSS derived-CEI contributed to preserve the structural integrity of cathode during extended cycling under high-voltage conditions.

In the revised manuscript, we have provided the CEI thickness information and the characterization of CEI. The changes of the above aspects have been supplemented in the revised manuscript highlighted with yellow, which can also be found below:

TEM analysis and Fast Fourier Transform (FFT) patterns showed a continuous and uniform CEI layer with a thickness of ~6.11 nm was spotted and that LRMO particles well retained their original layered structure with only a 2.7-nm-thick subsurface region undergoing phase transformation to the rock-salt structure, while the bulk interior kept its pristine layered phase structure (Fig. 3a and 3b). In sharp contrast, serious cracks

could be observed on the LRMO particle in BE electrolyte (Supplementary Fig. S12b), and BE system showed an uneven and much thicker CEI with a thickness over 14 nm (Fig. 3c), indicating phase degradation and a structural transition from a layered to a rock-salt configuration within a near-surface region of 9.8 nm in thickness (Fig. 3d).

***Fig. R28** were applied as Fig. 3a and c.*

***Fig. R29** were applied as Supplementary Figure 12 and Fig. 3b, d, e and f.*

***Fig. R30** were applied as Fig. 3g and h.*

2. The 0.1 C energy densities reported for the Li-metal pouch cells are recommended to be revised to cycled energy densities, as this would more accurately reflect practical performance.

Response: Thanks for the reviewer's suggestion. In response, while retaining the original energy-density calculation method, we have added additional energy-density values derived from different discharge rates to provide a more comprehensive reference. Specifically, the energy densities calculated from the first-cycle discharge at 0.1 C are 516.4 and 550.7 Wh kg^{-1}, whereas those calculated from the first-cycle discharge at 0.5 C are 485.2 and 514.1 Wh kg^{-1} (Fig. R31). These data have now been included in the main text and figure.

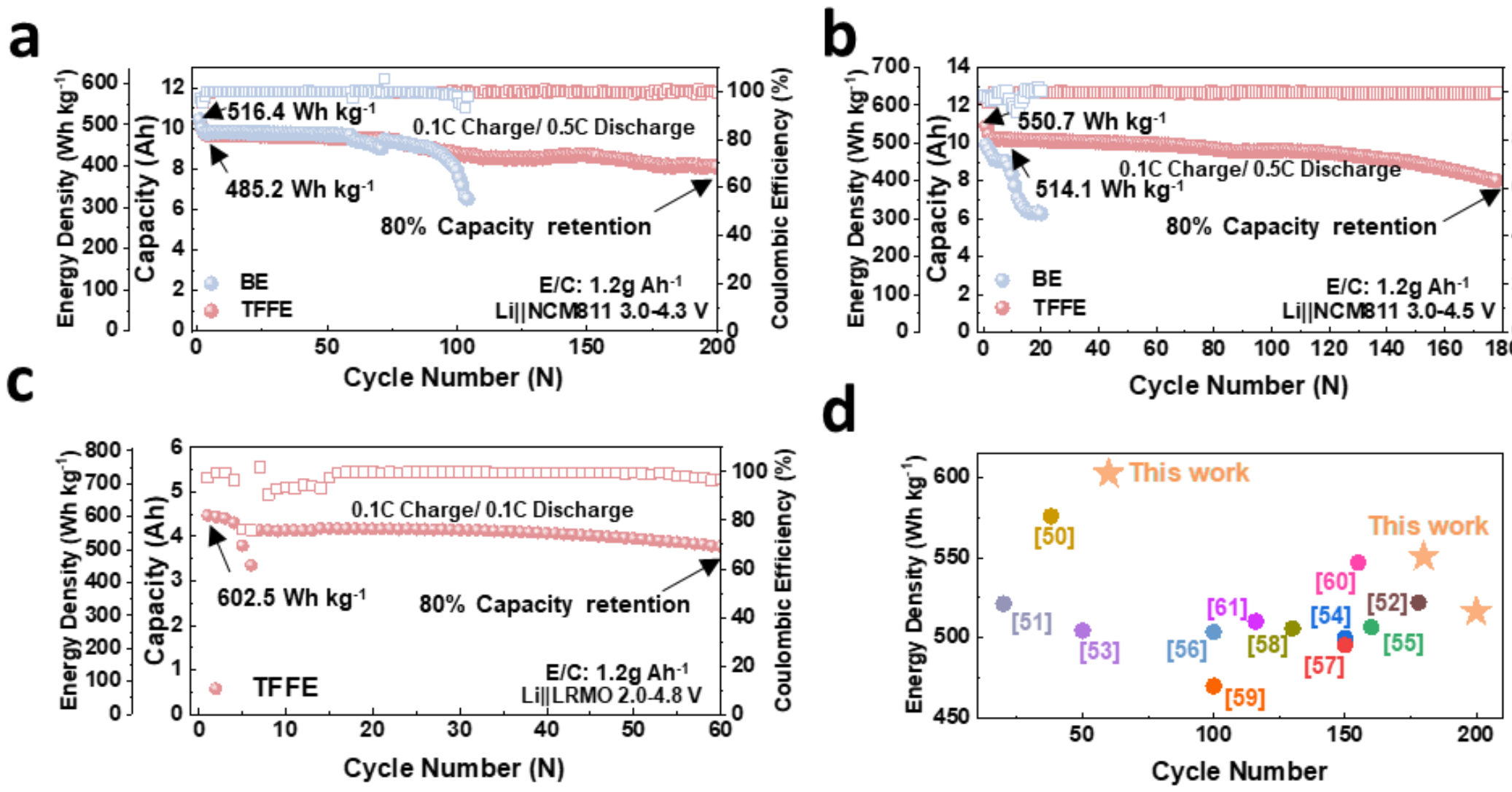

Fig. R31. Cycling performance comparison of Li||NCM811 pouch cells assembled with BE and TFFE electrolytes (a) from 3.0 to 4.3 V (b) from 3.0 to 4.5V; (c) Cycling performance of Li||LRMO pouch cells assembled with TFFE electrolytes from 2.0 to

4.8 V; (d) Energy density and cycle life benchmarking of the LMB pouch cell in this work compared to recently reported advanced electrolyte systems (Energy density was calculated from the first-cycle discharge at 0.1 C).

The changes of the above aspects have been supplemented in the revised manuscript highlighted with yellow, which can also be found below:

***Fig. R31** were applied as Fig. 6b, c, d and e.*

3. Please indicate the rate in Supplementary Figure 19. It is particularly interesting that the TFFE and BE systems only diverge at the highest rate. Why are the pouch cells not tested under the same rate and voltage window as the coin cells? A brief explanation or justification would improve the clarity of the data comparison.

Response: Thanks for the reviewer's suggestion. We have provided the specific rate information in the figure S19, now Fig. R32.

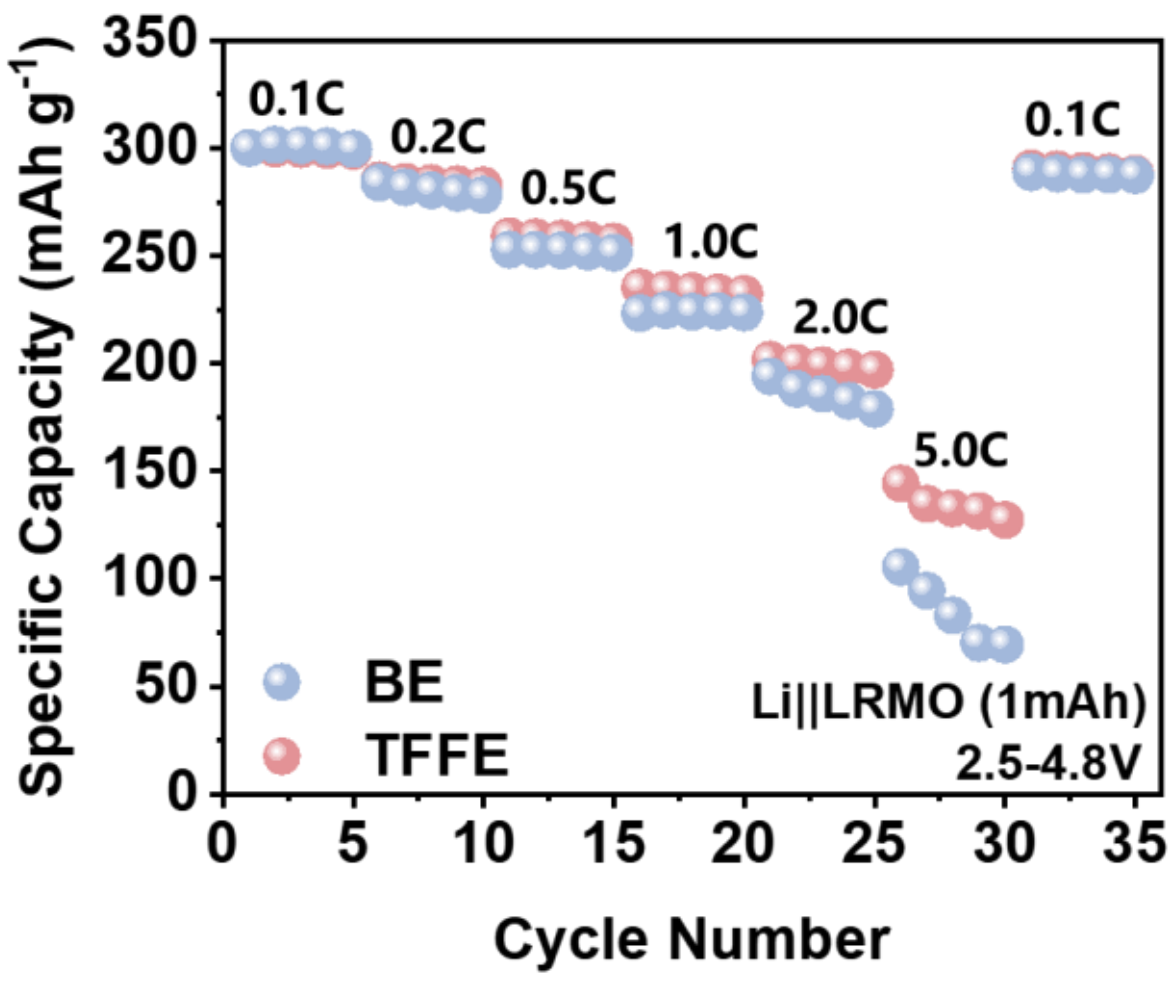

Fig. R32. Rate capability of Li||LRMO full cells assembled with BE and TFFE electrolyte.

Firstly, to achieve a higher energy density, the pouch cell was discharged down to 2.0 V, rather than 2.5V in coin cells. Aligning with established research and our experimental data (Table R7), the standard cycling condition for pouch cells is typically set below 0.5 C. The rationale lies in their constrained ability to manage the increased thermal and electrochemical stress, which manifests as accelerated degradation mechanisms, including lithium plating and active material cracking, ultimately compromising cycle life and safety. This issue is further exacerbated in high energy density pouch cells, which are typically tested under lower stack pressure (~0.2 MPa)

and higher areal capacity anode (>4 mAh cm^{-2}, usually 5 times higher than that of coin cell) than coin cells' testing situation, making them more susceptible to lithium dendrite growth and associated safety risks. Consequently, pouch-cell evaluations are commonly conducted at modest rate. Thus, operating within this moderate rate window is essential for achieving the durability expected from pouch cell designs.[22-32]

Table R7. The comparison of pouch cell performance with studies on High Density LMB.

Anode	Cathode	Energy desity (Wh kg^{-1})	Capa-city (Ah)	Current density (C)	Cycling performance	Ref
**Li**	NCM811	550.7	10.9	0.1/0.5	capacity retention of 80% after 180 cycles	This work
**Li**	NCM811	516.4	10.4	0.1/0.5	capacity retention of 80% after 200 cycles	This work
**Li**	LRMO	602.5	4.5	0.1/0.1	capacity retention of 80% after 60 cycles	This work
**Li**	AZ0.3SNCM	504.5	10.83	0.1/0.3	capacity retention of 73.7% after 50 cycles	22
**Li**	LNMO	505.9	19	0.1/0.1	capacity retention of 91% after 130 cycles	25
**Li**	LRMO	576	9	0.1/0.2	capacity retention of 84.4% after 38 cycles	26
**Li**	NCM811	495.5	5.97	0.1/0.3	capacity retention of 80% after 150 cycles	30

**Li**	NCM811	470	5.2	0.1/0.5	capacity retention of 81% after 100 cycles	31
**Li**	NCM811	503.7	7.3	0.1/0.5	capacity retention of 84.1% after 100 cycles	28
**Li**	NCM83	506.7	7	0.1/0.5	capacity retention of 80% after 160 cycles	24
**Li**	NCM83	521.3	39	0.1/0.1	stably cycle for 20 cycles	23
**Li**	NCM811	547	7.5	0.1/0.2	capacity retention of 79% after 155 cycles	27
**Li**	NCM811	510.3	5.6	0.1/0.25	capacity retention of 84% after 116 cycles	32
**Li**	NCM83	522	7.3	0.1/0.3	capacity retention of 80.9% after 178 cycles	29

To clarify, we evaluated the pouch cells under the same current densities as the coin cells. The current density of 0.1C for pouch cells is equal to 0.88 mA cm^{-2} and the current density of pouch cells at 0.5C is equal to the current density of coin cells at 5C. As is shown in Fig. R33, pouch cells were evaluated under different discharge rates, revealing that a moderate rate of 0.5C delivers the most reliable performance. In contrast, when the discharge rate was increased to 1C (8.88 mA cm^{-2}), the pouch cells exhibited almost no usable capacity. This behavior confirms that the pouch-cell configuration is intrinsically unsuitable for high-rate operation, owing to its much larger electrode area, higher areal capacity, and the resulting challenges in sustaining homogeneous and sufficiently fast Li-ion flux compared with coin-cell architectures. Therefore, for pouch-cell cycling we adopted a lower and practically relevant rate (0.1C

charge-0.5C discharge, and 0.1C charge-0.1C discharge), where the cells can operate without immediate dendritic failure. This allows a meaningful comparison of electrolyte effects under realistic pouch-cell conditions.

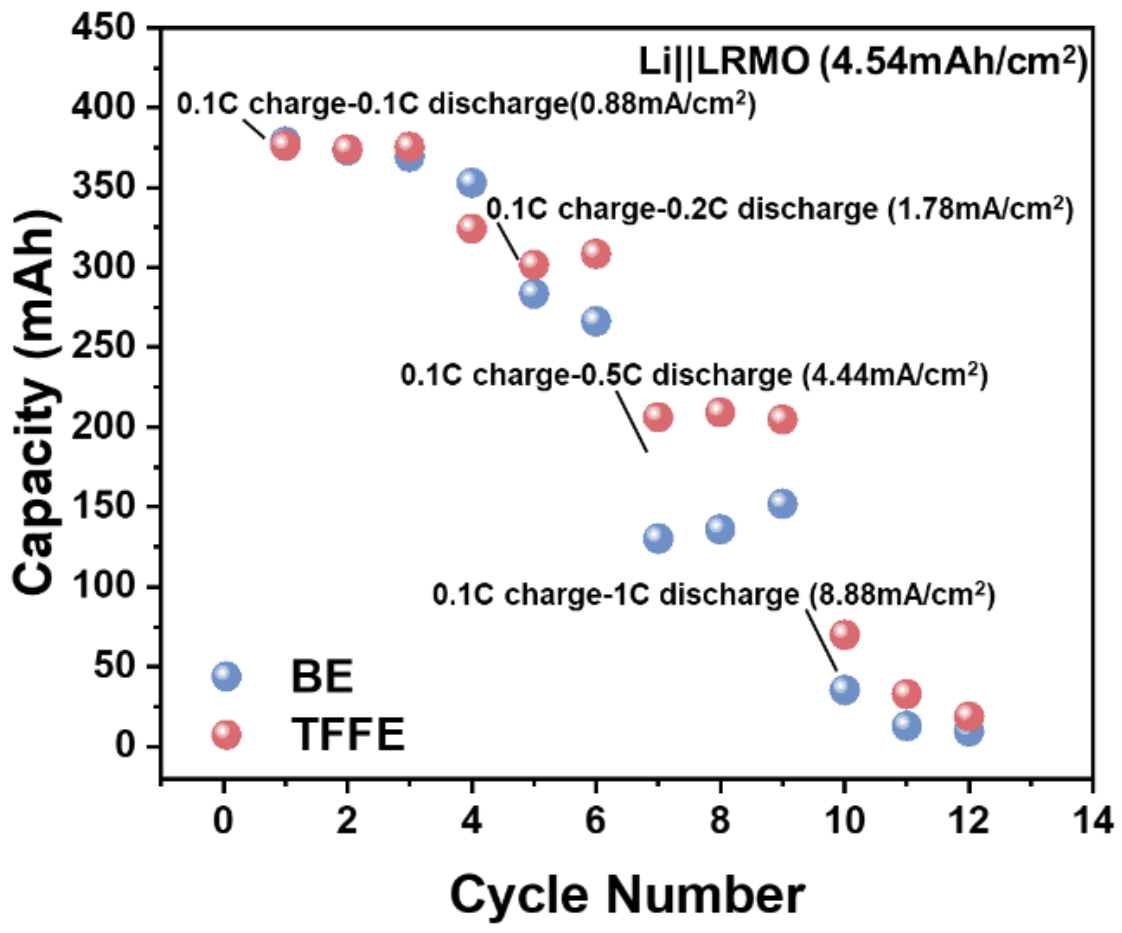

Fig. R33. Rate capability of Li||LRMO pouch cells assembled with BE and TFFE electrolyte.

In the revised manuscript, we have provided the CEI thickness information. The changes of the above aspects have been supplemented in the revised manuscript highlighted with yellow, which can also be found below:

Pouch cells were evaluated at different discharge rates, from which a moderate rate (<0.5C) was identified as the most suitable testing condition for reliable performance assessment (Supplementary Fig. S42).

Fig. R32 *were applied as Supplementary Figure 32.*

Fig. R33 *were applied as Supplementary Figure 42.*

Table R7 *were applied as Supplementary Table 6.*

Reviewer: 4

Comments:

This paper investigates the role of a designed additive in lithium metal batteries. The central rationale lies in that the electron-donating functional group (trimethylsiloxy) enhances the coordination between lithium and the additive, thereby forming a stable six-membered ring structure that facilitates the formation of robust SEI and CEI layers. The designed electrolyte demonstrates relatively good performance in lithium metal batteries. However, the paper suffers from two major issues.

1. First, the novelty of the design strategy is insufficient — the concept of tuning electron-withdrawing or electron-donating functional groups is rather common and fails to meet the journal's standard for innovation.

Response: We appreciate the reviewer's comment about the novelty of this work. We appreciate the reviewer's comment about the novelty of this work. For the novelty of this work, functional-group tuning is rather common, however, the additive strongly-coordinated solvation structure enabled by the molecular architecture of TMSFS is the key point, which fundamentally differs from previously reported electrolyte additives.

(1) Design of ACSS solvation structure.

Conventional organic ester-based electrolytes tend to form organic-rich interphases with poor passivation and mechanical robustness, leading to continuous electrolyte consumption and rapid degradation in high-voltage lithium-metal batteries (Fig. R34a). Although anion-dominated solvation strategies—such as high-concentration electrolytes, functional salts, and multiple additives—can promote inorganic-rich interphases, they inevitably suffer from increased viscosity, reduced ionic conductivity, and accelerated anion depletion (Fig. R34b). Moreover, most low-concentration additives (<5 wt.%) fail to enter the inner Li^+ solvation sheath due to insufficient Li^+-binding strength, resulting in low utilization efficiency. To address these intrinsic limitations, we propose an additive-coordinated solvation structure (ACSS) (Fig. R34c), in which a highly coordinating multifunctional additive is deliberately designed to integrate into the inner solvation sheath even at trace levels, enabling **efficient**

interphase formation while **minimizing solvent and anion consumption**, thereby simultaneously improving interfacial stability and cycling performance.

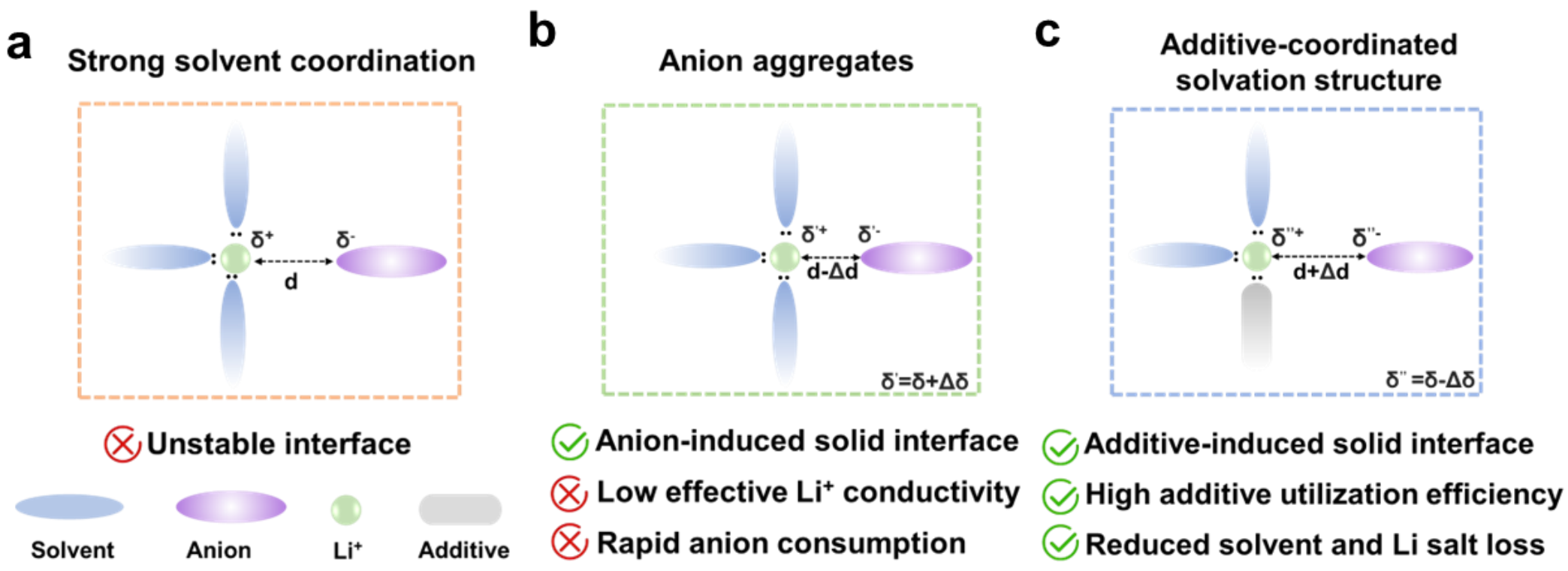

Fig. R34 Schematic illustrations of the solvation structure (a)strong solvent coordination (b)anion aggregates and (c)additive-coordinated solvation structure (ACSS).

(2) Molecular structures design of TMSFS.

Guided by key design principles—strengthening the binding affinity between the additive molecule and Li^+ to enhance additive utilization efficiency, we rationally designed the TMSFS molecule. As illustrated in Fig. R35, the strong affinity between TMSFS and Li^+ originates predominantly from two contributions: orbital energy and electrostatic energy. On the one hand, the –SO_2F–CHF_2–CO_2– fragment exhibits high polarizability and strong Li^+-coordinating capability. On the other hand, the trimethylsilyl group provides a strong electrostatic component, enriching negative charge at the carbonyl and sulfonyl oxygen atoms, leading to a strong Coulombic attraction between Li^+ and O. These factors synergistically enhance the intrinsic Li^+ binding affinity of TMSFS, rather than being achieved by adjusting the functional groups alone. Meanwhile, the F, S and Si containing species can be utilized to induce inorganic-rich interphases thereby synergistically enhancing the cycle life of the battery.

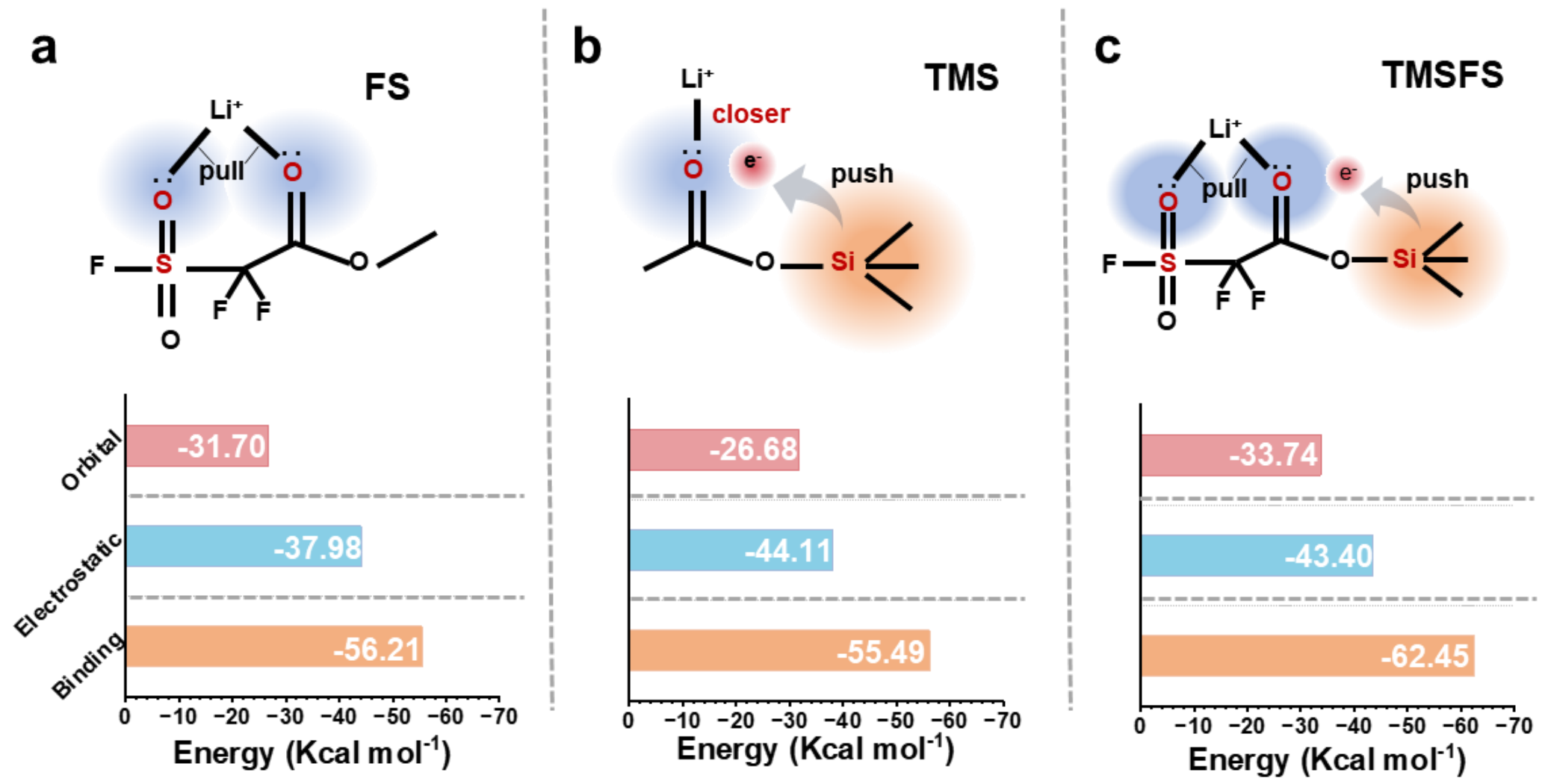

Fig. R35 The molecular structures and EDA (Energy Decomposition Analysis) maps roles of (d) FS, (e) TMS and (f) TMSFS molecules.

(3) Cycling performances enabled by ACSS.

In this work, the TFFE-based Li|| NCM811 pouch cell achieved a high energy density of 516.4 Wh kg^{-1} for over 200 cycles and 550.7 Wh kg^{-1} for 180 cycles with 80% capacity retention, while BE-based counterpart failed within 20 cycles (Fig. R36a and b). Also, a record-high reversible 602.5Wh kg^{-1} pouch cell paired with LRMO was assembled and demonstrated a stable cycling performance from 2.0V to 4.8 V exceeding 60 cycles with 80 % capacity retention (Fig. R36c). Compared to recently reported high energy density LMB pouch cell (≥450 Wh kg^{-1}), the TMSFS-formulated electrolyte demonstrated superior cycling stability, achieving ultrahigh specific energy and long-term stable cycling in LMB pouch cell (Fig. R36d). The results revealed superior performance in both energy density and cycle life, collectively highlighting the promising potential of ACSS electrolyte implemented through TMSFS additive for practical high-energy-density pouch cell applications.

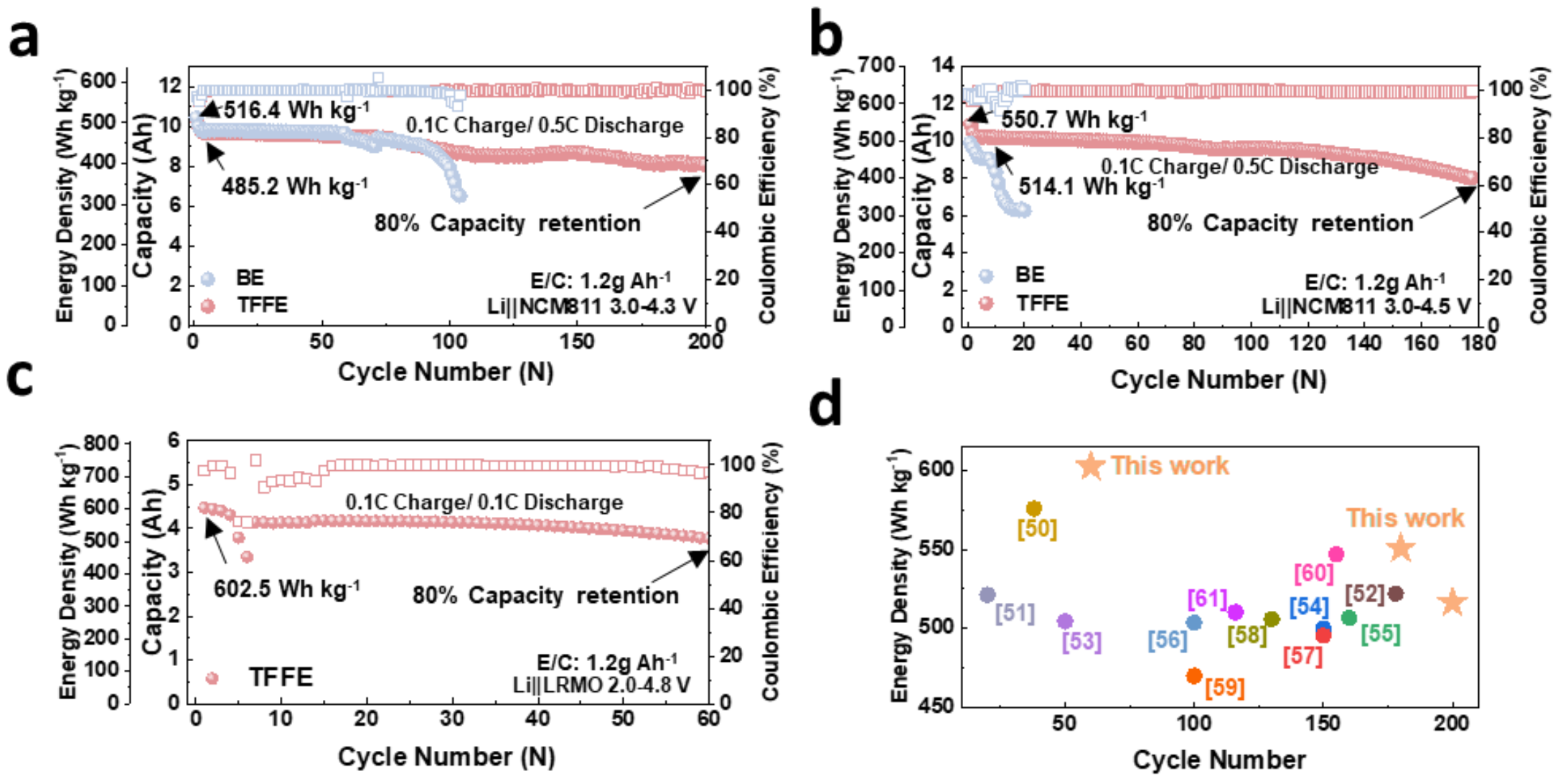

Fig. R36 Cycling performance comparison of Li||NCM811 pouch cells assembled with BE and TFFE electrolytes (a) from 3.0 to 4.3 V (b) from 3.0 to 4.5V; (c) Cycling performance of Li||LRMO pouch cells assembled with TFFE electrolytes from 2.0 to 4.8 V; (d) Energy density and cycle life benchmarking of the LMB pouch cell in this work compared to recently reported advanced electrolyte systems (Energy density was calculated from the first-cycle discharge at 0.1 C).

Together, these features demonstrate that the novelty of our study lies not in routine functional-group adjustment, but in the extreme high additive utilization efficiency and inorganic-rich interphases enabled by ACSS. We have revised the manuscript to better emphasize this mechanism-level innovation and to clearly distinguish our strategy from previous additive-design approaches.

In the revised manuscript, we have provided more characterizations to highlight the novelty of the design strategy. The changes of the above aspects have been supplemented in the revised manuscript highlighted with yellow, which can also be found below:

Electrostatic potential (ESP) maps reveal high electron density at the carbonyl oxygen (-22.52 kcal mol^{-1}) and sulfonyl oxygen atoms (-18.30 kcal mol^{-1}). This pronounced negative region exerts a strong pull effect on Li^+, enabling the formation of thermodynamically stable six-membered chelate rings. Energy decomposition analysis (EDA) reveals that the FS ligand exhibits substantial orbital interaction energy (-31.70 kcal mol^{-1}) and electrostatic interaction energy (-37.98 kcal mol^{-1}), collectively accounting for the overall Li^+–FS binding energy of -56.21 kcal mol^{-1}.

As shown in Fig. 1e and Supplementary Fig. S1b, the trimethylsilyl group of Trimethylsilylacetate (TMS) exerts a strong electron-pushing effect to the C=O, elevating its ESP to as low as -35.52 kcal mol^{-1}, which demonstrates the validity of this design. However, this type of structural configuration featuring a single C=O coordination is incapable of forming strong and stable coordination with Li^+ ions, which was confirmed by the low orbital (-26.68 kcal mol^{-1}) and binding energy (-55.49 kcal mol^{-1}), thereby limiting its ability to penetrate into the first solvation sheath.

The trimethylsilyl group exhibits strong electron-donating character, while the electron-withdrawing nature of the –CF_2–SO_2F– moiety concentrates negative charge on the sulfonyl and carbonyl oxygen atoms. These complementary electronic effects synergistically elevate both the orbital energy (-33.74 kcal mol^{-1}) and the electrostatic energy (-43.40 kcal mol^{-1}). As a result, TMSFS achieves the highest Li^+ binding energy among the studied reported candidates (-62.5 kcal mol^{-1}), fully satisfying the design criteria for the ACSS electrolyte.

***Fig. R34** were applied as Fig. 1a, b and c.*

***Fig. R35** were applied as Fig. 1d, e and f*

***Fig. R36** were applied as Fig. 6b, c, d and e.*

2. Second, the scientific rationale behind using trace additives to improve the stability of lithium metal batteries remains questionable. The core issue is that the SEI on lithium metal surfaces is renewed during each plating/stripping cycle, meaning that reactive trace additives have limited influence throughout the lithium lifecycle. This viewpoint has also been supported by previous work from Prof. J. R. Dahn (A. Eldesoky et al., J. Electrochem. Soc. 168, 120508 (2021)).

Response: Thanks for the reviewer's comment regarding the limited influence of trace additives on the continuously renewed SEI during Li plating/stripping. We agree that many highly reactive additives are rapidly consumed and may lose effectiveness after a few cycles, and cite the referenced work by Prof. Dahn. The effectiveness of trace additives in improving battery performance critically depends on initial film-forming state[33]. Thus, the present design aims to address this issue by enabling the additive to participate in the Li^+ solvation structure, thereby maximizing its interfacial film-

forming capability and constructing inorganic rich SEI layer, which ultimately leads to prolonged cycling life.

To elucidate the strong coordination effect of TMSFS, we employed internal-referenced diffusion-ordered spectroscopy (IR-DOSY), which is an effective technique to measure the coordination ratio of specific solvents in an electrolyte with more than one solvent [7]. The 2D ^{1}H NMR results reveal that upon the addition of the TMSFS, the as-calculated coordination ratio (α) of EMC and FEC decrease from 0.44 and 0.31 in BE to 0.39 and 0.28 in TFFE, respectively. Meanwhile, TMSFS exhibits a markedly high coordination fraction of 0.975, a more accurate descriptor of solvents' ability to solvate Li^+ than the commonly used dielectric constant. These results substantiate the strong Li^+-coordinating capability of TMSFS and its effectiveness in attenuating solvent–Li^+ coordination. (Fig. R37).

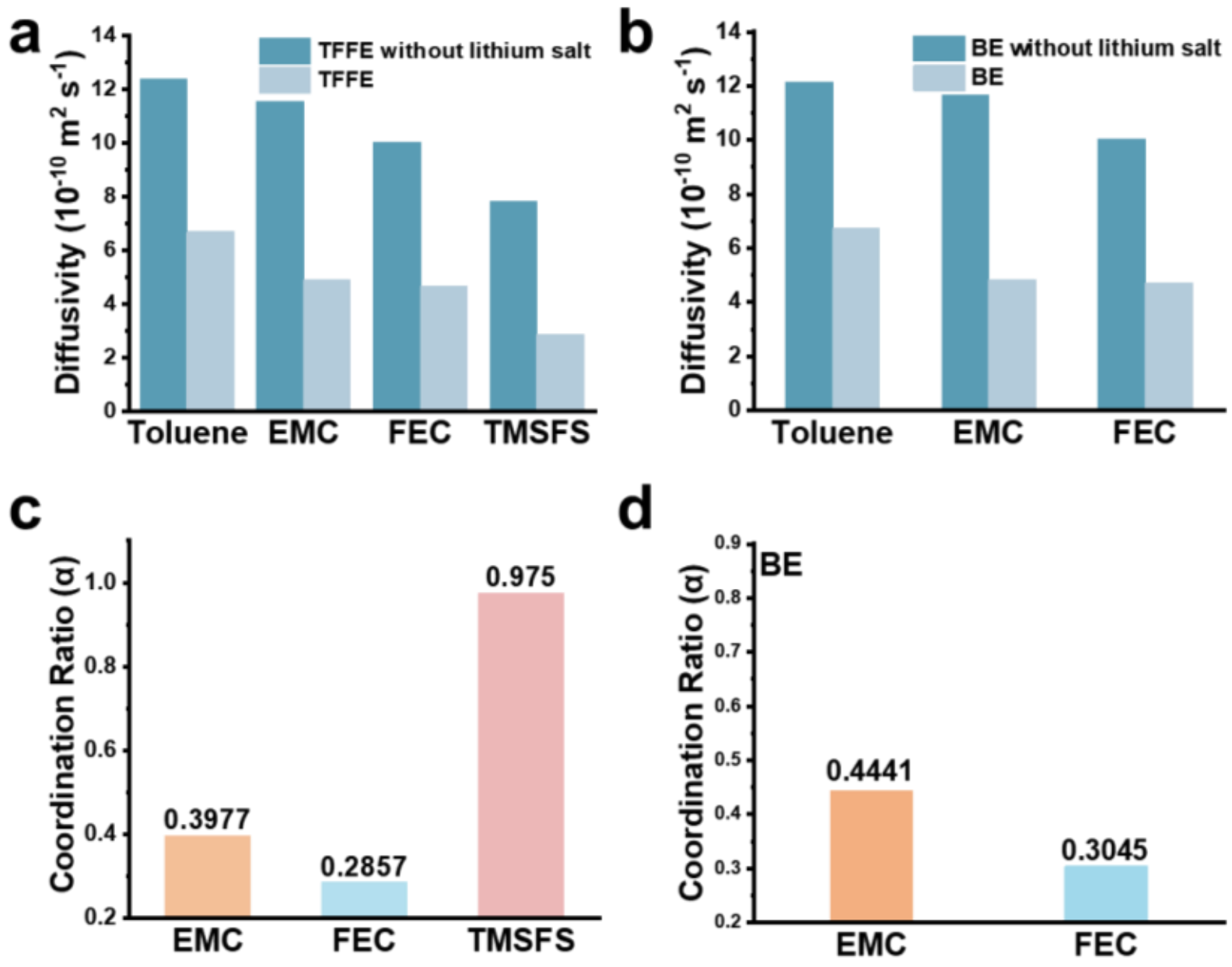

Fig. R37. Diffusion-ordered spectroscopy NMR (DOSY-NMR) analysis. The diffusivities of multiple components (toluene, EMC, FEC, TMSFS,) in (a)TFFE and TFFE without $LiPF_6$ and (b)BE and BE without $LiPF_6$; Corresponding coordination ratios calculated through DOSY of (c)TFFE and (d)BE. Toluene was used as an internal reference.

These results indicate that TMSFS exhibits a strong solvation affinity toward Li^+, and its incorporation leads to a substantial reorganization of the overall electrolyte solvation structure compared with other additives. Compared with conventional sacrificial additives, TMSFS can not only form a robust interfacial protective layer on electrode

surfaces, but also modulate of the bulk electrolyte solvation structure, thereby synergistically enhancing interfacial stability and long-term cycling durability.

Furthermore, the quality of the initial SEI structure has a significant impact on subsequent Li deposition and the inhibition of interfacial side reactions. The formation of an inorganic rich SEI layer helps achieve uniform deposition of Li metal, and continuously inhibits interfacial side reactions and dead Li accumulation during long cycling. As reported by studies on artificial-SEI layers, pre-treating the Li metal anode to build an initial uniform and inorganic-rich SEI layer, the cycling stability of the Li anode can be significantly improved.[34-36] In response to the reviewer's concern, we have provided experiments to support the advantage of this additive in stabilizing the Li metal anode, as well as its enhanced stabilizing effect during long-term cycling. The tailored solvation structure of the ACSS electrolyte promotes the formation of an inorganic-rich SEI (e.g., Li_2S and LiF), which is beneficial for achieving uniform lithium-metal deposition. High-resolution Cryo-TEM images present clear lattice fringes corresponding to crystalline Li_2S (200), Li_2O (200), and LiF (111) phases, with measured spacings of 0.29, 0.27, and 0.23 nm, respectively (Fig. R38a). XPS was also employed to elucidate the chemical composition of the SEI. The Li 1s revealed that the presence of TMSFS facilitates the formation of a multicomponent SEI dominated by inorganic species such as Li_2S, LiF, and Li_2O (Fig. R38b). Notably, the relative contribution of solvent-derived components (Li_2CO_3/$ROCO_2Li$ and $Li_xPO_yF_z$) is markedly reduced in the ACSS-derived SEI, consistent with the modified solvation structure that suppresses solvent participation in interphase formation.

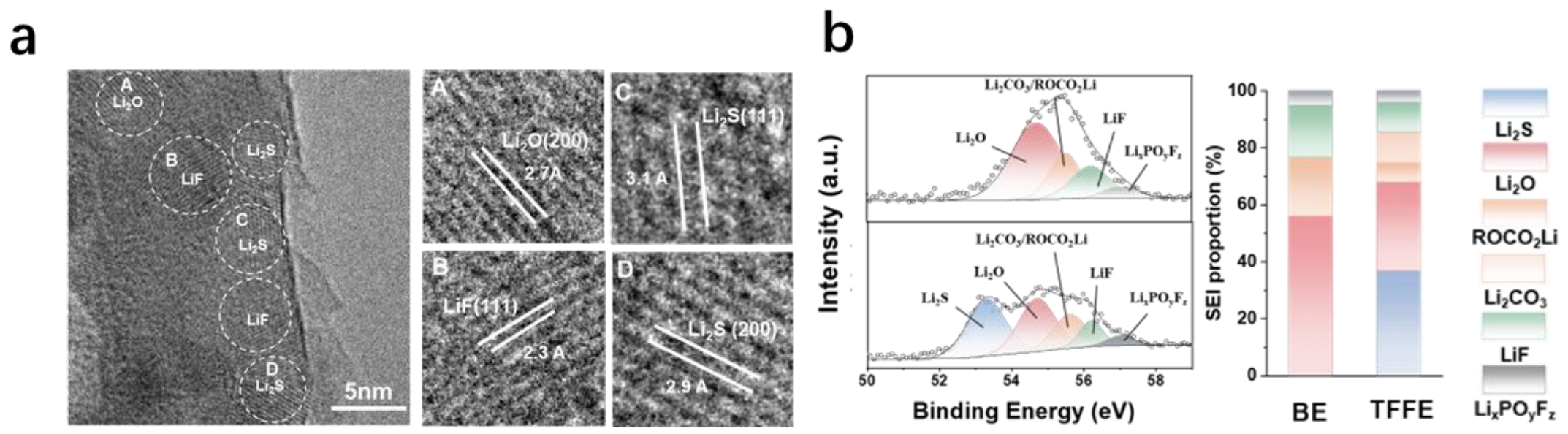

Fig. R38. (a) Cryo-TEM image of SEI layer derived from TFFE; (b) Surface XPS spectra of Li 1s in the SEI layers of Li deposits from BE and TFFE systems and proportion of different components of SEI.

Depth-profile XPS of the Li anode after 10 and 50 cycles (Fig. R39) shows clear signatures of TMSFS-derived species (Li_2S, and Li_xSO_y fragments), demonstrating that the additive contributes directly to the formation of the inorganic-rich SEI. The presence of these species indicates that TMSFS participates in SEI formation not only at the very beginning but also in later cycles, consistent with its partial retention in the electrolyte.

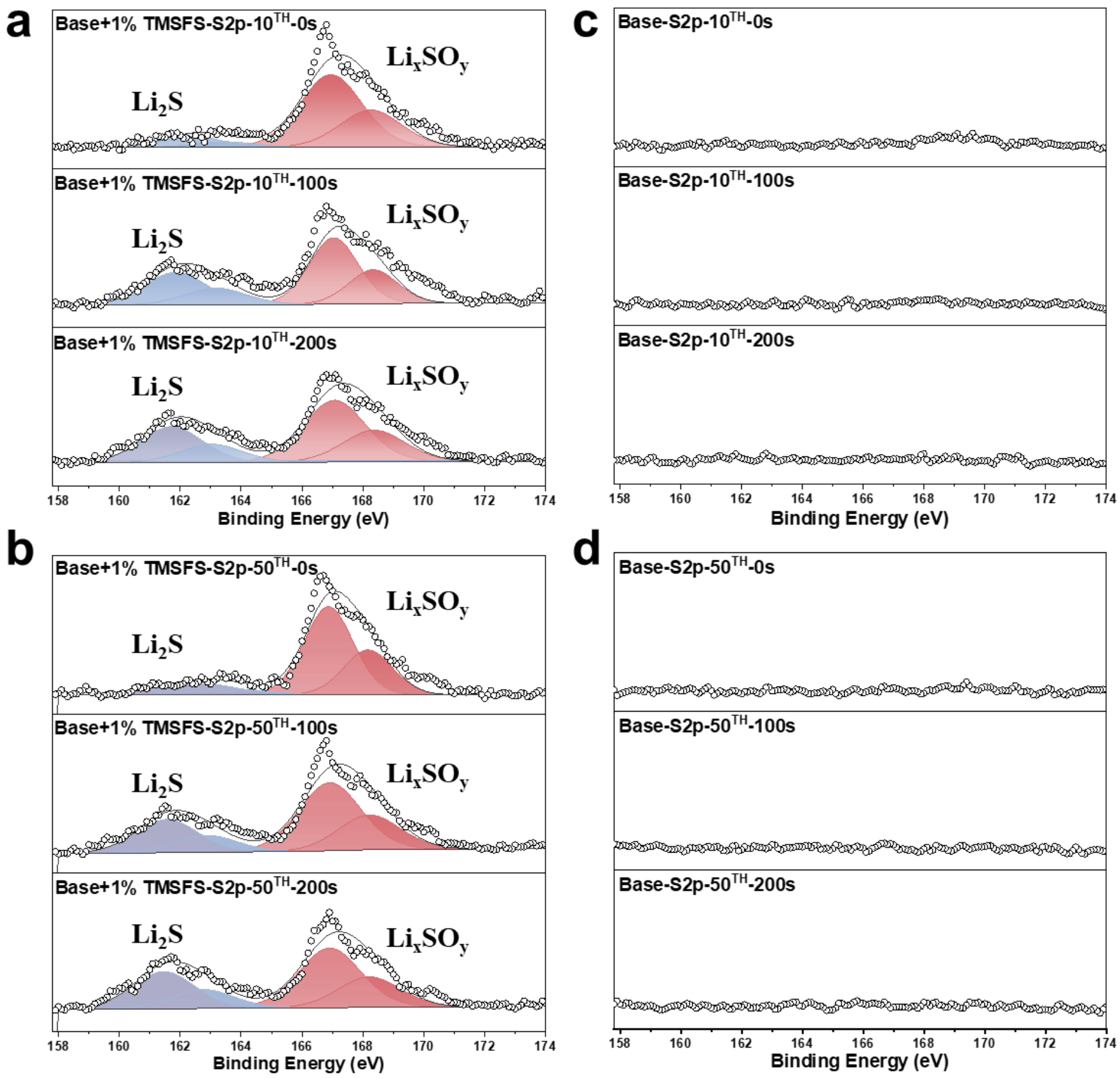

Fig. R39. Depth-profile XPS of S2p regions in the SEI layers of Li deposits from (a) TFFE (Base+1% TMSFS) after 10 cycles (b) 50 cycles and (c) BE (Base) systems after 10 cycles and (d)30 cycles.

Due to the stable and dense initial SEI layer, the average CE of Li||Cu half-cells further highlight the superior Li-metal reversibility enabled by TFFE. At a current density of 0.5 mA cm^{-2} and an areal capacity of 2 mAh cm^{-2}, the TFFE-based cell delivers a stable average CE of 97.8% over 50 cycles, whereas the BE-based counterpart fails after 30 cycles (Fig. R40a). Notably, during initial Li deposition, the TFFE system exhibits a markedly reduced nucleation overpotential (~40 mV), compared with ~140 mV for BE (Fig. R40b). Correspondingly, the voltage profiles demonstrate that the TFFE-based

cell maintains stable cycling with low polarization (<12 mV), while the BE-based cell shows much higher polarization (>20 mV) and fails at approximately 30 cycles (Fig. R40c). Also, as shown in the Fig. R40d, Li||Cu half cells assembled by TFFE exhibit much more stable cycling performance over 110 cycles compared to BE, which failed around 60 cycles. Consistent trends are observed in Li||Li symmetric cells. The TFFE electrolyte enables stable and reversible Li plating/stripping for over 2000 h at 1.0 mA cm^{-2} with a fixed capacity of 1.0 mAh cm^{-2}, maintaining a low overpotential of ~50 mV, whereas the BE-based cell exhibits rapidly increased overpotential after ~1000 h and a much shorter cycle life (Fig. R40e). These results demonstrate that TFFE effectively suppresses interfacial polarization, lowers Li nucleation barriers, and significantly enhances the cycling stability of the Li-metal anode, indicating that the TMSFS can function effectively over prolonged cycling lifetimes.

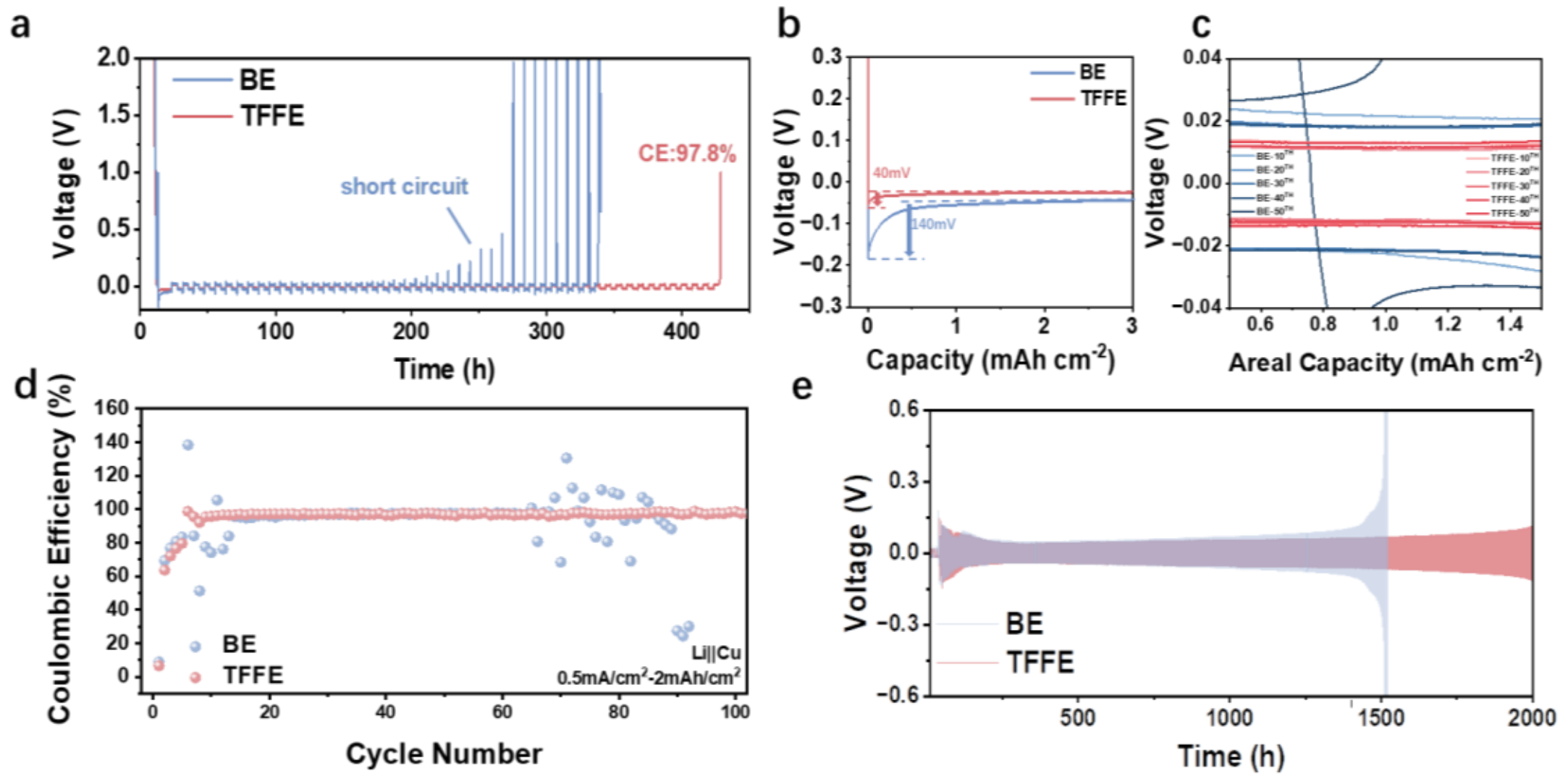

Fig. R40. (a) Average CE of BE- and TFFE-based Li||Cu half-cell over 50 cycles at 0.5 mA cm-2 and 2 mAh cm^{-2}; (b) Voltage-areal capacity profiles during the nucleation process for BE- and TFFE-based half-cells; (c)Voltage–areal capacity profiles of TFFE and BE systems at 0.5 mA cm^{-2} and 2 mAh cm^{-2}, recorded at different cycle numbers; (d) Cycling stability of BE and different amounts of TMSFS based Li||Cu half-cell at 0.5 mA cm^{-2} and 2 mAh cm^{-2}; (e)Cycling stability of BE- and TFFE-assembled Li||Li symmetric cells at 1 mA cm^{-2} and 1 mAh cm^{-2}.

In the revised manuscript, we have provided more discussion to demonstrate the effect of additive on solvation structure regulation and SEI formation. The changes of the above aspects have been supplemented in the revised manuscript highlighted with yellow, which can also be found below:

Meanwhile, internal-referenced diffusion-ordered spectroscopy (IR-DOSY) were employed to measure the solvent diffusivity. The two-dimensional ^{1}H NMR results reveal that upon the addition of the TMSFS, the as-calculated coordination ratio (α) of EMC and FEC decrease from 0.44 and 0.31 in BE to 0.39 and 0.28 in TFFE, respectively. Meanwhile, TMSFS exhibits a markedly high coordination fraction of 0.975. These results further substantiate the strong Li^+-coordinating capability of TMSFS and its effectiveness in attenuating solvent–Li^+ coordination. (Fig. 2g, h and Supplementary Fig. S7-9).

Meanwhile, depth-profile XPS of the Li anode after 10 and 50 cycles (Supplementary Fig. S26) shows clear signatures of TMSFS-derived species (Li_2S, and Li_xSO_y fragments), demonstrating that the additive contributes directly to the formation of the inorganic-rich SEI. The presence of these species indicates that TMSFS persist participating in SEI formation during tens of cycles.

Fig. R37 *were applied as Fig. 2g, h and Supplementary Figure 7.*

Fig. R38 *were applied as Fig. 4h and Supplementary Figure 25.*

Fig. R39 *were applied as Supplementary Figure 26.*

Fig. R40 *were applied as Fig. 4a, b, c and Supplementary Figure 16 and Supplementary Figure 21.*

[A. Eldesoky et al., J. Electrochem. Soc. 168, 120508 (2021)] was cited as Ref. 15

3. The primary reason why lithium metal pouch cells cannot achieve long cycling performance comparable to that of lithium-ion batteries lies in the rapid depletion of the electrolyte. This depletion mainly originates from the continuous renewal of the SEI layer on the lithium surface and the (electro)chemical corrosion of lithium. Although TMSFS–Li^+ possesses a low LUMO energy level that facilitates its decomposition on the lithium metal surface, its limited concentration means it is rapidly consumed during cycling and thus cannot play a decisive role throughout the battery's lifetime. Therefore, the low LUMO energy level should not be regarded as an advantage of this additive. I suggest that the authors shift the focus of the paper toward the formation of the cathode–electrolyte interphase (CEI), or alternatively, increase the TMSFS content instead of treating it merely as a trace additive.

Response: Thanks for the reviewer's suggestion. We agree that the formation and properties of the CEI are critical to understanding the performance enhancement observed in our electrolyte system. As suggested by the reviewer, we have incorporated a systematic analysis of CEI layer. Firstly, the TEM images (Fig. R41) clearly show a continuous and uniform CEI layer with a thickness of ~6.11 nm in the TFFE system, which falls within the commonly reported range (5–10 nm) for high-voltage cathode systems, while BE system shows an uneven and much thicker CEI with a thickness over 14 nm. These results confirms that the improved interfacial stability arises from the formation of a moderately thick, mechanically robust CEI of TFFE.

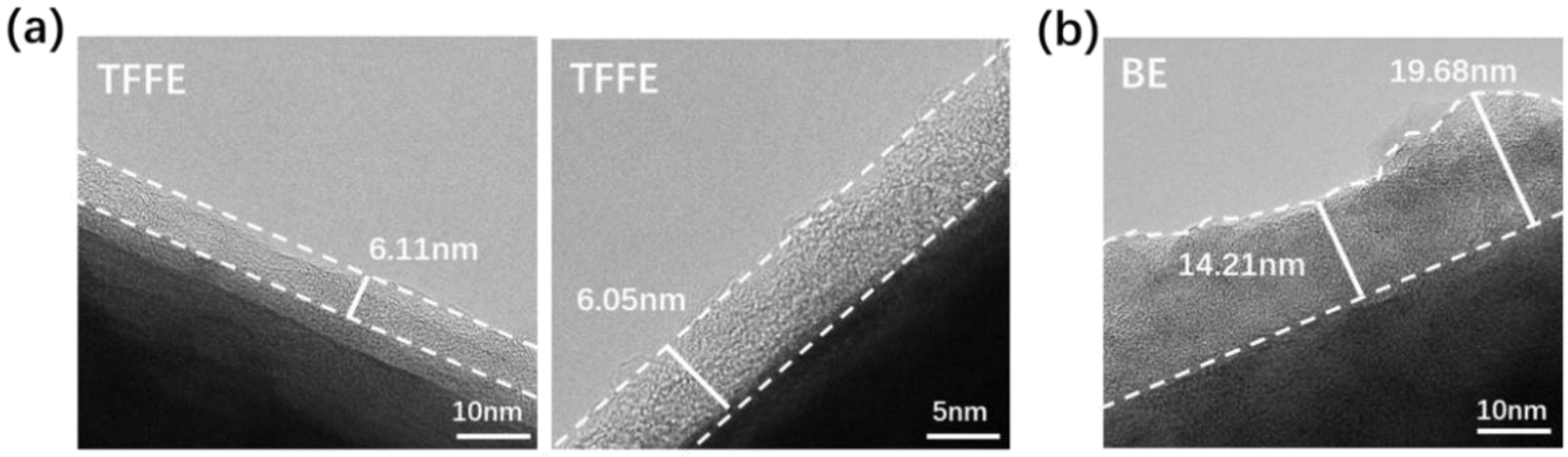

Fig. R41. TEM images of the CEI layer from Li||LRMO full cell assembled with (a)TFFE and (b)BE after 100 cycles.

Moreover, the LRMO particles displayed minimal structural damage and surface defects after 100 cycles in the TFFE electrolyte (Fig. R42a). TEM analysis and Fast Fourier Transform (FFT) patterns showed a well retained original layered structure with only a 2.7-nm-thick subsurface region undergoing phase transformation to the rock-salt structure, while the bulk interior kept its pristine layered phase structure (Fig. R42b). In sharp contrast, serious cracks could be observed on the LRMO particle in BE electrolyte, indicating phase degradation and a structural transition from a layered to a rock-salt configuration within a near-surface region of 9.8 nm in thickness ((Fig. R42c and d).

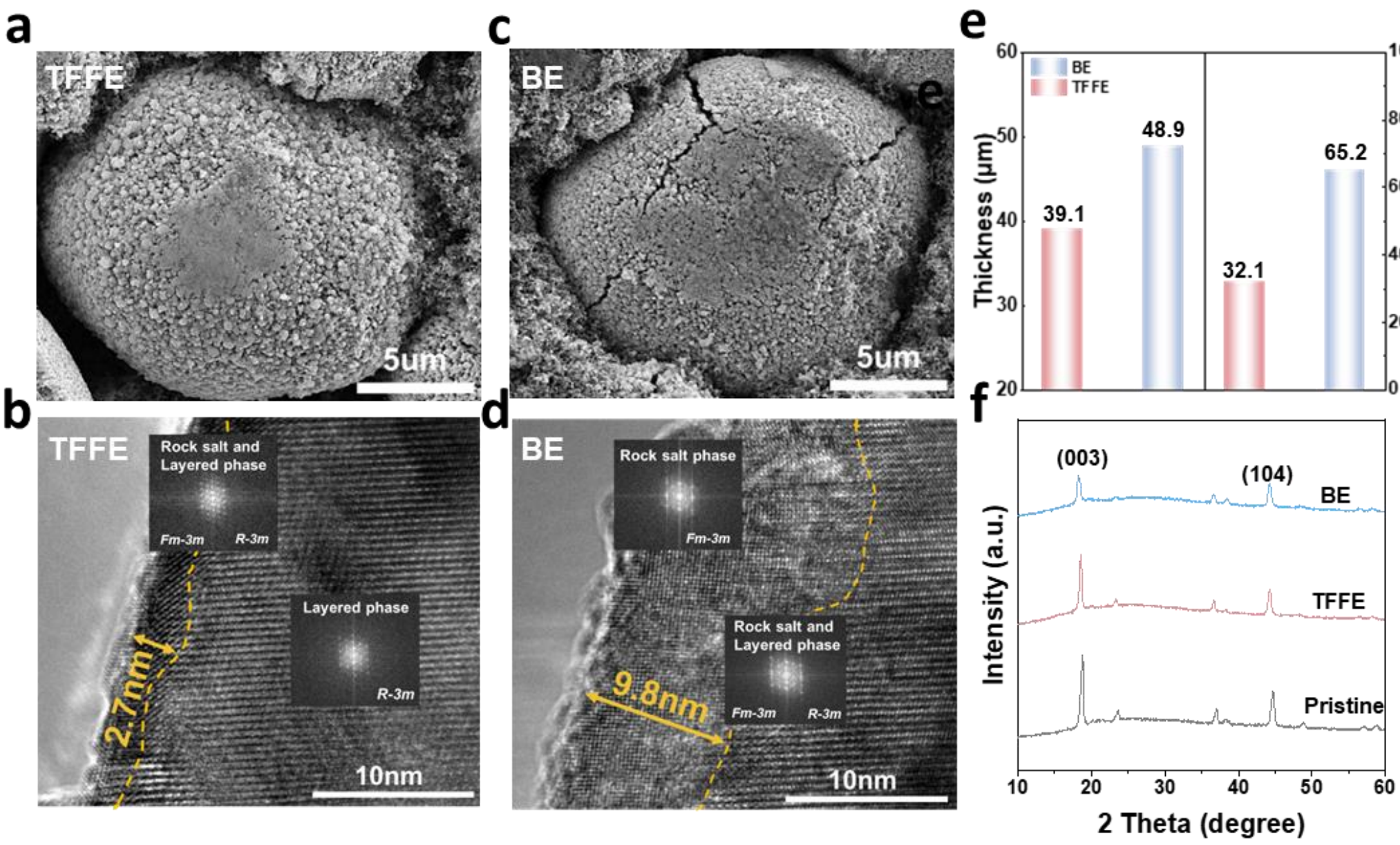

Fig. R42. (a) SEM image of the cathode surface from Li||LRMO full cell assembled with TFFE after 100 cycles; (b) TEM image of the cathode surface from Li||LRMO full cell assembled with TFFE after 100 cycles; (c) SEM image of the BE counterpart; (d) TEM image of the BE counterpart; (e) Thickness variation of the cathode after 100 cycles; (f) XRD patterns of fresh LRMOs and LRMOs after 100 cycled with BE and TFFE electrolytes.

The cross-section SEM images revealed that the thickness of the cathode cycled in TFFE increases from 29.6 μm to 39.1 μm, while the thickness of the cathode in BE expands dramatically to 48.9 μm (Fig. R42e). To further validate the cathode-stabilizing efficacy of ACSS, X-ray diffraction (XRD) analysis was conducted. As illustrated in Fig. R42f, the I (003)/I (104) ratios for pristine LRMO, cycled LRMO from TFFE-based cell and LRMO from BE-based cell are 1.522, 1.469, and 1.149, respectively, suggesting that TFFE mitigated the structural degradation of the cathode during cycling process. Conversely, the (003) peak of the BE-based sample shifted to a lower 2θ angle compared to the pristine material, accompanied by peak splitting into two distinct reflections, which indicated the severe irreversible phase transition.

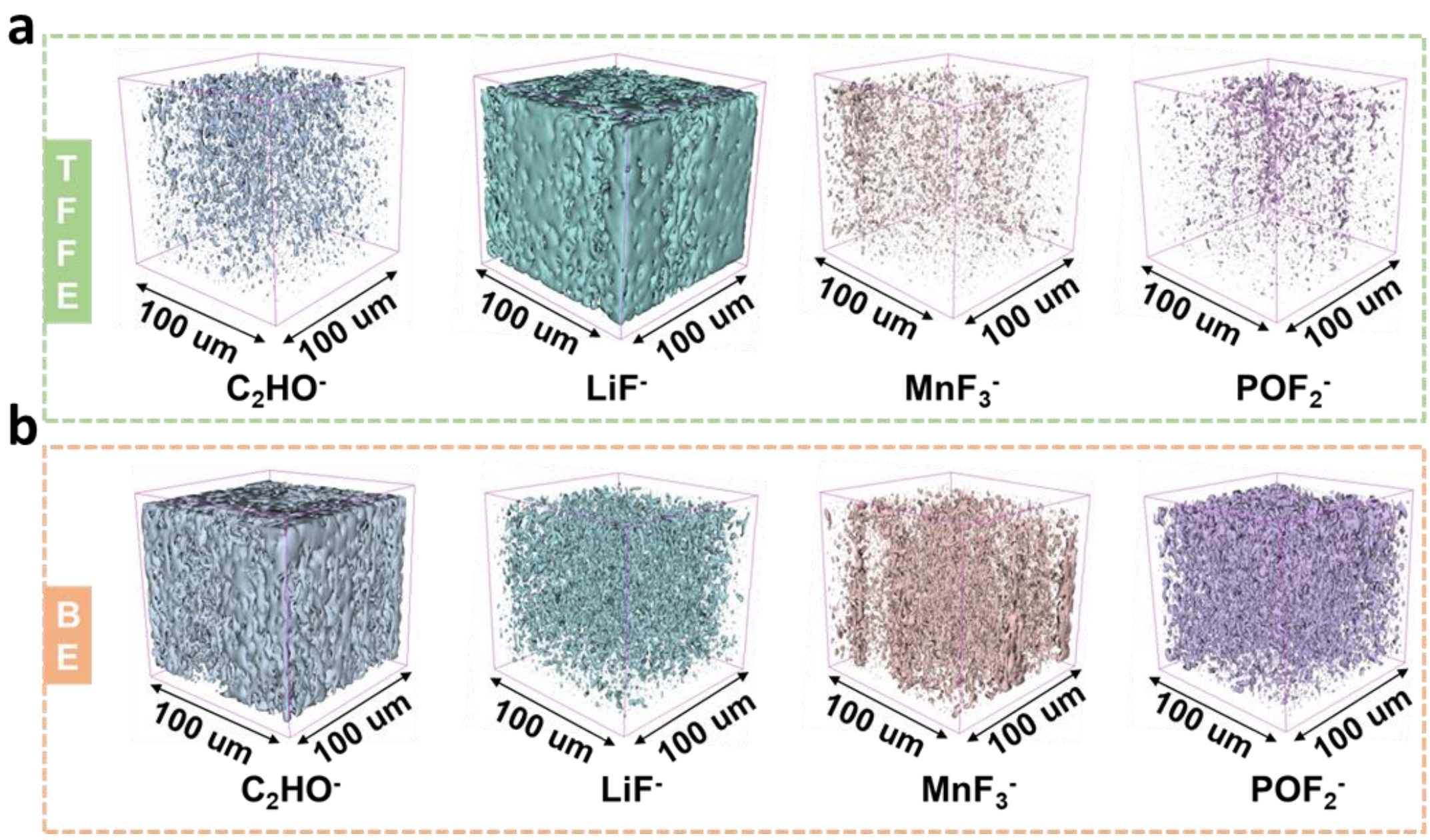

Fig. R43. 3D images of sputtering time-dependent of selected secondary ion fragments of different CEI (g) TFFE, (h) BE.

Depth sputtering ToF-SIMS was performed to investigate the role of ACSS in the formation of the CEI. As shown in Fig. R43, less C_2HO^- organic and rich inorganic species, such as LiF^-, $SiOC_2H_6^-$and SO_3^-, were uniformly dispersed across the CEI layer of TFFE. Meanwhile, MnF_3^-(from active mass dissolution products) and POF_2^-(from $LiPF_6$ decomposition) were significantly reduced compared to BE system, indicating the protective effect of the TMSFS on the cathode and the reduction of lithium salt consumption at the interface. These results indicated that the ACSS effectively suppressed the decomposition of lithium salts and solvents on the interface of electrode, and the resulting ACSS derived-CEI contributed to preserve the structural integrity of cathode during extended cycling under high-voltage conditions.

Besides, the limited cycling life of Li metal pouch cells is also affected by continuous SEI renewal and chemical/electrochemical corrosion of Li. Consequently, TMSFS contributes to enhanced SEI stability at the Li metal anode, thereby improving its long-term cycling performance. Scanning electron microscopy (SEM) images of Li deposition further validated the superiority of the ACSS-derived SEI. When 3 mAh cm^{-2} of Li was deposited on Cu electrodes, uniformly distributed Li nuclei with compact and homogeneous morphology were observed in the TFFE system (Fig. R44a). In contrast, BE displayed mossy and irregular Li deposits under the same conditions (Fig.

R44b). To further probe the depth-dependent composition of the SEI, time-of-flight secondary-ion mass spectrometry (ToF-SIMS) was conducted (Fig. R44c and d). The inorganic fragments LiO^-, LiS^-, and LiF^- exhibit a relatively uniform distribution near the surface, with signal intensities gradually decaying upon sputtering, in good agreement with the Cryo-TEM observations. Collectively, these results demonstrate that the ACSS-derived, inorganic-rich and structurally uniform SEI effectively homogenizes the Li^+ flux at the interface, thereby facilitating uniform lithium-metal deposition.

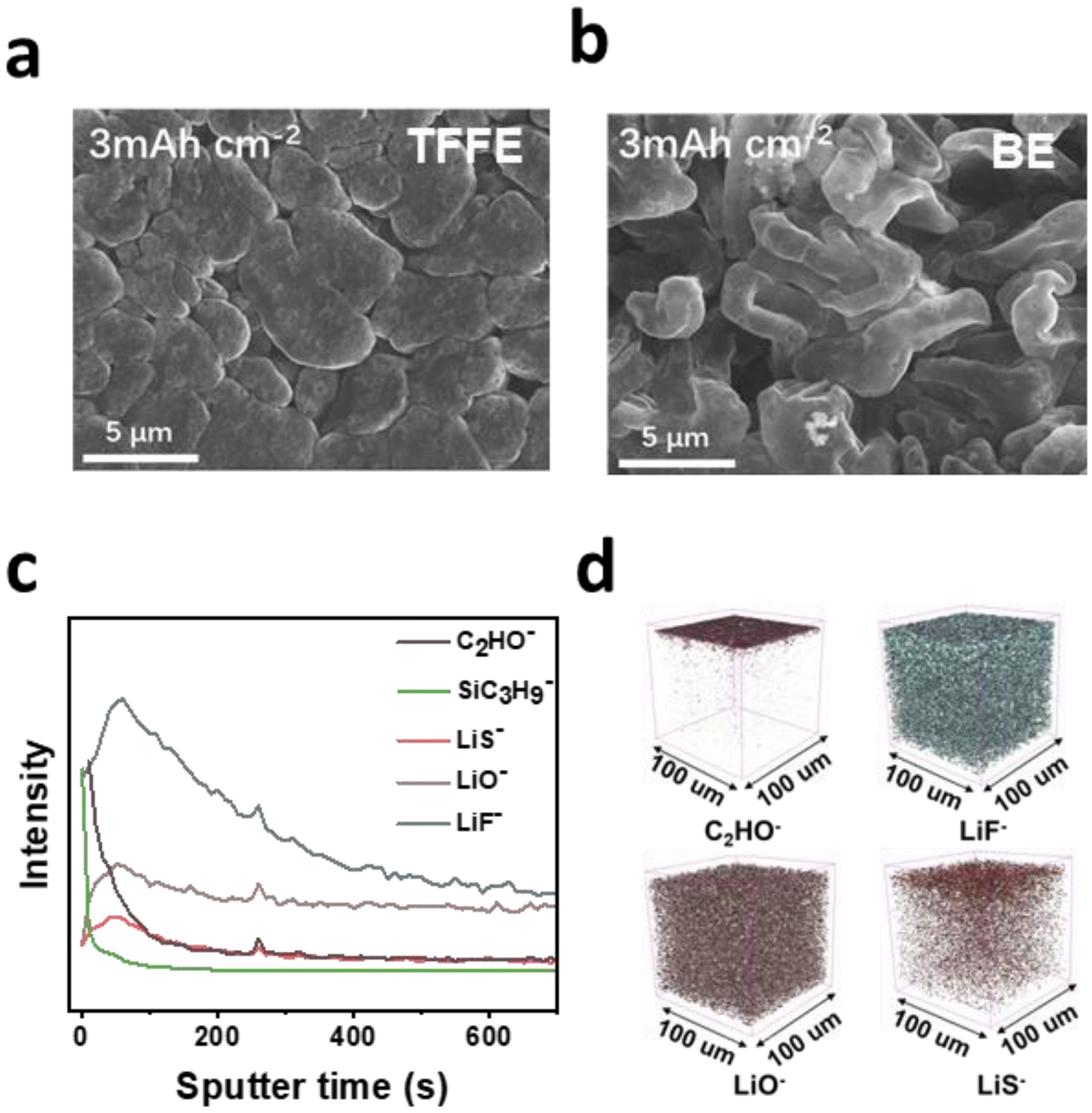

Fig. R44. SEM images of 3 mAh cm^{-2} of Li depositing on Cu electrodes of (a) TFFE- and (b)BE-based half-cells. (c) Sputtering time-dependent compositional profiles of selected secondary ion fragments of different SEI component and (d) corresponding 3D images;

Owing to TFFE promoting effect on interfacial film formation, pouch cell incorporating TFFE achieve a lifetime-averaged Coulombic efficiency exceeding 99.5%, with the Coulombic efficiency remaining consistently above 98% over extended cycling. These results indicate that a highly stable electrode–electrolyte interface and suggesting that cell failure is mainly associated with electrolyte depletion, which are also shown in Fig. R45. In contrast, the BE cell exhibits a simultaneous abrupt decay in both CE and

capacity, indicative of internal short circuit caused by cathode structure degradation and Li dendrite formation (Fig. R46), ultimately leading to cell failure.[8]

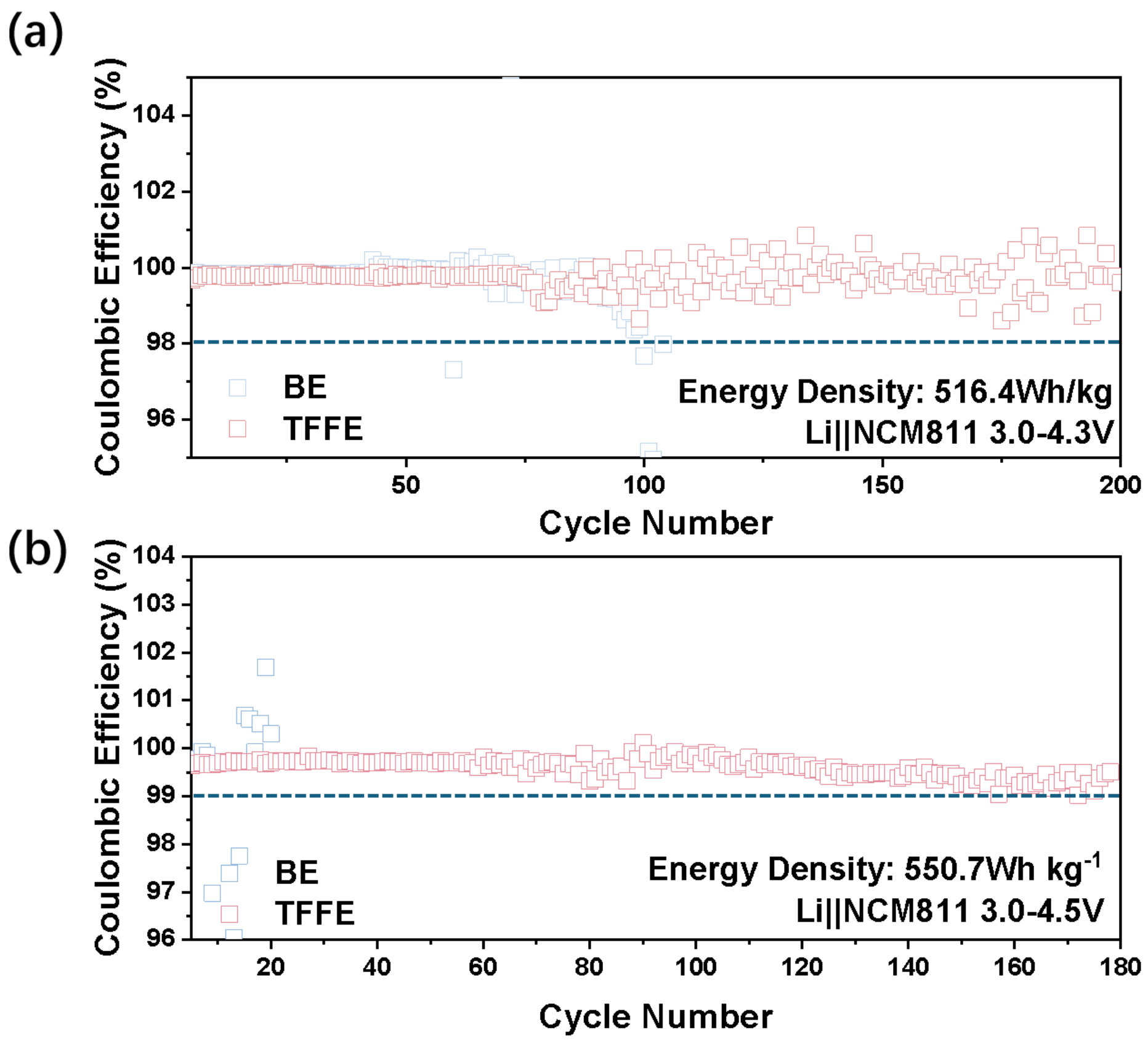

Fig. R45. The enlarged CE profile of assembled pouch cells. (a)516.4 Wh/kg and (b)550.7 Wh/kg.

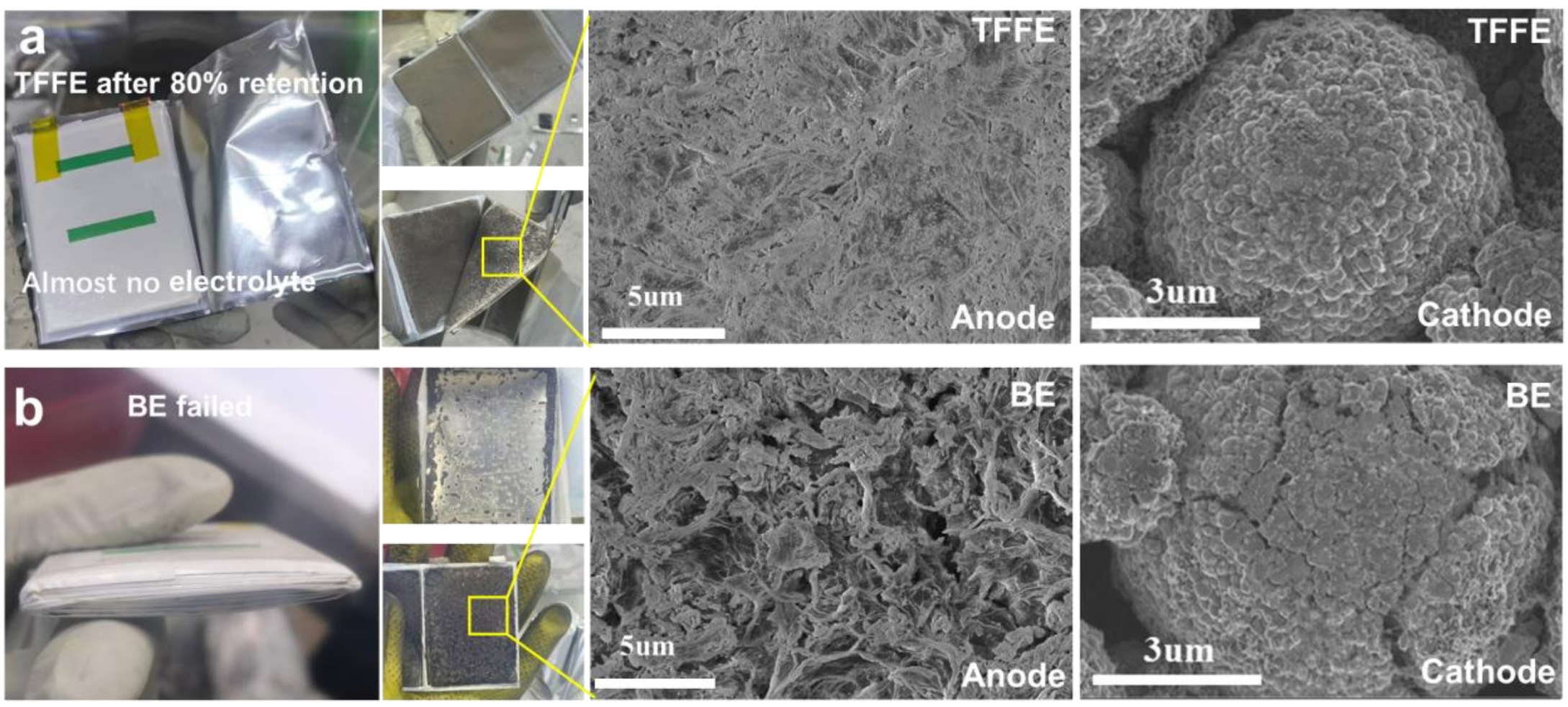

Fig. R46 Representative image and the corresponding SEM images of Li-metal pouch cell after cycling assembled with (a)TFFE, (b)BE.

Also, Li||LRMO full cells were assembled to test the cycling stability of different addition systems. 1wt.% addition of TMSFS system showed the most favorable cycling

stability and capacity retention (Fig. R47). This performance trend is attributed to the high content of additives can lead to severe side reactions, resulting in increased impedance and reduced efficiency. This will be further explained in the following sections.

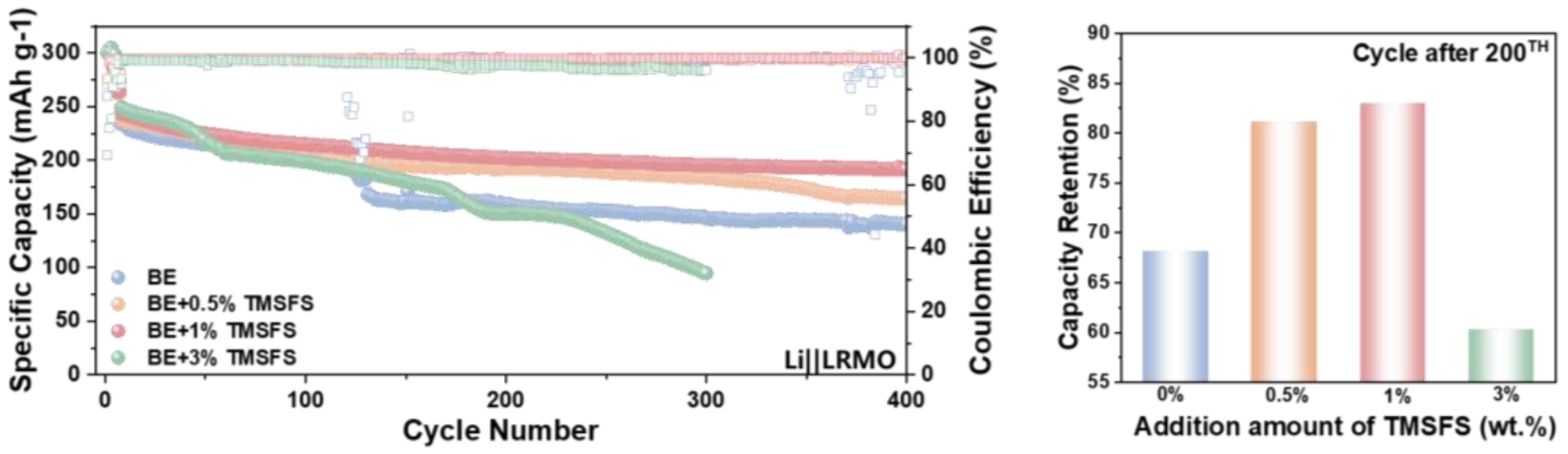

Fig. R47. (a) Li||LRMO Full coin cells assembled with different content of TMSFS and (b)corresponding capacity retention after 200 cycles.

We trust that the above discussion adequately addresses the reviewer's concerns regarding additive consumption during battery cycle life. In the revised manuscript, we have provided more discussion to highlight the CEI of the TFFE. The changes of the above aspects have been supplemented in the revised manuscript highlighted with yellow, which can also be found below:

TEM analysis and Fast Fourier Transform (FFT) patterns showed a continuous and uniform CEI layer with a thickness of ~6.11 nm was spotted and that LRMO particles well retained their original layered structure with only a 2.7-nm-thick subsurface region undergoing phase transformation to the rock-salt structure, while the bulk interior kept its pristine layered phase structure (Fig. 3a and 3b). In sharp contrast, serious cracks could be observed on the LRMO particle in BE electrolyte (Supplementary Fig. S12b), and BE system showed an uneven and much thicker CEI with a thickness over 14 nm (Fig. 3c), indicating phase degradation and a structural transition from a layered to a rock-salt configuration within a near-surface region of 9.8 nm in thickness (Fig. 3d).

As shown in Supplementary Fig. S44, TFFE-based pouch cells exhibit consistently high CE (>98%) throughout cycling, indicating interface-stable failure dominated by electrolyte depletion, whereas the control cells show simultaneous CE and capacity collapse, characteristic of Li anode degradation, leading to premature failure.

Furthermore, full-cell test results with different additive contents indicated that the

addition of 1 wt.% exhibited the optimal performance (Supplementary Fig. S31), which was caused by the fierce side reactions caused by excessive addition and the corresponding thicker and less conductive interface reaction layer.

***Fig. R41** were applied as Fig. 3a and c.*

***Fig. R42** were applied as Supplementary Figure 12 and Fig. 3b, d, e and f.*

***Fig. R43** were applied as Fig. 3g and h.*

***Fig. R44** were applied as Fig. 4f, g, i and j.*

***Fig. R45** were applied as Supplementary Figure 44.*

***Fig. R46** were applied as Supplementary Figure 45 and Supplementary Figure 46.*

***Fig. R47** were applied as Supplementary Figure 31.*

4. The amount of additive should be consistently defined as either a mass fraction or a volume fraction. In Fig. 2e and the Methods section, it is described as a mass fraction, whereas in the main text it is presented as a volume fraction. In addition, the authors should clarify the rationale for selecting this particular concentration of additive or provide an optimization study of the additive content.

Response: Thanks for the reviewer's suggestion. We have now revised the entire manuscript to consistently define the additive concentration using mass fraction (wt.%), including Fig. 2e, the main text, and the Methods section. The misleading volume-fraction description in the main text has been corrected accordingly. To clarify the rationale for selecting this particular concentration, we have systematically provided some basic properties.

From the anode side, CE cycling, impedance of SEI formed under the condition of different amount of TMSFS and the thickness and surface states of the corresponding interface reaction layer have been tested. As shown in Fig. R48, half cells containing 1wt.% TMSFS exhibited the most stable cycling behavior, showing a clear improvement compared with the baseline electrolyte (BE). However, as the additive content increases beyond 1wt.%, the beneficial effect becomes progressively less pronounced, and excessive addition (e.g., 3wt.%) ultimately leads to premature cell failure.

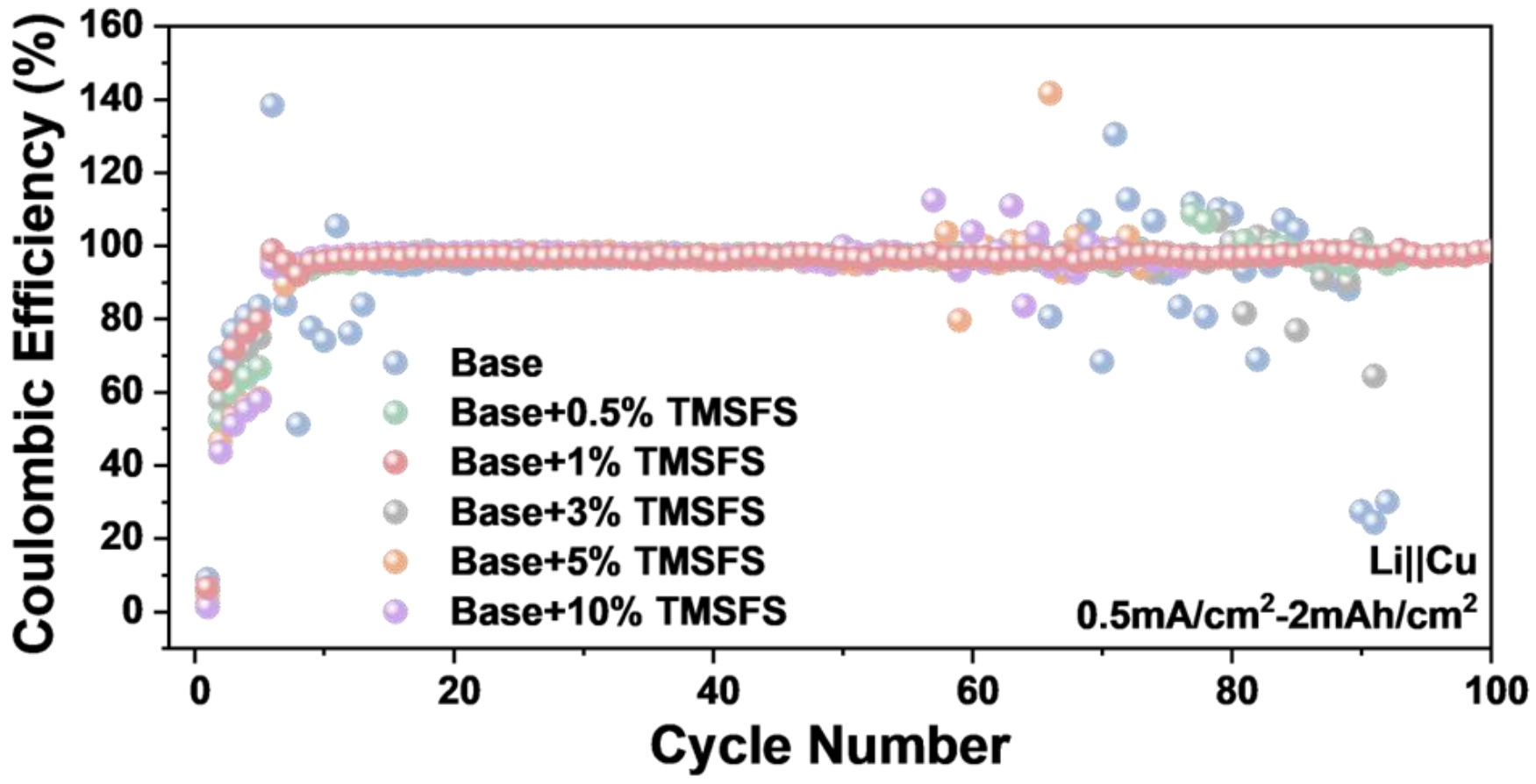

Fig. R48. Cycling stability of BE and different amounts of TMSFS based Li||Cu half-cell at 0.5 mA cm^{-2} and 2 mAh cm^{-2}.

Furthermore, 3mAh cm^{-2} of Li was deposited on the Cu foil to form SEI with different addition amount of TMSFS. According to the EIS results in Fig. R49, the impedance of the formed SEI showed a higher tendency as the addition amount increased from 0.5 wt.% to 10 wt.%, which indicates excessive addition may induce thicker or uneven SEI. Meanwhile, the cross-section FIB-SEM images and shots of surface states after 10 cycles (the surface of the sample has not been treated with solvent cleaning) also showed thicker interface reaction layer suggesting fierce side reactions caused by excessive addition of TMSFS (Fig. R50).

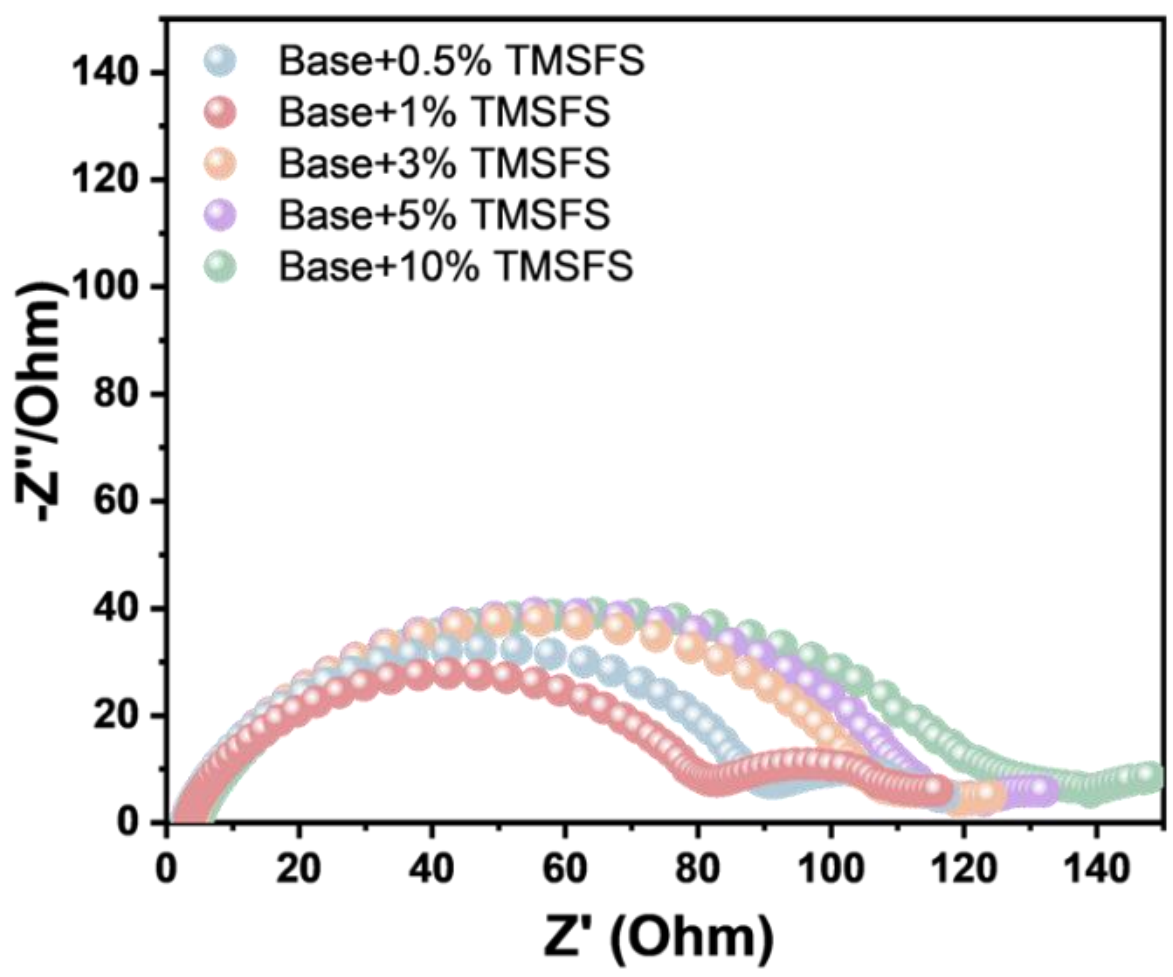

Fig. R49. EIS spectra measurements of cells after activation assembled by BE+ different amounts of TMSFS.

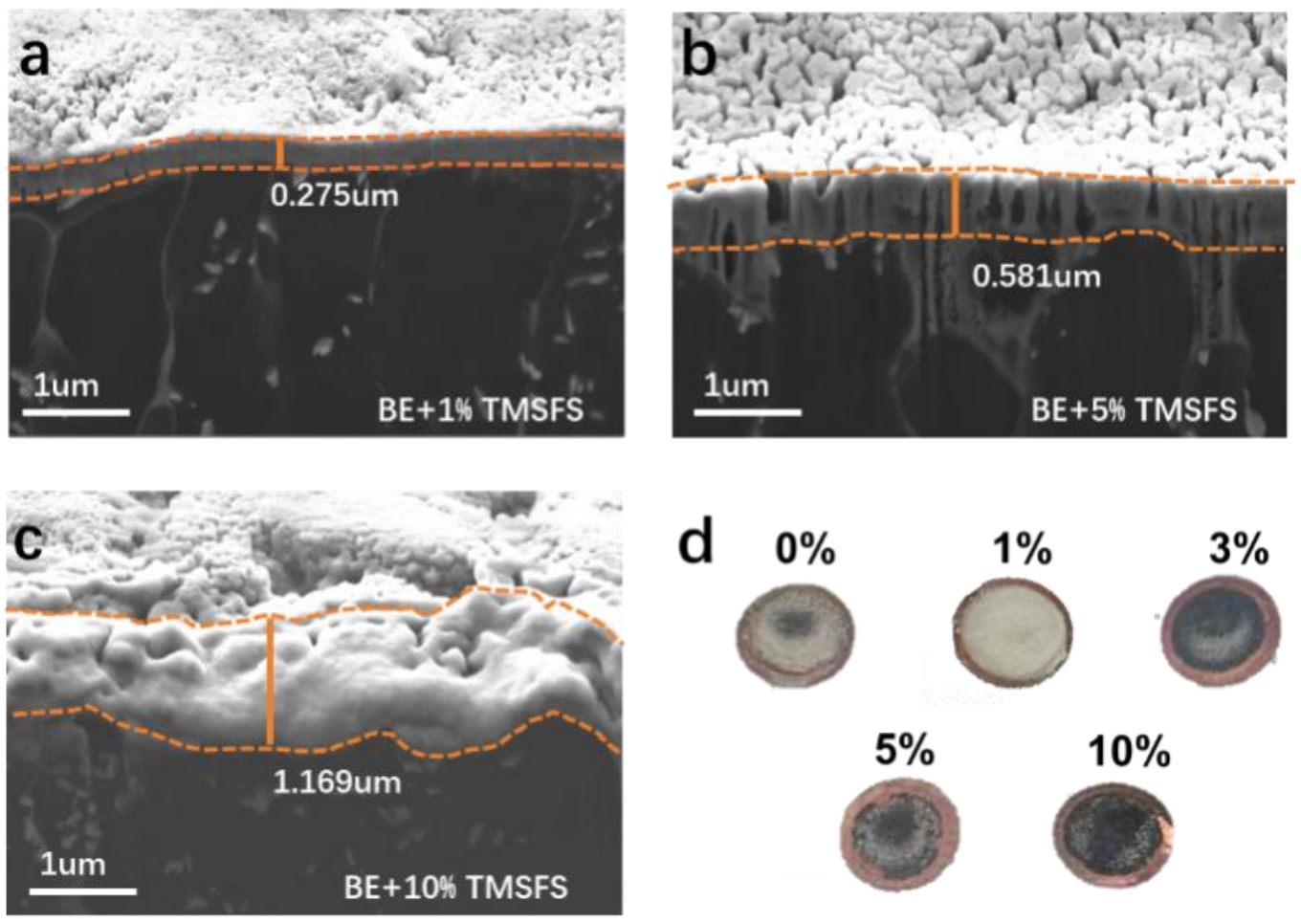

Fig. R50. Cross-section FIB-SEM images of deposited Li on Cu foil after 10 cycles of (a) BE+1wt.% TMSFS (TFFE)-, (b) BE+5wt.% TMSFS, (c) BE+10wt.% TMSFS and (d) corresponding shots of surface states.

These results of basic properties indicated that the 1 wt.% amount of TMSFS is the optimal. In the revised manuscript, we have provided more characterizations to highlight the novelty of the design strategy. The changes of the above aspects have been supplemented in the revised manuscript highlighted with yellow, which can also be found below:

Firstly, electrochemical performance tests were conducted with different addition of TMSFS. As shown in Supplementary Fig. S16, half-cells with 1 wt.% TMSFS exhibit the most stable cycling compared with the baseline electrolyte and other additive contents. Cross-sectional FIB–SEM and surface images reveal that increasing the TMSFS concentration leads to a progressively thicker interfacial reaction layer on the Li metal surface (Supplementary Fig. S17), which is attributed to excessive parasitic reactions between TMSFS and Li metal, as reported previously.

Fig. R48 *were applied as Supplementary Figure 16.*

Fig. R49 *were applied as Supplementary Figure 18.*

Fig. R50 *were applied as Supplementary Figure 17.*

5. In your calculations, the binding energy between Li and EMC is more negative (compared to that between Li and FEC). However, in the MD simulations, after introducing TMSFS, it primarily replaces EMC rather than FEC. This result is puzzling. In addition, it remains unclear why the coordination number of PF_6^- is significantly reduced upon the trace addition of TMSFS.

Response: We thank the reviewer for this critical comment. After carefully re-examining our MD setup, we realized that the previously reported MD solvation numbers and coordination statistics were not fully converged, due to two methodological issues in our original simulations.

First, we initially adopted a 1.20×CM5 charge model that had been optimized for use with the OPLS force field in neutral organic systems.[37] However, for ionic and highly concentrated Li-salt electrolytes, OPLS is a non-polarizable force field and the missing electronic polarization and screening are usually compensated by applying an electronic continuum correction (ECC) scaling to the partial charges.[38] The original 1.20×CM5 charge setup is therefore more appropriate for neutral molecules than for Li-salt electrolytes. In the revised work, we have replaced this scheme by RESP charges combined with an ECC scaling factor of 0.8, which has been successfully used for Li-ion systems.[39] Second, our original production runs were carried out in a rather small periodic box (only ~60 molecules in total, including 5 Li^+, ≈750 atoms, box length ≈22 Å). In such a small closed system, the radial distribution functions (RDFs) and coordination numbers are strongly influenced by the specific local environments of a few ions and are subject to significant finite-size artifacts, as discussed for example by Prof T. J. H. Vlugt (Molecular Simulation 44, 599-612 (2017)).[40] To reduce these artifacts and obtain statistically converged solvation structures, we rebuilt the system to contain ~1,500 molecules (~20,000 atoms) in a cubic box of 60.46 Å. At this box size, the relevant structural correlations (Li–O first-shell distances of ≈2–3 Å and Li–solvent correlations within ≲10 Å) are well below half the box length, and each trajectory frame samples more than one hundred Li^+ ions, which together provide robust statistics for the RDFs and coordination numbers. With the corrected charge model and the enlarged box, we reperformed all MD simulations and RDF/coordination-number analyses, and we have replaced the previous solvation numbers in the revised manuscript. And it is noted that these corrections refine the quantitative description of the Li^+ solvation structure but do not change the qualitative trends or the main conclusions of the manuscript.

In our DFT results, the Li^+–EMC and Li^+–FEC binding energies are very close

(difference < 1 kcal·mol^{-1}), indicating that EMC and FEC have comparable affinity toward Li^+. In the electrolyte composition EMC: FEC = 4:1 (by volume), EMC is the dominant solvent component and thus contributes most to the Li^+ first-shell population. Consequently, when a trace amount of TMSFS enters the inner solvation sheath, the largest absolute reduction is expected to appear in the majority ligand (EMC). Importantly, the updated MD results show that both EMC and FEC coordination numbers decrease slightly upon adding TMSFS, rather than an exclusive replacement of only EMC. The absolute decrease is larger for EMC (-0.157) because it dominates the solvation shell, while the fractional decrease of FEC (-0.031/0.591 ≈ -5.2%) is comparable to that of EMC (-0.157/3.464 ≈ -4.5%) (Fig. R51 and 52, Table R8).

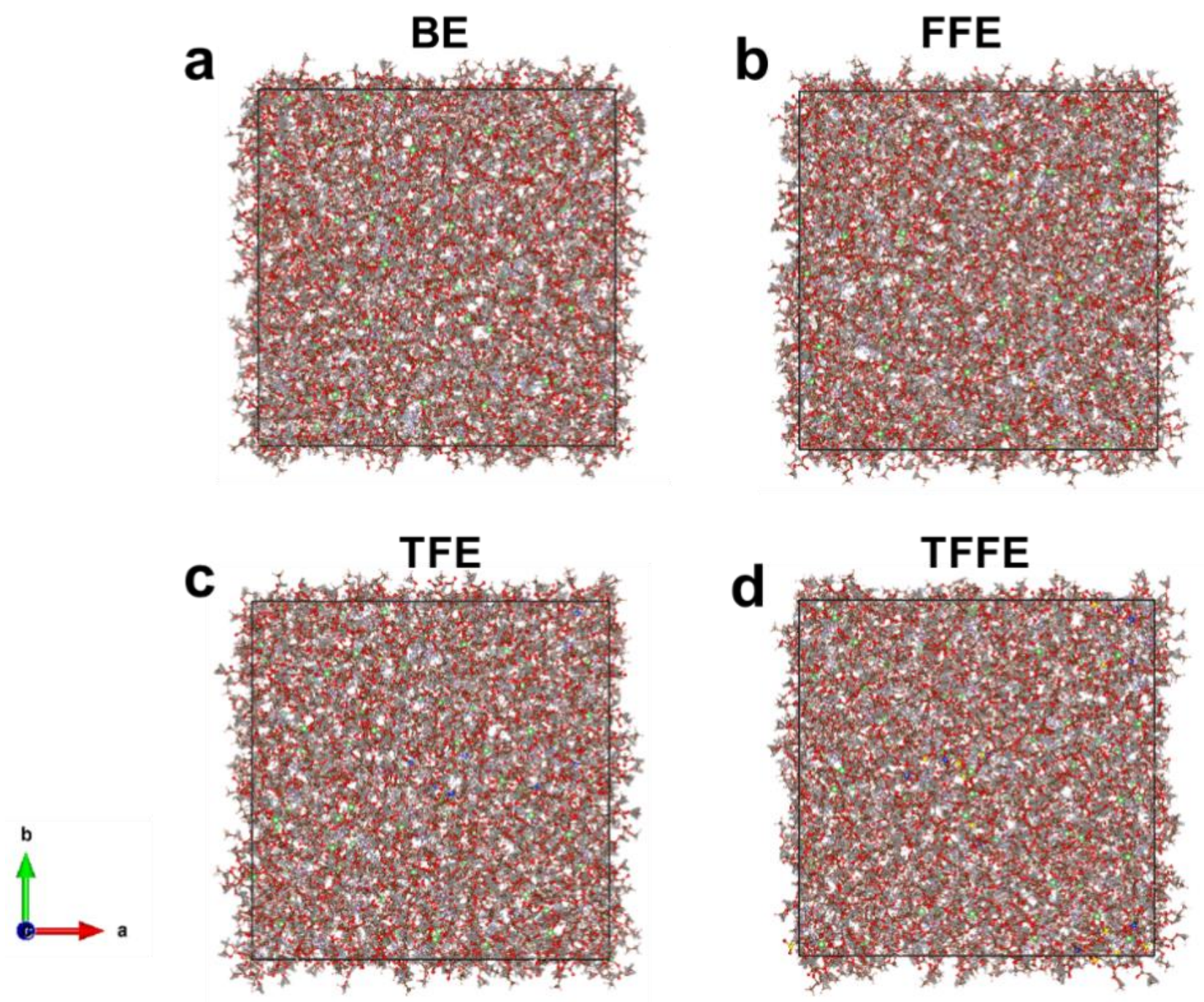

Fig. R51. Classical molecular dynamics (MD) simulations and schematic representations of solvation structures of (a) BE, (b) FFE, (c) TFE and (d) TFFE.

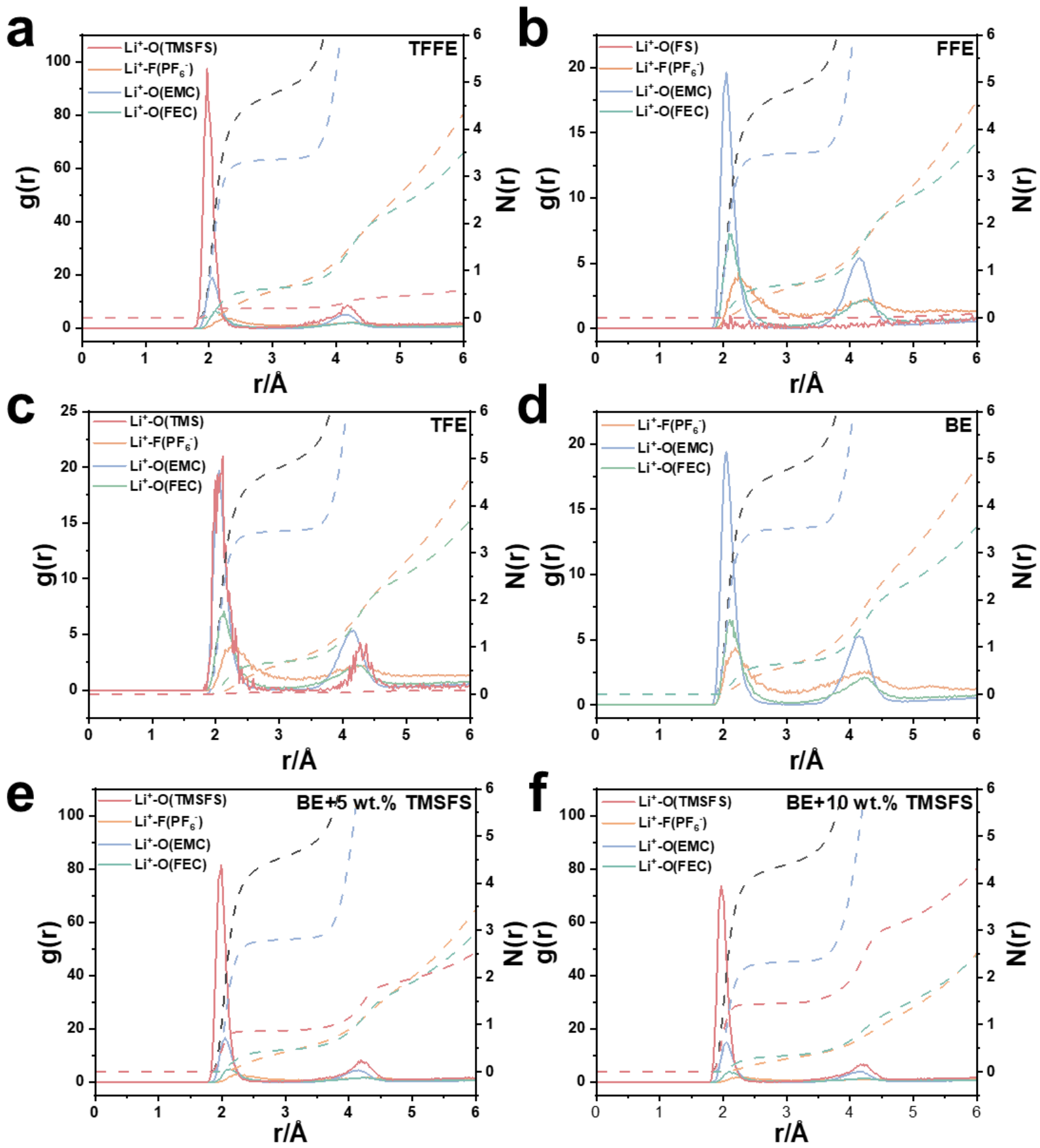

Fig. R52. RDFs of key species coordinating with Li^+ in (a) TFFE, (b) FFE, (c) TFE, (d) BE systems, (e) BE+5 wt.% TMSFS and (f) BE+10 wt.%TMSFS.

Table R8. The comparison of calculated coordination number of all species in BE, TFFE, FFE and TFE systems.

System	component	Coordination number
BE	PF_6^-	0.360
	EMC	3.464
	FEC	0.591
	Total	4.415
TFFE	**PF_6^-**	**0.264**
	EMC	3.307
	FEC	0.560
	**TMSFS**	**0.194**
	Total	4.325
FFE	PF_6^-	0.295
	EMC	3.435
	FEC	0.635
	FS	0.002
	Total	4.367
TFE	PF_6^-	0.296

EMC	3.415
FEC	0.624
TMS	0.034
Total	4.369

The revised MD indicates that PF_6^- in the first shell decreases from 0.360 to 0.264 per Li^+ upon introducing 1 wt.% TMSFS. This reduction is explained by site competition in the first solvation shell: TMSFS provides strongly coordinating oxygen sites (carbonyl/sulfonyl), and once TMSFS occupies part of the inner-shell coordination positions, the probability of direct Li^+–PF_6^- contact within the first-shell cutoff correspondingly decreases. Notably, after correcting the charge model and enlarging the box, the magnitude of the PF_6^- change becomes moderate and physically reasonable, in contrast to the overly strong reduction implied by the previous preliminary setup.

In the revised manuscript, we have replaced the previous MD solvation numbers with the revised values above. We have rewritten the discussion to clarify that TMSFS incorporation causes small reductions in both EMC and FEC coordination (with a larger absolute effect on EMC due to its higher population), and a moderate decrease in PF_6^- first-shell coordination consistent with first-shell site competition.

Further comparative analysis of the coordination numbers showed that TFFE exhibits less coordinated EMC and PF_6^- *(*$Li^+EMC_{3.307}FEC_{0.560}TMSFS_{0.194}PF_6^-{}_{0.264}$*) compared to BE system (*$Li^+EMC_{3.464}FEC_{0.591}PF_6^-{}_{0.360}$*), and upon further increasing the TMSFS content to 5 wt.% and 10 wt.%, CN(TMSFS) increases to 0.820 and 1.389, respectively (Supplementary Figs. S4). This indicates that TMSFS partially replaces EMC and FEC solvent molecules and anions in the first solvation shell of* Li^+*, thereby effectively suppressing interfacial side reactions involving solvent molecules and delaying the excessive consumption of anions.*

Fig. R51 *were applied as Supplementary Figure 3.*

Fig. R52 *were applied as Fig. 2b, c, d, e and Supplementary Figure 4.*

6. In the MD simulations, the total coordination number of Li is typically around 4.5 (and rarely below 4), which is determined by the van der Waals radius of lithium. However, in the electrolyte containing TMSFS, the reported total coordination number

is only 3.5, which appears problematic. Please verify whether the simulation box volume and system density are reasonable. Moreover, in the RDF results, the peak intensity of Li–TMSFS is 4.5 times that of Li–FEC. Considering that each TMSFS molecule contributes two coordinating oxygen atoms, while each FEC molecule contributes one (both containing three oxygens in total), and that the number of FEC molecules is 13 times that of TMSFS, the coordination number of FEC should theoretically be **13 / (4.5 / 2) = 5.77** times that of TMSFS. However, the authors report only a 2.55-fold difference. Please re-examine the calculation procedure.

Response: We appreciate the reviewer's careful check. We agree that our previously reported total coordination number (~3.5) in the TMSFS-containing electrolyte was not consistent with the typical Li^+ solvation environment in carbonate electrolytes. As detailed in our response to Comment 5, this underestimation originated from two limitations in the original MD setup: (i) the use of an early charge model that was not optimal for concentrated Li-salt electrolytes, and (ii) a small periodic box (≈22 Å, only ~60 molecules including 5 Li^+), which introduced strong finite-size and sampling artefacts in the RDFs and coordination statistics. In the revised work, we have corrected these issues by adopting RESP charges with ECC scaling and enlarging the simulation box to 60.46 Å (~1500 molecules, 133 Li^+). On this basis, we re-did the MD simulations and recomputed the RDFs and coordination numbers using a consistent and verifiable procedure.

As detailed in our previous response, we have corrected the MD setup by updating the charge model and enlarging the simulation box. Partial charges were obtained from RESP fitting and uniformly scaled with ECC (scaling factor 0.8). The revised TMSFS system was simulated in a cubic box of 60.46 Å, containing 133 $LiPF_6$, 982 EMC, 348 FEC, and 13 TMSFS molecules, corresponding to 1 M $LiPF_6$ in EMC: FEC = 4:1 (by volume) with 1 wt.% TMSFS. With this composition and box size, the resulting average density is ~1.22 g·cm^{-3}, which is in good agreement with our experimentally measured density for this 1 M $LiPF_6$ carbonate electrolyte. We have added the box size/composition and the corresponding density information in the revised Methods/Supporting Information for transparency.

Using the revised simulations, the total Li^+ first-shell coordination number is now: 4.415 for the baseline electrolyte (no additive) and 4.325 for the electrolyte with 1 wt.% TMSFS. These totals are consistent with the typical Li^+ coordination number (~4–5). The previous ~3.5 value mainly resulted from a severe underestimation of the PF_6^- contribution to the first solvation shell in the preliminary small-box/charge setup: as PF_6^- is the only ionic ligand and much less numerous than solvent molecules, its coordination statistics were particularly sensitive to finite-size and electrostatic artefacts. This has now been corrected, and all solvation numbers have been fully replaced by the present values in the revision.

The coordination numbers are computed from the standard RDF integration (or an equivalent neighbor-counting method) using the first-shell cutoff defined by the first minimum:

$$CN\ \ (r) = 4\pi\rho \int_{0}^{r_{min}} g(r)r^2dr$$

In the revised analysis, we used the first-minimum cutoff (2.57 Å) and reported molecular coordination numbers (counting distinct coordinating molecules within the cutoff) to avoid ambiguity from multi-oxygen ligands. Importantly, RDF peak intensity alone cannot be used to infer CN ratios, especially when comparing a trace additive to a major solvent. For a dilute component such as TMSFS, the RDF is normalized by a small bulk number density; therefore, a high first-peak value mainly indicates strong local enrichment around Li^+, not a proportionally large absolute coordination number. Consistent with this, in the revised system (Fig. R53), although FEC is far more abundant than TMSFS in the simulation box, the integrated first-shell molecular CN values are CN(FEC) = 0.560 and CN(TMSFS) = 0.194 (ratio ≈ 2.9), corresponding to a coordination fraction α(TMSFS) ≈ 0.99. Upon further increasing the TMSFS content to 5 wt.% and 10 wt.%, CN(TMSFS) increases to 0.820 and 1.389, respectively. This indicates that TMSFS is strongly enriched in the Li^+ inner solvation sheath relative to its bulk concentration.

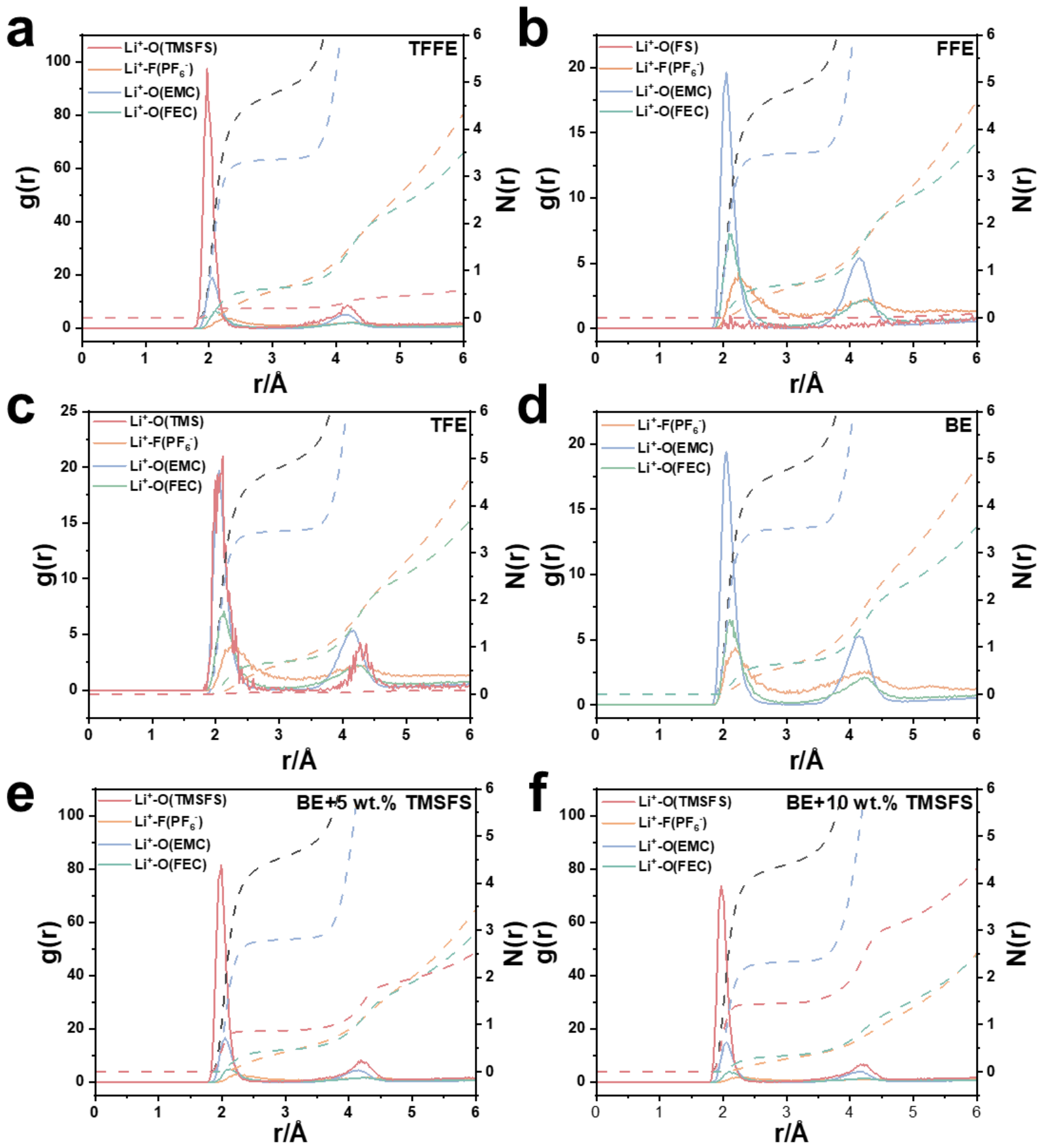

Fig. R53. RDFs of key species coordinating with Li^+ in (a) TFFE, (b) FFE, (c) TFE, (d) BE systems, (e) BE+5 wt.% TMSFS and (f) BE+10 wt.%TMSFS.

We have rechecked the calculation workflow and confirmed that the CN values reported in the revision are obtained using the consistent cutoff and proper normalization. In the revised manuscript, we have removed/replaced the previous coordination numbers that summed to ~3.5 and updated all related text/figures. We have added a concise description of the CN calculation method (first-minimum cutoff and molecular-based counting) and the validated box density:

The simulations were performed in a cubic simulation box with an edge length of 60.46 Å. For the TFFE system, the simulation box contained 133 $LiPF_6$ molecules, 982 EMC molecules, 348 FEC molecules, and 13 TMSFS molecules, corresponding to 1 M LiPF6 in EMC: FEC = 4:1 (by volume) with 1 wt.% TMSFS. The other electrolyte systems (BE, FFE, and TFE) were constructed analogously at the same salt concentration and solvent ratio, with or without the corresponding additive. A blank control system without any additive was also simulated.

Fig. R53 *were applied as Fig. 2b, c, d, e and Supplementary Figure 4.*

7. Please indicate the electrolyte injection amount in the figure, so that readers can better understand the performance level of the cells reported by the authors. Please check the presentation of units throughout the manuscript. For example, in expressions such as "1 M," there should be a space between the number and the unit ("1 M," not "1M").

Response: Thanks for the reviewer's suggestion. We have added the electrolyte injection amount in the main text (Fig. R54) and the supplementary materials (Table Supplementary Table 9 and 10), as well as checked and revised the units.

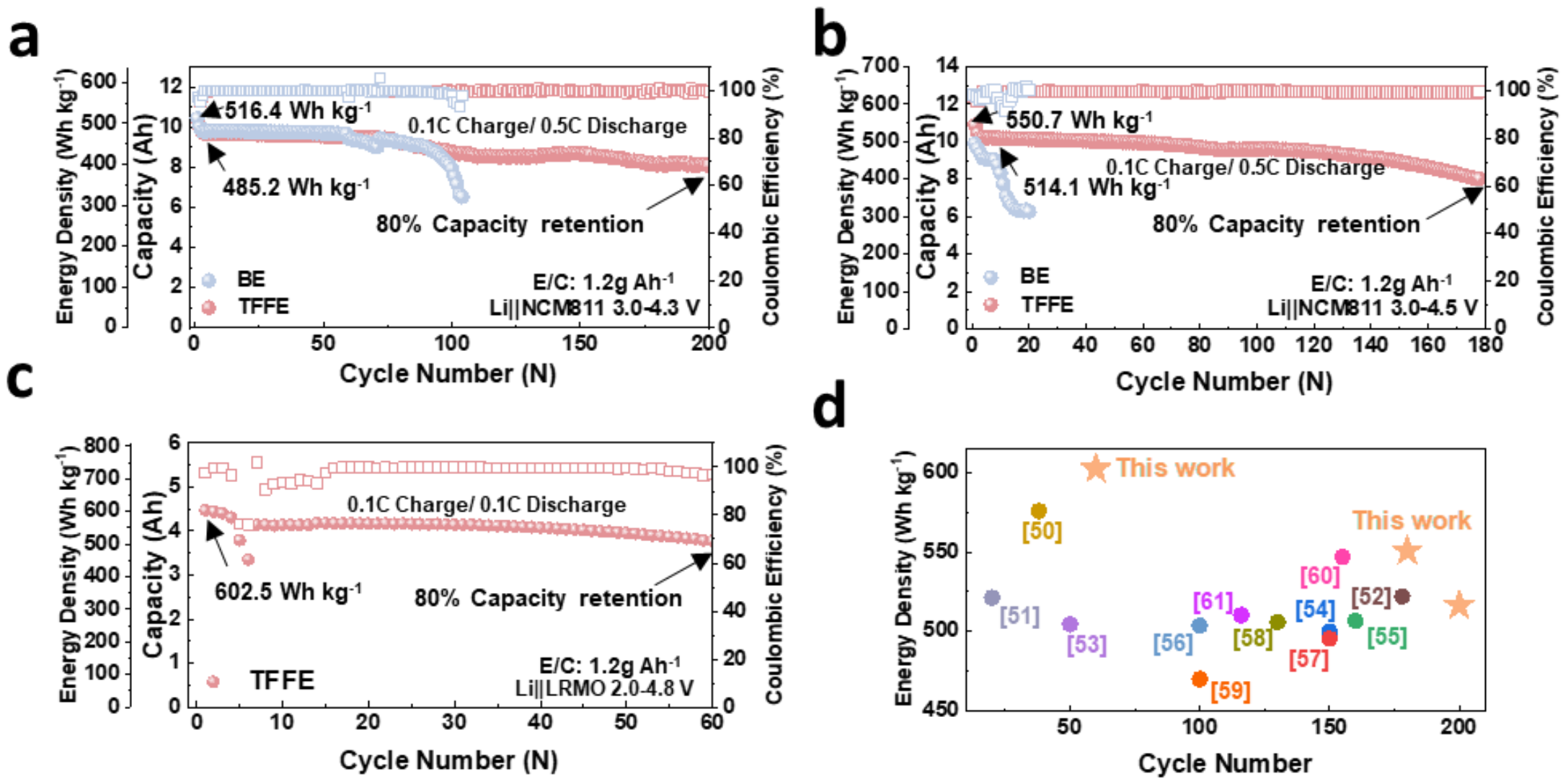

Fig. R54 Cycling performance comparison of Li||NCM811 pouch cells assembled with BE and TFFE electrolytes (a) from 3.0 to 4.3 V (b) from 3.0 to 4.5V; (c) Cycling performance of Li||LRMO pouch cells assembled with TFFE electrolytes from 2.0 to 4.8 V; (d) Energy density and cycle life benchmarking of the LMB pouch cell in this work compared to recently reported advanced electrolyte systems. (Energy density was calculated from the first-cycle discharge at 0.1 C).

Table R9. The specifications of the NCM811 pouch cell

Cell component	specification	Parameters
	Number	17
Cathode	Specific areal capacity (mAh cm^{-2}, each side)	4.2
(NCM811)	Size (cm^2)	7.5*9.5
	Thickness of Al foil (μm)	10
	Number	18
Anode	Size (cm^2)	7.7*9.7
	Thickness of Li foil (μm)	100
Electrolyte	Injection factor (g Ah^{-1})	1.2
Separator	Thickness (μm)	16
Package and	Weight (g)	2.8

tabs		
Full cell	Capacity at 4.3V/4.5V (Ah)	10.4/10.9
	Weight (g)	76.2
	Energy density at 4.3 V (Wh kg^{-1})	516.4
	Energy density at 4.5 V (Wh kg^{-1})	550.7

Table R10. The specifications of the LRMO pouch cell.

Cell component	specification	Parameters
Cathode (LRMO)	Number	10
	Specific areal capacity (mAh cm^{-2}, each side)	5.4
	Size (cm^2)	5.7*7.5
	Thickness of Al foil (μm)	12
Anode	Number	11
	Size (cm^2)	5.9*7.7
	Thickness of Li foil (μm)	50
Electrolyte	Injection factor (g Ah^{-1})	1.2
Separator	Thickness (μm)	16
Package and tabs	Weight (g)	1.9
Full cell	Capacity (Ah)	4.5
	Weight (g)	26.3
	Energy density (Wh kg^{-1})	602.5

The changes of the above aspects have been supplemented in the revised manuscript highlighted with yellow, which can also be found below:

All energy density values were calculated based on the total cell mass, including packaging materials and tabs, with an electrolyte injection amount of 1.2 g Ah^{-1}. And pouch cells were evaluated at different discharge rates, from which a moderate rate (<0.5C) was identified as the most suitable testing condition for reliable performance assessment (Supplementary Fig. S42).

***Fig. R54** were applied as Fig. 6b, c, d and e.*

***Table R9 and 10** were applied as Supplementary Table 4 and Supplementary Table 5.*

Reference:

1 Xia, M. et al. Quantitative and Non-Destructive Analysis of Failure Process of Pouch Lithium Metal Batteries. Advanced Energy Materials 15 (2025).

2 Chen, Z.-X. et al. Sufficient cathode infiltration for stable 500 Wh kg^{-1} level lithium–sulfur batteries. Journal of Energy Chemistry 109, 129-137 (2025).

3 Cheng, Q. et al. Deciphering the Degradation Mechanism of High-Rate and High-Energy-Density Lithium–Sulfur Pouch Cells. Advanced Energy Materials 13 (2023).

4 Kwon, H., Baek, J. & Kim, H.-T. Building lithium metal batteries under lean electrolyte conditions: Challenges and progress. Energy Storage Materials 55, 708-726 (2023).

5 Lechtenfeld, C. T. et al. Analyzing the Effect of Electrolyte Quantity on the Aging of Lithium-Ion Batteries. Advanced Science 11 (2024).

6 Niu, C. et al. Balancing interfacial reactions to achieve long cycle life in high-energy lithium metal batteries. Nature Energy 6, 723-732 (2021).

7 Su, C. C. et al. Solvation Rule for Solid-Electrolyte Interphase Enabler in Lithium-Metal Batteries. Angewandte Chemie International Edition 59, 18229-18233 (2020).

8 Jang, J. et al. Miniature Li^+ solvation by symmetric molecular design for practical and safe Li-metal batteries. Nature Energy 10, 502-512 (2025).

9 Yang, Y., Yang, W., Yang, H. & Zhou, H. Electrolyte design principles for low-temperature lithium-ion batteries. eScience 3 (2023).

10 Lu, Y., Korf, K., Kambe, Y., Tu, Z. & Archer, L. A. Ionic-liquid-nanoparticle hybrid electrolytes: applications in lithium metal batteries. Angew Chem Int Ed Engl 53, 488-492 (2014).

11 Wu, H., Zhuo, D., Kong, D. & Cui, Y. Improving battery safety by early detection of internal shorting with a bifunctional separator. Nat Commun 5, 5193 (2014).

12 Schomburg, F. et al. Lithium-ion battery cell formation: status and future directions towards a knowledge-based process design. Energy & Environmental Science 17, 2686-2733 (2024).

13 Ding, X. et al. An Ultra-Long-Life Lithium-Rich $Li_{1.2}Mn_{0.6}Ni_{0.2}O_2$ Cathode by Three-in-One Surface Modification for Lithium-Ion Batteries. Angew Chem Int Ed Engl 59, 7778-7782 (2020).

14 Zhang, H. et al. Enhancing Anionic Redox Reversibility and Structural Stability of Li-Rich Mn-Based Cathodes via Tuning Band Structure by Sn Doping. ACS Nano 19, 41223-41232 (2025).

15 Zhu, X. et al. Tailoring redox couples of Li-rich Mn-based cathode materials by in-situ surface reconstruction for high-performance lithium-ion batteries. Nano Energy 134 (2025).

16 Yang, Y. et al. Removing alpha-H in Carboxylate-Based Electrolytes for Stable Lithium Metal Batteries. Angew Chem Int Ed Engl 64, e202503616 (2025).

17 Chen, S. et al. Single fluorosilane ester additive for F/Si-enriched inorganic interphases enabling durable high-voltage graphite/$LiCoO_2$ pouch cells. Energy Storage Materials 81 (2025).
18 Wang, Y. N. et al. In Situ Polymerized Polysiloxane Enables Cohesive Solid-Electrolyte Interphase for Practical Lithium-Metal Batteries. Adv Mater, e19565 (2025).
19 Chen, H. et al. Uniform High Ionic Conducting Lithium Sulfide Protection Layer for Stable Lithium Metal Anode. Advanced Energy Materials 9 (2019).
20 Wu, X. et al. Integrating Lithium Sulfide as a Single Ionic Conductor Interphase for Stable All-Solid-State Lithium-Sulfur Batteries. Adv Sci (Weinh) 11, e2308604 (2024).
21 Yu, W. et al. Electrochemical formation of bis(fluorosulfonyl)imide-derived solid-electrolyte interphase at Li-metal potential. Nat Chem 17, 246-255 (2025).
22 Ou, X. et al. Enabling high energy lithium metal batteries via single-crystal Ni-rich cathode material co-doping strategy. Nat Commun 13, 2319 (2022).
23 Su, H. et al. Achieving Practical High-Energy-Density Lithium-Metal Batteries by a Dual-Anion Regulated Electrolyte. Adv Mater 35, e2301171 (2023).
24 Chen, Q. et al. Macroscopically uniform interface layer with Li^+ conductive channels for high-performance Li metal batteries. Nat Commun 15, 10045 (2024).
25 Jie, Y. et al. Towards long-life 500 Wh kg^{-1} lithium metal pouch cells via compact ion-pair aggregate electrolytes. Nature Energy 9, 987-998 (2024).
26 Liu, X. et al. 570 Wh kg^{-1}-Grade Lithium Metal Pouch Cell with 4.9V Highly Li^+ Conductive Armor-Like Cathode Electrolyte Interphase via Partially Fluorinated Electrolyte Engineering. Adv Mater 36, e2401505 (2024).
27 Ji, H. et al. Liquid-liquid interfacial tension stabilized Li-metal batteries. Nature 643, 1255-1262 (2025).
28 Qiao, R. et al. Non-fluorinated electrolytes with micelle-like solvation for ultra-high-energy-density lithium metal batteries. Chem 11 (2025).
29 Tang, T. et al. Long-Lifespan 522 Wh kg^{-1} Lithium Metal Pouch Cell Enabled by Compound Additives Engineering. Angew Chem Int Ed Engl 64, e202417471 (2025).
30 Wu, L. Q. et al. Regulating Amine Substitution in Fluorosulfonyl-Based Flame-Retardant Electrolytes for Energy-Dense Lithium Metal Batteries. J Am Chem Soc 147, 16506-16521 (2025).
31 Yang, W. et al. A Bipolar Solvent Molecule Design for Wide-Temperature High-Voltage Lithium Metal Batteries. Adv Mater 37, e2505285 (2025).
32 Yang, W., Chen, A., He, P. & Zhou, H. Advancing lithium metal electrode beyond 99.9% coulombic efficiency via super-saturated electrolyte with compressed solvation structure. Nat Commun 16, 4229 (2025).
33 Eldesoky, A., Louli, A. J., Benson, A. & Dahn, J. R. Cycling Performance of NMC811 Anode-Free Pouch Cells with 65 Different Electrolyte Formulations. Journal of The Electrochemical Society 168 (2021).

34 Jiang, Z. et al. Achieving a stable 518 Wh kg^{-1} Li metal pouch cell via SEI reconstruction engineering for high Li+ conductive hetero-grain boundaries. Energy & Environmental Science 18, 9240-9253 (2025).
35 Kim, M. S. et al. Langmuir–Blodgett artificial solid-electrolyte interphases for practical lithium metal batteries. Nature Energy 3, 889-898 (2018).
36 Pokharel, J. et al. Manipulating the diffusion energy barrier at the lithium metal electrolyte interface for dendrite-free long-life batteries. Nature Communications 15 (2024).
37 Dodda, L. S., Vilseck, J. Z., Tirado-Rives, J. & Jorgensen, W. L. 1.14*CM1A-LBCC: Localized Bond-Charge Corrected CM1A Charges for Condensed-Phase Simulations. The Journal of Physical Chemistry B 121, 3864-3870 (2017).
38 Leontyev, I. & Stuchebrukhov, A. Accounting for electronic polarization in non-polarizable force fields. Physical Chemistry Chemical Physics 13 (2011).
39 Liang, D., Chen, Y., Deng, C. & de Pablo, J. J. Charge Scaling in Classical Force Fields for Lithium Ions in Polymers. ACS Macro Letters 13, 1258-1264 (2024).
40 Dawass, N. et al. Finite-size effects of Kirkwood–Buff integrals from molecular simulations. Molecular Simulation 44, 599-612 (2017).

We thank the referees for the helpful and positive comments and have revised the manuscript accordingly. In this point-to-point response letter, comments from the referees are in black typeface, and our responses are in the blue typeface. Major changes have been highlighted in the revised manuscript.

Reviewer: 1:

The authors have satisfactorily addressed all the concerns and comments raised in my previous review. I have no further comments at this stage and recommend the manuscript for publication.

Response: Thanks for the reviewer's comment regarding positive evaluation of our manuscript. We greatly appreciate the insightful comments and constructive suggestions provided during the review process, which have significantly helped improve the quality and clarity of this work.

Reviewer: 2:

Most of questions are revised, therefore I recommend this draft to be published in this journal.

Response: Thanks for the reviewer's comment regarding positive evaluation of our manuscript. We greatly appreciate the insightful comments and constructive suggestions provided during the review process, which have significantly helped improve the quality and clarity of this work.

Reviewer: 4

Comments:

The authors have provided a comprehensive and generally satisfactory response. However, I would like to raise two additional comments:

Response: Thanks for the reviewer's comment regarding positive evaluation of our manuscript. We greatly acknowledging that our previous response was comprehensive and generally satisfactory. In addition, we have carefully considered the two supplementary comments raised by the reviewer, and our point-by-point responses are provided below.

1. Concerning the interpretation of self-diffusion coefficients in relation to coordination ratios.

There appears to be an issue in using self-diffusion coefficients to quantitatively infer coordination proportions. The authors did not clearly specify how the coordination ratio was calculated. It should be noted that ***upon salt addition, the viscosity of the system typically increases****. As a result, the diffusion coefficients of all species decrease regardless of whether they participate in coordination. Therefore, the observed reduction in diffusion coefficients can at most provide qualitative evidence for changes in coordination behavior, rather than a quantitative determination of coordination ratios. A more* ***rigorous analysis*** *or complementary structural evidence would be necessary to* ***substantiate such claims****.*

Response: Thanks for the reviewer's comment regarding the limitation of using absolute diffusion coefficients to infer coordination behavior. As the reviewer pointed out, the addition of lithium salt increases the viscosity of the electrolyte, which leads to a general decrease in diffusion coefficients for all species. Therefore, absolute diffusion coefficients alone cannot be directly used to quantitatively determine coordination ratios.

Hence, in this work, our analysis is inspired by previous studies (e.g., Khalil Amine et al., Energy Environ. Sci.,2019, 12, 1249 and Angew. Chem. Int. Ed. 2020, 59, 18229-18233), where 2D DOSY techniques were employed to probe solvation structures and relative coordination behaviors in electrolyte systems. The fundamental principle of these approaches is that species participating in the same solvation complex exhibit coupled translational motion and therefore similar diffusion coefficients, whereas non-coordinating or weakly interacting species retain distinct and faster diffusion behavior. As a result, relative diffusion characteristics, rather than absolute values, can be used to distinguish coordinated and free species and to evaluate their relative participation in the solvation structure.

Specifically, we employed toluene as an internal reference to normalize the viscosity effect based on the Stokes-Einstein relation. According to the Stokes-Einstein equation, the diffusion coefficient of toluene can be described as eq. 1.

$$D_{1T} = \frac{kT_1}{6\pi\eta_1 r_1} \quad (1)$$

Owing to toluene's low polarity and low donor number (DN), toluene hardly participates in the coordination of Li ions. Consequently, its hydrodynamic radius (r) is expected to remain unchanged upon the addition of lithium salt and the ratio of diffusion coefficients before and after salt addition reflects the change in viscosity of the system. Based on this principle, eq. (2) can be established as valid. By normalizing the diffusion coefficients of solvent molecules with respect to toluene, the viscosity contribution can be effectively eliminated, allowing us to extract the relative changes in diffusion behavior associated with coordination.[1-3]

$$\frac{D_{1T}}{D_{2T}} = \frac{kT_1}{6\pi\eta_1 r_1} / \frac{kT_2}{6\pi\eta_2 r_2} = \frac{T_1\eta_2}{T_2\eta_1} \tag{2}$$

Where D_{1T} and D_{2T} are respectively the diffusion coefficient of toluene before and after the addition of $LiPF_6$, T_1 and T_2 are respectively the temperature of the FEC: EMC=1:4 solution before and after the addition of $LiPF_6$, and the temperature was carefully controlled to remain constant during all measurements, η_1 and η_2 are the respective viscosity, k is the Boltzmann constant, and r_1 and r_2 represents the respective hydrodynamic radius.

The solvated solvent is supposed to present a similar diffusion coefficient as Li^+, as represented in eq. (3). Based on approximately the same hydrodynamic diameter ($D_{1solvent}$) of the non-coordinating solvent before and after the $LiPF_6$ addition, we estimate the non-coordinating free solvent's diffusion coefficient ($D_{free\ slovent}$) in the salted electrolyte as follows (eq. (4)).

The diffusion coefficient of solvent measured in the salted electrolyte $D_{2solvent}$ represents the average of the diffusion coefficients of coordinated solvent ($D_{Co\text{-}solvent}$) and free solvent ($D_{free\ solvent}$), as described in eq. (5), where α denotes the coordination ratio of solvent.

$$D_{Li} = D_{Co\ slvent} \tag{3}$$

$$D_{free\ solvent} = \left(\frac{\frac{kT_2}{6\pi\eta_2 r_2}}{\frac{kT_1}{6\pi\eta_1 r_1}}\right) D_{1solvent} = \frac{T_2\eta_1}{T_1\eta_2} D_{1solvent} \tag{4}$$

$$D_{2solvent} = D_{Co\ solvent}\alpha + D_{free\ solvent}(1-\alpha) \tag{5}$$

Using eqs. 1 to 5, the coordination percentage (α) of solvent can be calculated. Thus, the coordination ratio (α) derived here should therefore be understood as an apparent or

effective coordination fraction based on relative diffusion behavior, rather than an absolute structural population.

Therefore, the DOSY analysis is used in combination with complementary evidence (e.g., MD simulations / NMR shifts / Raman / etc.) to support the proposed coordination behavior.

By utilizing the method above and toluene as an internal standard to normalize the viscosity effect, the 2D-DOSY spectra before and after salt addition were measured. The 2D ^{1}H NMR results reveal that upon the addition of the TMSFS, the as-calculated coordination ratio (α) of EMC and FEC decrease from 0.44 and 0.31 in BE to 0.39 and 0.28 in TFFE, respectively. Meanwhile, TMSFS exhibits a markedly high coordination fraction of 0.975. These results further substantiate the strong Li^{+}-coordinating capability of TMSFS and its effectiveness in attenuating solvent–Li^{+} coordination. (Fig. R1).

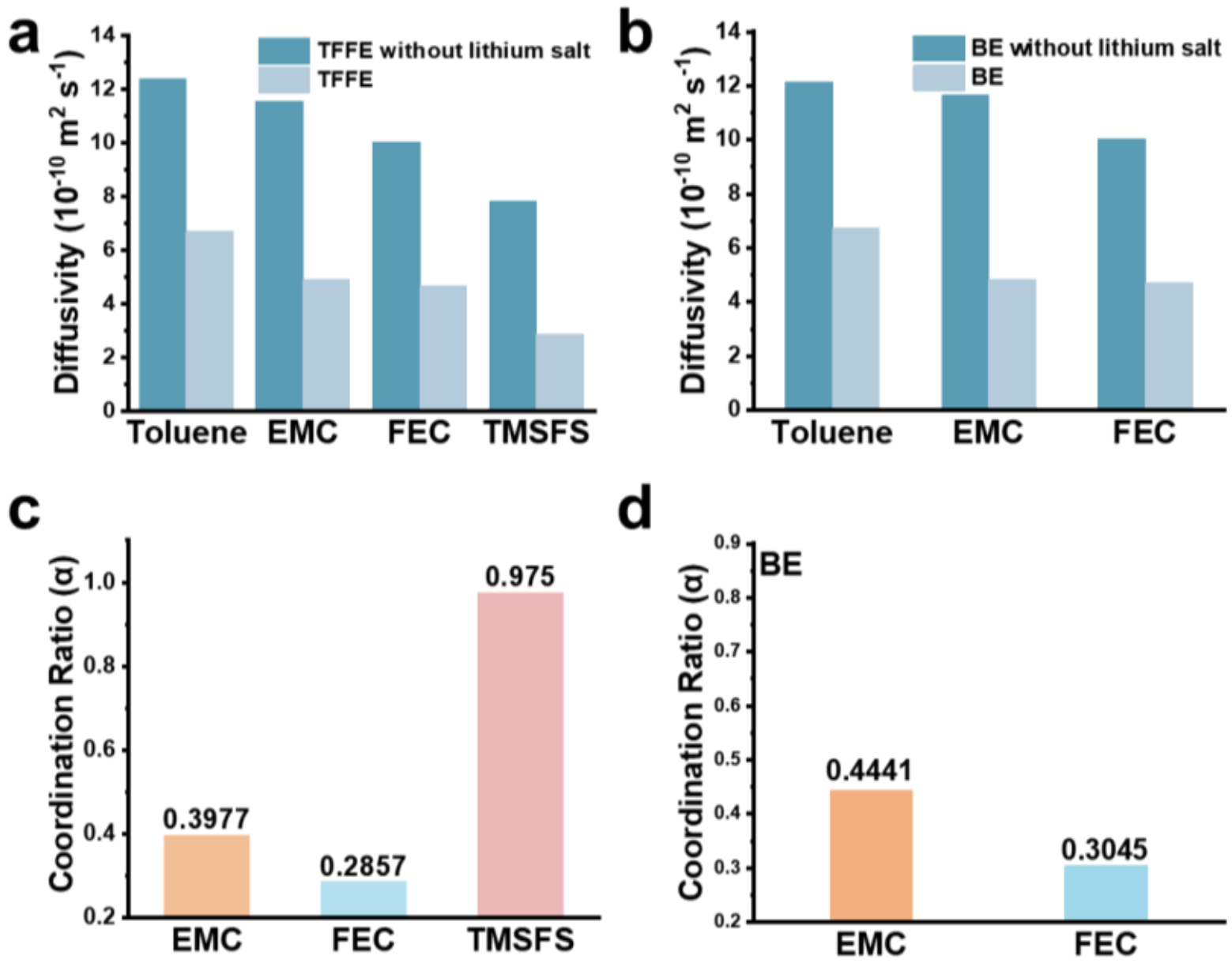

Fig. R1. Diffusion-ordered spectroscopy NMR (DOSY-NMR) analysis. The diffusivities of multiple components (toluene, EMC, FEC, TMSFS,) in (a)TFFE and TFFE without $LiPF_6$ and (b)BE and BE without $LiPF_6$; Corresponding coordination ratios calculated through DOSY of (c)TFFE and (d)BE. Toluene was used as an internal reference.

The changes of the above aspects have been supplemented in the revised manuscript

highlighted with yellow, which can also be found below:

The Diffusion-ordered spectroscopy (DOSY) was performed using the NMR tube with an external coaxial insert. For the experiment, DMSO-d6 was put into the coaxial insert, and the mixture of electrolytes and toluene (internal standard) was out into the outer NMR tube. According to the Stokes-Einstein equation, the diffusion coefficient of tolune can be described as eq. 2.

$$D_{1T} = \frac{kT_1}{6\pi\eta_1 r_1} \quad (2)$$

Owing to its low polarity and low donor number (DN), toluene hardly participates in the coordination of Li ions. Consequently, its hydrodynamic radius (r) is expected to remain unchanged upon the addition of lithium salt. therefore, Equation 3 holds.

$$\frac{D_{1T}}{D_{2T}} = \frac{kT_1}{6\pi\eta_1 r_1} / \frac{kT_2}{6\pi\eta_2 r_2} \quad (3)$$

Where D1T and D2T are respectively the diffusion coefficient of toluene before and after the addition of LiPF6, T1 and T2 are respectively the temperature of the FEC: EMC=1:4 solution before and after the addition of LiPF6, and the temperature was carefully controlled to remain constant during all measurements, $\eta 1$ and $\eta 2$ are the respective viscosity, k is the Boltzmann constant, and r1 and r2 represents the respective hydrodynamic radius.

The solvated solvent is supposed to present a similar diffusion coefficient as Li^+, as represented in eq. 4. Based on approximately the same hydrodynamic diameter (D1solvent) of the non-coordinating solvent before and after the LiPF6 addition, we estimate the non-coordinating free solvent's diffusion coefficient (Dfree slovent) in the salted electrolyte as follows (eq. (5)). The diffusion coefficient of solvent measured in the salted electrolyte D2solvent represents the average of the diffusion coefficients of coordinated solvent (DCo-solvent) and free solvent (Dfree solvent), as described in Eq. 6, where α denotes the coordination ratio of solvent.

$$D_{Li} = D_{Co\ slvent} \quad (4)$$

$$D_{free\ solvent} = \left(\frac{\frac{kT_2}{6\pi\eta_2 r_2}}{\frac{kT_1}{6\pi\eta_1 r_1}}\right) D_{1solvent} = \frac{T_2\eta_1}{T_1\eta_2} D_{1solvent} \quad (5)$$

$$D_{2solvent} = D_{Co\ solvent}\,\alpha + D_{free\ solvent}(1-\alpha) \quad (6)$$

Using eqs. 2 to 6, the coordination percentage (α) of solvents can be calculated.

2. Regarding the choice of force field in MD simulations.

Although polarizable force fields can, in principle, improve the physical realism of charge distributions, they do not necessarily offer clear advantages over conventional force fields in terms of applicability range, methodological complexity, accessible system size, or computational cost. For current electrolyte systems, well-established and validated simulation protocols already exist and have demonstrated reliable

accuracy in predicting monovalent ion coordination numbers, system density, viscosity, and related physicochemical properties. In this regard, I would recommend the use of the OPLS-AA/L force field in combination with 1.14CM1A-LBCC charges, with appropriate charge scaling applied when necessary. Nevertheless, it should also be acknowledged that each methodology has its own limitations, and based on the results presented, the force field currently employed by the authors appears to be acceptable.

Response: Thanks for the reviewer's valuable suggestion. We agree that OPLS-AA/L combined with 1.14*CM1A-LBCC charges, together with appropriate charge scaling, represents a well-established and practical fixed-charge protocol for electrolyte simulations. In the present work, however, our MD model also follows a fixed-charge framework: we employed the OPLS-AA force field, and the partial atomic charges of the molecular species were obtained by RESP fitting to the DFT electrostatic potential and then uniformly scaled by an electronic continuum correction (ECC) scheme to partially account for the missing electronic polarization. RESP-based OPLS-AA parameterizations have previously been adopted in MD studies of carbonate/LiPF6 electrolytes. For example, Hou et al. used non-polarizable OPLS-AA force fields with RESP-fitted charges for $LiPF_6$/EC/FEC and mixed-carbonate $LiPF_6$/EC/EMC/FEC systems, and benchmarked the simulated solvation structures and transport trends against experimental measurements, including FTIR, IR-DOSY, and conductivity data[4,5]. We therefore believe that the present RESP+ECC/OPLS-AA treatment is appropriate for the comparative solvation analysis performed here.

Importantly, the purpose of the MD simulations in this study is to reveal the relative change in the Li^+ solvation environment induced by TMSFS, rather than to establish a uniquely optimal force-field parametrization or an exact prediction of every bulk property. The central MD-derived trends are independently corroborated by our experiment results, all of which support the enhanced participation of TMSFS in the inner solvation sheath and the concomitant reduction of solvent/anion coordination.

Furthermore, the radial distribution function (RDF) results at different additive concentrations further validate this purpose (Fig. R2). At various TMSFS concentrations, the RDF profiles consistently show a marked increase in the

coordination of TMSFS with Li^+, highlighting a strong and consistent trend of TMSFS's enhanced involvement in the solvation structure. These RDF results, in conjunction with the other spectroscopic analyses, confirm that TMSFS not only participates more significantly in the inner solvation sheath but also leads to a reduction in the solvent/anion coordination, reinforcing the efficiency of the additive across varying concentrations.

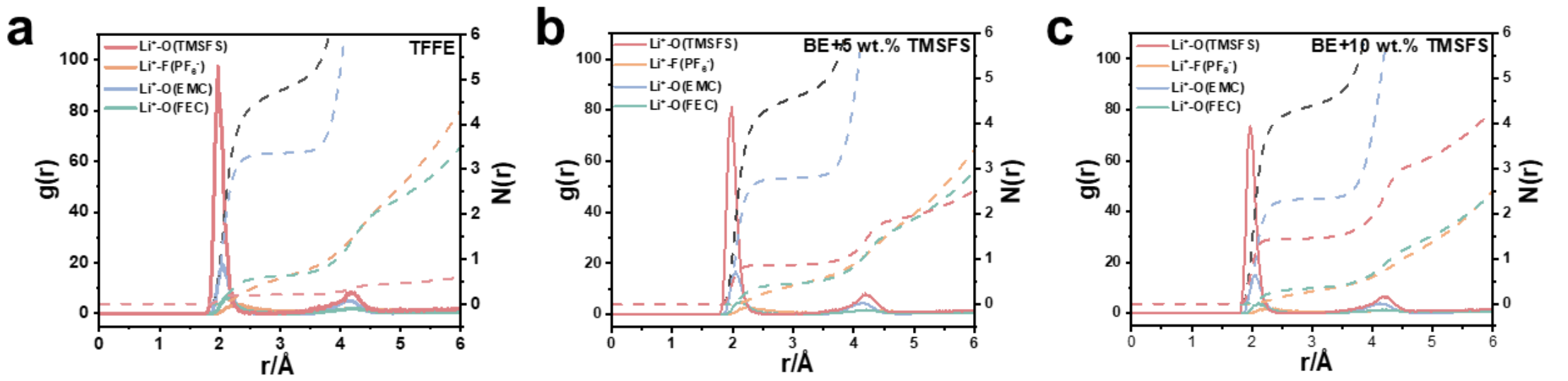

Fig. R2. Diffusion-ordered spectroscopy NMR (DOSY-NMR) analysis. RDFs of key species coordinating with Li^+ in (a) BE+1 wt.% TMSFS (TFFE); (b) BE+5 wt.% TMSFS, and (c) BE+10 wt.% TMSFS.

In response to the reviewer's suggestion, we have revised the Methods section to clarify the force-field description and to note prior literature precedent for RESP-based OPLS-AA simulations of LiPF6/carbonate electrolytes. We also acknowledge that alternative fixed-charge parametrizations, including OPLS-AA/L with 1.14*CM1A-LBCC charges, are reasonable alternatives for such systems.

The changes of the above aspects have been supplemented in the revised manuscript highlighted with yellow, which can also be found below:

Our MD model uses a fixed-charge framework with the OPLS-AA force field, where partial atomic charges are derived from RESP fitting to the DFT electrostatic potential and scaled by an electronic continuum correction (ECC) to account for missing electronic polarization.

All interactions were described using the OPLS-AA (Optimized Potentials for Liquid Simulations - All Atom) force field. The partial atomic charges for all molecules were obtained from RESP fitting to the DFT electrostatic potential and uniformly scaled following the electronic continuum correction (ECC) scheme, as also adopted in prior OPLS-AA MD studies of $LiPF_6$/carbonate electrolytes.

Reference:

1 Jang, J. *et al*. Miniature Li^+ solvation by symmetric molecular design for practical and safe Li-metal batteries. *Nat. Energy* 10, 502-512 (2025).

2 Su, C.-C. *et al*. Solvating power series of electrolyte solvents for lithium batteries. *Energy Environ. Sci.* 12, 1249-1254 (2019).

3 Su, C. C. *et al.* Solvation Rule for Solid-Electrolyte Interphase Enabler in Lithium-Metal Batteries. *Angew. Chem. Int. Ed.* 59, 18229-18233 (2020).

4 Hou, T. *et al*. A. Correction: The solvation structure, transport properties and reduction behavior of carbonate-based electrolytes of lithium-ion batteries. Chem Sci 13, 8205 (2022).

5 Hou, T. *et al*. The influence of FEC on the solvation structure and reduction reaction of $LiPF_6$/EC electrolytes and its implication for solid electrolyte interphase formation. *Nano Energy* 64 (2019).

We thank the referees for the helpful and positive comments and have revised the manuscript accordingly. In this point-to-point response letter, comments from the referees are in black typeface, and our responses are in the blue typeface. Major changes have been highlighted in the revised manuscript.

Reviewer #4 (Remarks to the Author):

The authors have adequately addressed all the issues and comments raised in my previous review. I have no further remarks at this stage and recommend the manuscript for publication.

Response: Thanks for the reviewer's comment regarding positive evaluation of our manuscript. We greatly appreciate the insightful comments and constructive suggestions provided during the review process, which have significantly helped improve the quality and clarity of this work.
